# PI3K/AKT/mTOR signaling transduction pathway and targeted therapies in cancer

**DOI:** 10.1186/s12943-023-01827-6

**Published:** 2023-08-18

**Authors:** Antonino Glaviano, Aaron S. C. Foo, Hiu Y. Lam, Kenneth C. H. Yap, William Jacot, Robert H. Jones, Huiyan Eng, Madhumathy G. Nair, Pooyan Makvandi, Birgit Geoerger, Matthew H. Kulke, Richard D. Baird, Jyothi S. Prabhu, Daniela Carbone, Camilla Pecoraro, Daniel B. L. Teh, Gautam Sethi, Vincenzo Cavalieri, Kevin H. Lin, Nathalie R. Javidi-Sharifi, Eneda Toska, Matthew S. Davids, Jennifer R. Brown, Patrizia Diana, Justin Stebbing, David A. Fruman, Alan P. Kumar

**Affiliations:** 1 Department of Biological, Chemical and Pharmaceutical Sciences and Technologies, University of Palermo, 90123 Palermo, Italy; 2 Department of Surgery, National University Hospital Singapore, National University of Singapore, Singapore, Singapore; 3 Department of Pharmacology, Yong Loo Lin School of Medicine, National University of Singapore, Singapore, 117600 Singapore; 4 NUS Centre for Cancer Research (N2CR), Yong Loo Lin School of Medicine, National University of Singapore, Singapore, 119077 Singapore; 5 Department of Medical Oncology, Institut du Cancer de Montpellier, Inserm U1194, Montpellier University, Montpellier, France; 6 Cardiff University and Velindre Cancer Centre, Museum Avenue, Cardiff, CF10 3AX UK; 7 Division of Molecular Medicine, St. John’s Research Institute, St. John’s Medical College, Bangalore, 560034 India; 8 The Quzhou Affiliated Hospital of Wenzhou Medical University, Quzhou People’s Hospital, Quzhou, 324000 Zhejiang China; 9 Department of Pediatric and Adolescent Oncology, Gustave Roussy Cancer Center, Inserm U1015, Université Paris-Saclay, Paris, France; 10 Section of Hematology and Medical Oncology, Boston University and Boston Medical Center, Boston, MA USA; 11 Cancer Research UK Cambridge Centre, Hills Road, Cambridge, CB2 0QQ UK; 12 Departments of Ophthalmology and Anatomy, Yong Loo Lin School of Medicine, National University of Singapore, and Neurobiology Programme, National University of Singapore, Singapore, Singapore; 13 Dana-Farber Cancer Institute, Harvard Medical School, Boston, MA USA; 14 Department of Biochemistry and Molecular Biology, Johns Hopkins School of Public Health, Baltimore, MD USA; 15 Division of Cancer, Imperial College London, Hammersmith Campus, Du Cane Road, London, W12 0NN UK; 16 Department of Molecular Biology and Biochemistry, University of California, 216 Sprague Hall, Irvine, CA USA

**Keywords:** PI3K/AKT/mTORC pathway, Pan PI3K inhibitors, Isoform-specific PI3K inhibitors, Dual PI3K/mTOR inhibitors, AKT inhibitors, Allosteric mTOR inhibitors, ATP-competitive mTOR inhibitors, Bi-steric mTOR inhibitors, PDK1 inhibitors, Cancer

## Abstract

The PI3K/AKT/mTOR (PAM) signaling pathway is a highly conserved signal transduction network in eukaryotic cells that promotes cell survival, cell growth, and cell cycle progression. Growth factor signalling to transcription factors in the PAM axis is highly regulated by multiple cross-interactions with several other signaling pathways, and dysregulation of signal transduction can predispose to cancer development. The PAM axis is the most frequently activated signaling pathway in human cancer and is often implicated in resistance to anticancer therapies. Dysfunction of components of this pathway such as hyperactivity of PI3K, loss of function of PTEN, and gain-of-function of AKT, are notorious drivers of treatment resistance and disease progression in cancer. In this review we highlight the major dysregulations in the PAM signaling pathway in cancer, and discuss the results of PI3K, AKT and mTOR inhibitors as monotherapy and in co-administation with other antineoplastic agents in clinical trials as a strategy for overcoming treatment resistance. Finally, the major mechanisms of resistance to PAM signaling targeted therapies, including PAM signaling in immunology and immunotherapies are also discussed.

**Supplementary Information:**

The online version contains supplementary material available at 10.1186/s12943-023-01827-6.

## The PAM pathway in cancer

### Introduction

The PAM signaling pathway is a highly conserved major transduction network in all higher eukaryotic cells that promotes cell survival, growth, and proliferation in response to external stimuli [1–3]. The two major functional proteins in this pathway are PI3K and AKT [4–7]. Importantly, external growth factors signaling to transcription factors in the PAM pathway is a highly regulated process involving extensive cross-talk with other cell signaling networks [8–10]. Dysregulation of the PAM pathway is known to drive cancer development and progression [11–13]. Indeed, PAM pathway aberration occurs in approximately 50% of tumors [14], and is the most commonly activated pathway in human cancer [15–17]. In addition, PAM pathway hyperactivation in cancer frequently underpins the development of treatment resistance [11, 14, 18]. Aberrant expression or mutation of many components of this pathway are known to be related to human oncogenesis [19, 20]. Thence, activation of membrane receptors (RTK or GPCR), induction of oncogenes upstream of PI3K [21], mutations or amplifications of kinases such as PIK3CA, reduced expression or inactivation of tumor suppressor PTEN [22], and/or mutations such as amplification and gain-of-function missense mutations in AKT oncogene [23], can possibly lead to the onset and/or progression of cancer [11, 24, 25]. Furthermore, overactivity of PAM pathway also promotes epithelial-mesenchymal transition (EMT) and metastasis through its remarkable impact on cell migration [26, 27]. Herein we highlight the biology and biochemistry of the PAM axis, describe its major dysregulations in cancer, show its main crosstalks with other signaling pathways, and discuss PI3K-, AKT-, mTOR-, and PDK1-targeted inhibitors, emphasizing on their mechanisms of therapeutic resistance. Thus, our manuscript is comprehensive on the whole PAM signaling pathway including its major effectors AKT and mTOR in biology and disease. For more detailed reviews on PI3K inhibitors targeting cancer stroma with a focus on immune modulation we refer readers to Okkenhaug et al. 2016 [28], and Vanhaesebroeck et al. 2022 [29]. Additional information on past and future PI3K inhibitors only we refer the reader to Castel et al. 2021 [30]. Moreover, while our manuscript focuses on multiple PI3K-driven cancers, for a specific perspective on the relevance of the PI3K pathway in estrogen receptor (ER) + breast cancer, and the crosstalk between ER and the PI3K pathway in breast cancer have been extensively reviewed by Vasan et al. 2019 [31]. Furthermore, since the present work does not discuss in details the role of PI3K pathway on metabolism, we direct the reader for this specific topic to Vasan and Cantley 2022 [32]. In this review, we first focus on major differences of the PAM axis between normal cells and cancer cells, and then, describe current PI3K, AKT, mTORC1/mTORC2, and PDK1 inhibitors, by summarising active and completed trials in a wide range of cancers.

### PAM signaling in cancer

#### RTK overactivation in cancer

**Fig. 1:**
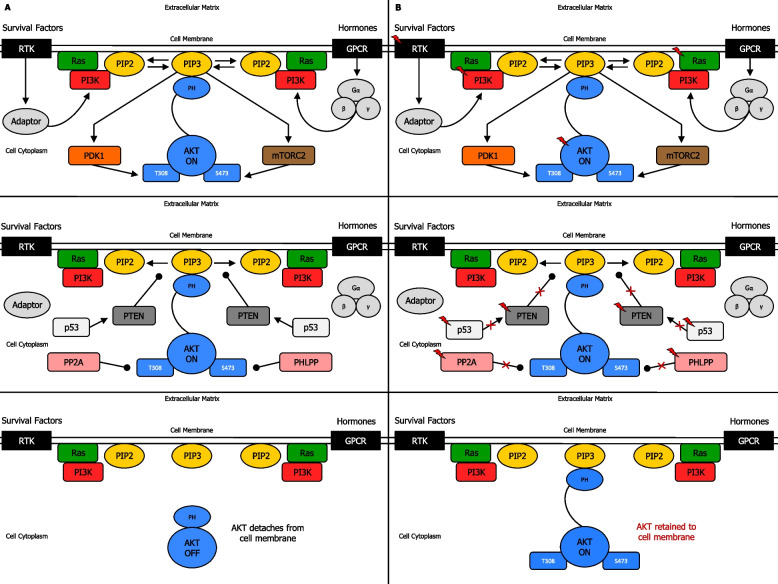
Biochemical mechanism of PI3K, PTEN and AKT regulation. **A** Mechanism of PI3K, PTEN and AKT regulation in normal cells. Induction of RTK or GPCR results in the activation of Ras-regulated PI3K, which interacts with PIP2, and produces PIP3 at the plasma membrane. Inactive AKT in the cytoplasmic matrix is recruited to cell membrane and binds PIP3 through a PH binding domain. This drives phosphorylation of T308 by PDK1, and phosphorylation of S473 by mTORC2, leading to complete activation of AKT (above). Signal termination is determined by loss of PI3K-PIP2 interaction, via inhibition by (PIP3) PTEN protein phosphatase, (AKT) PP2A protein phosphatase, and (AKT) PHLPP protein phosphatase, leading to AKT detaching from the cell membrane. Due to DNA damage response, p53 activates PTEN, whose function reduces PAM-induced cell proliferation (middle). AKT then shifts to off-mode in the cytoplasm (below). **B** Mechanism of PI3K, PTEN and AKT regulation in cancer cells. Mutations in RTK, Ras, PI3K, AKT (above), PTEN protein phosphatase, p53, (AKT) PP2A protein phosphatases and (AKT) PHLPP protein phosphatases may occur, resulting in AKT retention to cell membrane (middle). AKT then remains in on-mode in the cytoplasm (below), leading to dysregulation of PAM pathway signal transduction, and possibly cancer onset and/or progression (below). Activation (phosphorylation or non-phosphorylation) is shown with arrowhead lines, whereas dephosphorylation is indicated with roundhead lines. Red lightning symbol shows mutation for a particular gene in the PAM pathway. Red crosses emphasise signaling blockage. P: phosphoryl group

Growth factor-mediated induction of RTKs [33] or GPCRs [34] usually initiates the canonical pathway that engenders the activation of AKT, resulting in plasma membrane localization and induction of one, or more isoforms of the class I PI3K family [35] (Fig. 1a) (Supplementary information 1). Alterations in epidermal growth factor receptor (EGFR) [36, 37], fibroblast growth factor receptor (FGFR) [38, 39], platelet-derived growth factor receptor (PDGFR) [40, 41], vascular endothelial growth factor receptor (VEGFR) [42, 43], hepatocyte growth factor (HGF) [44, 45], leukocyte receptor tyrosine kinase (LTK) [46, 47], and insulin receptor (INSR) [48, 49] family genes make a significant contribution to treatment failure mainly through the activation of PAM pathway (Fig. 1b) (Supplementary information 1).

#### PI3K mutation and amplification in cancer

Among the numerous types of PI3K, only class I can exert lipid phosphorylation following growth stimulation [50]. PI3K (class I) is a heterodimer comprising two distinct subunits: the regulatory subunit p85, and the catalytic subunit p110 [51, 52]. Specifically, the regulatory subunit p85 of PI3K can bind to phosphorylated tyrosine residues on the activated RTKs through its Src homology 2 (SH2) domain. Subsequently, PI3K catalytic subunit p110 can form a complete active PI3K enzyme [53] (Fig. 1a) (Supplementary information 1). PIK3CA activating mutation, encoding the p110α catalytic subunit of PI3K, is the most commonly mutated oncogene detected across tumor lineages [15, 54]. Indeed, PIK3CA is usually either mutated or amplified in several human cancers [55], including colorectal cancer (CRC) [56], breast [57], lung [58], gastric [59], prostate [60], and cervical cancer [61, 62]. Most mutations are found close to spots E545K (exon 9) and H1047 (exon 20). The hotpots E542K and E545K, present in the helical phosphatidylinositol (inositol phospholipid) kinase homology domain, decrease suppression of p110α by the p85 regulatory subunit, whereas H1047, which is adjacent to the end of the catalytic domain, enhances the p110α-lipid membrane interaction [63, 64]. Mutations in p110β, p110γ and p110δ subunits are rather uncommon; however, their overexpression can easily promote oncogenicity in cultured cells [65]. PIK3CA mutations are present in head and neck squamous cell cancer (HNSCC) [66], gastric cancer [67], gallbladder cancer [68], and melanoma [69]. Besides, mutations of PIK3CA E542K and PIK3CA E545K are known to endorse proliferation and glycolysis of cervical cancer [64]. Also, PIK3CA mutation leads to prostate cancer in mice and correlates with poor prostate cancer prognosis. Notably, PIK3CA mutation and PTEN loss coexist in prostate cancer patients and synergistically can cooperate in vivo to accelerate carcinogenesis and cancer progression via PAM pathway hyperactivation [70, 71]. PIK3CA mutations are frequently associated with FGFR3 mutations in metastatic non-muscle invasive bladder cancer [72]. In line with this, mutations in PAM pathway are detected in 25% of osteosarcoma patients. In fact, PIK3CA and mTOR are critical for survival and proliferation of osteosarcoma cells [73]. Mutations in PI3K family genes, especially PIK3CA or PIK3R1 are often present in glioblastoma multiforme [74], testicular germ cell tumors [75], and Ewing’s sarcoma [76], resulting in alteration pattern of PAM pathway. Mutations of PIK3CA, PIK3R1, and PIK3R2 are frequently detected in small-cell lung cancer (SCLC), non-small-cell lung cancer (NSCLC) [77], hepatocellular cancer [78], and ovarian serous cystadenocarcinoma [79]. Similarly, dysregulation of PAM pathway in CRC is mainly due to mutations in PIK3CA, and to a lesser extent PIK3R1 and PIK3R2. In addition, CRC PIK3CA mutations are generally associated with KRAS mutations [80, 81]. The genetic alterations of PAM pathway in renal cancer are also due to PIK3CA, PIK3R1, and PIK3R2 mutations. Moreover, PIK3R1 can regulate EMT, as well as stem-like phenotype, of renal cell carcinoma cells via the AKT/GSK3β/β-catenin signaling pathway [82, 83]. Oesophageal squamous cell carcinoma present genetic alterations of PI3K family genes and PTEN, especially somatic mutations of PIK3CA, PIK3CG, PIK3C2A, and PIK3C2G [84]. Furthermore, mutations in PAM pathway genes such as PIK3CA, PIK3CG, PIK3C2G, PIK3C3, PIK3R1, and PIK3R2 are often detected in poorly differentiated thyroid cancer and anaplastic thyroid cancer [85]. Thus, mutations in the PAM pathway can affect RTKs and growth factors, as well as Ras and PI3K p110 subunits, resulting in abnormal signaling activity (Fig. 1b).

#### PTEN inactivation or loss in cancer

PI3K/PIP3 signal termination is mainly attained by tumor suppressor PTEN, which exerts dephosphorylation on PIP3, thereby switching it back to PIP2. Thus, PTEN acts as an essential negative regulator of the PAM pathway affecting cell growth survival, whereas loss of PTEN results in the sustained output of these intracellular signalings [86] (Fig. 1a) (Supplementary information 1). PTEN loss-of-function mutations are present in a several tumors [87, 88]. PTEN loss frequently occurs in primary and metastatic breast cancer leading to hyperactivation of PAM pathway, and consequently, enhancing cell proliferation [89, 90]. Both PTEN downregulation and PAM pathway activation are related to anti-estrogen therapy resistance [91]. Mutations in PTEN are often detected in gastric cancer [67]. Besides, overexpressed [92] and/or amplified [93] PRL-3 downregulates the expression of PTEN through dephosphorylation [94], and as a result indirectly increases signals through PAM pathway [95] in human gastric cancer [93]. Mutations in PTEN are observed in CRC too [80]. Notably, loss of TGF-β signalling results in PRL-3 upregulation and PAM pathway activation, which can promote EMT and tumor aggressiveness in primary CRC [96]. Mutations in PTEN are also reported in bladder cancer since loss of PTEN combined with altered TP53 determines a negative effect, enhancing tumor progression [97]. The PAM pathway can also function as a pro-survival factor in leukemia stem cells, and thus, genetic aberrations in PTEN are likely to be detected in leukemia. In fact, PTEN regulates the activity of hematopoietic stem cell via a niche-dependent mechanism, as well as leukemogenesis and hematopoiesis [98]. PTEN loss-of-function alterations, especially deletion, are also detected in brain cancer [87], glioblastoma multiforme [99], anaplastic/poorly differentiated thyroid cancer [85], SCLC, NSCLC [77], melanoma [69], oesophageal cancer [100], gallbladder cancer [68], pancreatic cancer [101], renal cell carcinoma [102], prostate cancer [103], testicular germ cell tumors [75], cervical cancer [104], ovarian cancer [105], and many types of sarcoma [106, 76], leading to the typical pathological effects of PAM pathway (Fig. 1b).

#### AKT overactivation in cancer

Phosphorylated phosphatidylinositol lipids on the inner face of plasma membrane can directly bind intracellular proteins which contain PH or FYVE zinc finger domains. Indeed, PIP3 binds AKT and PDK1, and as a result, they can accumulate near the membrane [107]. Once activated, AKT migrates from plasma membrane to cytoplasm and nucleus, where many substrates are located [108] (Fig. 1a) (Supplementary information 1). Mutations in AKT genes are rather infrequent in human cancers [1]. However, gain-of-function missense mutations and amplification in genes that encode one of the three isoforms of oncogenic protein AKT, known to be implicated in regulating cell survival, proliferation, growth, apoptosis, and glycogen metabolism [109], have been reported [23]. Notably, the most frequent mutation is AKT1 point mutation in the PH domain where glutamic acid at residue 17 is replaced with lysine (E17K) at residue 17, resulting in enhanced activity of AKT1 by inducing its constitutive localization to the plasma membrane [110]. Moreover, other activating mutations include E49K (AKT1) substitution occurring in the PH domain, and G171R (AKT3) substitution occurring in the kinase domain [111]. Indeed, activated p-AKT levels are significantly increased in cancer cell lines due to these point mutations, and levels of p-AKT correlates with sensitivity to AKT inhibition [112]. Furthermore, high-resolution sequencing studies in breast cancer have also reported further somatic variants in the AKT PH domain [113, 114]. However, due to the relative infrequency of AKT mutations, their significance as drivers of oncogenesis has not been thoroughly clarified. In fact, changes in AKT activity normally occur through the activating mutations or amplifications upstream AKT, such as in PIK3CA or in PTEN, growth factor or cytokine receptors, and intracellular oncogenes like Ras, which lead to enhanced expression and activity of one, two or all three isoforms of AKT [1]. Unlike AKT mutations, AKT gene amplifications are more frequent, and have been detected in breast [115], colon, gastric, ovarian, pancreatic, oesophageal and thyroid cancers, with major amplifications usually involving the AKT2 isoform [116]. In addition, AKT post-translational modification, such as lysine modifications, tyrosine phosphorylation, O-GlcNAcylation, acetylation, and sumoylation are important in retaining AKT hyperactivation in cancers, even in conditions where normal PI3K and PTEN activity persists [1, 117, 118]. AKT is elevated in a subset of premalignant breast lesions. Indeed, p-AKT is overexpressed in 33% ductal carcinoma in situ lesions and in 38% of invasive breast cancers, where most tumors (79%) express the oestrogen receptor [119]. Additionally, phosphorylation of AKT at Ser473 can promote breast cancer metastasis [120], and increased AKT1 activity has been observed in 40% of breast cancers [121]. Moreover, AKT overactivation has been observed in several other types of cancers [77, 122, 123, 80, 78, 82, 75] (Fig. 1b) (Supplementary information 1).

#### AKT protein targets in cancer

**Fig. 2:**
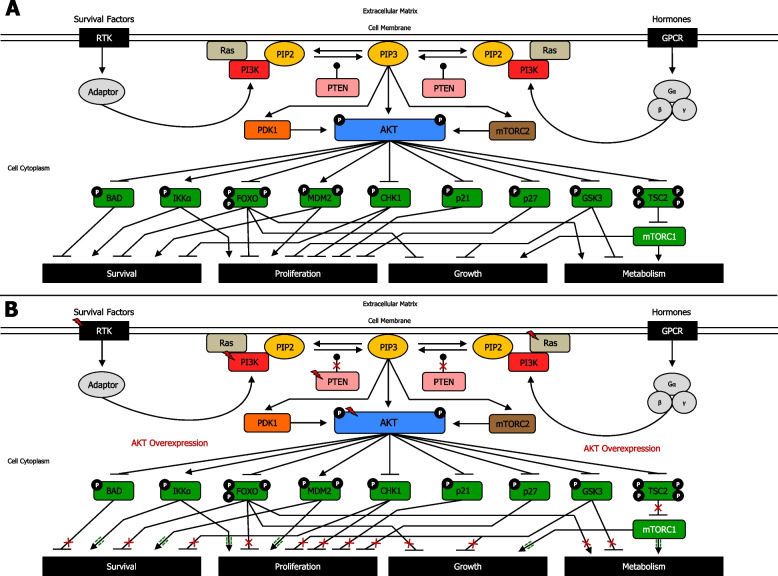
AKT signaling network targets and regulates critical cellular substrates. **A** AKT regulation of targeted proteins in normal cells. AKT phosphorylation of downstream substrates determines regulation of distinct cellular functions. There are several AKT cytoplasmic targets, including BAD, IKKα, FOXO, MDM2, CHK1, p21, p27, GSK-3, and TSC2, representing crucial signaling nodes that interlink AKT signaling with supplementary cellular regulatory circuits. In normal conditions, PAM pathway moderately promotes essential cellular functions such as survival, proliferation, growth and metabolism. **B** AKT regulation of targeted proteins in cancer cells. Mutations in RTK, Ras, PI3K, PTEN protein phosphatase, AKT, and/or other proto-oncogenes, may occur, resulting in AKT overexpression, leading to enhanced inhibition of BAD, FOXO, CHK1, p21, p27, GSK3, and TSC2, as well as increased activity of IKKα, MDM2, with consequently higher survival, increased proliferation, enhanced growth and boosted metabolism. Activation (phosphorylation or non-phosphorylation) is shown with arrowhead lines, inhibition (phosphorylation or non-phosphorylation) is indicated with blocked lines, and dephosphorylation, carried out by phosphatases, is displayed with roundhead lines. Red lightning symbol shows mutation for a particular gene in the PAM pathway. Red crosses emphasise signaling blockage, whereas green dash-dotted lines (adjacent to arrowhead lines) highlight signaling enhancement. P: phosphoryl group

AKT phosphorylation of downstream substrates determines the regulation of distinct cellular functions [124] (Fig. 2a) (Supplementary information 1). In cancer cells, AKT overactivation due to mutation of AKT or mutations upstream the PAM pathway, can trigger phosphorylation on substrates, determining either blockage or enhancement of their activities [125]. Indeed, in cancer, AKT can exert numerous important functions: 1) increases phosphorylation on BAD, thereby inhibiting apoptosis, and thus, increasing cell survival [126]; 2) enhances phosphorylation on IKKα, contributing to cell survival and proliferation [127]; 3) increases phosphorylation, and thus, inhibits the transcriptional functions of FOXO, contributing to cell survival, proliferation, growth [128] and reprogramming cell metabolism [129]; 4) increases MDM2 phosphorylation, thereby regulating and inhibiting p53 response, promoting cell survival and proliferation, leading to tumorigenesis [130]; 5) enhances phosphorylation on Chk1, endorsing cell survival and proliferation [131]; 6) increases phosphorylation, and thus, inhibition of p21 and p27, thereby increasing cell proliferation [132]; 7) enhances phosphorylation on GSK3 leading to increase in cell proliferation and growth, as well as boosting cellular anabolism [133]; and 8) increases phosphorylation on TSC2, and consequently, reduces inhibition of mTORC1, resulting in enhanced cell growth and cell metabolism [134] (Fig. 2b) (Supplementary information 1).

#### AKT-mediated GSK3 inhibition in cancer

**Fig. 3:**
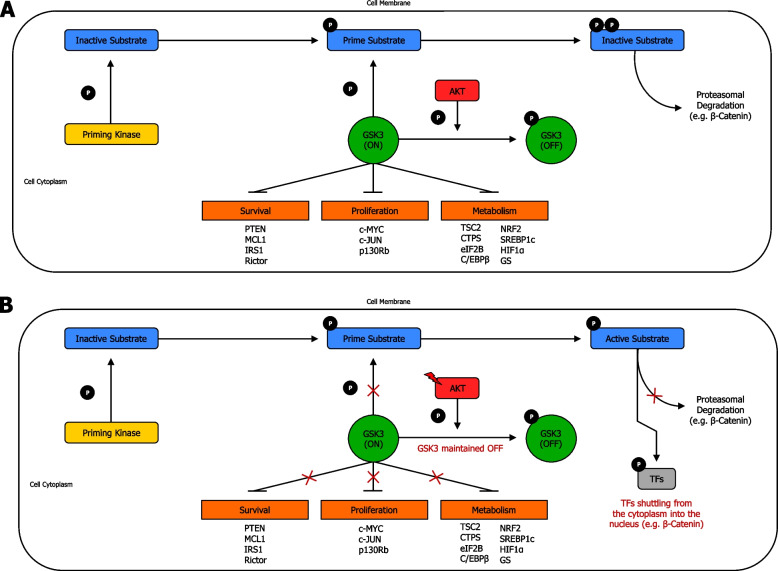
AKT-mediated GSK3 phosphorylation and regulation. **A** AKT-mediated GSK3 regulation in normal cells. AKT-mediated GSK3 regulation is exerted by AKT phosphorylation on GSK3 amino-terminus, thereby creating an intramolecular pseudo-substrate that occludes the phosphate-binding pocket, and inhibits substrate accessibility to GSK3. When in active (on) form, GSK3 can only recognise and phosphorylate substrates previously phosphorylated by a priming kinase. Conversely, when in inactive (off) form, GSK3 results blocked due to AKT phosphorylation, and thus, its access to primed substrates is denied. Some GSK3 substrates, with their corresponding cellular function are shown. **B** AKT-mediated GSK3 regulation in cancer cells. Mutations in AKT can enhance phosphorylation, and thus, inactivation of GSK3. Consequently, inactivation or limited amount of active GSK3 can lead to dysregulation of several signal transduction, resulting in cancer onset and/or progression. Reduction or absence of phosphorylation, and thence decreased proteasomal degradation of molecules (e.g. β-catenin) can arise from excessive inhibition of GSK3 by AKT phosphorylation, which can lead to increased survival, enhanced proliferation, and boosted metabolism. Red lightning symbol shows mutation for a particular gene in the PAM pathway. TFs: transcription factors. Phosphorylation is shown with arrowhead lines, whereas inhibition is indicated with blocked lines. Red crosses emphasise signaling blockage. P: phosphoryl group

AKT-mediated GSK3 inhibition is determined by AKT phosphorylation on GSK3 NH2-terminus, forming an intramolecular pseudo-substrate that obstructs the phosphate-binding pocket, and consequently, suppresses substrate availability to GSK3 [135] (Fig. 3a) (Supplementary information 1). GSK3 closely interacts with the PAM pathway and can function both as a tumor promoter and tumor suppressor. In fact, abnormal expression of GSK3 can interfere with the advancement and evolution of tumor through dysregulation of cell cycle, apoptosis, and senescence, as well as resistance to chemotherapy and radiotherapy [133]. GSK3 is a signal integrator that often acts at the intersection of several biochemical pathways. Indeed, a GSK3-associated signaling transduction pathway that is often involved in human tumor is the EGFR/Ras/PI3K/PTEN/AKT/GSK3/mTORC1 axis, which exerts an important role in natural cell growth, and is frequently overactivated with mutations mainly occurring in PI3K (PIK3CA), Ras, and PTEN [136]. Notably, AKT can directly phosphorylate, inactivate and target GSK3 for degradation [137]. Consequently, inactivation, or low levels of active GSK-3, can lead to dysregulation of multiple signaling pathways. In fact, when mTOR and TSC2 are not inactivated by GSK3, the mTORC1 complex results active, leading to translation of several growth-regulating mRNAs [138]. Importantly, the WNT/β-catenin is the major pathway regulated by GSK3. The WNT/β-catenin axis is also critical in cellular proliferation and in EMT, which is essential for epithelial tumor metastasis. GSK3, when present in its active form, phosphorylates β-catenin, resulting in the proteasome degradation of β-catenin; and thus, several important genes required for cell proliferation are not transcribed. Conversely, AKT phosphorylation suppresses GSK3, thereby allowing β-catenin to shuttle from the cytoplasm into the nucleus to function as transcription factor, and consequently, leading to cell proliferation [139] (Fig. 3b) (Supplementary information 1).

#### AKT-induced FOXO regulation in cancer

**Fig. 4:**
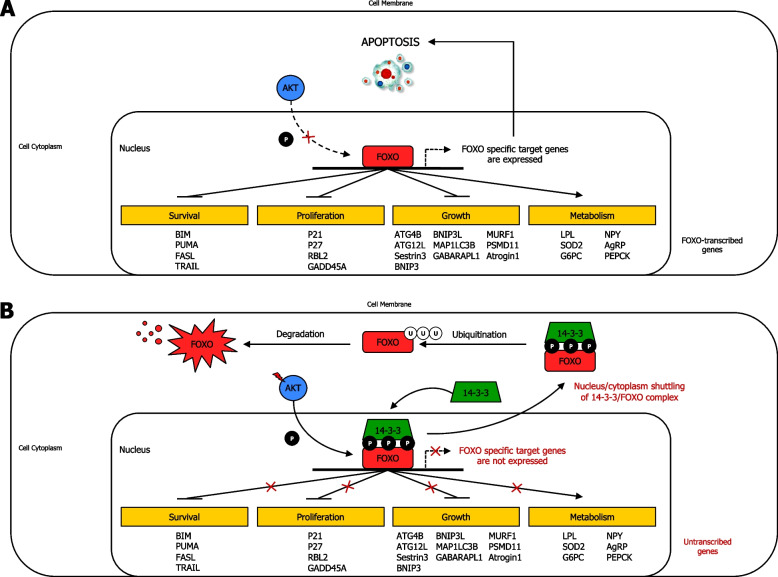
AKT-induced FOXO phosphorylation and regulation. **A** AKT-induced FOXO phosphorylation and regulation in normal cells. In normal condition, AKT exerts an ordinary moderate FOXO phosphorylation, which allows FOXO to transcribe its target genes. **B** AKT-induced FOXO phosphorylation and regulation in cancer cells. Mutations upstream AKT and/or AKT, with its consequent overexpression, can increase FOXO phosphorylation by AKT, resulting in binding of 14–3-3 adapter protein to FOXO, and leading to 14–3-3/FOXO complex being shuttled from nucleus to cytoplasm, thereby inhibiting the expression of FOXO gene targets. Therefore, excessive inhibition of FOXO by AKT phosphorylation can ultimately increase survival, enhance proliferation, increase growth, and suppress cell metabolism. Activation (phosphorylation or non-phosphorylation), interactions, and nucleus/cytoplasm shuttling are shown with arrowhead lines, moderate or possible phosphorylation is indicated with dotted-arrowhead lines, and inhibition is displayed with blocked lines. Red lightning symbol shows mutation for a particular gene in the PAM pathway. Red crosses emphasise signaling blockage. P: phosphoryl group. Survival: Cell survival; Proliferation: Cell proliferation; Growth: Cell growth; Metabolism: tissue-specific metabolic changes

Several gene targets mainly involved in response to different insulin and insulin-like growth factor 1 (IGF1) signaling are regulated by FOXO transcription factor family [140]. FOXO-targeted genes are associated with activation of apoptosis, cell cycle blockage, growth inhibition, and tissue-specific metabolic changes [141] (Fig. 4a) (Supplementary information 1). In cancer, overactivation of AKT induces continuous phosphorylation of FOXO and binding of FOXO to 14-3-3 protein, which consequently results in durable FOXO nuclear export. FOXO3 then undergoes ubiquitination in the cytoplasm, and thus is degraded by the proteasome. This AKT-mediated activity causes stable blockage of FOXO expression, thereby promoting cell survival (e.g. due to BIM inactivation), cell proliferation (e.g. due to p21 inactivation), cell growth (e.g. due to ATG4B inactivation) [142], and reduction of tissue-specific metabolic changes (e.g. due to LPL inactivation) [143]. Thus, due to its pro-apoptotic activity, FOXO can also function as tumor suppressor in several types of cancer [144] (Fig. 4b).

#### AKT-mediated regulation of mTORC1 and TSC2 in cancer

**Fig. 5:**
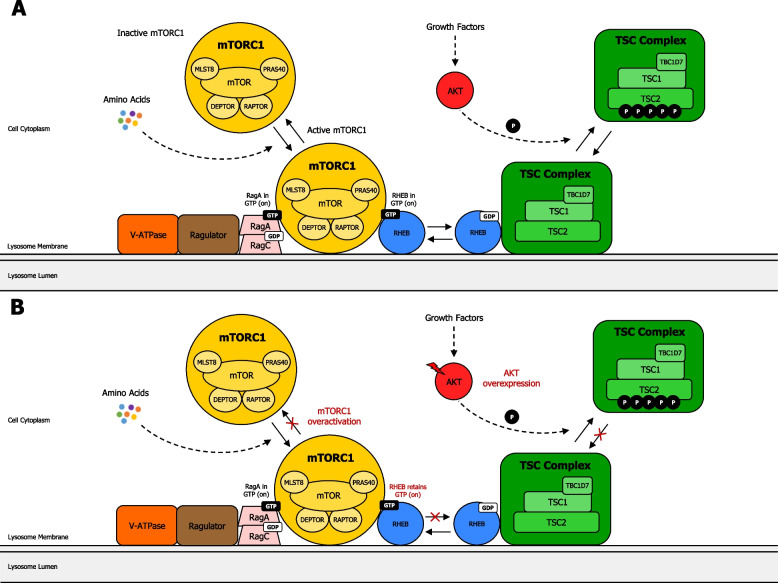
Regulation of mTORC1 through the TSC complex. **A** Regulation of mTORC1 through the TSC complex in normal cells. The signal integration model of mTORC1 is regulated by growth factors and amino acids. Rag heterodimer (RagA and RagC) interacts with Ragulator and V-ATPase on the lysosome membrane. Amino acids then allow connection of mTORC1 to Rag heterodimer/Ragulator/V-ATPase complex. The TSC complex maintains Rheb in the GDP-bound state. Growth factor-induced AKT phosphorylates TSC2, leading to dissociation from the lysosomal membrane, promoting Rheb to become GTP loaded, and thus, activating mTORC1. **B** Regulation of mTORC1 through the TSC complex in cancer cells. Mutations in AKT or upstream genes in the PAM pathway, can potentially lead to overactivation of mTORC1, due to TSC complex being released from Rheb. Consequently, Rheb becomes GTP loaded, resulting in activation of mTORC1, recruited by Rag proteins. This dysregulation may promote the onset and/or progress of cancer, resulting in enhanced cell survival, proliferation, growth, and metabolism in cancer cells. Activation of mTORC1 potentially sends critical signals that engender tumor cells to metastasize and invade new tissues. mTORC1: mechanistic target of rapamycin complex 1; DEPTOR: DEP domain-containing mTOR-interacting protein; MLST8: mammalian lethal with SEC13 protein 8; PRAS40: proline-rich AKT1 substrate 1; RAPTOR: regulatory-associated protein of mTOR; RHEB: Ras homolog enriched in brain; TBC1D7: TBC1 Domain Family Member 7; TSC2: tuberous sclerosis complex 2. Activation (phosphorylation or non-phosphorylation) is shown with arrowhead lines or dotted-arrowhead lines. Red lightning symbol shows mutation for a particular gene in the PAM pathway. Red crosses emphasise signaling blockage. P: phosphoryl group

Cell growth is mainly regulated through AKT-mediated activation of the protein kinase mTORC1 [145]. Activation of mTORC1 occurs through nutrient- and AKT-induced inhibitory phosphorylation of TSC2 [146], which acts in a molecular complex (known as the TSC complex) that also incorporates TSC1 and TBC1D7 [145]. mTORC1 exerts a dual role: a promoting downstream effector of PAM signaling pathway, and an inhibiting regulator with remarkable negative feedback effects on the induction of AKT by cell surface receptors (RTKs or GPCRs) [147] (Fig. 5a) (Supplementary information 1). In cancer cells, mutations [23] and/or gene amplifications in AKT [116], or mutations upstream genes in the PAM pathway, especially PI3K genes [15] and PTEN [148], can potentially result in AKT-induced overactivation of mTORC1, leading to increased cell survival, growth, proliferation, and metabolism in cancer cells. Moreover, altered mTORC1 activation sends critical signals that enhance tumor cells to metastasize, and invade new healthy tissues [149]. Thus, mutations in PAM pathway allow TSC complex to be released from Rheb, and consequently, Rheb turns into GTP loaded, leading to activation of mTORC1, recruited by Rag proteins [150] (Fig. 5b).

### PAM Signaling network in cancer

#### Feedback mechanisms in cancer

**Fig. 6:**
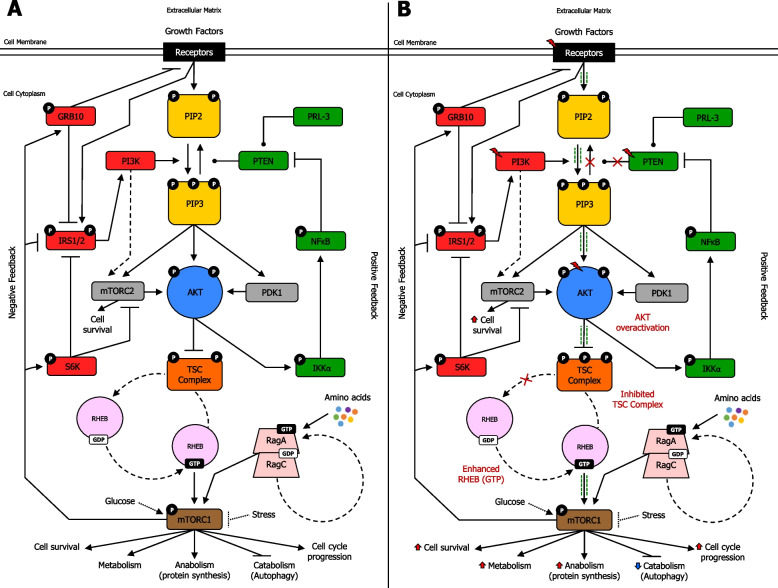
The PAM signaling pathway and its downstream functions. **A** PAM pathway downstream functions in normal cells. PI3K activation occurs by growth factor-induced receptors or through interaction with scaffolding adaptors, including IRS1/2 proteins. PI3K is then recruited to its substrate PIP2, promoting generation of PIP3. Inactive AKT in the cytoplasmic matrix binds to PIP3 on the cell membrane, allowing phosphorylation by PDK1 and mTORC2, leading to complete activation of AKT, which subsequently phosphorylates several downstream targets, including multiple sites on TSC2, which forms a functional complex with TSC1 (TSC Complex). AKT-induced phosphorylation on TSC2 hampers the ability of TSC Complex to act as a GAP toward the small GTPase Rheb, endorsing Rheb-GTP accumulation. As a result, Rheb-GTP remarkably activates mTORC1, which phosphorylates and activates S6K. In the negative PAM feedback loop, mTORC1 and S6K1 directly phosphorylate IRS1/2, impeding PI3K activation. In addition, mTORC1 blocks GRB10-mediated growth factor-induced receptor signaling to PI3K. Conversely, in the positive PAM feedback loop, AKT phosphorylates IKKα, which indirectly activates transcription factor NF-κB, allowing PTEN phosphatase inhibition. Besides, PRL-3 phosphatase can also inhibit PTEN phosphatase. PAM downstream functions include cell survival, metabolism, anabolism, catabolism, and cell cycle progression. **B** PAM pathway downstream functions in cancer cells. Mutations in RTK, PI3K, AKT, PTEN, and possibly other genes, may occur. Overactivation of AKT strongly enhances phosphorylation on TSC2, which further hampers the ability of the TSC Complex to act as a GAP toward the small GTPase Rheb, thereby remarkably endorsing Rheb-GTP accumulation. Thus, dysregulation of PAM pathway signal transduction, due to mutations and/or inevitable alterations in the negative feedback loop or positive feedback loop, can possibly lead to cancer onset and/or progression. This results in enhanced PAM downstream functions, such as increased cell survival, boosted metabolism, enhanced anabolism, reduced catabolism, and increased cell cycle progression. Activation (phosphorylation or non-phosphorylation) is shown with arrowhead lines or dotted-arrowhead lines, inhibition (phosphorylation or non-phosphorylation) is indicated with blocked lines, and dephosphorylation is displayed with roundhead lines. Red lightning symbol shows mutation for a particular gene in the PAM pathway. Red crosses emphasise signaling blockage, whereas green dash-dotted lines (adjacent to arrowhead lines or blocked lines) highlight signaling enhancement. Red upper-arrows show increases, whereas blue lower-arrows indicate reduction. Red upper-arrows show increases, whereas blue lower-arrows indicate reduction. P: phosphoryl group

The PAM pathway is regulated by negative and positive feedback to ensure that stimulation signal transductions are captured and delivered transiently [151] (Fig. 6a) (Supplementary information 1). In cancer, mutations in RTK [152], PI3K [153], AKT [23], mTORC2 [154], and/or mTORC1 genes [155] can alter the PAM negative feedback signal transduction. Likewise, mutations in NF-κB [156], PRL-3 [92] and/or PTEN [157] can dysregulate the PAM positive feedback loop. Thus, PAM pathway mutations in either negative or positive feedback loops can potentially lead to the onset and/or progression of cancer [158, 145] (Fig. 6b).

#### Major PAM pathway cross-regulation with Ras/ERK and Wnt/GSK3/β-catenin pathways

**Fig. 7:**
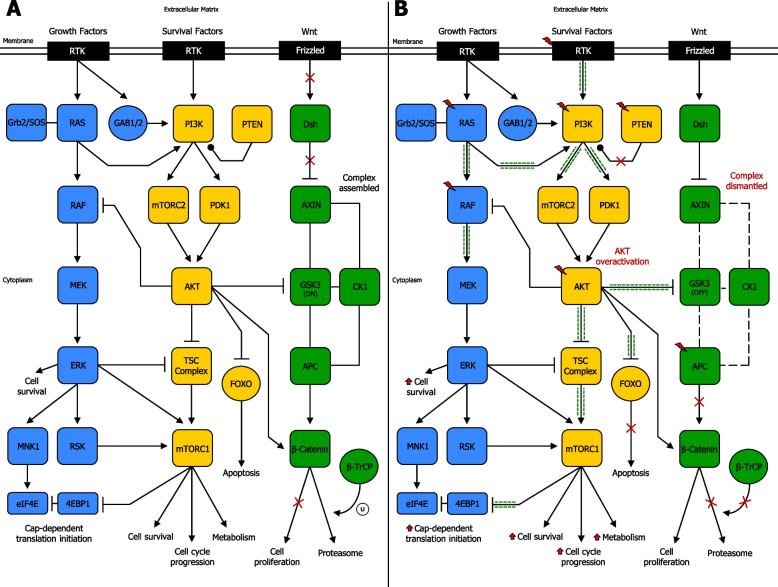
Network of signaling cross-regulation between PAM pathway and RAS/ERK pathway or Wnt/GSK3/β-catenin pathway. **A** Simplified cross-regulation between PAM pathway and RAS/ERK pathway or Wnt/GSK3/β-catenin pathway in normal cells. Numerous cross-talk points occur between PAM pathway and RAS/ERK pathway or Wnt/GSK3/β-catenin pathway, leading to ordinary cell survival and proliferation, cell cycle progression, cell metabolism, apoptosis, and other cellular functions. **B** Simplified cross-regulation between PAM pathway and RAS/ERK pathway or Wnt/GSK3/β-catenin pathway in cancer cells. Mutations in RTK, RAS, PI3K, PTEN, AKT, APC, and possibly other genes, may occur, resulting in cross-talk dysregulations between PAM pathway and RAS/ERK pathway or Wnt/GSK3/β-catenin pathway. This can lead to enhanced PAM downstream signaling, such as increased cell survival and proliferation, enhanced cell cycle progression, boosted cell metabolism, reduced apoptosis, and dysregulation of other important cellular functions. Activation (phosphorylation or non-phosphorylation) is shown with arrowhead lines, inhibition (phosphorylation or non-phosphorylation) is indicated with blocked lines, interaction is displayed with continuous lines, disassociation is shown with dotted lines, and dephosphorylation, carried out by phosphatases, is indicated with roundhead lines. Red lightning symbol shows mutation for a particular gene in the PAM pathway. Red crosses emphasise signaling blockage, whereas green dash-dotted lines (adjacent to arrowhead lines or blocked lines) highlight signaling enhancement. Red upper-arrows show increases. U: ubiquitination

The PAM pathway frequently cross-regulates with the Ras/ERK pathway [159] (Fig. 7a) (Supplementary information 1). In human tumors, mutations in genes encoding effector molecules of PAM pathway and Ras/ERK pathway commonly coexist. Indeed, PI3K genes and Ras genes mutations, PI3K genes and BRAF mutations, or PTEN and BRAF mutations often occur together in numerous cancer types. Interestingly, concurrent mutations in these two pathways can abrogate the dependence on a single pathway in tumor cells due to the induction of molecules whose function integrates the effects of both these signalling axes. In fact, 4E-BP1, a repressor of mRNA translation, is a crucial integrator of PAM pathway and Ras/ERK pathway, acting as a key mediator of their effects on malignant transformation [160]. Importantly, mTOR persistently restrains the tumor-suppressive function of 4E-BP1 via phosphorylation thereby releasing 4E‐BPs from eIF4E and enabling cap-dependent translation initiation in cancer [161, 162]. In breast tumor cells, which overexpress ErbB-2, PI3K inhibition causes an activation of Ras/ERK pathway, as a consequence of ErbB receptor induction. Treatment with ErbB-2 inhibitors or MEK inhibitors enhances the activity of PI3K inhibitors, leading to reduced proliferation and improved anticancer efficacy, in comparison to a single agent [163]. Further studies have described the cross-regulation between PAM pathway and Ras/ERK pathway in cancer (Fig. 7b) (Supplementary information 1). The PAM pathway also often cross-regulates with the Wnt/GSK3/β-catenin pathway [164] (Fig. 7a) (Supplementary information 1). In cancer, AKT is usually overexpressed, and consequently, GSK3 is often further inactivated [133]. Besides, AKT can directly phosphorylate (Ser552) and activate β-catenin, triggering its nuclear translocation, enhancing its transcriptional activity [139], resulting in uncontrolled, cell proliferation and playing a critical role in cancer invasion and development [165]. Likewise, AKT expression can also be regulated by Wnt/β-catenin signaling. In fact, β-catenin is able to increase AKT activation in colorectal tumors [166]. Moreover, GSK3 can phosphorylate TSC complex [167]. Interestingly, TSC complex, in turn has been reported to regulate β-catenin signaling activity through GSK3, and this crosstalk promotes degradation of β-catenin. In fact, mutations of the TSC complex in tuberous sclerosis-patients are often associated with incidence of cancers such as subependymal giant cell astrocytoma (SEGA). Higher levels of β-catenin have been detected in tumor tissues of SEGA patients, and this augmentation was associated with translocation of β-catenin from cytoplasm to nucleus, with upregulation of target proto-oncogene c-Myc [168]. Thus, when TSC2 complex is not disabled by GSK3, the mTORC1 complex is effective and can lead to translation and proliferation of several growth-regulating mRNAs [138]. More studies have focussed on the cross-regulation between PAM pathway and Wnt/GSK3/β-catenin pathway in cancer [169] (Fig. 7b) (Supplementary information 1).

#### Major cross-regulation between PAM pathway and other pathways

**Fig. 8:**
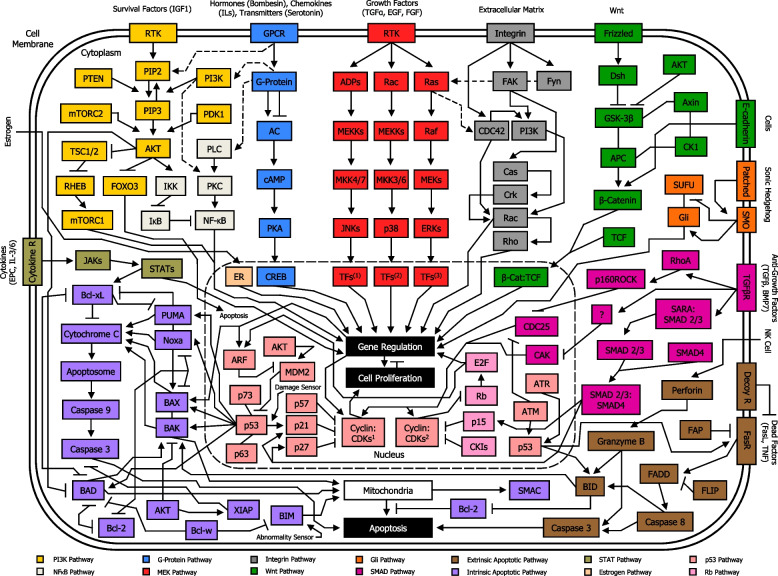
Simplified overview of PAM signaling pathway location among other major signal transduction pathways in the network circuit of a normal cell. Each component of a signaling pathway is termed according to the role it plays with respect to the initial stimulus, such as ligands (e.g. IGF1), receptors (e.g. RTK), and effectors (e.g. PI3K). In normal cells, PAM pathway moderately promotes essential cellular functions such as survival, proliferation, growth and metabolism. Signaling cross-talks between PAM pathway and other pathways play a major role in the ultimate function of the cell. PI3K pathway, NF-κB pathway, G-Protein pathway, MEK pathway, Integrin pathway, Wnt pathway, Gli pathway, SMAD pathway, extrinsic apoptotic pathway, intrinsic apoptotic pathway, STAT pathway, Estrogen pathway, p53 pathway, and Rb pathway are shown. Nuclear membrane is delimited by a long-dashed circular dotted line. Arrowhead lines: activation (phosphorylation or non-phosphorylation). Blocked lines: inhibition (phosphorylation or non-phosphorylation). Continuous lines: interaction. Dotted arrowhead lines: cross-talk activation (phosphorylation or non-phosphorylation) between pathways. Dotted blocked lines: cross-talk inhibition (phosphorylation or non-phosphorylation) between pathways. PI3K: PI3K/Ras complex. MEKs: MEK1/2. ERKs: ERK1/2. TFs1: Transcription factors Jun, ATF2, RNPK, p53, NFAT4, Shc. TFs2: Transcription factors CHOP, ATF2, MNK, MSK, MEF2, Elk-1. TFs3: Transcription factors Elk-1, Ets-2, RSK, MNK, MSK, cPLA2, Fos, Myc. FAK: FAK/Src complex. Fyn: Fyn/Shc complex. Dsh: Dishevelled. Cyclin:CDKs1: Cyclin A:CDK1, Cyclin A:CDK2, Cyclin B:CDK1, Cyclin E:CDK2. Cyclin:CDKs2: Cyclin D:CDK4, Cyclin D:CDK6. CKIs: Cyclin-dependent kinase inhibitors (p16, p18, p19)

**Fig. 9:**
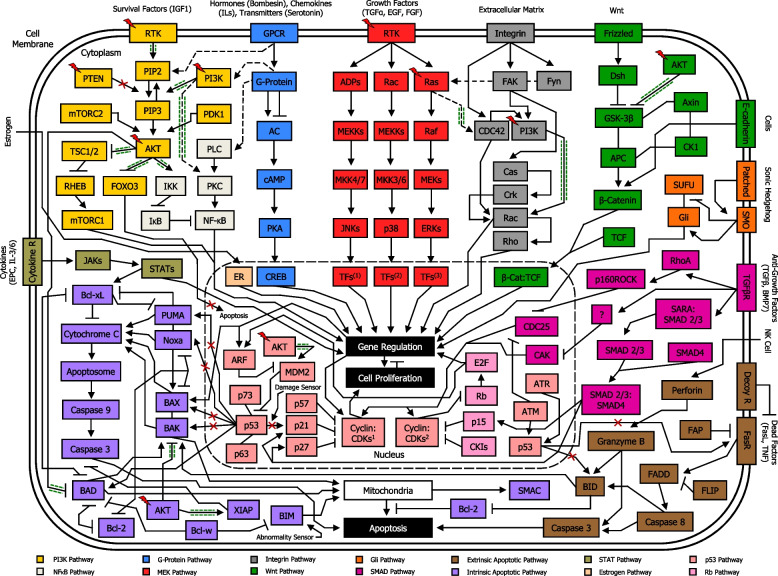
Simplified overview of PAM signaling pathway location among other major signal transduction pathways in the network circuit of a cancer cell. Each component of a signaling pathway is termed according to the role it plays with respect to the initial stimulus, such as ligands (e.g. IGF1), receptors (e.g. RTK), and effectors (e.g. PI3K). In cancer cells, mutations in RTK, Ras, PI3K, PTEN protein phosphatase, AKT, and/or other proto-oncogenes, may occur, resulting in PAM signaling pathway overexpression. This condition can lead to stronger AKT-induced inhibition of pro-apoptotic proteins such as FOXO, BAD, BAX, BAK, NOXA, PUMA, as well as enhanced activity of anti-apoptotic proteins such as XIAP, and amplified activity of mTORC1, IKKα, PKC, CDC42, Rac, GSKβ, and MDM2, with consequently uncontrolled survival, proliferation, growth and boosted metabolism. Signaling cross-talk between PAM pathway and other pathways play a major role in the dysregulation of functions in a cancer cell. PI3K pathway, NF-κB pathway, G-Protein pathway, MEK pathway, Integrin pathway, Wnt pathway, Gli pathway, SMAD pathway, extrinsic apoptotic pathway, intrinsic apoptotic pathway, STAT pathway, Estrogen pathway, p53 pathway, and Rb pathway are shown. Nuclear membrane is delimited by a long-dashed circular dotted line. Arrowhead lines: activation (phosphorylation or non-phosphorylation). Blocked lines: inhibition (phosphorylation or non-phosphorylation). Continuous lines: interaction. Dotted arrowhead lines: cross-talk activation (phosphorylation or non-phosphorylation) between pathways. Dotted blocked lines: cross-talk inhibition (phosphorylation or non-phosphorylation) between pathways. Red lightning symbol shows mutation for a particular gene in the PAM pathway. Red crosses: signaling blockage. Green dash-dotted lines (adjacent to arrowhead lines or blocked lines or dotted arrowhead lines): signaling enhancement. PI3K: PI3K/Ras complex. MEKs: MEK1/2. ERKs: ERK1/2. TFs1: Transcription factors Jun, ATF2, RNPK, p53, NFAT4, Shc. TFs2: Transcription factors CHOP, ATF2, MNK, MSK, MEF2, Elk-1. TFs3: Transcription factors Elk-1, Ets-2, RSK, MNK, MSK, cPLA2, Fos, Myc. FAK: FAK/Src complex. Fyn: Fyn/Shc complex. Dsh: Dishevelled. Cyclin:CDKs1: Cyclin A:CDK1, Cyclin A:CDK2, Cyclin B:CDK1, Cyclin E:CDK2. Cyclin:CDKs2: Cyclin D:CDK4, Cyclin D:CDK6. CKIs: Cyclin-dependent kinase inhibitors (p16, p18, p19)

Apart from MEK pathway and Wnt pathway, there are other signaling pathways interacting directly with PAM pathway, including NF-κB pathway [170], G-protein pathway [171], integrin pathway [172], intrinsic apoptotic pathway [173], and p53 pathway [174, 175] (Fig. 8). In cancer cells, mutations in RTK and PI3K genes, as well as Ras genes, PTEN, AKT, and/or other proto-oncogenes can occur, leading to PAM signaling pathway overexpression. AKT overactivation can trigger phosphorylation of substrates, determining either blockage or enhancement of their activities. In addition, signaling cross-talks between PAM pathway and other pathways exert a significant role in the dysregulation of cellular functions in tumor. Importantly, dysregulation of PAM signaling pathway can often result in stronger AKT-induced inhibition of pro-apoptotic proteins, including FOXO [128], BAD [126], BAX [176], BAK [173], and/or enhanced activity of anti-apoptotic proteins such as XIAP [177]. Besides, this condition can lead to AKT-induced higher activity of mTORC1 [134], IKKα [127], and MDM2 [130], as well as AKT-induced phosphorylation and consequent inhibition of GSKβ [133]. Moreover, PI3K-mediated increased activity of PKC [178], and Rac [179], as well as Ras-induced overexpression of CDC42 [180], may occur. Altogether, these altered transductions signaling can thereafter result in uncontrolled survival, proliferation, growth and boosted metabolism in cancer cells (Fig. 9).

### Epigenetic regulation of PAM pathway in cancer

Accumulating evidence suggests that epigenetic alterations are as crucial as genetic mutations in dysregulating PAM signaling pathway in different types of cancer [181–184]. In particular, DNA methylation [185], histone post-translational modifications [186], and non-coding RNAs modulation [187, 188], are three main epigenetic mechanisms that affect PAM pathway and have been associated with cancer. Accordingly, loss of PTEN tumor suppressor gene expression due to aberrant promoter hypermethylation has been implicated in the development of gastric [189], colorectal [190], and endometrial cancers [191]. Similarly, promoter hypermethylation coupled with transcriptional silencing has been documented in loci coding for negative regulators of AKT, including tumor suppressor genes SCGB3A1 in NSCLC [192] and PPP2R2B in breast cancer [193], therefore contributing to cancer onset/progression. In turn, AKT hyperactivation in cancer cells brings further dysregulation in various epigenetic players participating in the PAM pathway, such as DNA maintenance methyltransferase DNMT1 [194], histone acetyltransferase CBP/p300 complex [195], histone H3K27 methyltransferase EZH2 [196], and histone H3K4 methyltransferase and demethylase KMT2D and KDM5A, respectively [31]. It could be argued that the imbalance in histone acetylation and methylation at chromatin loci regulated by the PAM pathway frequently occurs during cancer progression. Indeed, exposure of thyroid cancer cell lines to AKT inhibitor B2311 drastically reduces H3K9ac and H3K4me3 levels, both transcription activation marks; and increases levels of H3K27me3, a well-known transcription repression mark, at the promoter of tumor suppressor tshr gene, leading to downregulation of tshr [197]. The epigenetic signature of different cancers is also linked to the impact of multiple long and short noncoding RNAs in the PAM pathway. In fact, distinct miRNAs trigger PAM pathway by targeting PTEN mRNA, including miR-21 in gastric cancer [198], miR-20b and miR-301a-3p in esophageal carcinoma [199], and miR-421 in NSCLC [200]. Stepwise investigation reveales cooperative roles of distinct components of the epigenetic machinery to induce cancer cell proliferation through PAM pathway. For instance, altered chromatin topology and DNA methylation pattern at the imprinted IGF2-AS locus provoke significant decreased transcription of the long noncoding IGF-AS RNA in prostate [201] and CRC [202], as well as in cervical intraepithelial neoplasia [203]. Moreover, patients with low IGF2-AS abundance likely develop larger tumor size. In contrast, overtranscription of IGF2-AS inhibits breast cancer cell proliferation, thereby retarding tumor malignancy and progression in vivo. Mechanistically, IGF2-AS inhibits the expression of its sense-cognate igf2 gene in an epigenetic DNMT1-dependent manner, leading to the inactivation of downstream PAM pathway [204]. Thus, identification and understanding of epigenetic changes, such as DNA methylation, histone post-translational modifications, and non-coding RNA-mediated transcriptional silencing, occurring along the PAM pathway may hold a great promise for improving personalized medicine to a larger number of cancer patients.

## Clinical developments in PAM inhibitors

### PI3K Inhibitors

**Fig. 10:**
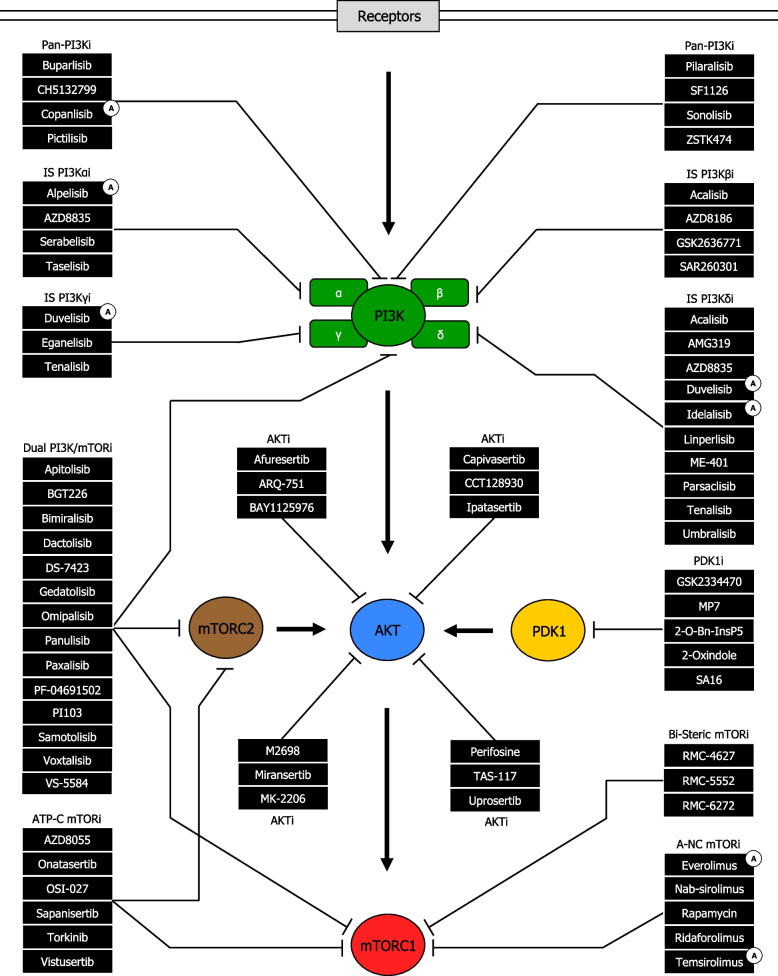
PI3K-, AKT-, mTOR- and PDK1-targeted inhibitors along the PAM pathway in cancer cells. PI3K inhibitors are divided into Pan-PI3K inhibitors (Pan-PI3Ki), Dual PI3K/mTOR inhibitors (Dual PI3K/mTORi), and Isoform-Specific PI3K inhibitors (IS PI3Ki), named Isoform-Specific PI3Kα inhibitors (IS PI3Kαi), Isoform-Specific PI3Kβ inhibitors (IS PI3Kβi), Isoform-Specific PI3Kγ inhibitors (IS PI3Kγi), and Isoform-Specific PI3Kδ inhibitors (IS PI3Kδi). Overall AKT inhibitors are referred as AKTi. mTOR inhibitors are divided into allosteric (non-competitive) mTOR inhibitors (A-NC mTORi), ATP-competitive mTOR inhibitors (ATP-C mTORi), and Bi-Steric mTOR inhibitors (Bi-Steric mTORi). PDK1 inhibitors are referred as PDK1i. Substrate activation (phosphorylation) along the PAM pathway is shown with arrowhead lines. Substrate inhibition along the PAM pathway, exerted by Pan-PI3Ki, Dual PI3K/mTORi, IS PI3Kαi, IS PI3Kβi, IS PI3Kγi, IS PI3Kδi, AKTi, A-NC mTORi, ATP-C mTORi, Bi-Steric mTORi, and PDK1i, is shown with blocked lines. Ⓐ: FDA-approved inhibitor

Along the PAM pathway PI3K is a major drug target for cancer treatment since its hyperactivity is remarkably correlated with human tumor progression, enhanced tumor microvessel formation, and increased number of invasive cancer cells [205]. A strenuous effort has been committed to improve inhibitors targeting PI3K signaling. Indeed, several pharmaceutical companies have developed drug inhibitors of PI3K during the last decades [206]. Notwithstanding some inhibitors have been approved by the Food and Drug Administration (FDA), there is still concern regarding the development of resistance, sensitivity markers, and toxicology [207]. Importantly, PI3K inhibitors are classified into three main groups: pan-PI3K inhibitors (pan-PI3Ki), isoform-specific PI3K inhibitors (IS PI3Ki), and dual PI3K/mTOR inhibitors (dual PI3K/mTORi) [208] (Fig. 10).

#### Pan-PI3K Inhibitors

Pan-PI3Ki suppress the cataytic activity of all four PI3K class I isoforms: PI3Kα, PI3Kβ, PI3Kγ, and PI3Kδ, encoded by PIK3CA, PIK3CB, PIK3CG, and PIK3CD, respectively. Thus, these drugs are normally effective in tumors producing high level of PIP3, regardless of the type of PI3K gene or PTEN alterations implicated. Potentially, pan-PI3Ki provide a broader range of activity by comprising numerous molecular targets, although exists increasing risk of on-target and off-target toxicity [209]. Several pan-PI3Ki have been in clinical development, but only copanlisib, a potent and selective agent with targeted activity predominantly against PI3K p110α and p110δ isoforms, has reached significant effectiveness in clinical trials [210]. Indeed, copanlisib has exhibited remarkable clinical benefits in patients with relapsed follicular lymphoma (FL) who have received ≥ 2 prior systemic therapies, and thus has been FDA-approved for use in this cohort (Supplementary information 2) (Supplementary figure 1). The safety profile of copanlisib in FL patients is favourable or acceptable. Treatment-related adverse events of any grade include hyperglycaemia, leukopenia, decreased energy, diarrhea, hypertension, neutropenia, nausea, thrombocytopenia, and infections. Serious adverse reactions comprise pneumonia (8%), pneumonitis (5%), and hyperglycaemia (5%) [211]. Clinical anticancer activity of copanlisib has been shown as monotherapy in hematological malignancies [212–215] and in advanced solid tumors [216, 217], as well as in combination with either ibrutinib [218], gemcitabine [219], bendamustine [220], rituximab [221], or rituximab + cyclophosphamide + doxorubicin + vincristine + prednisone [220], in hematological malignancies, and in combination with either refametinib [222], gemcitabine [223], or gemcitabine + cisplatin [224], in advanced solid tumors. Besides, antineoplastic activity has been observed with other pan-PI3Ki including buparlisib as monotherapy/in combination in hematological malignancies and advanced solid tumors, and pictilisib, pilaralisib and sonolisib as monotherapy/in combination with other drugs in advanced solid tumors (Supplementary information 2) (Supplementary table 1).

#### Isoform-Specific PI3K Inhibitors

IS PI3Ki have been established to target cancer types dependent on either PI3Kα, PI3Kβ, PI3Kγ, or PI3Kδ isoforms. Conventionally, these drugs show a wider therapeutic index, and lesser off-target-based toxicologic effects due to the reduced expression of the diverse PI3K isoforms in non-cancerous cells. Notably, PI3Kα and PI3Kβ isoforms are ubiquitously expressed, whereas PI3Kγ and PI3Kδ isoforms are predominantly restrained to leukocytes [225]. Among all IS PI3Ki, only three have been approved by the FDA: alpelisib, duvelisib, and idelalisib. Alpelisib, a potent and specific drug with targeted efficacy against PI3Kα isoform, in combination with fulvestrant, has displayed significant clinical benefits in hormone receptor (HR) + /HER2- PIK3CA-mutated advanced or metastatic breast cancer patients, and as a result has been FDA-approved for use in this cohort (Supplementary information 2) (Supplementary figure 1). The safety profile of this combination in breast cancer patients is favourable or manageable. Grade 1–2 adverse events include hyperglycemia, diarrhea, rash, nausea, fatigue, diarrhea, anemia, stomatitis, reduced appetitie, vomiting, anorexia, and alopecia [226, 227]. Clinical antitumor activity of alpelisib has been shown as monotherapy in breast cancer [228] and other advanced solid tumors [229, 230], as well as in combination with either trastuzumab-emtansine [231], fulvestrant [232–235], letrozole [236–239], nab-paclitaxel [240], olaparib [241], or trastuzumab + LJM716 [242], in breast cancer, and in combination with either imatinib [243], BGJ398 [244], binimetinib [245], olaparib [246], cetuximab + intensity modulated radiation therapy [247], everolimus + exemestane [248], or cetuximab + encorafenib [249, 250], in advanced solid tumors. Duvelisib, a potent and selective agent with targeted efficacy against PI3Kγ and PI3Kδ isoforms, has exhibited remarkable clinical benefits in relapsed or refractory chronic lymphocytic leukemia (CLL) or small lymphocytic lymphoma (SLL) patients after ≥ 2 prior therapies, and in relapsed or refractory FL patients after ≥ 2 prior systemic therapies, and thus has been FDA-approved for use in these cohorts (Supplementary information 2) (Supplementary figure 1). The safety profile of duvelisib in CLL, SLL, and FL patients is favourable or tolerable. The most common adverse events include diarrhea, neutropenia, pyrexia, anaemia, nausea, and cough. In CLL/SLL patients only, grade ≥ 3 immune-related toxicities consist of colitis (12%), pneumonitis (3%), increased alanine aminotransferase (ALT) (3%), and enhanced aspartate transferase (AST) (3%); in addition, the most frequently reported serious adverse event is pneumonia (15%) [251]. Clinical anticancer activity of duvelisib has been shown as monotherapy [252–258], as well as in combination with either rituximab + bendamustine [259], or fludarabine + cyclophosphamide + rituximab [260], in several hematological malignancies. Idelalisib, a specific drug with targeted efficacy against PI3Kδ isoform, has displayed significant clinical benefits as monotherapy in SLL and FL patients who have received ≥ 2 prior systemic therapies, and in combination with rituximab in CLL patients for whom rituximab singly would be considered appropriate therapy due to co-morbidities. As a result, idelalisib has been FDA-approved for use in these cohorts [261, 262] (Supplementary information 2) (Supplementary figure 1). The safety profile of idelalisib in SLL, FL and CLL patients is favourable or acceptable. The most frequent ≥ grade 3 adverse events after idelalisib monotherapy in SLL and FL patients include decreased neutrophils (25%), increased ALT (18%), pneumonia (16%), diarrhea (14%), and enchanced AST (12%) [261]; whereas the incidence of ≥ grade 3 adverse events after combination of idelalisib and rituximab in CLL patients comprise neutropenia (34%), thrombocytopenia (10%), anemia (5%), elevation in transaminases (5%), and diarrhea (4%) [262]. Clinical antitumor activity of idelalisib has been shown as monotherapy [263–269], in different hematological malignancies, and in combination with either rituximab [270–275], bendamustine [270], tirabrutinib [276], ofatumumab [277], or rituximab + bendamustine [270, 278], in chronic lymphocytic leukemia, as well as in combination with obinutuzumab in Waldenström’s macroglobulinemia [279]. Notably, dual inhibitor of PI3Kδ/casein kinase-1-ε umbralisib received its first FDA approval in 2021 for the treatment of relapsed or refractory marginal zone lymphoma (MZL) patients who had received ≥ 1 prior anti-CD20-based regimen, and relapsed or refractory FL patients who had received ≥ 3 prior lines of systemic therapy [280]. Nonetheless, due to safety concerns the FDA withdrew its approval in 2022. Several clinical studies have shown anticancer activity of umbralisib, both as monotherapy [281] and in combination [282], in different hematological malignancies. Besides, antineoplastic activity has been observed with other IS PI3Ki such as PI3Kαi taselisib and serabelisib as monotherapy/in combination with other drugs in advanced solid tumors, PI3Kβi GSK2636771 and AZD8186 as monotherapy/in combination with other agents in advanced solid tumors, PI3Kβ/δi acalisib as monotherapy in hematological malignancies, PI3Kγi eganelisib in combination in advanced solid tumors, PI3Kγ/δi tenalisib as monotherapy in hematological malignancies, PI3Kδi linperlisib as monotherapy in hematological malignancies, and PI3Kδi parsaclisib as monotherapy in hematological malignancies and in combination in advanced solid tumors (Supplementary information 2) (Supplementary table 1).

#### Dual PI3K/mTOR Inhibitors

Dual PI3K/mTORi are mostly effective against all PI3K isoforms, as well as mTORC1/mTORC2, leading to suppression of the three crucial intersections of the PAM signaling pathway. There are several ongoing clinical trials using dual PI3K/mTORi, including apitolisib, dactolisib and paxalisib as monotherapy in advanced solid tumors, gedatolisib and samotolisib as monotherapy/in combination with other drugs in advanced solid tumors, and voxtalisib monotherapy/in combination in hematological malignancies and advanced solid tumors. However, none of them has reached FDA approval (Supplementary information 2) (Supplementary table 1).

### AKT Inhibitors

Numerous drugs can specifically inhibit AKT proteins, thereby impeding overactivation of downstream proteins in PAM signalling pathway. Indeed, there are several ongoing clinical trials using AKT inhibitors, such as capivasertib, ipatasertib and M2698 as monotherapy/in combination with other drugs in advanced solid tumors, afuresertib and perifosine as monotherapy/in combination with other agents in advanced solid tumors and in combination only in hematological malignancies, and MK-2206 as monotherapy/in combination in advanced solid tumors and hematological malignancies, and TAS-117 as monotherapy in advanced solid tumors. However, none of these agents has been approved by the FDA (Fig. 10). Notably, capivasertib, a potent and selective inhibitor of all three AKT isoforms [283], is the most prominent agent against AKT in advanced solid tumors, particularly in breast cancer [284]. Indeed, in the recent phase 3 CAPItello-291 study, combination of capivasertib and fulvestrant doubled PFS compared to fulvestrant singly in HR + , HER2- breast cancer patients who have developed resistance to aromatase inhibitors and CDK4/CDK6 inhibitors [285, 286]. Thus, capivasertib represents a new valuable treatment option for these patients and is expected to receive FDA approval in due time [227] (Supplementary information 2) (Supplementary table 2).

### mTOR Inhibitors

mTOR inhibitors were the first PAM-targeting drugs to advance to the clinic [287]. Induction of mTORC1 enhances the formation of proteins, lipids, nucleotides, and decreases autophagy, resulting in cell survival, proliferation, and growth; whereas activation of mTORC2 regulates protein kinases, including AKT, leading to cell survival, and proliferation [288, 289]. Therefore, both mTORC1 and mTORC2 functions provide an important rationale for targeting mTOR complexes in tumor, although the effectiveness of some mTORC inhibitors may be compromised by a compensatory feedback loop leading to AKT activation [290]. There are three types of mTOR inhibitors: first-generation allosteric (non-competitive) mTOR inhibitors (allosteric mTORi), which inhibit mTORC1 only; second-generation ATP-competitive mTOR inhibitors (ATP-competitive mTORi), which suppress both mTORC1 and mTORC2, and third-generation bi-steric mTOR inhibitors (bi-steric mTORi), which inhibit mTORC1 only (Fig. 10).

#### Allosteric mTOR Inhibitors

Allosteric (non-competitive) mTORi act against mTORC1. Since allosteric inhibitors can only exert their function towards mTORC1 they cannot avoid the feedback loop-based induction of AKT determined by the suppression of mTORC1 [291]. Besides, allosteric mTORi can modestly reduce p4E-BP1 levels through the inhibition of (4E-BP1) phosphorylation, and consequently, cannot effectively restrain eIF4E-mediated cap-dependent translation initiation in cancer [161]. Therefore, these agents exert a weaker PAM signalling inhibition, compared to ATP-competitive mTORi, resulting in decreased antitumor activity. The first-generation allosteric mTORi includes rapamycin and its analogues, commonly known as rapalogs, which only exert a specifical inhibition towards mTORC1 [291]. Everolimus, a selective agent with targeted efficacy against mTORC1, has exhibited moderate clinical benefits in several types of cancers. Accordingly, everolimus has been FDA-approved as monotherapy in neuroendocrine tumor (NET) patients, TSC-associated SEGA patients, renal TSC-associated angiomyolipoma adult patients, and advanced-stage renal cell carcinoma (RCC) patients, as well as in combination with lenvatinib in advanced-stage RCC patients who have received a prior antiangiogenic therapy, and in combination with exemestane in postmenopausal HR + /HER2­ breast cancer patients with recurrence or progression following prior therapy with letrozole or anastrozole (Supplementary information 2) (Supplementary figure 1). The safety profile of everolimus in NET, SEGA, angiomyolipoma, RCC, and breast cancer patients is acceptable. Grade 3–4 drug-related adverse events after everolimus monotherapy in NET patients include stomatitis (9%), diarrhoea (7%), infections (7%), anaemia (4%), fatigue (3%), and hyperglycaemia (3%). The most common grade 3–4 treatment-emergent adverse event after combination of everolimus and lenvatinib in RCC patients are constipation (37%) and diarrhea (20%) [4, 292]. Clinical anticancer activity of everolimus has been shown as monotherapy in advanced solid tumors [293–298], and in combination with either exemestane [299–301], letrozole [302], exemestane + ribociclib [303], exemestane + xentuzumab [304], letrozole + trastuzumab [305], or paclitaxel + trastuzumab [306], in breast cancer, as well as in combination with either ribociclib [307], lenvatinib [292], letrozole [308], paclitaxel [309], alpelisib + exemestane [248], or letrozole + metformin [310], in advanced solid tumors. Temsirolimus, an ester of sirolimus (rapamycin), is an intravenous-administered drug that establishes a complex with FKBP12, which is then integrated into mTORC1, but not mTORC2, thereby inhibiting mTORC1 [311], and consequently suppressing the generation of proteins implicated in cell cycle [312] and angiogenesis [313]. Temsirolimus has displayed significant clinical benefits as monotherapy in RCC patients, and thus has been FDA-approved for use in this cohort (Supplementary information 2) (Supplementary figure 1). The safety profile of temsirolimus in RCC patients is acceptable. The most common temsirolimus-induced grade 3–4 adverse reactions include hypertriglyceridemia (44%), anemia (20%), hypophosphatemia (18%), lymphocytopenia (16%), hyperglycemia (16%), asthenia (11%), dyspnea (9%), neutropenia (5%), rash (5%), and pain (5%) [314]. Clinical antitumor activity of temsirolimus has been shown as monotherapy in hematological malignancies [315, 316], and RCC [287], in combination with either lenalidomide [317], etoposide + cyclophosphamide [318], or rituximab + cladribine [319], in advanced solid tumors, and in combination with either perifosine [320], capecitabine [321], bevacizumab [322], or chemotherapy [323], in advanced solid tumors. Besides, antineoplastic activity has been observed with other agents such as nab-sirolimus as monotherapy in advanced solid tumors, as well as rapamycin and ridaforolimus as monotherapy/in combination with other drugs in advanced solid tumors (Supplementary information 2) (Supplementary table 3).

#### ATP-competitive mTOR inhibitors

ATP-competitive mTORi (also known as active-site mTORi) can act against both mTORC1 and mTORC2, avoiding the feedback loop-based induction of AKT determined by the suppression of mTORC1 [291]. Notably, ATP-competitive mTORi remarkably reduce p4E-BP1 levels through the inhibition of (4E-BP1) phosphorylation, and as a result, can effectively prevent eIF4E-mediated cap-dependent translation initiation in cancer. Hence, ATP-competitive mTORi actively promote dephosphorylation on Thr46 (4E-BP1), thereby re-establishing its endogenous functions of growth suppression and pro-apoptosis [161]. Therefore, these inhibitors induce a stronger PAM signalling inhibition compared to allosteric mTORi, leading to enhanced antitumor activity [291]. This difference is mainly due to the fact that allosteric mTORi, which represent the first-generation mTORi, suppress mTORC1 indirectly by binding to FKBP12; while ATP-competitive mTORi, which represents the second-generation mTORi, suppress both mTORC1 and mTORC2 by inhibiting the mTOR kinase directly [324]. In fact, in regard to allosteric mTORi, there is a catalytic cleft within the FKBP12-rapamycin-binding (FRB) domain of mTOR, enabling limited access to 4E-BP1 as a substrate; whereas ATP-competitive mTORi are able to bind deeper inside the catalytic cleft, thereby abolishing the capacity to phosphorylate 4E-BP1 [161]. There are several ongoing clinical trials using ATP-competitive mTORi including sapanisertib as monotherapy in hematological malignancies and advanced solid tumors, as well as in combination in advanced solid tumors, especially breast cancer, onatasertib and AZD8055 as monotherapy in advanced solid tumors, vistusertib monotherapy in hematological malignancies and in combination in advanced solid tumors, and OSI 027 monotherapy in hematological malignancies. However, mainly due to toxicity, none of them has reached approval by the FDA (Supplementary information 2) (Supplementary table 3).

#### Bi-steric mTOR inhibitors

Since the clinical benefits of ATP-competitive mTORi are obstacled by toxicity, a third-generation mTORi named bi-steric mTORi (also known as RapaLinks) that selectively inhibit mTORC1 and not mTORC2 has recently been designed [325]. These inhibitors, which contain a rapamycin-like core moiety covalently linked to an mTOR active-site inhibitor [326], are termed bi-steric due to their simultaneous engagement of the allosteric FRB domain and orthosteric catalytic domain of mTOR, in order to deepen the suppression of mTORC1 while also retaining selectivity for mTORC1 over mTORC2 [325]. Importantly, bi-steric mTORi such as RMC-4627, have shown potent and selective inhibition of 4E-BP1 phosphorylation leading to tumor regression in B-cell acute lymphoblastic leukemia xenografts [327] and breast cancer xenografts [325, 326]. Besides, bi-steric mTORi RMC-6272-mediated inhibition of mTORC1 in ER + /HER2- breast cancer has displayed significant efficacy in hormone therapy-resistant acquired patient-derived xenografts, and in patient-derived xenografts from CDK4/6 inhibitor-resistant patients [328]. Notably, these compounds cause less relief of AKT-dependent feedback inhibition of RTK expression, which notoriously results in RTK receptor reactivation-induced adaptive resistance, and toxicity in comparison to ATP-competitive mTORi. Also, bi-steric mTORi display a longer dwell-time on target compared to other types of mTORi, and thus can be regularly used in intermittent dosing schedules [327, 325]. Interestingly, bi-steric mTORi RMC-5552 [329] in combination with Ras inhibitors has exhibited clinical anticancer activity with a favourable safety profile in relapsed or refractory RAS-mutated solid tumor patients [330]. Thus, according to these pre-clinical and clinical data bi-steric mTORi can be considered a major candidate to be used as mTORi in future cancer treatments (Supplementary information 2) (Supplementary table 3).

### PDK1 Inhibitors

PDK1, also known as PDPK1, is a crucial regulator of PAM signalling pathway due to its phosphorylation on AKT. Indeed, PDK1 can exert a potential role in developing chemoresistance in various types of malignancy [331]. Thus, it is reasonable to suggest that PDK1 inhibition, singly or in combination with other PAM inhibitors, could contribute to the enhancement of antitumor efficacy in different types of human cancer (Fig. 10). Several PDK1 inhibitors have shown great potential as anticancer drugs in vitro and in vivo, but none of them has yet reached the clinic. This is mainly due to the fact that PDK1 drug discovery efforts have been somehow obstracted by the remarkable attention given to other more characterized kinases such as PI3K, AKT and mTOR. Nevertheless, an increasing number of patent applications have reported on putative PDK1 inhibitors since its discovery [332] (Supplementary information 2).

## Resistance

The PAM signaling pathway involves numerous feedback loops, compensatory pathways, and crosstalk nodes with other signal transduction axes that hamper the inhibition of PI3K, AKT, mTORC1, mTORC2, and PDK1 in cancer. Indeed, recent studies have reported that short administration of drug inhibitor therapies can determine feedback loop inductions that consecutively reduce the overall response rate (ORR). Additionally, chronic administration of inhibitor-based therapies can lead to the accumulation of slow-cycling cells that can possibly gain genetic mutation contributing to drug resistance [333]. The major mechanisms of resistance to PAM signalling-targeted inhibitors are revised below.

### Mechanisms of resistance to PI3K Inhibitors

Even though PAM-targeted agents, particularly PI3K drug inhibitors, have demonstrated significant therapeutic activity in human cancer, acquired and intrinsic resistance has hindered their clinical efficacy [334–336]. Thus, a rationale for alternative clinical strategies could be provided by an accurate understanding of the biochemical mechanisms whereby resistance to PI3Ki occur. Various targeted therapies can induce several possible mechanisms of resistance to PI3Ki as described below.

#### RTK Reactivation

RTKs are often induced following treatment with specific inhibitors, and as a result, they promote the activation of the PAM and Ras/MEK/ERK signalling cascades [163, 337]. This feature is determined by the loss of the suppression that AKT exerts on mTORC1 and FOXO, leading to RTK transcription, and reinduction of these signal transduction axes [338, 163]. When the PAM signaling pathway is activated, AKT-induced phosphorylation and blockage of transcription factor FOXO in the cytoplasm reduces the activity of FOXO molecules, and consequently, ceases the induction of FOXO target genes related to the promotion of cell cycle arrest or apoptosis [339]. Concurrently, this mechanism hampers the capacity of FOXO to regularly transcribe different RTKs, representing an indirect feedback mechanism that limits extracellular stimuli-mediated induction of RTKs. Conversely, when PI3K is inhibited, AKT-induced FOXO phosphorylation is suppressed, enhancing the expression of FOXO in the nucleus, which leads to the stimulation of RTKs and partial restoration of PIP3 activity. Thus, PAM signaling induction cannot be thoroughly suppressed, since PIP3 level is preserved, resulting in cell proliferation.

PI3K p110β is the major PI3K isoform that drives PI3K-mediated signaling in PTEN-null cancers [340]. PI3K p110β inhibitor AZD8186 treatment of PTEN-null cells remarkably reduces PI3K signal transduction and cancer cell survival. Accordingly, specific inhibition of PI3K p110α in these tumor cells displays no effect since their type of cancer only depend on PI3K p110β signaling. Nevertheless, downregulation of AKT and mTOR in these tumor cells is transient, since mTOR downregulation leads to FOXO de-repression, and consequently, RTK transcription, resulting in PI3K p110α-induced AKT signal transduction cascade. Hence, targeted suppression with an IS PI3Ki (e.g. p110β) induces AKT and mTOR signaling due to the reinduction of the alternative PI3K isoform (e.g. p110α). Notably, this reciprocal activation has been successfully abrogated through the concurrent inhibition of PI3K p110α and PI3K p110β, leading to a more significant anticancer efficacy compared to monotherapy with either PI3K inhibitors [341].

Clinical studies with PI3Kα inhibitor alpelisib have provided evidence that activation of alternative signaling pathways may contribute to primary resistance or early emergence of resistance in cancer patients. Gene expression profile analysis of paired pre-treatment and on-treatment tumor samples, collected from patients treated with alpelisib as a single agent or in combination with an aromatase inhibitor, has shown that ESR1 and its target gene PGR are among the most highly induced genes upon PI3K inhibition. This supports the notion that ER mRNA increases during PI3K inhibition, and suggests that activation of this compensatory pathway may decrease antitumor efficacy [342]. Besides, recent studies have also demonstrated that ESR1 activating mutations expand in number and allele fraction after combination treatment of alpelisib and aromatase inhibitor in HR + metastatic breast cancer patients, and their presence is associated with resistance [239] Table 1.

**Table 1:** Pre-existing and acquired mutations implicated in clinical resistance to PAM inhibitors

Mutations implicated in clinical resistance to PAM inhibitors						
**PI3K Inhibitors**						
**Agent**	**Type of mutations**	**Genes mutated**	**P/A prior to treatment**	**Type of resistance**	**Disease setting**	**References**
Alpelisib	*ESR1* activating mutations	ESR1 E380Q	P	Primary/Secondary	*PIK3CA*-mutated metastatic breast cancer	239
		ESR1 L363Q	P			
		ESR1 F461V	P			
		ESR1 H524L	P			
		ESR1 Y537N	P			
		ESR1 Y537C	P			
		ESR1 D538G	P			
Alpelisib	*PTEN* copy number loss and loss of function mutations	PTEN D97H	P	Primary/Secondary	*PIK3CA*-mutated metastatic breast cancer	239, 351
		PTEN L108H	P			
		PTEN A126S	P			
		PTEN R130*	P			
		PTEN M134I	A			
		PTEN	A			
		L139Nfs*3	A			
		PTEN T167P	A			
		PTEN Q214R	A			
		PTEN E242G	A			
		PTEN S339fs	A			
		K342_splice	A			
Idelalisib	*PIK3R1* inactivating mutation	PIK3R1	A	Secondary	CLL	352
Idelalisib	*MAP2K1, BRAF* and *KRAS* activating mutations	MAP2K1 Q56P	A	Primary	CLL	361
		MAP2K1	A			
		E203K	A			
		KRAS G13D	A			
		KRAS Q22K*	A			
		BRAF G469A	A			
		BRAF	A			
		N581_splice	A			
		BRAF V600E	A			
		BRAF K601E	A			
Idelalisib	*BIRC3* inactivating mutation	BIRC3	A	Secondary	CLL	352
**mTOR Inhibitors**						
**Agent**	**Type of mutations**	**Genes mutated**	**P/A prior to treatment**	**Type of resistance**	**Disease setting**	**References**
Everolimus	Loss-of-binding/drug resistance mutation	MTOR F2108L	A	Secondary	Metastatic ATC	375

This table includes mutations and copy number changes in primary patient samples following clinical treatment with PAM inhibitors. Expression level changes in clinical samples and resistance mutations generated in cell lines or ex vivo culture models are not included

*A/P* Mutation absent/present, *P* Present (mutation present prior to treatment), *A* Absent (mutation absent prior to treatment), *ATC* Anaplastic thyroid cancer

IGF1R upregulation has been identified as a potential resistance mechanism to PI3Kδ inhibition in CLL patients without activating MAPK pathway mutations. Indeed, IGF1R expression is elevated, both at baseline and at the time of disease progression, in RNA samples of patients who develop resistance to idelalisib compared to a set of previously untreated CLL samples. This indicates a potential role for IGF1R signalling in resistance to PI3Kδ inhibitors [343]. An expanded analysis of idelalisib-refractory patients, pooled from three clinical trials, has further demonstrated IGF1R overexpression in 87.5% of patients, whose paired RNA from treatment initiation and refractory time point were available [344]. Notably, this analysis has confirmed an enrichment for MAPK pathway variants in the primary refractory subset, further discussed below; whereas IGF1R upregulation was present at the point of secondary resistance. This emphasizes the importance of understanding the specific molecular pathways involved in different settings of resistance to define strategies to overcome treatment challenges.

#### Acquired mutation and amplification of PI3K genes

Acquired mutation and/or amplification of PIK3CA or PIK3CB, which often result in enhanced overall PI3K activity, are notorious to increase resistance to PI3K-targeted inhibitors [345, 346]. Notably, phosphorylation of PI3K regulatory subunit p85 also plays an important role in developing resistance against PI3K inhibitors. Besides, resistance to PI3K inhibition is also conferred by the existence of a regulatory loop between PI3K p85 and Src [347]. When PTEN is absent, cancer cell proliferation mainly depends on PI3K p110β isoform activity [348, 349]. PTEN loss singly is unable to generate resistance to pictilisib, a class I PI3K inhibitor; nevertheless, amphiregulin can significantly increase the resistance, leading to enhanced EGFR signaling [347]. Moreover, it has been shown that continuous mTORC1 activity positively correlates with intrinsic resistance to PI3K p110α inhibitors. In fact, growth factors including IGF1 and neuregulin 1 are known to induce mTOR, thereby mediating PI3K p110α inhibitor resistance [350].

Analysis of tumor biopsies from a PIK3CA-mutated metastatic breast cancer patient, enrolled on a study of alpelisib, has demonstrated the clinical relevance of progressive loss of PTEN expression and consequent gain of dependency on PI3K p110β isoform [351]. In line with this, evidence that dependence on PI3K p110β isoform is of clinical relevance has also emerged from the analysis of multiple tumor biopsies from a patient with PIK3CA-mutated metastatic breast cancer, enrolled in a human study of alpelisib. This patient first achieved a long clinical response and then suffered a relapse with new lung metastases. At the time of death, after metastatic sites were analyzed, all lesions displayed a copy loss of PTEN not present in the pre-treatment sample. Besides, metastases that progressed on therapy had additionally gained various PTEN alterations, which consequently, resulted in its loss of expression. The same mechanism has been identified in a longitudinal analysis of tumor and plasma circulating tumor DNA from patients treated with alpelisib and an aromatase inhibitor. Indeed, loss-of-function PTEN mutations were observed in 25% of patients with resistance [239] Table 1. Recently, an acquired mutation in the PIK3R1 gene has been reported in a CLL patient who became treatment-refractory to idelalisib after 4.4 years [352] Table 1.

#### Other mechanism of resistance

Other mechanism of resistance such as insulin signalling and PI3K reactivation [353, 354], altered cell metabolism [355, 356], interactions between PAM pathway and other pathways [357, 358], and cellular plasticity [359, 360] can contribute to drug tolerance (Supplementary information 3).

Constitutive mutational MAPK pathway activation has been identified as a clinical mechanisms of primary resistance in CLL. In a recent analysis of patient samples collected from trials involving various PI3K inhibitors, but mostly idelalisib, 60% of CLL patients who had no initial response to therapy have shown activating MAPK pathway mutations, which are specifically found in MAP2K1, KRAS and BRAF [361] Table 1. Recently, a study focusing on acquired resistance to idelalisib in CLL has revealed, among other alterations, the acquisition of a BIRC3 mutation, suggesting the activation of the NF-κB pathway as another potential bypass mechanism [352] Table 1. Also, there is emerging evidence for the role of secreted factors, primarily IL-6, in the development of resistance to PI3K inhibitors, with significant clinical relevance in primary and secondary resistance to idelalisib in MZL patients [362].

### Mechanisms of resistance to AKT Inhibitors

Resistance to AKT inhibitors has been the object of several studies in oncology research [363, 364]. AKT inhibition has been reported to activate phosphorylation and expression of several RTKs, and these RTK signaling can lead to the attenuation of their anticancer activity in breast cancer cells, suggesting that combinatorial suppression of AKT activity and HER kinase activity exerts a more significant efficacy compared to mono-agent therapies [365]. Interestingly, AKTE17K mutation is considered a promising biomarker since clinical data report a correlation between the presence of this alteration and a response to AKT inhibitor capivasertib [366]. Importantly, upregulation of AKT3, which is epigenetically regulated by extra terminal domain proteins and bromodomain proteins, has been found to confer AKT inhibitor MK-2206 resistance in breast cancer, providing a rationale for developing future drug therapies targeting AKT3 in order to evade this acquired resistance [367]. Mutations that confer resistance to one allosteric AKT inhibitor do not necessarily determine resistance to other AKT allosteric inhibitors. In fact, clinically relevant activating AKT1Q79K mutation can confer resistance to miransertib, but not to MK-2206, further emphasising the importance of understanding AKT genotype for an appropriate treatment selection [368].

### Mechanisms of resistance to mTORC Inhibitors

Various mechanisms are involved in the failure of first-generation allosteric mTORi [369]. Rapalogs are known to inhibit only some of the mTORC1 functions since they are FKBP12-dependent allosteric inhibitors. In addition, rapamycin generally exerts its activity against unstable mTORC1 substrates including ribosomal protein S6K, instead of stable substrates such as eIF4E-binding protein 4E-BP1, and consequently, only exerts a partial inhibition of the mTORC1-mediated protein synthesis [370]. Since 4E-BP1 is the substrate whereby mTORC1 regulates cell proliferation, the inadequate inhibition of this strong and stable molecule can be considered a plausible explanation of the modest antiproliferative efficacy of rapamycin on tumor cells [371, 372]. Another mechanism of escaping rapalogs inhibition is the compensatory activation of various signal transduction pathways due to mTORC1-induced suppression of negative feedback loops. The major effect is determined by PAM signaling activation, since rapalogs mitigate the mTORC1-induced negative feedback inhibition of insulin and IGF1 receptor mitogenic signalling [373]. Indeed, S6K activates phosphorylation of insulin receptor substrate 1 (IRS1), thereby leading to IRS1 suppression, or IRS1 degradation, or IRS1 binding to IGF1 receptor. Thus, rapamycin treatment enhances the level of IRS1 and induces the PAM axis by insulin and IGF1 receptor signaling [374]. Further studies have described other mechanisms involved in the failure of allosteric mTORi (Supplementary information 3).

Clinically, allosteric mTOR inhibitors have shown activity in various malignancies with mTOR-pathway activating mutations, and in some cases additional mutations within the pathway have been linked to acquired resistance. For instance, a patient with metastatic anaplastic thyroid cancer who had an exceptional response to everolimus lasting 18 months prior to treatment was found to have a nonsense mutation in the tumor suppressor gene TSC2, leading to mTOR pathway activation, and at progression acquired a mutation in MTOR that conferred resistance to allosteric mTOR inhibition. Importantly, the acquired MTOR mutation did not confer resistance to ATP-competitive mTOR inhibitors [375] Table 1. Several studies have demonstrated an enrichment for mTOR pathway mutations in responders or exceptional responders to these agents. Absence of mTOR pathway dependence may conversely be considered a mechanism of primary resistance. Examples include a study of rapalog-treated metastatic RCC patients, which identified an enrichment for MTOR, TSC1 and TSC2 mutations in responders [376], a report of a single exceptional responder to pazopanib and everolimus with urothelial carcinoma who was found to have two activating mutations in MTOR [377], and two studies of patients with perivascular epithelioid cell tumors (PEComas) who responded to sirolimus, some of which demonstrated loss of TSC1/TSC2 or TSC2 aberrations [378, 379]. As a counterpoint it should be noted that a substantial proportion of responders in some of the above-mentioned studies [376] had no identifiable alterations in mTOR pathway genes. Likewise, in the setting of postmenopausal nonsteroidal aromatase inhibitor-resistant ER + HER2- breast cancer patients treated with everolimus and exemestane, comprehensive sequencing analysis did not identify predictive biomarkers, although patients with PIK3CA exon 9 mutations experienced a quantitative benefit compared to patients with PIK3CA exon 20 mutations [380].

Several mechanisms of resistance can hamper the efficiency of second-generation ATP-competitive mTORi [369]. Some kinase domain mutations, including the methionine 2327 isoleucine substitution (M2327I), enhance mTOR catalytic activity, and therefore, both mTORC1/mTORC2 activeness. Consequently, concentration of ATP-competitive mTORi (e.g. AZD8055) necessary to suppress mTORC1 and mTORC2 substrates must be higher, compared to those required for wild-type mTOR kinase [326]. Tumor cells can also gain resistance to ATP-competitive mTORi by exerting downregulation of 4E-BPs, such as EIF4E-BP1, and EIF4E-BP2. This results in an enhanced EIF4E/4E-BPs ratio, which limits the suppressive action of these agents on the translation of EIF4E-susceptible mRNAs, and thus, decreases their anticancer efficacy [381, 382]. Interestingly, there is an inverse correlation between 4E-BP1 expression and Snail level in tumor cell lines and in clinical biospecimens. Indeed, Snail can suppress 4E-BP1 transcription by binding to three E-boxes located in the human 4E-BP1 promoter. Accordingly, ectopic expression of Snail in tumor cell lines remarkably inhibits 4E-BP1 expression, and consequently, reduces the anti-proliferative effect of ATP-competitive mTORi [383]. Also, ATP-competitive mTORi are more efficacious than rapalogs in numerous tumor models since they can act against both mTORC1 and mTORC2, avoiding the feedback loop-based induction of AKT caused by the suppression of mTORC1 [373]. Nevertheless, ATP-competitive mTORi induce overactivation of the Ras/MEK/ERK pathway through PI3K-independent feedback loops. Indeed, in multiple myeloma cells, torkinib induces ERK through an mTORC1/eIF-4E/Raf pathway [384]. More studies have reported different mechanisms involved in escaping ATP-competitive mTORi (Supplementary information 3). Interestingly, third-generation bi-steric mTORi can drive cancer regression more significantly than ATP-competitive mTORi, due to their potent and selective inhibition of 4E-BP1 phosphorylation, while minimizing adaptive resistance due to the relief of feedback inhibition of RTK expression and signaling, as well as undesirable toxicity and glucose intolerance [369]. Thus, bi-steric mTORi may be useful for treating cancers that are driven by activated mTORC1 [325, 327].

## PAM signaling in immunology and immunotherapy

PAM signaling has emerged as a dominant regulator of the immune response. Indeed, PAM pathway-mediated control of the immune system is finely-tuned, allowing for precise mobilization or suppression of immune cell subsets through tightly-regulated signalings. The role of the PAM axis in immunoregulation and the possible roles for PAM-directed therapies in combination with immunotherapy are outlined below.

### Effects of PAM signaling on the immune system and tumor microenvironment

PAM signaling plays crucial roles in immune cell maturation, differentiation, recruitment, and survival. However, each layer of the PAM signaling axis exerts its own unique control over the constituent subsets of the immune system.

PI3K, especially in the leukocyte-predominant PI3Kδ and PI3Kγ isoforms, is an important regulator of immune homeostasis. PI3Kδ is unique among the PI3K isoforms in its near-monopoly over B-cell receptor signaling, more specifically in CLL and mantle cell lymphoma (MCL). Several studies have shown that, in explanted human regulatory T-cells (Tregs), murine Tregs, and murine models of inactivated PI3Kδ, immunosuppressive Tregs are exquisitely reliant on PI3Kδ [385–387]. Consequently, inhibition of PI3Kδ can suppress the innate and adaptive immune system, predisposing patients to serious infections, including Pneumocystis jirovencii and cytomegalovirus. Accordingly, the observation that immunosuppressive Tregs are also uniquely dependent on PI3Kδ may begin to explain why autoimmune pneumonitis and colitis often accompany PI3Kδ inhibition [388]. Like PI3Kδ, the PI3Kγ isoform is active in lymphocytes. PI3Kγ and its regulatory subunits PIK3R5 and PIK3R6 are uniquely overexpressed within the myeloid compartment, where PI3Kγ serves as the principal catalytic PI3K [389]. Direct inhibition of PI3Kγ has been shown to diminish the immunosuppressive myeloid phenotype, notably shifting myeloid cells away from the immunosuppressive M2-like phenotype and towards the immunostimulatory M1-like phenotype [389–391]. Both PI3Kδ and PI3Kγ have also been implicated in the activity of myeloid-derived suppressor cells (MDSCs), and blocking the two isoforms impairs MDSC immunosuppression [392].

AKT plays key roles in both innate and adaptive immunity. In particular, T-cell receptor (TCR) signaling promotes T-cell survival and cell cycle progression through the activation of AKT-dependent transcriptional programs [393]. AKT1/2 promotes terminal differentiation of CD8 + T-cells and blunts the development of central memory and effector memory CD8 + T-cell populations [394, 395]. Additionally, AKT is also important in CD4 + T-cells, where it guides T helper (Th) cell differentiation in an isoform-specific manner [396]. T-cell activation via TCR and CD28 co-stimulation promotes downstream induction of T helper type 1 (Th1) cell-mediated cytokines IL-2 and IFN-γ in an AKT-dependent manner. Notably, regulation of T helper type 2 (Th2) cell-induced cytokines IL-4 and IL-5 is AKT-independent. AKT has also been shown to control Treg homeostasis through inhibitory phosphorylation of FOXO1, which in turn promotes Treg suppression [397]. Titration of this signaling axis modulates immune tolerance, implying that upstream inhibition of PI3K may disrupt tumor immune tolerance [398]. Additionally, it has recently been shown that inhibitory signaling through PD-1 restricts activation of Treg-mediated immunosuppression in an AKT-dependent manner [399]. Moreover, AKT is also known to modify myeloid cell fate and survival through its regulation of β-catenin, NF-κB signaling, and cytokine production [400]. Furthermore, isoform-dependent AKT signaling is a key determinant of M1/M2 macrophage polarization [401, 402], and AKT is required for the maturation of bone marrow-derived dendritic cells [403]. mTOR-mediated regulation of the immune system is complex and is comprehensively reviewed elsewhere [404, 405]. mTOR is the catalytic domain of the complementary mTORC1 and mTORC2 complexes. These complexes co-regulate cellular metabolism, the primary mechanism by which mTOR regulates the immune system, as mobilizing immune cells is energetically expensive and requires metabolic reconfiguration. mTORC1, in particular, has been shown to regulate memory T-cell differentiation [406] and establish the function of immunosuppressive Tregs [407]. Therefore, even though rapamycin-induced inhibition of mTORC1 promotes Treg development from naïve T-cells [408] and subsequent Treg expansion [409, 410], rapamycin can still promote autoimmunity through the suppression of Treg function [407]. The role of mTORC2 is more subtle, but it has been shown to play an inhibitory role in the generation of CD8 + memory T-cells, driven by mTORC2-induced inhibition of FOXO-mediated IL-15R expression [411]. Importantly, mTORC1 and mTORC2 are also directly involved in regulating innate immunity. mTORC1 controls monocyte/macrophage-mediated inflammation by regulating production of inflammatory cytokines via inhibition of NF-κB (downstream PI3Kγ) [412]. Again, mTORC2 plays a complementary role, modifying chemotaxis in mast cells and neutrophils. Besides, mTORC2 downregulates IL-12 in dendritic cells, due to inhibition of FOXO1 [413], and plays an important role in IL-4-dependent, alternative activation of M2 macrophages [414, 415]. Cancer cell-autonomous activation of PAM signaling has been shown to promote expression of immunosuppressive cytokines, chemokines, and immune checkpoints, which can be reversed through pharmacologic inhibition of PAM signaling [416]. mTOR regulates the expression of inflammatory cytokines such as IL-10, IL-12, TGF-β, and TNF [417, 418]. Activation of neoplastic PAM signaling also promotes expression of vascular endothelial growth factor (VEGF), an important mediator of angiogenic signaling that also acts as a chemoattractant of immunosuppressive MDSCs [419] and Tregs [420]. Reciprocally, inhibition of mTOR with everolimus, has been shown to partially phenocopy the anti-angiogenic effect of VEGF inhibitors [421]. Moreover, due to the role of PAM and especially mTOR in metabolic regulation, PAM inhibition within nutrient-constrained tumor microenvironments can disrupt the balance between CD4 + /CD8 + T-cells, Tregs/T helper 17 (Th17) cells, and M1/M2 macrophages [417]. Finally, many studies have associated activated PAM signaling with increased PD-L1 expression [422, 423], and PAM inhibition has been shown to be capable of reducing PD-L1 expression in PTEN-driven breast and CRC cells [424].

### Targeting PAM Signaling alongside immunotherapy

Over the last decade, the treatment of cancer has been revolutionized by the introduction of immunotherapies. New agents that target the PD-1/PD-L1 and CTLA-4 immune checkpoints, known as immune checkpoint inhibitors (ICIs), and cellular therapies designed to seek out cancer cells, have demonstrated remarkable efficacy. Yet, despite these successes, many cancers do not respond or respond incompletely to immunotherapy, underscoring the importance of better understanding the mechanisms underlying the resistance to these agents.

#### Immune checkpoint inhibitors (ICIs)

Many of the mechanisms that underlie ICI treatment failure, including defective neoantigen presentation, T-cell resistance, excess immunosuppressive cytokines, and intratumoral inhibitory MDSCs or Tregs [425], are associated with PAM signaling; and thus, may be reversible with PAM inhibition. Indeed, there is increasing evidence that targeting the PAM pathway can reduce the production of immunosuppressive cytokines and restrict the proliferation and penetration of immunosuppressive Tregs and MDSCs, thereby boosting the anti-tumor effects of immune checkpoint blockade [389, 426, 427]. Activation of oncogenic pathways like PAM and MAPK promote transcriptional upregulation of neoplastic PD-L1. Accordingly, therapies targeting PAM and upstream kinase signaling have been shown to reduce intrinsic neoplastic PD-L1 expression. In PTEN-driven murine models of melanoma, the efficacy of both anti-PD-1 and anti-CTLA-4 antibodies has been improved by co-inhibition of PI3K [427]. In syngeneic and genetically-engineered mouse models of lung cancer, inhibition of mTOR bolsters the effects of PD-1 blockade [422]. Similar results have been reported with anti-PD-1 and anti-CTLA-4 therapies in breast cancer [428]. Combining PAM inhibition with ICI treatment decreases Treg populations and enhances the CD8 + memory T-cell response in murine models and in vitro patient cell assays [429]. Many early-phase clinical studies have been testing the aforementioned principles by assessing the tolerability and efficacy of combined PAM inhibition and immune checkpoint inhibition, primarily in advanced solid tumors, and lymphomas [430, 206]. Some of these clinical trials are highlighted below. Phase 1/2 clinical trial NCT03131908 is studying the combination of PD-1 inhibitor pembrolizumab and PI3Kβ inhibitor GSK2636771 in PD-1 refractory patients with metastatic melanoma and PTEN loss [431]. Besides, phase 1/2 clinical trial NCT04688658 is focussing on the combination of PD-1 inhibitor nivolumab and PI3Kδγ inhibitor duvelisib in patients with unresectable melanoma that have already progressed on PD-1 therapy, with clinical response in at least one patient in preliminary results [432]. Moreover, phase 2 clinical trial NCT03484819 is evaluating the combination of nivolumab and PI3Kαδ inhibitor copanlisib in relapsed/refractory diffuse large B-cell lymphoma or primary mediastinal large B-cell lymphoma. This clinical study is also trying to characterize the effects of this combination on the tumor microenvironment and immune response [433]. Furthermore, phase 1 clinical trial (NCT03884998) is investigating the the combination of nivolumab and pan-PI3K inhibitor copanlisib in patients with Richter’s transformation, with to date a favorable safety profile [434].

#### Chimeric antigen receptor (CAR) T-cell therapy

Another emerging pillar of immunotherapy is cellular therapy, particularly chimeric antigen receptor (CAR) T-cell therapy, where synthetically-engineered receptors direct pre-harvested T-cells to cancer cells expressing a select antigen. There is evidence that PAM-directed therapies could serve as a novel modifier to CAR-T cell therapy, possibly at the point of treatment [435], but primarily as a cell culture additive during T-cell expansion [430, 436]. Mechanistically, it is reported that anti-CD3/CD28 stimulation of T-cell receptor during CAR T-cell manufacturing process induces PAM signaling, resulting in terminal T-cell differentiation and decreased CAR T-cell persistence [430]. Earlier studies associate PAM activation with CAR T-cell decreased persistence and showed that PI3K inhibition with the non-selective PI3K inhibitor LY294002 was able to restrict CAR T-cell differentiation. This enables the maintenance of a less-differentiated state and ultimately improving persistence in vivo [437]. Subsequently, other groups have shown that targeted PI3K inhibition, focusing on the PI3Kγ and PI3Kδ isoforms, is capable of decreasing the expression of T-cell exhaustion markers PD-1 and Tim-3 while upregulating the lymph node homing marker CD62L [438], decreasing the expression of immune checkpoints, and increasing the yield of T-stem cell memory and central memory CD8 + CAR T-cells [439, 440]. These initial observations in CAR T-cell therapy may also be applicable to the growing field of bispecific antibodies, some of which have recently been approved for the treatment of B-cell non-Hodgkin's lymphomas.

## Conclusion

PI3K inhibition is considered a major target for antitumor therapy. Unfortunately, past and present clinical trials using PI3Ki have exhibited modest anticancer activity mainly due to resistance to PI3K inhibition and insufficient target inhibition at tolerated doses. Indeed, over the last few decades, pan-PI3Ki and dual PI3K/mTORi, not only have demonstrated limited efficacy but also are associated with significant side effects. As a result, few PI3Ki have been approved by the FDA. Since selective isoforms of PI3K perform pivotal functions in the biology of specific cancer subtypes, the present-day focus is centered on the development of IS PI3Ki. Of late, IS PI3Ki have shown promising results in several clinical trials, largely exhibiting better target specificity and toxicity profiles as compared to conventional pan-PI3Ki and/or dual PI3K/mTORi. PAM pathway inhibitors have produced limited therapeutic efficacy and significant treatment-related toxicity particularly when combined with standard treatments or other targeted agents. Hence, most PAM pathway inhibitors are unsuitable for adoption as mainstream oncological treatment strategies. For this reason, combination therapy with two or more agents in conjunction with other therapeutic strategies, such as surgery, hormonal therapy, and other antitumor drugs, has been the trend in recent years. In line with this, combination of PI3Ki with ncRNAs, or inhibitors of crucial signaling nodes in other cross-interacting pathways, may represent a future approach to effectively suppress the PAM pathway, whilst minimizing the risk of developing drug resistance and occurrence of adverse events. In future studies it would be ideal to identify stable biomarkers for patient stratification, according to cancer types and genetic profiles, to better benefit from PI3K inhibition. The mechanism exerted by PI3Ki has not been thoroughly elucidated, and will require further studies to properly delineate its pros and cons as part of the endeavour to personalize oncological therapeutics. The design of novel PI3K inhibitors structurally-based on different binding sites would undoubtedly improve specificity, and reduce toxicity of IS PI3Ki. Therefore, demystifying the mechanisms of PI3K signaling and PI3K inhibition is essential to improve combination strategy, and patient selection, which would in turn enhance the efficacy of these cancer drugs.

Inhibition of AKT can potentially exert remarkable antineoplastic effects, although dose-limiting toxicity and insufficient knowledge of the different isoforms have impeded its successful pharmacological usage. Indeed, pharmacodynamic markers of AKT inhibitors have shown incomplete target modulation. Nowadays, there are no approved biomarkers that can predict therapeutic responses to specific AKT inhibitors before using a particular agent. Thus, it is not possible to predict which patients can have adverse effects from AKT inhibition. Targeting AKT is considered a critical research area in clinical oncology, and possibly, in future studies, AKT inhibitors in combination with synergistic cytotoxic drugs could probably improve their clinical efficacy.

Several classes of mTORi have been developed, but only everolimus and temsirolimus have obtained approval as therapy for human tumors. The clinical application of these agents is impeded by resistance determined through a variety of molecular mechanisms common to the different class of drugs, suggesting that co-targeting alternative pathways may be a more feasible strategy than improving the action of mTORi. Importantly, pre-clinical and clinical data suggest that novel bi-steric mTORi can drive cancer regression more significantly compared to other mTORi, by enhancing inhibition of 4E-BP1 phosphorylation, and reducing adaptive resistance due to the relief of feedback inhibition of RTK expression. Thus, bi-steric mTORi may be the most promising inhibitors in treating activated mTORC1-driven cancers. Also, developing novel therapeutic combinations could lead to the identification of molecular factors resistant to each class of drugs to improve the selection of patients who may benefit from a specific treatment. Besides, optimisation of treatment sequence using different agents may be useful to delay or overcome resistance to mTOR inhibitors.

Additionally, PAM signaling exert a fine control over the immune system-mediated functions, including antitumor response. Ongoing studies are evaluating the impact of PAM-targeted treatments on cancer response, immune reaction and their synergy with immune-based cancer therapies.

The potential of PAM inhibitors clearly depends on the combinatorial strategies. However, the main difficulties are: 1) defining the ideal combination for each patient, and 2) managing toxicity of combination approaches that necessitate innovative scheduling. In addition, the biomarker development is challenged by the complex interactions of the pathway and in general multiple co-existing alterations that help to maintain cancer survial. Finally, we must also improve our understanding of the effects determined by PAM signaling pathway inhibitors on the tumour microenvironment for plausible drug combinations with specific immunotherapies.

### Supplementary Information

**Additional file 1: Supplementary figure 1.**

**Additional file 2: Supplementary information 1. **

**Additional file 3: Supplementary information 2. **

**Additional file 4: Supplementary information 3. **

**Additional file 5: Supplementary table 1. **

**Additional file 6: Supplementary table 2. **

**Additional file 7: Supplementary table 3. **

## Data Availability

Not applicable.

## Abbreviations

CAR: Chimeric antigen receptor
CLL: Chronic lymphocytic leukemia
CRC: Colorectal cancer
EGFR: Epidermal growth factor receptor (ERBB)
EMT: Epithelial-mesenchymal transition
ER: Estrogen receptor
FDA: Food and Drug Administration
FGFR: Fibroblast growth factor receptor
FRB: FKBP12-rapamycin-binding
HGF: Hepatocyte growth factor
HNSCC: Head and neck squamous cell cancer
HR: Hormone receptor
ICIs: Immune checkpoint inhibitors
IGF1: Insulin-like growth factor 1
INSR: Insulin receptor
IRS1: Insulin receptor substrate 1
LTK: Leukocyte receptor tyrosine kinase
MCL: Mantle cell lymphoma
MDSCs: Myeloid-derived suppressor cells
MCL: Mantle cell lymphoma
MZL: Marginal zone lymphoma
NET: Neuroendocrine tumor
NSCLC: Non-small-cell lung cancer
ORR: Overall response rate
PAM: PI3K/AKT/mTOR
PDGFR: Platelet-derived growth factor receptor
RCC: Renal cell carcinoma
SCLC: Small-cell lung cancer
SEGA: Subependymal giant cell astrocytoma
SLL: Small lymphocytic lymphoma
TCR: T-cell receptor
Tregs: Regulatory T-cells
VEGF: Vascular endothelial growth factor
VEGFR: Vascular endothelial growth factor receptor
