# Supplementary material for: PI3K/AKT/mTOR signaling transduction pathway and targeted therapies in cancer
Source: Mol Cancer. 2023 Aug 18;22:138. doi: 10.1186/s12943-023-01827-6 (PMC10436543; doi:10.1186/s12943-023-01827-6)
Supplement: Supplementary file 1 — Additional file 1: Supplementary figure 1. [file 12943_2023_1827_MOESM1_ESM.pptx]

## Slide 1
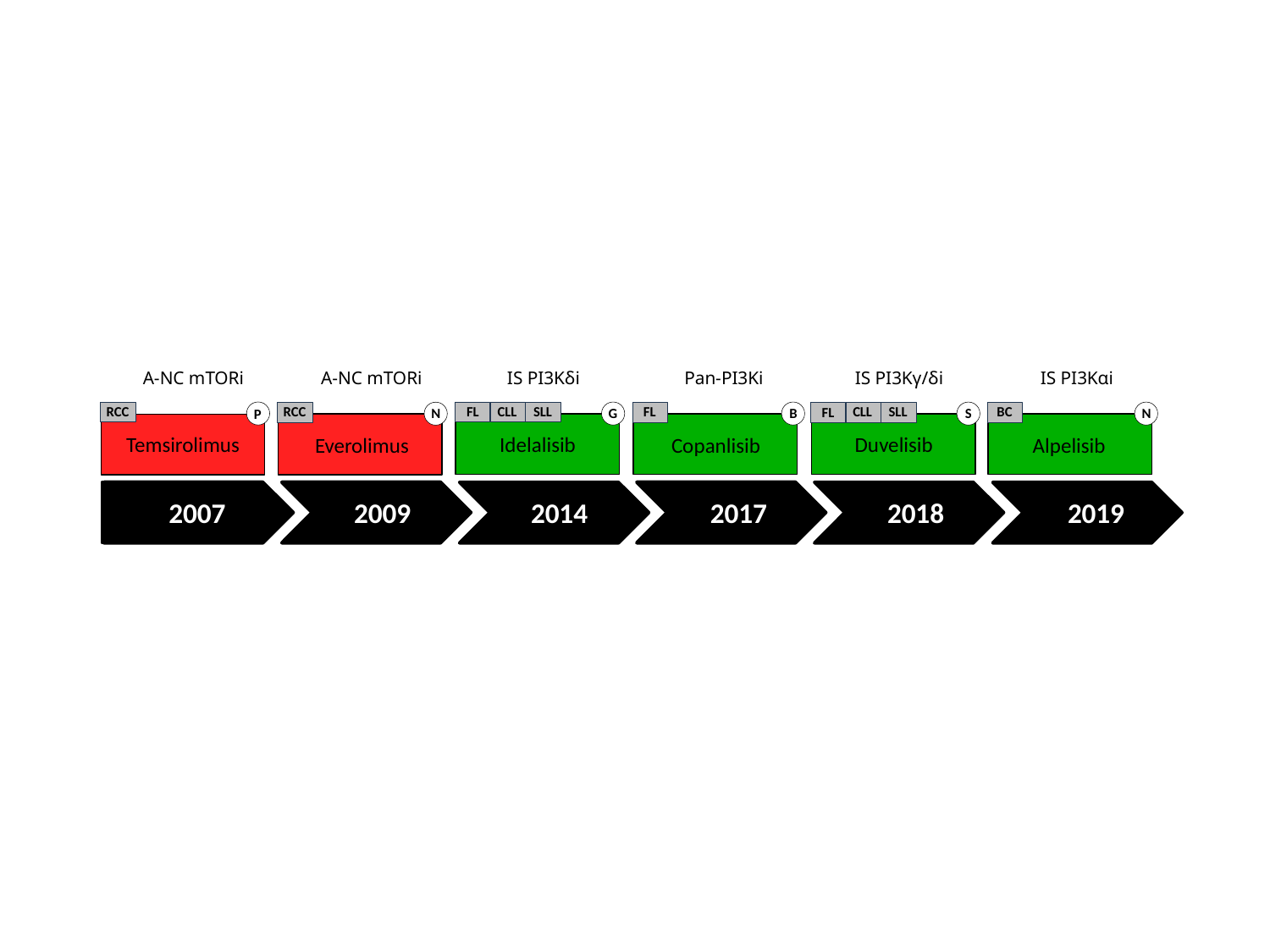

Pan-PI3Ki
IS PI3Kδi
IS PI3Kγ/δi
IS PI3Kαi
A-NC mTORi
A-NC mTORi
CLL
SLL
FL
RCC
SLL
CLL
RCC
FL
BC
FL
B
G
N
S
N
P
Idelalisib
Temsirolimus
Duvelisib
Everolimus
Alpelisib
Copanlisib
2007
2009
2017
2014
2018
2019
