# Supplementary material for: PI3K/AKT/mTOR signaling transduction pathway and targeted therapies in cancer
Source: Mol Cancer. 2023 Aug 18;22:138. doi: 10.1186/s12943-023-01827-6 (PMC10436543; doi:10.1186/s12943-023-01827-6)
Supplement: Supplementary file 2 — Additional file 2: Supplementary information 1. [file 12943_2023_1827_MOESM2_ESM.docx]

**SUPPLEMENTARY INFORMATION 1**

**THE PAM PATHWAY IN CANCER**

**RECEPTORS**

***RTK activation in normal cells***

The PI3K/AKT/mTORC (PAM) pathway is mainly induced by growth factors through their RTKs and GPCRs. Extracellular stimuli activate PI3K leading to subsequent increase of AKT kinase activity in the cell (1). Thus, PI3K, as well as its lipid products are normally rate-limiting factors specifically for the activation of AKT. Induction of RTKs (2) or GPCRs (3) usually initiates the canonical pathway that engenders the activation of AKT, resulting in plasma membrane localization and induction of one, or more isoforms of the class I PI3K family (4) (**FIGURE 1a**).

***RTK overactivation in cancer cells***

*Epidermal growth factor receptor (EGFR)/ERBB family*

The most widely studied *EGFR* alterations in lung adenocarcinoma is the deletion of exon 19 (dels746-750) or the substitution of arginine at codon 858 of exon 21 for leucine (L858R). These two mutations occur in the ATP-binding domain of tyrosine kinases, thereby increasing EGFR kinase activity (5) (6) (7) (8). Dysregulated EGFR activity can result in the hyperactivation of downstream pro-oncogenic signaling cascades such as the PAM and Ras/Raf/MEK/ERK pathways. Consequently, these downstream molecules may activate expression of several biologic factors leading to the proliferation of cancer cells (9) (10) (11). Additionally, *HER2* amplification has been detected in breast (12), gastric, gastroesophageal, pancreatic, and bladder cancer (13). The main aberration of *HER2* is gene amplification, which results in up to 37% protein overexpression manifesting as a driver mutation in breast cancer (12). Also, HER3 overexpression endorses cancer advancement by enhancing metastatic potential, thereby leading to treatment inefficacy in several human tumors (14). Indeed, HER3 promotes tumor progression by interacting with different RTKs, and makes a significant contribution to treatment failure mainly through the activation of PAM, MAPK/ERK and JAK/STAT pathways (15).

*Fibroblast growth factor receptor (FGFR) family*

Several studies have demonstrated genetic changes of *FGFR* in human tumors, including activating mutations, gene amplification, and chromosomal translocations. In fact, gene amplification and protein overexpression of fibroblast growth factor receptor 1 (FGFR1) have been observed in breast, lung (16), and gastric cancer (17). FGFR somatic activating mutations, especially in fibroblast growth factor receptor 2 (*FGFR2*) and fibroblast growth factor receptor 3 (*FGFR3*), are normally detected in numerous cancers such as gastric, NSCLC, endometrial, urothelial, and ovarian (18).

*Platelet-derived growth factor receptor (PDGFR) family*

PDGFR stimulates the PAM pathway, as well as the MAPK pathway (19). PDGFR family gene mutations, especially *PDGFRα* and *c-KIT* mutations, are quite common in gastrointestinal stromal tumors (GISTs) (20). In fact, *c-KIT* activating mutations are observed in almost all sporadic GISTs, usually in exon 11, between L550 and R588, coinciding to the juxtamembrane domain (21).

*Vascular endothelial growth factor receptor (VEGFR) family*

Vascular endothelial growth factor (VEGF) is the main regulator of tumor angiogenesis through endothelial cell proliferation and vascular permeability (22) (23) (24) (25). Differently to other RTKs that normally act through PAM pathway or Ras pathway, VEGF-VEGFR acts through PAM pathway (26) or PLCγ/PKC/MAPK pathway (27). VEGF forms VEGF/VEGFR dimers, which induce the PAM axis to mediate cancer survival, metastasis and angiogenesis (26). VEGF is normally overexpressed in several human tumors, including breast, renal, and colon cancer. Besides, the prognosis of tumor patients with elevated VEGF expression is poor (28).

*Hepatocyte growth factor (HGF) family*

HGF, which binds to proto-oncogenic *c-Met* receptor, can exert regulation of cell growth and morphogenesis through the activation of a tyrosine kinase signaling cascade (29). Moreover, HGF can stimulate mitogenesis and matrix invasion, playing a crucial role in carcinogenesis and angiogenesis (30). Since HGF can interact with hepatocyte growth factor receptor (HGFR), the protein product of *c-Met* oncogene (31), concurrent induction of HGFR through the overexpression of the HGF ligand and HGFR can contribute to oncogenesis (32).

*Leukocyte receptor tyrosine kinase (LTK) family*

*ALK* gene is mutated or amplified in neuroblastoma and glioblastoma (33), and is fused to *EML4* on chromosome 2 in non-small-cell lung cancer (NSCLC) (34). Consequently, the *EML4*/*ALK* fusion gene has become the most important research hotspot in NSCLC discovery. Indeed, current therapeutic applications of crizotinib in NSCLC patients displaying *EML4*/*ALK* fusion has reached remarkable curative effects over the last years, although drug resistance is a critical factor that obstructs this targeted therapy (35).

*Insulin receptor (InsR) family*

InsR and insulin-like growth factor 1 receptor (IGF1R) are members of the insulin receptor family class II (36). Ligand binding to InsR causes auto-phosphorylation at the tyrosine residues, which then follows the PAM pathway, as well as the Ras/Raf/MEK/ERK pathway (37). Generally, IGF1R is necessary for tumorigenesis, and minor insulin-like growth factor (IGF) activity safeguards against the onset of neoplasia. Thus, augmented IGF1R signaling is associated with high risk of cancer and aggressiveness (36). Both IGF1R or InsR can bind to insulin receptor substrate 1 (IRS1) through the phosphotyrosine-binding domain, thereby phosphorylating numerous sites on tyrosine in IRS1, resulting in the cooperation with different signaling molecules, including PI3K, GRB2, and SHP2, whose dysregulation can be implicated in the onset of cancer (38) (**FIGURE 1b**).

**PI3K**

***PI3K activation in normal cells***

Among the numerous types of PI3K, only class I can exert lipid phosphorylation following growth stimulation (39). PI3K (class I) is a heterodimer comprising two distinct subunits: the regulatory subunit p85, and the catalytic subunit p110 (40) (41). Specifically, the regulatory subunit p85 of PI3K can bind to phosphorylated tyrosine residues on the activated receptor through its Src homology 2 (SH2) domain. Subsequently, PI3K catalytic subunit p110 can form a complete active PI3K enzyme (42). Moreover, PI3K catalytic subunit p110 is also regulated by p85-independent mechanisms. Grb2 is also capable of binding to Ras-GEF SOS1 to activate Ras. Consequently, Ras-GTP binds the PI3K p110 subunit. Therefore, PI3K interconnection with members of the Ras subfamily GTPases is also important for its own regulation (43). Besides, PI3K can also be activated by GPCR by directly binding to the PI3K G-protein βγ dimer or Ras (44). Importantly, activation of PI3K can promote the addition of phosphates to the 3'-OH position of the inositol ring of phosphatidylinositol, generating three products of lipids, as follows: Phosphatidylinositol (PI) → PI 3-phosphate [PI(3)P or PIP] → PI 3,4-bisphosphate [PI(3,4)P2 or PIP2] → PI 3,4,5-triphosphate [PI(3,4,5)P3 or PIP3]. In the PAM pathway, following PI3K activation, class I PI3K phosphorylates PIP2, thereby producing PIP3 (4) (**FIGURE 1a**).

**PTEN**

***PTEN function in normal cells***

PI3K/PIP3 signal termination is mainly attained by tumor suppressor PTEN, which exerts dephosphorylation on PIP3, thereby switching it back to PIP2. This signal termination is generally accomplished by the activity of PTEN in combination with transient inactivation of PI3K (45) (46) (47). Dimeric PTEN complexes exert a stronger activity compared to PTEN monomers in PIP3 dephosphorylation, and consequently, in PI3K signaling control (48). Thus, by dephosphorylating PIP3, PTEN acts as an essential negative regulator of the PAM pathway affecting cell growth survival, whereas loss of *PTEN* results in the sustained output of these intracellular signalings (49) (**FIGURE 1a**).

**AKT**

***(PI3K-dependent) AKT activation in normal cells***

Phosphorylated phosphatidylinositol lipids on the inner face of plasma membrane can directly bind intracellular proteins which contain PH or FYVE zinc finger domains. Indeed, PIP3 binds AKT and PDK1, and as a result, they can accumulate near the membrane (50). Triggering PI3K leads to phosphorylation of two major residues on isomer AKT1 T308, present in the activation T-loop that is based in the catalytic kinase core, and S473, located in the C-terminal hydrophobic motif. Importantly, both aforementioned phosphorylations are necessary for full activation of the kinase (51). Particularly, these phosphorylation events correspond to different residues in isomer AKT2 (occurring in T309 and S474) and isomer AKT3 (occurring in T305 and S472) (52). Primarily, this localization of AKT and PDK1 to membrane sites of PIP3 or PI3,4P2 synthesis determine conformational changes enabling PDK1 to access residue T308 on AKT for phosphorylation. Indeed, the PH domain of AKT is suppressive in its inactive conformation, and this so-called “PH-in-conformation” is loosened by the binding of the PH domain with PI3K products, leading to a “PH-out-conformation”, which discharges the kinase domain and permits its phosphorylation by PDK1 (53). The primary AKT S473 kinase is mTORC2 (54). Moreover, the PH domain in the SIN1 module of mTORC2 binds to PIP3, thereby reducing the self-inhibition of mTOR kinase activity in the complex (55). Notably, in response to DNA damage, mTOR-related kinase DNA-PK replaces mTORC2 activity in order to exert phosphorylation on AKT S473; however, the mechanism of this regulation has not been elucidated (56). Once activated, AKT migrates from plasma membrane to cytoplasm and nucleus, where many substrates are located (57) (58) (59). Even though AKT is the most extensively known effector of PAM pathway, there are several other downstream effectors that are committed to the successive cellular response (4). Importantly, blockage of AKT function is determined by two protein phosphatases, named as PP2A and PHLPP. PP2A exerts dephosphorylation on AKT T308, thereby leading to the inhibition of the kinase (60). Remarkably, PHLPPs such as PHLPP1 and PHLPP2, dephosphorylate the same residue S473 on specific AKT isoforms (61) (**FIGURE 1a**).

***(PI3K-independent) AKT activation in normal cells***

Activation of AKT can also occur in the absence of PI3K, which is commonly referred as PI3K-independent AKT activation (62). Indeed, other tyrosine or serine/threonine kinases are involved in directly activating AKT in response to growth factors, DNA damage, or inflammation (63). Alternative activations of AKT can also occur with different mechanism of actions via ACK1 (63), IKKε (64), Src (65), PTK6 (66), DNA-PK (67), and TBK1 (68).

***AKT overactivation in cancer cells***

Notably, AKT1 amplification is the main mechanism for acquired resistance to cisplatin in human lung carcinoma A549 cells (69). Besides, mutations of AKT1 and AKT2 are frequently detected in small-cell lung cancer (SCLC) and NSCLC (70). Higher AKT1 and AKT2 activity has been frequently detected in several ovarian cancers too (71). Likewise, an increase in AKT activity has also been observed in human papillomavirus (HPV)-head and neck squamous cell cancer (HNSCC) cell lines (72). It has been reported that AKT induction can be enhanced during melanoma onset and advancement. Moreover, AKT3 expression, but not AKT1 and AKT2 expression, has been related to the formation of sporadic melanoma, especially when combined with PTEN loss (73). Cross-regulation between AKT pathway and BRAF pathway has been detected in melanoma. In fact, BRAF is frequently mutated in melanomas, and AKT1 Q79K mutations have been found in cases of acquired resistance to BRAF inhibitors (74). Increased p-AKT is detected in most human specimens of high-grade prostatic intraepithelial neoplasia and in invasive carcinomas (75). Genetic alterations of AKT1 and AKT2 have also been detected in colorectal (76), hepatocellular (77), and renal cancer (78); whereas only AKT1 aberration has been observed in testicular germ cell tumors (79). Loss of PH domain and PHLPP activity can result in hyperphosphorylation of AKT, and therefore, PHLPP expression is usually decreased or lost in numerous tumors (80). Indeed, PHLPP level is remarkably decreased in human pancreatic ductal adenocarcinoma with high level of AKT phosphorylation (81). Moreover, PHLPP1- and PHLPP2-mediated dephosphorylation can cease AKT signalling, leading to suppression of tumour growth (61) (**FIGURE 1b**).

***AKT protein targets***

AKT phosphorylation of downstream substrates determines the regulation of distinct cellular functions. AKT exerts a direct phosphorylation of diverse functional classes of protein targets, such as protein kinases, lipid kinases, cell cycle regulators, small GTPase regulators, metabolic enzymes, transcription factors, E3 ubiquitin ligases, and several others (82). Notably, AKT-mediated phosphorylation of these targets occurs on Ser/Thr residues to inhibit, or less often, activate the target protein function (62). Substrates preferred by AKT consist of a specific minimal consensus recognition sequence identified as R∙X∙R∙X∙X∙[Ser/Thr]∙Hyd; where X refers to any amino acid, and Hyd indicates a propensity for lengthy hydrophobic amino acid residues, albeit other factors (including subcellular localization and 3-D structure) may also be relevant (83) (84). Three major downstream targets of AKT are GSK3, FOXO, and mTORC1, which represent crucial signaling intersections that connect AKT with further regulatory circuits of the cell. However, other proteins such as apoptosis inducer BAD, cell cycle inhibitors p21 and p27, p53 inhibitor mdm2, key signal transducer in the network of genome integrity checkpoints Chk1, and pro-survival NF-κB inducer IKKα, are also important AKT substrates. Additionally, ASK1, MERIT40, Raf1, TBC1D4, PRAS40, ACLY, PFKFB2, PDE3B, eNOS, AMPK, WNK1, PIKfyve, Palladin, and Huntingtin are also regulated through AKT phosphorylation (85) (**FIGURE 2a**). Normally, most of AKT substrates are subjected to phosphorylation and regulation by all AKT isoforms, known as AKT1, AKT2, and AKT3. Notably, AKT1 inhibits migration as well as metastasis, AKT2 endorses metastatic dissemination in breast cancer (86), and AKT3 overexpression is associated with high expression of epithelial-mesenchymal transition (EMT) genes in colorectal cancer (CRC) (87). Interestingly, distinctive molecular characteristics of each isoform, their pertinent expression level in a specific setting, and their definite subcellular localization, are considered some of the mechanisms contributing to AKT isoform-specificity for downstream substrates (88). Besides, *AKT1*, or *AKT2*, or *AKT3* oncogenic activating somatic mutations can distinctively affect the arrangement of diverse cell signalling, and specific substrate phosphorylation (89) (**FIGURE 2b**).

***AKT-mediated GSK3 inhibition***

The multi-functional Ser/Thr protein kinase GSK3 can exist in two different isoforms, GSK3α and GSK3β, whose sequence homology is approximately 85%. The function of these isoforms is redundant in some contexts, and isoform-specific in certain tissues (90). GSK3 is present in different signal transduction pathways in cells, among which, the most prominent is the Wnt/β-catenin pathway. AKT-mediated GSK3 inhibition is determined by AKT phosphorylation on GSK3 NH_2_-terminus, forming an intramolecular pseudo-substrate that obstructs the phosphate-binding pocket, and consequently, suppresses substrate availability to GSK3 (91). In normal cells, GSK3 regulates numerous downstream targets with diverse functions, which are generally suppressed by GSK3-induced phosphorylation (90). AKT actively regulates targets in the growth factor signalling pathway via GSK3 inhibition. Diverse signaling transduction pathways prime each GSK3 substrate, which undoubtedly deepen the complexity of the regulation of GSK3 targets through various signal inputs. Phosphorylation of GSK3 targets related to regulation of cell survival or proliferation is identified by specific E3 ubiquitin ligases that ultimately exert targeting of the substrate for subsequent proteasomal degradation. E3 ubiquitin ligases act on anti-apoptotic and pro-survival MCL-1 protein, primed for GSK3 recognition following JNK-mediated phosphorylation (92), and transcription factor c-Myc, primed for GSK3 recognition following ERK-induced phosphorylation (93). Therefore, the signaling network of AKT is important since it stabilizes these target substrate proteins by inhibiting GSK3. Moreover, in normal cells GSK3 can control the metabolic status of the cell, either through direct phosphorylation and suppression of metabolic enzymes, e.g. glycogen synthase (94), or indirect inhibition of specific transcription factors such as SREBP1c, HIF1α, and NRF2 (90) (**FIGURE 3a**). GSK3 can phosphorylate other important proteins in the WNT/β-catenin multi-protein complex, including AXIN, APC, LPR5, and LPR6, whose function is implicated in EMT, which is essential for normal, as well as cancerous growth. In cancer, GSK3 function can differ depending on genetic mutations and tumor type. Mutations in *AXIN* can also affect the GSK3 phosphorylation sites, leading to abnormal phosphorylation and inactivation. In fact, if GSK-3 in unable to inactivate β-catenin, thereby indirectly increasing the activity of the latter, enhanced cell proliferation and chemoresistance can occur (95). Furthermore, GSK3 can also regulate NF-κB activity through phosphorylation and stabilization of NEMO, which cooperates with IKK, and is crucial for the activity of NF-κB. Notably, point mutation-mediated destabilization, and proteasomal degradation of NEMO can occur, resulting in reduced NF-κB activity. When GSK3 is inactive, there is a reduced NF-κB activity, and consequently, NF-κB is incapable of transcribing numerous metastasis- and inflammation-related genes frequently dysregulated in tumors (96) (97) (98) (99) (100). Consequently, proliferation and invasion of cancer cells without GSK3 and NF-κB activity may not be possible (**FIGURE 3b**).

***AKT-induced FOXO regulation***

Several gene targets mainly involved in response to different insulin and insulin-like growth factor 1 (IGF1) signaling are regulated by FOXO transcription factor family, consisting of FOXO1, FOXO3, FOXO4, and FOXO6 proteins (101). FOXOs are precisely regulated to ensure that transcription of specific target genes is responsive to environmental conditions. In normal cells, under oxidative stess (102), FOXO ability to restrain cell proliferation by endorsing quiescence and/or apoptosis plays an important role in numerous cellular processes including tumor suppression. The non-phosphorylated active forms of FOXO are located in the nucleus, and are involved in promoting mitochondria-dependent apoptosis by activating the expression of Bcl-2 family members. A primary type of regulation is AKT-induced phosphorylation of FOXO in response to insulin or growth factors. AKT signals regulate nucleus-to-cytoplasm FOXO shuttling to exert a transcriptional control of cellular proliferation and survival. In fact, AKT activation induces FOXO translocation from nucleus to cytoplasm, which consequently weakens its role in transcription (103). AKT exerts this regulation by phosphorylating three conserved residues on FOXO protein. AKT phosphorylation on FOXO, which occurs at the most amino-terminal site in proximity of DNA-binding domain, and at an extra site included in a nuclear localization sequence on FOXO, determines recognition motifs for the 14-3-3 family of phospho-binding adapter protein, allowing FOXO sequestration, 14-3-3/FOXO complex formation, and ultimately FOXO shuttling from nucleus to cytoplasm, thereby reducing the expression of FOXO gene targets. FOXO is an important downstream target of AKT for numerous critical cellular processes. Indeed, FOXO-targeted genes are associated with activation of apoptosis (Bcl-2 family members such as BIM, Bcl-xL, and PUMA), cell cycle blockage (p21 and p27), growth inhibition (BNIP3, MAP1LC3B, and Sestrin3), and tissue-specific metabolic changes (G6PC and PEPCK) (104). Besides, FOXO can also stimulate mitochondria-independent apoptosis by inducing death receptor ligands such as FasL and TRAIL (105) (**FIGURE 4a**).

***AKT-mediated regulation of mTORC1 and TSC2***

Cell growth is mainly regulated through AKT-mediated activation of the protein kinase mTORC1 (106). Activation of mTORC1 occurs through nutrient- and AKT-induced inhibitory phosphorylation of TSC2 (107), which acts in a molecular complex (known as the TSC complex) that also incorporates TSC1 and TBC1D7. Through its carboxyl-terminal domain, TSC2 functions as a GAP specific for Ras-related GTPase Rheb, thereby converting Rheb-GTP into Rheb-GDP. Thus, Rheb in its GTP-bound structure is considered a crucial mTORC1 activator. Conversely, the TSC complex through its Rheb-GAP activity can potently suppresses mTORC1. Nevertheless, AKT can diminish this suppression by phosphorylating TSC2, thereby activating mTORC1 (106). Besides, activation of mTORC1 is also controlled by Rags, a second class of small GTPases which mediate a separate input to mTORC1 via amino acid sensing (108). A heterodimer of Rag isoform localizes onto the lysosome membrane through a cooperation with Ragulator, a specific molecular complex, which interacts with lysosomal V-ATPase. Importantly, amino acids alter the guanine nucleotide-binding state of Rag, which influence the capability of Rag to interact with mTORC1. Notably, Rag proteins function in a complex of obligate heterodimers of redundant small GTPases consisiting of either RagA or RagB and RagC or RagD. These Rag proteins are recognised in an “on” active form when RagA/RagB is in the GTP-bound state (RagA/B ^GTP^) and RagC/RagD is in the GDP-bound state (RagC/D ^GDP^); whereas they are in an “off” inactive form when RagA/RagB is in the GDP-bound state (RagA/B ^GDP^) and RagC/RagD is in the GTP-bound state (RagC/D ^GTP^) (109). Indeed, when cellular amino acids are present, Rag proteins can recruit mTORC1 onto the surface of lysosome, the normal location of Rheb. In the absence of growth factors, the TSC complex interacts with Rheb on the lysosome membrane, thereby keeping it in the GDP-bound state, incapable of activating mTORC1 (110). Induction of growth factors promptly releases the TSC complex from Rheb at this location; this reaction depends on AKT and its five phosphorylation sites located on TSC2. Consequently, release of TSC complex from Rheb permits Rheb to turn into GTP loaded, therefore inducing mTORC1 recruited by Rag proteins. Notably, these distinct signals assure that mTORC1 is strongly activated only if cellular amino acids are recognized upstream the Rag proteins, and exogenous growth factor signals are produced via AKT and the TSC complex (111). The complex of mTOR enzyme with Raptor and other proteins, named mTORC1, can directly phosphorylate p70 ribosomal protein S6K kinase triggering negative PAM feedback (62). Indeed, a second enzyme complex, mTORC2, which contains mTOR and rictor, is phosphorylated, and thus, negatively regulated by S6K, thereby exerting a rate-limiting inhibitory effect on AKT (112). Thus, AKT is able to connect growth factor signals to a crucial signaling intersection that controls the metabolic changes underlying cell growth through its TSC/Rheb/mTORC1 circuit regulation (106). In fact, activation mTORC1 produces several anabolic processes, including protein, nucleotide, and lipid synthesis, whilst concurrently reducing the cellular catabolic process of autophagy. Therefore, it can be emphasised that mTORC1 exerts a dual role: a promoting downstream effector of PAM signaling pathway, and an inhibiting regulator with remarkable negative feedback effects on the induction of AKT by cell surface receptors (RTKs or GPCRs) (113) (**FIGURE 5a**).

**PAM SIGNALING NETWORK**

***Feedback mechanisms***

The PAM pathway is regulated by negative and positive feedback to ensure that stimulation signal transductions are captured and delivered transiently. In the negative feedback loop, numerous molecules downstream AKT cause an abrogating effect towards AKT activation, thereby regulating the pathway activity with a cyclical pattern. The induction of mTORC1 determines serine phosphorylation and degradation of IRS1/2 exerted by mTORC1 itself, or S6K (114), or other kinases, thereupon impeding the triggering of PI3K (115). Therefore, mTORC1 inhibition enhances IRS1 and insulin receptor substrate 2 (IRS2) stability, endorsing a more efficient signaling from insulin/IGF1 to PI3K/AKT. Besides, GRB10, another adaptor protein which negatively regulates RTK signalling network, is a direct target of mTORC1 (116). Thus, mTORC1 phosphorylates, stabilizes, and augments GRB10 efficienctly to diminish signal transduction from insulin/IGF1Rs and IRS1/2, consequenlty attenuating PAM pathway activation. Moreover, crosstalk between mTORC1 and mTORC2 interfere with AKT activation. Indeed, S6K1-induced phosphorylation of mTORC2 components Sin1 (T86, T398) and Rictor (T1135) reduces mTORC2-mediated phosphorylation on AKT (S473) (117). Apart from signaling of insulin/IGF1, inhibition of mTOR also augments the stimulation of other RTK types, including EGFR family members, such as EGFR, ErbB2 (HER2), ErbB3 (HER3), and ErbB4 (HER4) (118). Besides, suppression of AKT signaling can increase expression of insulin, IGF1Rs, and ErbB3 (HER3) through FOXO-induced transcriptional activation, thereby enhancing cell susceptibility and response to consecutive growth factor-induced triggering of PAM signaling (119). Conversely, via positive feedback, PI3K and AKT can regulate PTEN levels by decreasing its transcription. In addition, AKT phosphorylates and activates IKKα, resulting in the activation of NF-κB, a protein complex that controls transcription of PPARβ/δ agonists and TNFα, which ultimately suppress PTEN expression (120). E3 ligase NEDD4-1, which is upregulated by the PAM pathway, recognises PTEN for degradation. Hence, following AKT activation, PTEN is subjected to further inhibition in a positive feedback loop (121) (**FIGURE 6a**).

***Major PAM pathway cross-regulation with Ras/ERK and Wnt/GSK3/β-catenin pathways***

*Major PAM pathway cross-regulation with Ras/ERK pathway*

The PAM pathway frequently cross-regulates other major signaling axes, particularly Ras/ERK (122) pathway and Wnt/GSK3/β-catenin pathway (123). In cross-regulation between PAM pathway and Ras/ERK pathway, suppressors of one signaling axis can often stimulate the other (124). Indeed, AKT can phosphorylate B-Raf on S364, and a corresponding site of c-Raf on S259, leading to RAF inhibition (125). Besides, induction of ERK can inhibit RTK-induced activation of PAM signaling through phosphorylation of the scaffolding adaptors GAB1 and GAB2. Interestingly, PAM pathway and Ras/ERK pathway also engage in convergent regulation of several common downstream molecules, such as GSK3, FOXO, TSC2, and other cellular mechanisms (124). In fact, TSC2 inhibition through ERK- and RSK-mediated phosphorylation (126), as well as mTORC1 activation through ERK phosphorylation (127), and RSK phosphorylation (128), represents a convergence to regulate the control of cap-dependent translation (129). Moreover, ERK signaling induces kinases MNK1 and MNK2, which exert phosphorylation of the mRNA 5' cap-binding protein eIF4E, thereby promoting its ability to initiate cap-dependent translation (130). This ERK regulation occurs downstream from mTORC1-induced phosphorylation of 4E-BP proteins, which triggers their release from inhibitory binding of eIF4E (**FIGURE 7a**). Further studies have described the cross-regulation between PAM pathway and Ras/ERK pathway in cancer. Inhibition of mTORC1 with everolimus in cancer cells results in the activation of Ras/ERK pathway, which depends on an S6K-PI3K signaling network. Suppression of the Ras/ERK pathway increases the anticancer effect of mTORC1 inhibition by everolimus in tumor cells and in a xenograft mouse model (131). Consequently, PAM pathway and Ras/ERK pathway can cross-inhibit each other, and simultaneously, can also act cooperatively to properly regulate key cellular processes related to cell proliferation and growth, whose dysregulation can lead to tumor onset and/or progression (132). Mutation and overexpression of *HRAS* decreases vulnerability to PI3K inhibitors, whereas knockdown improves susceptibility (133). In addition, tumor cell proliferation and PI3K inhibitor resistance can also be mediated by reciprocal actions between MAP2K4 and NEK9 (134). Interestingly, PAM pathway and the Ras/ERK pathway converge on the BH3 family of proteins, known to exert regulation of apoptosis (38). PAM and Ras/ERK signaling, which are often abnormally activated in numerous human cancers, have been co-targeted for cancer therapy with limited benefits due to this complex crosstalk and significant toxicity to normal tissues (135). Thus, novel strategies such as PAM + Ras/ERK dual inhibitors with reduced side effects may be considered in the future for developing anti-cancer therapies (**FIGURE 7b**).

*Major PAM pathway cross-regulation with Wnt/GSK3/β-catenin pathway*

In cross-regulation between PAM pathway and Wnt/GSK3/β-catenin pathway (136) AKT can inhibit GSK3 by phosphorylation on GSK3 amino-terminus (137), thereby indirectly exerting activation of downstream transcription factor β-catenin (91) (**FIGURE 7a**). More studies have focussed on the cross-regulation between PAM pathway and Wnt/GSK3/β-catenin pathway in cancer. Chemotherapeutic-induced inhibition of mTOR also inhibits β-catenin signaling in osteosarcoma (138). Besides, treatment with rapamycin also inhibits growth of MMTV-Wnt-1 mammary cancers through reduction of mTOR (139). Importantly, PTEN can also exert a direct inhibition of β-catenin nuclear translocation, by enhancing phosphorylation, and thus, proteasomal degradation of β-catenin (140). PTEN expression also decreases β-catenin-induced enhancement of androgen receptor transactivation in prostate tumor (141). Notably, altered expression of GSK3 influences the advancement and evolution of cancer through dysregulation of apoptosis, cell cycle, senescence, and therapy resistance (95). Indeed, the GSK3-associated axis EGFR/PI3K/AKT/GSK3/mTORC1 is often involved in cancer, since it can be remarkably activated due to mutations in important constituent genes (142). The synergism related to dysregulation of PAM pathway and Wnt/GSK3/β-catenin pathway in the development and progression of granulosa cell tumor and testicular cancer has also been reported (143). Thus, PAM and Wnt/GSK3/β-catenin signaling can mainly crossregulate through PTEN, GSK3, and/or AKT in numerous different tumor types (144). Additionally, IGF1 has been found to activate the Wnt/GSK3/β-catenin pathway through mediation of PAM pathway in melanoma cells (145). Furthermore, IL-1β can inactivate GSK3β via AKT, therefore inducing the Wnt/GSK3/β-catenin cascade in colon cancer cells (146). Finally, galectin-3, which has been correlated with metastasis in colon cancer, worsens cancer progression by regulating AKT and GSK3 (147). Thus, it is reasonable to develop therapeutics that can target both the PAM pathway and the Wnt/GSK3/β-catenin pathway simultaneously (**FIGURE 7b**).

**ABBREVIATIONS**

**CRC**: Colorectal cancer

**EGFR**: Epidermal growth factor receptor (ErbB-1) (HER1)

**EMT**: Epithelial-mesenchymal transition

**FGFR**: Fibroblast growth factor receptor

**FGFR1**: Fibroblast growth factor receptor 1

**FGFR2**: Fibroblast growth factor receptor 2

**FGFR3**: Fibroblast growth factor receptor 3

**GISTs**: Gastrointestinal stromal tumors

**HGF**: Hepatocyte growth factor

**HGFR**: Hepatocyte growth factor receptor

**HNSCC**: Head and neck squamous cell cancer

**HPV**: Human papillomavirus

**IGF**: Insulin-like growth factor

**IGF1**: Insulin-like growth factor 1

**IGF1R**: Insulin-like growth factor 1 receptor

**InsR**: Insulin receptor

**IRS**: Insulin receptor substrate

**IRS1**: Insulin receptor substrate 1

**IRS2**: Insulin receptor substrate 2

**LTK**: Leukocyte receptor tyrosine kinase

**NSCLC**: Non-small-cell lung cancer

**PAM**: PI3K/AKT/mTORC

**PDGFR**: Platelet-derived growth factor receptor

**SCLC**: Small-cell lung cancer

**VEGF**: Vascular endothelial growth factor

**VEGFR**: Vascular endothelial growth factor receptor

**REFERENCES**

1. Liu R, Chen Y, Liu G, Li C, Song Y, Cao Z, et al. PI3K/AKT pathway as a key link modulates the multidrug resistance of cancers. Cell Death Dis. 2020;11(9):797.

2. Ciardiello F, Tortora G. EGFR antagonists in cancer treatment. N Engl J Med. 2008;358(11):1160-74.

3. Law NC, White MF, Hunzicker-Dunn ME. G protein-coupled receptors (GPCRs) That Signal via Protein Kinase A (PKA) Cross-talk at Insulin Receptor Substrate 1 (IRS1) to Activate the phosphatidylinositol 3-kinase (PI3K)/AKT Pathway. J Biol Chem. 2016;291(53):27160-9.

4. Vanhaesebroeck B, Guillermet-Guibert J, Graupera M, Bilanges B. The emerging mechanisms of isoform-specific PI3K signalling. Nat Rev Mol Cell Biol. 2010;11(5):329-41.

5. Lynch TJ, Bell DW, Sordella R, Gurubhagavatula S, Okimoto RA, Brannigan BW, et al. Activating mutations in the epidermal growth factor receptor underlying responsiveness of non-small-cell lung cancer to gefitinib. N Engl J Med. 2004;350(21):2129-39.

6. Wang L, Syn NL, Subhash VV, Any Y, Thuya WL, Cheow ESH, et al. Pan-HDAC inhibition by panobinostat mediates chemosensitization to carboplatin in non-small cell lung cancer via attenuation of EGFR signaling. Cancer Lett. 2018;417:152-60.

7. Wang C, Kar S, Lai X, Cai W, Arfuso F, Sethi G, et al. Triple negative breast cancer in Asia: An insider's view. Cancer Treat Rev. 2018;62:29-38.

8. Sethi G, Ahn KS, Chaturvedi MM, Aggarwal BB. Epidermal growth factor (EGF) activates nuclear factor-kappaB through IkappaBalpha kinase-independent but EGF receptor-kinase dependent tyrosine 42 phosphorylation of IkappaBalpha. Oncogene. 2007;26(52):7324-32.

9. Wee P, Wang Z. Epidermal Growth Factor Receptor Cell Proliferation Signaling Pathways. Cancers (Basel). 2017;9(5).

10. Puar YR, Shanmugam MK, Fan L, Arfuso F, Sethi G, Tergaonkar V. Evidence for the Involvement of the Master Transcription Factor NF-κB in Cancer Initiation and Progression. Biomedicines. 2018;6(3).

11. Tewari D, Nabavi SF, Nabavi SM, Sureda A, Farooqi AA, Atanasov AG, et al. Targeting activator protein 1 signaling pathway by bioactive natural agents: Possible therapeutic strategy for cancer prevention and intervention. Pharmacol Res. 2018;128:366-75.

12. Verma S, Miles D, Gianni L, Krop IE, Welslau M, Baselga J, et al. Trastuzumab emtansine for HER2-positive advanced breast cancer. N Engl J Med. 2012;367(19):1783-91.

13. Apicella M, Corso S, Giordano S. Targeted therapies for gastric cancer: failures and hopes from clinical trials. Oncotarget. 2017;8(34):57654-69.

14. Lyu H, Han A, Polsdofer E, Liu S, Liu B. Understanding the biology of HER3 receptor as a therapeutic target in human cancer. Acta Pharm Sin B. 2018;8(4):503-10.

15. Mishra R, Patel H, Alanazi S, Yuan L, Garrett JT. HER3 signaling and targeted therapy in cancer. Oncol Rev. 2018;12(1):355.

16. Peifer M, Fernández-Cuesta L, Sos ML, George J, Seidel D, Kasper LH, et al. Integrative genome analyses identify key somatic driver mutations of small-cell lung cancer. Nat Genet. 2012;44(10):1104-10.

17. Matsumoto K, Arao T, Hamaguchi T, Shimada Y, Kato K, Oda I, et al. FGFR2 gene amplification and clinicopathological features in gastric cancer. Br J Cancer. 2012;106(4):727-32.

18. Babina IS, Turner NC. Advances and challenges in targeting FGFR signalling in cancer. Nat Rev Cancer. 2017;17(5):318-32.

19. Wu E, Palmer N, Tian Z, Moseman AP, Galdzicki M, Wang X, et al. Comprehensive dissection of PDGF-PDGFR signaling pathways in PDGFR genetically defined cells. PLoS One. 2008;3(11):e3794.

20. Hirota S, Isozaki K, Moriyama Y, Hashimoto K, Nishida T, Ishiguro S, et al. Gain-of-function mutations of c-kit in human gastrointestinal stromal tumors. Science. 1998;279(5350):577-80.

21. Heinrich MC, Corless CL, Duensing A, McGreevey L, Chen CJ, Joseph N, et al. PDGFRA activating mutations in gastrointestinal stromal tumors. Science. 2003;299(5607):708-10.

22. Senger DR, Galli SJ, Dvorak AM, Perruzzi CA, Harvey VS, Dvorak HF. Tumor cells secrete a vascular permeability factor that promotes accumulation of ascites fluid. Science. 1983;219(4587):983-5.

23. Loo SY, Syn NL, Koh AP, Teng JC, Deivasigamani A, Tan TZ, et al. Epigenetic derepression converts PPARγ into a druggable target in triple-negative and endocrine-resistant breast cancers. Cell Death Discov. 2021;7(1):265.

24. Kirtonia A, Sethi G, Garg M. The multifaceted role of reactive oxygen species in tumorigenesis. Cell Mol Life Sci. 2020;77(22):4459-83.

25. Lee JH, Kim C, Lee SG, Sethi G, Ahn KS. Ophiopogonin D, a Steroidal Glycoside Abrogates STAT3 Signaling Cascade and Exhibits Anti-Cancer Activity by Causing GSH/GSSG Imbalance in Lung Carcinoma. Cancers (Basel). 2018;10(11).

26. He Y, Sun MM, Zhang GG, Yang J, Chen KS, Xu WW, et al. Targeting PI3K/Akt signal transduction for cancer therapy. Signal Transduct Target Ther. 2021;6(1):425.

27. Shibuya M. Vascular Endothelial Growth Factor (VEGF) and Its Receptor (VEGFR) Signaling in Angiogenesis: A Crucial Target for Anti- and Pro-Angiogenic Therapies. Genes Cancer. 2011;2(12):1097-105.

28. Ferrara N. VEGF and the quest for tumour angiogenesis factors. Nat Rev Cancer. 2002;2(10):795-803.

29. Bottaro DP, Rubin JS, Faletto DL, Chan AM, Kmiecik TE, Vande Woude GF, et al. Identification of the hepatocyte growth factor receptor as the c-met proto-oncogene product. Science. 1991;251(4995):802-4.

30. Ding X, Ji J, Jiang J, Cai Q, Wang C, Shi M, et al. HGF-mediated crosstalk between cancer-associated fibroblasts and MET-unamplified gastric cancer cells activates coordinated tumorigenesis and metastasis. Cell Death Dis. 2018;9(9):867.

31. Naldini L, Weidner KM, Vigna E, Gaudino G, Bardelli A, Ponzetto C, et al. Scatter factor and hepatocyte growth factor are indistinguishable ligands for the MET receptor. EMBO J. 1991;10(10):2867-78.

32. Johnson M, Koukoulis G, Kochhar K, Kubo C, Nakamura T, Iyer A. Selective tumorigenesis in non-parenchymal liver epithelial cell lines by hepatocyte growth factor transfection. Cancer Lett. 1995;96(1):37-48.

33. Chen Y, Takita J, Choi YL, Kato M, Ohira M, Sanada M, et al. Oncogenic mutations of ALK kinase in neuroblastoma. Nature. 2008;455(7215):971-4.

34. Soda M, Choi YL, Enomoto M, Takada S, Yamashita Y, Ishikawa S, et al. Identification of the transforming EML4-ALK fusion gene in non-small-cell lung cancer. Nature. 2007;448(7153):561-6.

35. Rikova K, Guo A, Zeng Q, Possemato A, Yu J, Haack H, et al. Global survey of phosphotyrosine signaling identifies oncogenic kinases in lung cancer. Cell. 2007;131(6):1190-203.

36. Pollak M. The insulin and insulin-like growth factor receptor family in neoplasia: an update. Nat Rev Cancer. 2012;12(3):159-69.

37. Ward CW, Lawrence MC. Ligand-induced activation of the insulin receptor: a multi-step process involving structural changes in both the ligand and the receptor. Bioessays. 2009;31(4):422-34.

38. Engelman JA. Targeting PI3K signalling in cancer: opportunities, challenges and limitations. Nat Rev Cancer. 2009;9(8):550-62.

39. Zhao L, Vogt PK. Class I PI3K in oncogenic cellular transformation. Oncogene. 2008;27(41):5486-96.

40. Cantrell DA. Phosphoinositide 3-kinase signalling pathways. J Cell Sci. 2001;114(Pt 8):1439-45.

41. Kim C, Lee JH, Ko JH, Chinnathambi A, Alharbi SA, Shair OHM, et al. Formononetin Regulates Multiple Oncogenic Signaling Cascades and Enhances Sensitivity to Bortezomib in a Multiple Myeloma Mouse Model. Biomolecules. 2019;9(7).

42. Castellano E, Downward J. RAS Interaction with PI3K: More Than Just Another Effector Pathway. Genes Cancer. 2011;2(3):261-74.

43. Rodriguez-Viciana P, Warne PH, Dhand R, Vanhaesebroeck B, Gout I, Fry MJ, et al. Phosphatidylinositol-3-OH kinase as a direct target of Ras. Nature. 1994;370(6490):527-32.

44. New DC, Wong YH. Molecular mechanisms mediating the G protein-coupled receptor regulation of cell cycle progression. J Mol Signal. 2007;2:2.

45. Auger KR, Serunian LA, Soltoff SP, Libby P, Cantley LC. PDGF-dependent tyrosine phosphorylation stimulates production of novel polyphosphoinositides in intact cells. Cell. 1989;57(1):167-75.

46. Abadi AJ, Zarrabi A, Gholami MH, Mirzaei S, Hashemi F, Zabolian A, et al. Small in Size, but Large in Action: microRNAs as Potential Modulators of PTEN in Breast and Lung Cancers. Biomolecules. 2021;11(2).

47. Ashrafizadeh M, Najafi M, Ang HL, Moghadam ER, Mahabady MK, Zabolian A, et al. PTEN, a Barrier for Proliferation and Metastasis of Gastric Cancer Cells: From Molecular Pathways to Targeting and Regulation. Biomedicines. 2020;8(8).

48. Papa A, Wan L, Bonora M, Salmena L, Song MS, Hobbs RM, et al. Cancer-associated PTEN mutants act in a dominant-negative manner to suppress PTEN protein function. Cell. 2014;157(3):595-610.

49. Chow LM, Baker SJ. PTEN function in normal and neoplastic growth. Cancer Lett. 2006;241(2):184-96.

50. Cantley LC. The phosphoinositide 3-kinase pathway. Science. 2002;296(5573):1655-7.

51. Alessi DR, Andjelkovic M, Caudwell B, Cron P, Morrice N, Cohen P, et al. Mechanism of activation of protein kinase B by insulin and IGF-1. EMBO J. 1996;15(23):6541-51.

52. Alessi DR, James SR, Downes CP, Holmes AB, Gaffney PR, Reese CB, et al. Characterization of a 3-phosphoinositide-dependent protein kinase which phosphorylates and activates protein kinase Balpha. Curr Biol. 1997;7(4):261-9.

53. Calleja V, Laguerre M, Parker PJ, Larijani B. Role of a novel PH-kinase domain interface in PKB/Akt regulation: structural mechanism for allosteric inhibition. PLoS Biol. 2009;7(1):e17.

54. Sarbassov DD, Guertin DA, Ali SM, Sabatini DM. Phosphorylation and regulation of Akt/PKB by the rictor-mTOR complex. Science. 2005;307(5712):1098-101.

55. Liu P, Gan W, Chin YR, Ogura K, Guo J, Zhang J, et al. PtdIns(3,4,5)P3-Dependent Activation of the mTORC2 Kinase Complex. Cancer Discov. 2015;5(11):1194-209.

56. Bozulic L, Surucu B, Hynx D, Hemmings BA. PKBalpha/Akt1 acts downstream of DNA-PK in the DNA double-strand break response and promotes survival. Mol Cell. 2008;30(2):203-13.

57. Vanhaesebroeck B, Alessi DR. The PI3K-PDK1 connection: more than just a road to PKB. Biochem J. 2000;346 Pt 3:561-76.

58. Kim SW, Kim SM, Bae H, Nam D, Lee JH, Lee SG, et al. Embelin inhibits growth and induces apoptosis through the suppression of Akt/mTOR/S6K1 signaling cascades. Prostate. 2013;73(3):296-305.

59. Shanmugam MK, Ong TH, Kumar AP, Lun CK, Ho PC, Wong PT, et al. Ursolic acid inhibits the initiation, progression of prostate cancer and prolongs the survival of TRAMP mice by modulating pro-inflammatory pathways. PLoS One. 2012;7(3):e32476.

60. Andjelković M, Jakubowicz T, Cron P, Ming XF, Han JW, Hemmings BA. Activation and phosphorylation of a pleckstrin homology domain containing protein kinase (RAC-PK/PKB) promoted by serum and protein phosphatase inhibitors. Proc Natl Acad Sci U S A. 1996;93(12):5699-704.

61. Brognard J, Sierecki E, Gao T, Newton AC. PHLPP and a second isoform, PHLPP2, differentially attenuate the amplitude of Akt signaling by regulating distinct Akt isoforms. Mol Cell. 2007;25(6):917-31.

62. Manning BD, Toker A. AKT/PKB Signaling: Navigating the Network. Cell. 2017;169(3):381-405.

63. Mahajan K, Mahajan NP. Shepherding AKT and androgen receptor by Ack1 tyrosine kinase. J Cell Physiol. 2010;224(2):327-33.

64. Guo JP, Coppola D, Cheng JQ. IKBKE protein activates Akt independent of phosphatidylinositol 3-kinase/PDK1/mTORC2 and the pleckstrin homology domain to sustain malignant transformation. J Biol Chem. 2011;286(43):37389-98.

65. Jiang T, Qiu Y. Interaction between Src and a C-terminal proline-rich motif of Akt is required for Akt activation. J Biol Chem. 2003;278(18):15789-93.

66. Zheng Y, Peng M, Wang Z, Asara JM, Tyner AL. Protein tyrosine kinase 6 directly phosphorylates AKT and promotes AKT activation in response to epidermal growth factor. Mol Cell Biol. 2010;30(17):4280-92.

67. Toulany M, Rodemann HP. Potential of Akt mediated DNA repair in radioresistance of solid tumors overexpressing erbB-PI3K-Akt pathway. Transl Cancer Res. 2013;2(3):190-202.

68. Joung SM, Park ZY, Rani S, Takeuchi O, Akira S, Lee JY. Akt contributes to activation of the TRIF-dependent signaling pathways of TLRs by interacting with TANK-binding kinase 1. J Immunol. 2011;186(1):499-507.

69. Chun KH, Kosmeder JW, Sun S, Pezzuto JM, Lotan R, Hong WK, et al. Effects of deguelin on the phosphatidylinositol 3-kinase/Akt pathway and apoptosis in premalignant human bronchial epithelial cells. J Natl Cancer Inst. 2003;95(4):291-302.

70. Pérez-Ramírez C, Cañadas-Garre M, Molina M, Faus-Dáder MJ, Calleja-Hernández M. PTEN and PI3K/AKT in non-small-cell lung cancer. Pharmacogenomics. 2015;16(16):1843-62.

71. Bellacosa A, Testa JR, Moore R, Larue L. A portrait of AKT kinases: human cancer and animal models depict a family with strong individualities. Cancer Biol Ther. 2004;3(3):268-75.

72. Gupta AK, Lee JH, Wilke WW, Quon H, Smith G, Maity A, et al. Radiation response in two HPV-infected head-and-neck cancer cell lines in comparison to a non-HPV-infected cell line and relationship to signaling through AKT. Int J Radiat Oncol Biol Phys. 2009;74(3):928-33.

73. Stahl JM, Sharma A, Cheung M, Zimmerman M, Cheng JQ, Bosenberg MW, et al. Deregulated Akt3 activity promotes development of malignant melanoma. Cancer Res. 2004;64(19):7002-10.

74. McCubrey JA, Steelman LS, Abrams SL, Lee JT, Chang F, Bertrand FE, et al. Roles of the RAF/MEK/ERK and PI3K/PTEN/AKT pathways in malignant transformation and drug resistance. Adv Enzyme Regul. 2006;46:249-79.

75. Malik SN, Brattain M, Ghosh PM, Troyer DA, Prihoda T, Bedolla R, et al. Immunohistochemical demonstration of phospho-Akt in high Gleason grade prostate cancer. Clin Cancer Res. 2002;8(4):1168-71.

76. Jin J, Shi Y, Zhang S, Yang S. PIK3CA mutation and clinicopathological features of colorectal cancer: a systematic review and Meta-Analysis. Acta Oncol. 2020;59(1):66-74.

77. Kitagawa M, Liao PJ, Lee KH, Wong J, Shang SC, Minami N, et al. Dual blockade of the lipid kinase PIP4Ks and mitotic pathways leads to cancer-selective lethality. Nat Commun. 2017;8(1):2200.

78. Lin A, Piao HL, Zhuang L, Sarbassov dD, Ma L, Gan B. FoxO transcription factors promote AKT Ser473 phosphorylation and renal tumor growth in response to pharmacologic inhibition of the PI3K-AKT pathway. Cancer Res. 2014;74(6):1682-93.

79. Feldman DR, Iyer G, Van Alstine L, Patil S, Al-Ahmadie H, Reuter VE, et al. Presence of somatic mutations within PIK3CA, AKT, RAS, and FGFR3 but not BRAF in cisplatin-resistant germ cell tumors. Clin Cancer Res. 2014;20(14):3712-20.

80. Chen M, Pratt CP, Zeeman ME, Schultz N, Taylor BS, O'Neill A, et al. Identification of PHLPP1 as a tumor suppressor reveals the role of feedback activation in PTEN-mutant prostate cancer progression. Cancer Cell. 2011;20(2):173-86.

81. Nitsche C, Edderkaoui M, Moore RM, Eibl G, Kasahara N, Treger J, et al. The phosphatase PHLPP1 regulates Akt2, promotes pancreatic cancer cell death, and inhibits tumor formation. Gastroenterology. 2012;142(2):377-87.e1-5.

82. Agarwal AK. How to explain the AKT phosphorylation of downstream targets in the wake of recent findings. Proc Natl Acad Sci U S A. 2018;115(27):E6099-E100.

83. Nicholson KM, Anderson NG. The protein kinase B/Akt signalling pathway in human malignancy. Cell Signal. 2002;14(5):381-95.

84. Yudushkin I. Control of Akt activity and substrate phosphorylation in cells. IUBMB Life. 2020;72(6):1115-25.

85. Manning BD, Cantley LC. AKT/PKB signaling: navigating downstream. Cell. 2007;129(7):1261-74.

86. Dillon RL, Marcotte R, Hennessy BT, Woodgett JR, Mills GB, Muller WJ. Akt1 and akt2 play distinct roles in the initiation and metastatic phases of mammary tumor progression. Cancer Res. 2009;69(12):5057-64.

87. Buikhuisen JY, Gomez Barila PM, Torang A, Dekker D, de Jong JH, Cameron K, et al. AKT3 Expression in Mesenchymal Colorectal Cancer Cells Drives Growth and Is Associated with Epithelial-Mesenchymal Transition. Cancers (Basel). 2021;13(4).

88. Kim EK, Yun SJ, Ha JM, Kim YW, Jin IH, Yun J, et al. Selective activation of Akt1 by mammalian target of rapamycin complex 2 regulates cancer cell migration, invasion, and metastasis. Oncogene. 2011;30(26):2954-63.

89. Lien EC, Lyssiotis CA, Juvekar A, Hu H, Asara JM, Cantley LC, et al. Glutathione biosynthesis is a metabolic vulnerability in PI(3)K/Akt-driven breast cancer. Nat Cell Biol. 2016;18(5):572-8.

90. Kaidanovich-Beilin O, Woodgett JR. GSK-3: Functional Insights from Cell Biology and Animal Models. Front Mol Neurosci. 2011;4:40.

91. Hermida MA, Dinesh Kumar J, Leslie NR. GSK3 and its interactions with the PI3K/AKT/mTOR signalling network. Adv Biol Regul. 2017;65:5-15.

92. Morel C, Carlson SM, White FM, Davis RJ. Mcl-1 integrates the opposing actions of signaling pathways that mediate survival and apoptosis. Mol Cell Biol. 2009;29(14):3845-52.

93. Welcker M, Orian A, Jin J, Grim JE, Grim JA, Harper JW, et al. The Fbw7 tumor suppressor regulates glycogen synthase kinase 3 phosphorylation-dependent c-Myc protein degradation. Proc Natl Acad Sci U S A. 2004;101(24):9085-90.

94. Parker PJ, Caudwell FB, Cohen P. Glycogen synthase from rabbit skeletal muscle; effect of insulin on the state of phosphorylation of the seven phosphoserine residues in vivo. Eur J Biochem. 1983;130(1):227-34.

95. Duda P, Akula SM, Abrams SL, Steelman LS, Martelli AM, Cocco L, et al. Targeting GSK3 and Associated Signaling Pathways Involved in Cancer. Cells. 2020;9(5).

96. Götschel F, Kern C, Lang S, Sparna T, Markmann C, Schwager J, et al. Inhibition of GSK3 differentially modulates NF-kappaB, CREB, AP-1 and beta-catenin signaling in hepatocytes, but fails to promote TNF-alpha-induced apoptosis. Exp Cell Res. 2008;314(6):1351-66.

97. Mirzaei S, Zarrabi A, Hashemi F, Zabolian A, Saleki H, Ranjbar A, et al. Regulation of Nuclear Factor-KappaB (NF-κB) signaling pathway by non-coding RNAs in cancer: Inhibiting or promoting carcinogenesis? Cancer Lett. 2021;509:63-80.

98. Manu KA, Shanmugam MK, Ramachandran L, Li F, Siveen KS, Chinnathambi A, et al. Isorhamnetin augments the anti-tumor effect of capecitabine through the negative regulation of NF-κB signaling cascade in gastric cancer. Cancer Lett. 2015;363(1):28-36.

99. Ahn KS, Sethi G, Jain AK, Jaiswal AK, Aggarwal BB. Genetic deletion of NAD(P)H:quinone oxidoreductase 1 abrogates activation of nuclear factor-kappaB, IkappaBalpha kinase, c-Jun N-terminal kinase, Akt, p38, and p44/42 mitogen-activated protein kinases and potentiates apoptosis. J Biol Chem. 2006;281(29):19798-808.

100. Sawhney M, Rohatgi N, Kaur J, Shishodia S, Sethi G, Gupta SD, et al. Expression of NF-kappaB parallels COX-2 expression in oral precancer and cancer: association with smokeless tobacco. Int J Cancer. 2007;120(12):2545-56.

101. Jiang Y, Yan F, Feng Z, Lazarovici P, Zheng W. Signaling Network of Forkhead Family of Transcription Factors (FOXO) in Dietary Restriction. Cells. 2019;9(1).

102. Brunet A, Sweeney LB, Sturgill JF, Chua KF, Greer PL, Lin Y, et al. Stress-dependent regulation of FOXO transcription factors by the SIRT1 deacetylase. Science. 2004;303(5666):2011-5.

103. Brunet A, Bonni A, Zigmond MJ, Lin MZ, Juo P, Hu LS, et al. Akt promotes cell survival by phosphorylating and inhibiting a Forkhead transcription factor. Cell. 1999;96(6):857-68.

104. Webb AE, Brunet A. FOXO transcription factors: key regulators of cellular quality control. Trends Biochem Sci. 2014;39(4):159-69.

105. Fu Z, Tindall DJ. FOXOs, cancer and regulation of apoptosis. Oncogene. 2008;27(16):2312-9.

106. Saxton RA, Sabatini DM. mTOR Signaling in Growth, Metabolism, and Disease. Cell. 2017;168(6):960-76.

107. Inoki K, Li Y, Zhu T, Wu J, Guan KL. TSC2 is phosphorylated and inhibited by Akt and suppresses mTOR signalling. Nat Cell Biol. 2002;4(9):648-57.

108. Takahara T, Amemiya Y, Sugiyama R, Maki M, Shibata H. Amino acid-dependent control of mTORC1 signaling: a variety of regulatory modes. J Biomed Sci. 2020;27(1):87.

109. Brady OA, Diab HI, Puertollano R. Rags to riches: Amino acid sensing by the Rag GTPases in health and disease. Small GTPases. 2016;7(4):197-206.

110. Menon S, Dibble CC, Talbott G, Hoxhaj G, Valvezan AJ, Takahashi H, et al. Spatial control of the TSC complex integrates insulin and nutrient regulation of mTORC1 at the lysosome. Cell. 2014;156(4):771-85.

111. Sekulić A, Hudson CC, Homme JL, Yin P, Otterness DM, Karnitz LM, et al. A direct linkage between the phosphoinositide 3-kinase-AKT signaling pathway and the mammalian target of rapamycin in mitogen-stimulated and transformed cells. Cancer Res. 2000;60(13):3504-13.

112. Rosner M, Siegel N, Valli A, Fuchs C, Hengstschläger M. mTOR phosphorylated at S2448 binds to raptor and rictor. Amino Acids. 2010;38(1):223-8.

113. Xu F, Na L, Li Y, Chen L. Roles of the PI3K/AKT/mTOR signalling pathways in neurodegenerative diseases and tumours. Cell Biosci. 2020;10(1):54.

114. Shah OJ, Hunter T. Turnover of the active fraction of IRS1 involves raptor-mTOR- and S6K1-dependent serine phosphorylation in cell culture models of tuberous sclerosis. Mol Cell Biol. 2006;26(17):6425-34.

115. Tzatsos A, Kandror KV. Nutrients suppress phosphatidylinositol 3-kinase/Akt signaling via raptor-dependent mTOR-mediated insulin receptor substrate 1 phosphorylation. Mol Cell Biol. 2006;26(1):63-76.

116. Yu Y, Yoon SO, Poulogiannis G, Yang Q, Ma XM, Villén J, et al. Phosphoproteomic analysis identifies Grb10 as an mTORC1 substrate that negatively regulates insulin signaling. Science. 2011;332(6035):1322-6.

117. Liu P, Gan W, Inuzuka H, Lazorchak AS, Gao D, Arojo O, et al. Sin1 phosphorylation impairs mTORC2 complex integrity and inhibits downstream Akt signalling to suppress tumorigenesis. Nat Cell Biol. 2013;15(11):1340-50.

118. Rodrik-Outmezguine VS, Chandarlapaty S, Pagano NC, Poulikakos PI, Scaltriti M, Moskatel E, et al. mTOR kinase inhibition causes feedback-dependent biphasic regulation of AKT signaling. Cancer Discov. 2011;1(3):248-59.

119. Chandarlapaty S, Sawai A, Scaltriti M, Rodrik-Outmezguine V, Grbovic-Huezo O, Serra V, et al. AKT inhibition relieves feedback suppression of receptor tyrosine kinase expression and activity. Cancer Cell. 2011;19(1):58-71.

120. Carracedo A, Pandolfi PP. The PTEN-PI3K pathway: of feedbacks and cross-talks. Oncogene. 2008;27(41):5527-41.

121. Wang X, Trotman LC, Koppie T, Alimonti A, Chen Z, Gao Z, et al. NEDD4-1 is a proto-oncogenic ubiquitin ligase for PTEN. Cell. 2007;128(1):129-39.

122. Shafei MA, Forshaw T, Davis J, Flemban A, Qualtrough D, Dean S, et al. BCATc modulates crosstalk between the PI3K/Akt and the Ras/ERK pathway regulating proliferation in triple negative breast cancer. Oncotarget. 2020;11(21):1971-87.

123. Baryawno N, Sveinbjörnsson B, Eksborg S, Chen CS, Kogner P, Johnsen JI. Small-molecule inhibitors of phosphatidylinositol 3-kinase/Akt signaling inhibit Wnt/beta-catenin pathway cross-talk and suppress medulloblastoma growth. Cancer Res. 2010;70(1):266-76.

124. Mendoza MC, Er EE, Blenis J. The Ras-ERK and PI3K-mTOR pathways: cross-talk and compensation. Trends Biochem Sci. 2011;36(6):320-8.

125. Guan KL, Figueroa C, Brtva TR, Zhu T, Taylor J, Barber TD, et al. Negative regulation of the serine/threonine kinase B-Raf by Akt. J Biol Chem. 2000;275(35):27354-9.

126. Ma L, Chen Z, Erdjument-Bromage H, Tempst P, Pandolfi PP. Phosphorylation and functional inactivation of TSC2 by Erk implications for tuberous sclerosis and cancer pathogenesis. Cell. 2005;121(2):179-93.

127. Carriere A, Romeo Y, Acosta-Jaquez HA, Moreau J, Bonneil E, Thibault P, et al. ERK1/2 phosphorylate Raptor to promote Ras-dependent activation of mTOR complex 1 (mTORC1). J Biol Chem. 2011;286(1):567-77.

128. Carrière A, Cargnello M, Julien LA, Gao H, Bonneil E, Thibault P, et al. Oncogenic MAPK signaling stimulates mTORC1 activity by promoting RSK-mediated raptor phosphorylation. Curr Biol. 2008;18(17):1269-77.

129. Romeo Y, Zhang X, Roux PP. Regulation and function of the RSK family of protein kinases. Biochem J. 2012;441(2):553-69.

130. Siddiqui N, Sonenberg N. Signalling to eIF4E in cancer. Biochem Soc Trans. 2015;43(5):763-72.

131. Carracedo A, Ma L, Teruya-Feldstein J, Rojo F, Salmena L, Alimonti A, et al. Inhibition of mTORC1 leads to MAPK pathway activation through a PI3K-dependent feedback loop in human cancer. J Clin Invest. 2008;118(9):3065-74.

132. Cao Z, Liao Q, Su M, Huang K, Jin J, Cao D. AKT and ERK dual inhibitors: The way forward? Cancer Lett. 2019;459:30-40.

133. Ruicci KM, Pinto N, Khan MI, Yoo J, Fung K, MacNeil D, et al. ERK-TSC2 signalling in constitutively-active HRAS mutant HNSCC cells promotes resistance to PI3K inhibition. Oral Oncol. 2018;84:95-103.

134. Mundt F, Rajput S, Li S, Ruggles KV, Mooradian AD, Mertins P, et al. Mass Spectrometry-Based Proteomics Reveals Potential Roles of NEK9 and MAP2K4 in Resistance to PI3K Inhibition in Triple-Negative Breast Cancers. Cancer Res. 2018;78(10):2732-46.

135. Li Q, Li Z, Luo T, Shi H. Targeting the PI3K/AKT/mTOR and RAF/MEK/ERK pathways for cancer therapy. Mol Biomed. 2022;3(1):47.

136. Fukumoto S, Hsieh CM, Maemura K, Layne MD, Yet SF, Lee KH, et al. Akt participation in the Wnt signaling pathway through Dishevelled. J Biol Chem. 2001;276(20):17479-83.

137. Cross DA, Alessi DR, Cohen P, Andjelkovich M, Hemmings BA. Inhibition of glycogen synthase kinase-3 by insulin mediated by protein kinase B. Nature. 1995;378(6559):785-9.

138. Gazitt Y, Kolaparthi V, Moncada K, Thomas C, Freeman J. Targeted therapy of human osteosarcoma with 17AAG or rapamycin: characterization of induced apoptosis and inhibition of mTOR and Akt/MAPK/Wnt pathways. Int J Oncol. 2009;34(2):551-61.

139. Svirshchevskaya EV, Mariotti J, Wright MH, Viskova NY, Telford W, Fowler DH, et al. Rapamycin delays growth of Wnt-1 tumors in spite of suppression of host immunity. BMC Cancer. 2008;8:176.

140. Persad S, Troussard AA, McPhee TR, Mulholland DJ, Dedhar S. Tumor suppressor PTEN inhibits nuclear accumulation of beta-catenin and T cell/lymphoid enhancer factor 1-mediated transcriptional activation. J Cell Biol. 2001;153(6):1161-74.

141. Sharma M, Chuang WW, Sun Z. Phosphatidylinositol 3-kinase/Akt stimulates androgen pathway through GSK3beta inhibition and nuclear beta-catenin accumulation. J Biol Chem. 2002;277(34):30935-41.

142. Davis NM, Sokolosky M, Stadelman K, Abrams SL, Libra M, Candido S, et al. Deregulation of the EGFR/PI3K/PTEN/Akt/mTORC1 pathway in breast cancer: possibilities for therapeutic intervention. Oncotarget. 2014;5(13):4603-50.

143. Boyer A, Paquet M, Laguë MN, Hermo L, Boerboom D. Dysregulation of WNT/CTNNB1 and PI3K/AKT signaling in testicular stromal cells causes granulosa cell tumor of the testis. Carcinogenesis. 2009;30(5):869-78.

144. Mulholland DJ, Dedhar S, Wu H, Nelson CC. PTEN and GSK3beta: key regulators of progression to androgen-independent prostate cancer. Oncogene. 2006;25(3):329-37.

145. Desbois-Mouthon C, Cadoret A, Blivet-Van Eggelpoël MJ, Bertrand F, Cherqui G, Perret C, et al. Insulin and IGF-1 stimulate the beta-catenin pathway through two signalling cascades involving GSK-3beta inhibition and Ras activation. Oncogene. 2001;20(2):252-9.

146. Kaler P, Godasi BN, Augenlicht L, Klampfer L. The NF-κB/AKT-dependent Induction of Wnt Signaling in Colon Cancer Cells by Macrophages and IL-1β. Cancer Microenviron. 2009;2(1):69-80.

147. Song S, Mazurek N, Liu C, Sun Y, Ding QQ, Liu K, et al. Galectin-3 mediates nuclear beta-catenin accumulation and Wnt signaling in human colon cancer cells by regulation of glycogen synthase kinase-3beta activity. Cancer Res. 2009;69(4):1343-9.
