# Supplementary material for: PI3K/AKT/mTOR signaling transduction pathway and targeted therapies in cancer
Source: Mol Cancer. 2023 Aug 18;22:138. doi: 10.1186/s12943-023-01827-6 (PMC10436543; doi:10.1186/s12943-023-01827-6)
Supplement: Supplementary file 3 — Additional file 3: Supplementary information 2. [file 12943_2023_1827_MOESM3_ESM.docx]

**SUPPLEMENTARY INFORMATION 2**

**PI3K INHIBITORS**

The PI3K/AKT/mTORC (PAM) signaling pathway is probably the most common aberrantly induced signal transduction axis in cancer, and as a result, represents a primary therapeutic target. Notably, along the PAM pathway, PI3K is a major drug target for cancer treatment since its hyperactivity is remarkably correlated with human tumor progression, enhanced tumor microvessel formation, and increased number of invasive cancer cells (1). A strenuous effort has been committed to improve inhibitors targeting PI3K signaling. Indeed, several pharmaceutical companies have developed drug inhibitors of PI3K during the last decades. Notwithstanding four inhibitors (alpelisib, copanlisib, duvelisib, and idelalisib) have been approved by the Food and Drug Administration (FDA) for cancer treatment (**SUPPLEMENTARY FIGURE 1**), there is still concern regarding the development of resistance, sensitivity markers, and toxicology. Importantly, PI3K inhibitors are classified into three main groups: pan-PI3K inhibitors (pan-PI3Ki), isoform-specific PI3K inhibitors (IS PI3Ki), and dual PI3K/mTOR inhibitors (dual PI3K/mTORi) (2) (**FIGURE 10**).

**PAN-PI3K INHIBITORS**

Pan-PI3Ki suppress the cataytic activity of all four PI3K class I isoforms: PI3Kα, PI3Kβ, PI3Kγ, and PI3Kδ, encoded by *PIK3CA*, *PIK3CB*, *PIK3CG*, and *PIK3CD*, respectively. Thus, these drugs are normally efficient in tumors producing high level of PIP3, regardless of the type of PI3K genes or *PTEN* alterations implicated. Potentially, pan-PI3Ki provide a broader range of activity by comprising numerous molecular targets, although exists increasing risk of on-target and off-target effects, as well as possibly enhanced toxicity exist (3). Herein, we included the major pan-PI3Ki that are currently in different stages of human clinical trials. A summary of the most representative studies on pan-PI3Ki is shown in **SUPPLEMENTARY TABLE 1**.

**Buparlisib** (BKM120, NVP-BKM120)

Buparlisib is a potent oral reversible bioavailable agent that inhibits pan-class I PI3K in an ATP-competitive manner. Buparlisib targets p110 -α, -β, -δ, and -γ PI3K isoforms with IC_50_ values of 52 nM, 166 nM, 116 nM and 262 nM, respectively (4). Buparlisib suppresses PAM pathway by blocking phosphorylation of PIP2, and thus, by impeding its conversion into PIP3, hampering AKT overactivation, thereby limiting protein synthesis and cell growth (5). In addition, buparlisib has also been shown to inhibit tumor cell growth through FOXO3a-dependent PUMA induction in colon cancer cells (6). Interestingly, a study has reported that buparlisib in combination with vincristine, and to a lesser extent with doxorubicin, can exert synergistic effect in terms of reduced proliferation, increased cell mortality, and enhanced caspase-3 activity in several PI3K-upregulated osteosarcoma cell lines (7). It has been shown that PD-L1 expression is associated with PAM pathway. Accordingly, buparlisib treatment for 72 hours lead to PD-L1 downregulation in head and neck squamous cell cancer (HNSCC) cells, therefore implying that inhibition of PAM pathway can represent a further therapy for patients showing poor response to immune checkpoint therapy (8). Moreover, buparlisib not only has displayed significant inhibition of proliferation *in vitro*, but also has demonstrated robust anticancer activity in human tumor xenograft models with regular drug tolerance (4). Clinical studies with favourable or acceptable safety profile have shown anticancer activity of buparlisib, both singly and in combination, in several advanced solid tumors, and hematological malignancies (9) (10) (11) (12) (13) (14) (15) (16) (17) (18) (19) (20) (21) (22) (23) (24) (25) (26) (27) (**SUPPLEMENTARY TABLE 1**).

**CH5132799** (CH5)

CH5132799 is a potent anticancer agent against tumors harboring oncogenic *PIK3CA* mutations. Therefore, prediction of response to this drug based on *PIK3CA* mutations may allow patient stratification in clinical settings. Accordingly, cancers with *PIK3CA* mutations are remarkably sensitive to CH5132799 *in vitro* (28). In human cancer cell lines with activated PI3K pathway, CH5132799 has shown significant inhibitory activity against PI3Kα by resulting in a reduction in cell proliferation. Moreover, orally available CH5132799 demonstrates significant anticancer activity in PI3K pathway-activated human tumor xenograft models in mice (29). In line with this, cancers with *PIK3CA* mutations are significantly suppressed by CH5132799 in mouse xenograft models. Combination of CH5132799 with trastuzumab determines cancer resolution in trastuzumab-insensitive breast cancer model harbouring a *PIK3CA* mutation. Furthermore, CH5132799 displays advantages over mTORC1 inhibitors by exerting a concrete inhibition of PAM signaling since it suppresses 4E-BP1 phosphorylation and avoids S6K inhibition-mediated AKT activation. Importantly, these features can result in a regression of cancers that have regrown after long-term mTOR- inhibitor everolimus treatment (28). A clinical study with favourable safety profile has demonstrated anticancer activity of CH5132799 monotherapy in advanced solid tumors (30) (**SUPPLEMENTARY TABLE 1**).

**Copanlisib** (BAY 80-6946, Aliqopa)

Copanlisib is a robust and remarkably selective pan-PI3Ki with targeted activity predominantly against PI3K p110α and p110δ isoforms (31). Copanlisib stimulates growth inhibition and apoptosis through modulation of the AKT/FOXO3a/PUMA signalling axis in colorectal cancer (CRC) cells. Thus, PUMA-mediated apoptosis is essential for the antitumor effects of copanlisib, and manipulation of PUMA could possibly enhance its activity (32). Copanlisib remarkably inhibits PAM pathway activation, resulting in reduced cell viability and proliferation in gastrointestinal stromal tumor (GIST) cells. Moreover, concurrent inhibition of PI3K with copanlisib and tyrosine-protein kinase KIT (CD117) inhibitor imatinib, leads to further reduction of cell viability. Besides, high levels of cleaved-caspase 3 and phospho-S6 proteins support the significant antiproliferative effect of copanlisib in GIST. Furthermore, copanlisib both singly and combined with imatinib inhibits tumor growth in GIST xenografts (33). Interestingly, copanlisib has been found to synergise with conventional agents such as venetoclax in T-cell lymphoma *in vivo*, and B-cell lymphoma models (34). Copanlisib has exhibited remarkable clinical benefits in relapsed follicular lymphoma patients who have received at least two prior systemic therapies, and thus has been FDA-approved for use in this cohort (35) (**SUPPLEMENTARY FIGURE 1**). Clinical studies with favourable or acceptable safety profile have shown anticancer activity of copanlisib, both singly and in combination, in different hematological malignancies, as well as in advanced solid tumors (36) (37) (38) (39) (40) (41) (42) (43) (44) (45) (46) (47) (48) (48) (**SUPPLEMENTARY TABLE 1**).

**Pictilisib** (GDC-0941, RG7321)

Pictilisib is a potent PI3K inhibitor that increase the lysosomal compartment through transcription factor EB, priming glioblastoma cells to lysosomal membrane permeabilization, and triggering cell death through induction of caspase-3, activation of BAX, and loss of MtMP, a key indicator of mitochondrial activity (49). Pictilisib sensitises breast cancer to ABT-737 both *in vitro* and *in vivo*. In fact, ABT-737 alone exerts modest lethality in breast cancer cells, but in combination with pictilisib achieves a remarkable synergistic cytotoxicity towards cancer cells. Accordingly, ABT-737 in combination with pictilisib has been shown to significantly increase caspase-mediated apoptosis, and most importantly, promote proteasomal degradation of MCL1, whose overexpression is the main cause of ABT-737 resistance (50). Interestingly, using an *in vitro* co-culture system, presence of macrophages results in the activation of NF-κB signaling in breast cancer cells, thereby increasing resistance to pictilisib. Additionally, combination of pictilisib with aspirin arrests the activation of NF-κB signaling, leading to attenuation of cell growth and enhancement of apoptosis in breast cancer cells (51). Combination of pictilisib with CXCR1/2 analogue (G31P) enhances their efficiency against breast cancer, by causing a significant reduction in cancer cell proliferation, and an increase in apoptosis. Moreover, migration of breast cancer cell lines is remarkably decreased by the combination therapy, compared to treatment of each compound singly (52). Pictilisib treatment significantly improves the anticancer effect of doxorubicin, and prevents tumor-mediated bone destruction in osteosarcoma by suppressing the PI3K/AKT signalling pathway *in vitro*. Indeed, pictilisib increases the sensitivity of osteosarcoma cell lines to doxorubicin by promoting cell cycle arrest at G0/G1 phase and S phase. Moreover, pictilisib suppresses tumor-related osteolysis *in vivo*, through blockage of the PI3K/AKT pathway (53). Pictilisib has shown significant antitumor activity in human tumor xenograft murine models (54) and is presently in clinical development (3). Pictilisib enhances macrophage infiltration and determines the expression of macrophage-associated chemokines and cytokines in mouse models of breast cancer. Besides, combination of pictilisib with aspirin arrests the activation of NF-κB signaling, thereby causing an enhancement of apoptosis, a decreased in cancer burden, macrophage infiltration, and pulmonary metastasis in mouse models of breast cancer (51). Clinical studies with favourable or acceptable safety profile have displayed anticancer activity of pictilisib, both singly and in combination, in a variety of advanced solid tumors (55) (56) (57) (57) (58) (58) (59) (**SUPPLEMENTARY TABLE 1**).

**Pilaralisib** (XL147, SAR-245408)

CDKL5 overexpression enhances the PI3K/AKT axis, promotes proliferation, increases invasion, growth, migration, and drug resistance of glioma cells *in vitro*. Interestingly, combination of pilaralisib with cisplatin significantly reduces cell proliferation and CDKL5-induced drug resistance in xenografts overexpressing CDKL5 (60). Clinical studies with favourable or acceptable safety profile have shown anticancer activity of pilaralisib, both singly and in combination, in several advanced solid tumors (61) (62) (63) (64) (65) (66) (67) (**SUPPLEMENTARY TABLE 1**).

**SF1126** (936487-67-1)

SF1126 significantly suppresses proliferation, survival, and cell cycle progression in primary human colon tumor cells, and CRC cell line HT-29. A remarkable induction of apoptosis is observed after SF1126 treatment on CRC cells. Moreover, SF1126 arrests mTORC1/mTORC2 transduction signaling, and downregulates BRD4-targeted oncogenic cyclin D1 and MYC proteins. Notably, SF1126 also activates p38 signaling in CRC cells, whereas p38 inhibitors suppresses SF1126-induced apoptosis and cytotoxicity towards CRC cells (68). SF1126 reduces the expression level of proto-oncogene c-Myc in hepatocellular carcinoma cells. In fact, SF1126 arrests reciprocal action of BRD4 with acetylated histone-H4 chromatin mark protein, and dislocate BRD4 co-activator protein from the transcriptional start site of MYC in hepatocellular carcinoma cells (69). Moreover, SF1126 inhibits cell survival and suppresses phosphorylation of AKT in several human Ewing sarcoma cell lines and shows a remarkable decrease in tumor volume in Ewing sarcoma xenograft models, suggesting that dual PI3K/BRD4 inhibition, exerts significant anticancer activity (70). Additionally, subcutaneous treatment with SF1126 remarkably reduces human colorectal cancer HT-29 xenograft tumor growth in nude mice (68). Moreover, SF1126 treatment either singly or in combination with sorafenib demonstrates remarkable anticancer activity in hepatocellular carcinoma *in vivo* (69).

**Sonolisib** (PX-866)

Inhibition of PI3K by sonolisib reduces temozolomide-induced autophagy and endorses apoptosis in glioblastoma cells. Indeed, blood-brain barrier (BBB) penetrating sonolisib is also an inhibitor of autophagic flux, and thus, sequential treatment of temozolomide followed by sonolisib, instead of combined co-treatment, can effectively suppress autophagy-induced survival in gliobastoma cells while enhancing apoptosis (71). Clinical studies with favourable safety profile have demonstrated the anticancer activity of sonolisib, both singly and in combination, in different advanced solid tumors (72) (73) (74) (75) (**SUPPLEMENTARY TABLE 1**).

**ZSTK474** (475110-96-4)

ZSTK474 has been shown to suppress PI3K downstream pathways and inhibit cell growth in several different cancer cell lines. Besides, ZSTK474 induces apoptosis in synovial sarcoma, alveolar rhabdomyosarcoma, and Ewing's sarcoma cell lines, suggesting that ZSTK474 can be a potential agent for treating sarcomas (76). In addition, ZSTK474 displays a robust induction of G1 cell cycle arrest, as well as strong antiproliferative activity in acute myeloid leukemia HL60 cells, and in adriamycin-resistant HL60/ADR cells. Moreover, ZSTK474 decreases phosphorylated Rb, enhances p27, and reduces cyclin D1 protein level in dose-dependent manner in both cell lines. Besides, ZSTK474 treatment also decreases downstream PI3K pathway proteins such as phosphorylated AKT, PDK1, and GSK3β, in both cell lines. Furthermore, ZSTK474 enhances intracellular accumulation of adriamycin, reverses adriamycin resistance, and decreases the expression of multi drug resistance (MDR) proteins such as MRP1 and P-gp in HL60/ADR cells. Furthermore, combination of ZSTK474 with chemotherapeutic drugs vincristine or cytarabine results in a synergistic effect in both cell lines (77). ZSTK474 induces autophagy and G1 cell cycle blockage in human breast cancer MCF-7 cells. This ZSTK474-induced cell cycle arrest in G1 phase is mediated by the PI3K/AKT/GSK3β/cyclin D1/p-Rb axis. Additionally, ZSTK474 in combination with autophagy inhibitors increases cell viability, emphasising that ZSTK474-mediated autophagy may be contributing to the anticancer efficacy (78). Interestingly, combination of ZSTK474 and ERK1/2 inhibitor AZD0364 exhibits a synergistic anticancer activity in acute lymphoblastic leukemia REH and MOLT-4 cell lines, as well as acute myeloid leukemia MOLM-14 cells. Indeed, this combination remarkably reduces both AKT and ERK1/2 activation, inhibits cell viability, enhances the production of reactive oxygen species (ROS), and increases apoptosis in these leukemia cells (79). In addition, ZSTK474 significantly reduces cell growth in several melanoma cell lines, as well as in vemurafenib-resistant melanoma cells (80).

**ISOFORM-SPECIFIC PI3K INHIBITORS**

IS PI3Ki have been established to target cancer types dependent on either PI3Kα, or PI3Kβ, or PI3Kγ, or PI3Kδ isoforms. Conventionally, these drugs show a wider therapeutic index, and lesser off-target-based toxicologic effects due to the reduced expression of the diverse PI3K isoforms in non-cancerous cells. Notably, PI3Kα and PI3Kβ isoforms are ubiquitously expressed, whereas PI3Kγ and PI3Kδ isoforms are predominantly restrained to leukocytes (81). Herein, we included the major IS PI3Ki that are currently in various stages of clinical trials. A summary of the most representative studies on IS PI3Ki is shown in **SUPPLEMENTARY TABLE 1**.

*PI3Kα Inhibitors*

**Alpelisib** (NVP-BYL719, BYL719, Piqray)

Alpelisib, a potent and selective agent with targeted efficacy against PI3K p110α isoform, has shown anticancer efficacy in tumor xenograft models, mostly with mutation or amplification of *PIK3CA*, emphazising its efficacy in *PIK3CA*-altered cancer patients (82). Alpelisib augments the anticancer activity of paclitaxel in human gastric cancer in vitro. Indeed, alpelisib monotherapy reduces AKT and S6K1 phosphorylation and causes G0/G1 phase arrest of the cell cycle, independently of *PIK3CA* mutational status, whereas combination of alpelisib and paclitaxel synergistically enhances the antiproliferative effects, especially on *PIK3CA*-mutated cells, leading to increased DNA damage response and apoptosis. Besides, this combination improves the anti-migratory activity of *PIK3CA*-mutant gastric cancer cells and increases anticancer activity by reducing Ki-67 expression and enhancing apoptosis in xenograft models of MKN1 gastric cancer cells. Furthermore, this combination also prolongs the survival of cancer-bearing mice (83). Alpelisib in combination with fulvestrant has exhibited significant clinical benefits in men and postmenopausal women with HR^+^, HER2^-^, *PIK3CA*-mutated, advanced or metastatic breast cancer, and as a result has been FDA-approved for use in this cohort (84) (**SUPPLEMENTARY FIGURE 1**). Clinical studies with favourable or acceptable safety profile have shown anticancer activity of alpelisib, both singly and in combination, in a variety of advanced solid tumors, especially breast cancer (85) (86) (87) (88) (89) (90) (91) (92) (93) (94) (95) (96) (97) (98) (99) (100) (101) (102) (103) (104) (105) (106) (107) (**SUPPLEMENTARY TABLE 1**).

**AZD8835** (1620576-64-8)

PI3Kα and PI3Kδ inhibitor AZD8835 demonstrates significant anticancer efficacy through reduction of proliferation, and induction of apoptosis, in activated B-cell-like (ABC)-diffuse large B-cell lymphoma (DLBCL) *in vitro*, and in patient-derived xenograft mouse models. Additionally, combination of AZD8835 with ibrutinib synergistically enhances the antitumor effects both *in vitro* and *in vivo* (108). Besides, AZD8835 inhibits AKT phosphorylation, and downstream effectors 4E-BP1 and S6K. In addition, AZD8835 enhances sensitivity of ovarian cancer cells to paclitaxel and cisplatin treatment. Moreover, AZD8835 reduces p-ERK protein expression, as well as BRCA1 and BRCA2 mRNA expression, in OVCAR-8 cells with *KRAS* mutation, and brings about a remarkable reduction in tumorigenesis in mouse xenograft models (109). PI3Kα/δ inhibition with AZD8835 shows cancer growth suppression by an intermittent dosing schedule *in vivo*. Indeed, AZD8835 exerts remarkable anticancer activity, associated with enhanced CD8^+^ T-cell activation and memory, as well as suppression of regulatory T-cells (Tregs), in mouse syngeneic cancer models. AZD8835 determines significant CD8^+^ T-cell activation, associated with improving effector cell function and cell viability. Therefore, AZD8835 can be considered a promising immune-oncology drug, which strongly warrants its further clinical investigation (110).

**Serabelisib**(TAK-117, MLN1117, INK1117)

Combination of serabelisib with novel mTORC1/mTORC2 inhibitor TAK-228 shows synergistic anticancer effects both *in vitro* and *in vivo*. In fact, this combination remarkably reduces cell proliferation in human bladder cancer cell lines, and suppresses cancer growth, as well as angiogenesis in preclinical bladder tumor models (111). The most common adverse event caused by serabelisib in healthy subjects is nausea. Interestingly, the percentage of adverse events decreases when serabelisib is combined with lansoprazole. This suggests that scrupulous management of intragastric pH modulatory concomitant medications may be required when serabelisib is used as a cancer treatment (112). A clinical study with acceptable safety profile has displayed limited anticancer activity of serabelisib monotherapy, whereas another clinical trial with favourable safety profile has shown efficacy of serabelisib in triple combination with sapanisertib and paclitaxel, in advanced solid tumors (113) (114) (**SUPPLEMENTARY TABLE 1**).

**Taselisib** (GDC-0032, RG7604)

Clinical studies with favourable or acceptable safety profile have demonstrated anticancer activity of taselisib combined with other drugs, in several advanced solid tumors, particularly breast cancer. Conversely, taselisib monotherapy has only exhibited modest effectiveness in advanced solid tumors (115) (116) (117) (118) (119) (120) (121) (122) (123) (**SUPPLEMENTARY TABLE 1**).

*PI3Kβ Inhibitors*

**Acalisib** (CAL-120, GS-9820)

A clinical study with acceptable safety profile has shown anticancer activity of acalisib monotherapy in relapsed or refractory lymphoid malignancies (124) (**SUPPLEMENTARY TABLE 1**).

**AZD8186** (1627494-13-6)

AZD8186 monotherapy exhibits antitumor activity towards *PTEN*-deficient triple-negative breast cancer (TNBC) cells by inhibiting PI3K signalings, promoting apopotosis, and reducing proliferation; however, AZD8186 displays mild efficiency *in vivo*. Nevertheless, combination of AZD8186 with paclitaxel and anti-PD1 remarkably increases the overall efficacy *in vivo* (125). Moreover, combination of AZD8186 with selumetinib, used to simultaneously target p110β/PI3K and MEK respectively, significantly reduces the proliferation of primary human pleural malignant mesothelioma cells and improves the survival of *PTEN*/*Trp53*-null mice without severe toxicity. These findings can be considered a rationale for using AZD8186 combined with selumetinib in patients suffering from malignant mesothelioma with sarcomatoid characteristics (126). AZD8186 inhibits phosphorylation of AKT1, AKT2, and RPS6 for four to seven hours post treatment, in PC3 prostate cancer cell line and HCC70 TNBC cell line xenograft tumors. In addition, AZD8186 suppresses AKT3 phosphorylation in PC3 xenografts at all doses tested, but only at the highest dose in HCC70 xenografts (127). Clinical studies with acceptable safety profile have displayed anticancer activity of AZD8186, both singly and in combination, in advanced solid tumors (128) (129) (**SUPPLEMENTARY TABLE 1**).

**GSK2636771** (GSK-2636771)

GSK2636771 impedes the growth of *Rac1*-mutated melanoma cells, but not *BRAF*-altered melanoma cells, suggesting that targeting PI3Kβ inhibitors such as GSK2636771 can be considered as a promising strategy to inhibit Rac1 signalling in malignant melanoma (130). OX40 agonist-based treatments are a novel approach to enhance the efficacy of tumor immunotherapy since its activation and signaling improves the anticancer activity of CD8^+^ T-cells. Interestingly, combination of GSK2636771 with anti-OX40 reduces cancer growth and prolongs the survival of mice with *PTEN*-null melanomas. Even though this combination treatment exhibits no increase in the number of tumor-infiltrating lymphocytes, it remarkably increases proliferation of CD8^+^ tumor-infiltrating lymphocytes, and augments serum level of interferon-γ (IFNγ), CCL4, and CXCL10, which are primarily generated by effector T-cells and/or memory T-cells (131). Clinical studies with acceptable safety profile have shown anticancer activity of GSK2636771, both singly and in combination, in different advanced solid tumors (132) (133) (134) (135) (**SUPPLEMENTARY TABLE 1**).

**SAR260301** (SAR-260301)

SAR260301 monotherapy blocks PI3K pathway signaling, particularly in *PTEN*-deficient human tumor models, and in combination with BRAF inhibitor vemurafenib or MEK inhibitor selumetinib shows synergistic anticancer activity in *PTEN*-deficient/*BRAF*-altered human melanoma cancer models. Tolerability of both combinations yields favorable results without major adverse events, providing preclinical basis to support the evaluation of SAR260301-based combinations in further clinical studies (136). Phase 1 SAR260301 monotherapy has shown no objective response, with grade 3 pneumonitis dose-limiting toxicity (DLT), in different advanced-stage solid tumour patients. According to pharmacokinetics data, the rapid clearance of SAR260301 hinders a proper inhibition of PI3Kβ, and thus, this drug has not been further advanced towards the clinic (137).

*PI3Kγ Inhibitors*

**Duvelisib** (IPI-145, INK-1197)

Duvelisib, a vigorous and selective PI3Kγ/PI3Kδi, determines a reduction in primary chronic lymphocytic leukemia (CLL) cells in a dose- and time-dependent manner, without causing cytotoxicity to normal human B cells (138). A study carried out with chronic lymphocytic leukemia patient samples has shown that various BH3-only pro-apoptotic genes are upregulated with duvelisib treatment. Since anti-apoptotic protein Bcl-2 is also upregulated in duvelisib-treated patient samples, combination of duvelisib and Bcl-2 inhibitor venetoclax leads to increased apoptosis in chronic lymphocytic leukemia cells (139). Duvelisib has exhibited remarkable clinical benefits in relapsed or refractory CLL or small lymphocytic lymphoma (SLL) patients after at least two prior therapies, and in relapsed or refractory follicular lymphoma (FL) patients after at least two prior systemic therapies, and thus has been FDA-approved for use in these cohorts (140) (**SUPPLEMENTARY FIGURE 1**). Clinical studies with favourable or acceptable safety profile have demonstrated anticancer activity of duvelisib, both singly and in combination, in several hematological malignancies (141) (142) (143) (144) (145) (146) (147) (148) (149) (**SUPPLEMENTARY TABLE 1**).

**Eganelisib** (IPI-549)

Eganelisib significantly causes reduced cell survival and proliferation in a dose-dependent manner in HSC-2 and Cal-27 oral squamous cell carcinoma cells (150). Interestingly, eganelisib reverses P-gp-mediated MDR in paclitaxel-treated human colon carcinoma SW620/AD300 cells. Indeed, eganelisib increases the load of paclitaxel into the cell and suppresses the clearance of paclitaxel out of SW620/AD300 cells. Moreover, eganelisib enhances the anticancer efficacy of paclitaxel in MDR SW620/AD300 xenograft tumors overexpressing P-gp (151). Intravenously delivered combination of eganelisib with anti-fibrotic agent silibinin in mice leads to an increment in anticancer efficacy and apoptotic cancer tissue compared to each treatment singly in 4T1 breast cancer cell-derived tumors. Moreover, a remarkable decrease in Tregs and myeloid-derived suppressor cells (MDSCs) is also observed. Additionally, normalised tumor microenvironment features are also detected, including antifibrotic effects, inhibition of angiogenesis, and the suppression of collagen formation in cancer tissue, remarkably improving the overall anticancer effects (152). Clinical studies with favourable or acceptable safety profile have shown anticancer activity of eganelisib in combination with nivolumab in advanced solid tumors (153) (154) (**SUPPLEMENTARY TABLE 1**).

**Tenalisib** (RP-6530)

Tenalisib is a dual PI3Kγ/PI3Kδi that has demonstrated efficacy, in preclinical and clinical studies, to target cancer cells and tumor microenvironment in Hodgkin lymphoma. In fact, tenalisib remarkably inhibits proliferation of Hodgkin lymphoma cells *in vitro*, and downregulates lactic acid metabolism, thereby transforming macrophages from an immunosuppressive M2-like state to a typical inflammatory M1-like phenotype. The specific PI3Kδ/PI3Kδγ-dependent signaling associated with the metabolic reprogramming of tumor cells is identified as tumor glycolysis; and besides, the metabolic regulator pyruvate kinase M2, is identified as the main mediator of cancer-induced immunosuppressive state of macrophages. Moreover, tenalisib is able to repolarize tumor-associated macrophages (TAMs) into pro-inflammatory macrophages, and therefore inhibits angiogenesis in cancer, which results in cancer regression in human tumor xenografts (155). A clinical study with favourable safety profile has displayed anticancer activity of tenalisib monotherapy in relapsed or refractory peripheral and cutaneous T-cell lymphoma (156) (**SUPPLEMENTARY TABLE 1**).

*PI3Kδ Inhibitors*

**Acalisib** (GS-9820, CAL-120)

Already mentioned above in the PI3Kβ inhibitors.

**AMG-319** (ACP-319)

A significant improvement in anticancer activity is identified by exposing lymphoma cell lines to a combination of AMG-319 and Bruton tyrosine kinase (BTK) inhibitor acalabrutinib, compared to each agent singly. AMG-319 also demonstrates antitumor activity in pre-clinical models of mantle cell lymphoma (MCL), marginal zone lymphoma (MZL), and ABC-DLBCL (157). Accordingly, combination of AMG319 with acalabrutinib enhances survival and cancer control in a CLL mouse model. Notably, single-drug therapy increases survival by a week compared to control mice, but combination therapy extends survival by over two weeks compared with either single agent. Moreover, this combination remarkably reduces cancer burden in the peripheral blood and spleen of mice, decreases cancer proliferation, diminishes the expression of anti-apoptotic MCL-1 and Bcl-xL proteins, and suppresses NF-κB signaling, more significantly, compared to single-drug therapy (158). In a human study, AMG319 has proven evidence of cancer therapeutic activity in CLL patients. Besides, AMG319 determines serious adverse events, including anaemia and colitis, each occurring in 10.7% patients (159).

**AZD8835** (1620576-64-8)

Already mentioned above in the PI3Kα inhibitors.

**Duvelisib** (IPI-145, INK-1197)

Already mentioned above in the PI3Kγ inhibitors.

**Idelalisib** (GS-1101, CAL-101)

Idelalisib is a PI3Kδi that is cancer selective and does not interfere with PAM signaling that sustains for normal cellular function. Idelalisib has been found to reduce AKT activation, reduce cell viability, and increase apoptosis, in Hodgkin lymphoma cellular models. Moreover, decrease of cell viability and apoptosis are further enhanced when idelalisib is combined with mTOR inhibitor everolimus (160). Idelalisib significantly blocks constitutive signaling proximally downstream of PI3K in primary lymphoma patient cells, displaying a remarkable inhibition of p-AKT levels (161). Idelalisib treatment, at doses of 40 µg/g or 80 µg/g for 5 days/week for 4 weeks, induces apoptosis of lymphoid tissues in intraperitoneally injected mice. In fact, idelalisib causes thymic involution, reduces CD4^+^/CD8^+^ T-cell population, and enhances CD4^-^/CD8^-^ T-cell population. Besides, idelalisib reduces lymphocyte viability and cell count in the spleen. In addition, idelalisib augments cleaved caspase-3 expression in the spleen, lung, and thymus tissues. Moreover, idelalisib increases thoracic and airway resistance, and reduces thoracic compliance (162). Furthermore, combination of idelalisib with Bcl-2 inhibitor venetoclax exerts significant antitumor activity in follicular lymphoma in *ex vivo* studies. In fact, idelalisib reshapes the follicular lymphoma immune microenvironment and re-establishes dependency on Bcl-2, thereby causing cell death. Also, addition of idelalisib reduces the cytotoxic activity of venetoclax. Therefore, this study provides a rationale for studying further combinations of PI3Kδi and venetoclax in future clinical trials (163). Idelalisib has displayed significant clinical benefits as monotherapy in SLL and FL patients who have received at least two prior systemic therapies, and in combination with rituximab in CLL patients for whom rituximab singly would be considered appropriate therapy due to co-morbidities. As a result, idelalisib has been FDA-approved for use in this cohorts (164) (165) (**SUPPLEMENTARY FIGURE 1**). Clinical studies with favourable or acceptable safety profile have shown anticancer activity of idelalisib, both singly and in combination, in different hematological malignancies (166) (167) (168) (169) (169) (169) (170) (171) (172) (173) (174) (175) (176) (177) (178) (179) (180) (181) (182) (**SUPPLEMENTARY TABLE 1**).

**Linperlisib** (YY-20394, PI3Kδ-IN-2)

Clinical studies with acceptable safety profile have exhibited significant anticancer activity of linperlisib monotherapy in hematological malignancies (183) (184) (**SUPPLEMENTARY TABLE 1**).

**ME-401** (1595129-71-7)

ME-401 is an orally absorbed specific inhibitor that exerts its mechanism of action by accumulating in target tissue and cells with strong affinity in a B-cell lymphoma mouse model. This feature highlights the clinical efficiency detected in B-cell malignancies that differentiate ME-401 from other PI3Kδi already approved or in development (185). A phase 1 study of ME-401 has shown that pharmacodynamics and pharmacokinetics are positive with a single dose of 60 mg displaying maximal PI3Kδ inhibition in healthy volunteers (186). Additionally, another study has demonstrated that using an intermittent ME-401 dosing schedule results in a low rate of grade 3 side effects in most patients (187).

**Parsaclisib** (INCB050465, IBI-376)

Parsaclisib is a highly selective, next-generation PI3Kδi that differs in structure from first-generation PI3Kδ inhibitors. Parsaclisib shows potent anticancer and immunomodulatory activities, and decreases hepatotoxicity in B-cell malignancy models. Indeed, PI3Kδ exerts more than 1000-fold selectivity for B cell neoplasms compared to other classes of PI3K isozymes, and directly blocks PI3K signaling that mediates cell proliferation in B-cell lines *in vitro*. Moreover, parsaclisib can also indirectly control cancer growth by reducing immunosuppression, through regulatory T-cell inhibition, in syngeneic lymphoma models. Interestingly, DLBCL cell lines, which overexpress MYC, display no response to parsaclisib-induced proliferation blockade through inhibition of PI3Kδ signaling; despite this, their proliferation is decreased by suppressing MYC gene transcription. These observations emphasise that hepatotoxicity detected with PI3Kδ first-generation inhibitors could be determined by an inadequate drug molecular structure, leading to an off-target effect. Currently, parsaclisib is being tested in several clinical trials as a therapy against hematologic B-cell malignancies (188). Clinical studies with acceptable safety profile have demonstrated anticancer activity of parsaclisib monotherapy in B-cell lymphomas (189) (190) (191) (192) (**SUPPLEMENTARY TABLE 1**).

**Tenalisib** (RP-6530)

Already mentioned above in the PI3Kγ inhibitors.

**Umbralisib** (TGR-1202, RP5264)

Umbralisib, a dual inhibitor of PI3Kδ and casein kinase-1-ε, sustains normal and CLL-associated FoxP3+ human Tregs in *ex vivo* studies. Indeed, umbralisib treatment preserves Treg number and function in CLL-bearing mice, whereas duvelisib- and idelalisib-treated mice show reduced numbers of Tregs, decreased Treg function, and enhanced immune-mediated toxicity, emphasising that inhibition of casein kinase-1-ε improved CLL Treg number and function. Moreover, casein kinase-1-ε inhibition mitigates impairment of CLL Tregs by PI3K inhibitors in combinatorial treatments, suggesting that a reduction of adverse events of umbralisib is determined by its dual PI3Kδ/CK1ε inhibitory activity (193). Combination of umbralisib with JAK1/2 inhibitor ruxolitinib in *ex vivo* experiments with primary chronic myelomonocytic leukemia (CMML) patient samples has shown synergism for cell viability and clonogenicity. Additionally, this combination promotes a concurrent reduction of AKT, STAT5, ERK, and S6 phosphorylation. These results highlight the importance of dual-inhibiting PI3Kδ and JAK1/2, thereby supporting the use of this combined drug therapy in CMML (194). Notably, umbralisib received its first FDA approval in 2021 for the treatment of relapsed or refractory MZL patients who had received ≥ 1 prior anti-CD20-based regimen, and relapsed or refractory FL patients who had received ≥ 3 prior lines of systemic therapy (195). However, due to safety concerns, the FDA withdrew its approval in 2022. Clinical studies with favourable or acceptable safety profile have shown anticancer activity of umbralisib, both singly and in combination, in different hematological malignancies (196) (197) (198) (199) (200) (201) (202) (**SUPPLEMENTARY TABLE 1**).

**DUAL PI3K/mTOR INHIBITORS**

Dual PI3K/mTORi are mostly effective against all PI3K isoforms, as well as mTORC1/mTORC2, leading to suppression of the three crucial intersections of the PAM signaling pathway. Herein, we included the major dual PI3K/mTORi currently in different stages of clinical trials. A summary of the most representative studies on dual PI3K/mTORi is shown in **SUPPLEMENTARY TABLE 1**.

**Apitolisib** (GDC-0980, RG7422, GNE 390)

Apitolisib is known to be particularly beneficial in patients with mutations in genes along the PAM pathway. Indeed, apitolisib treatment with gemcitabine and/or cisplatin synergistically decreases cholangiocarcinoma cell growth through the inhibition of PAM pathway (203). Furthermore, combination of apitolisib plus venetoclax shows potent anti-acute myeloid leukemia activity *in vitro*, mainly through the induction of pro-apoptotic proteins BAX and BAK, emphasising that dual PI3K and Bcl-2 inhibition merit further evaluation for effective treatment of acute myeloid leukemia (204). Clinical studies with acceptable safety profile have only displayed modest anticancer activity of apitolisib monotherapy in advanced solid tumors (205) (206) (**SUPPLEMENTARY TABLE 1**).

**BGT226** (NVP-BGT226)

BGT226 demonstrates potent cytotoxic activity in hypoxic hepatocellular carcinoma cells. Indeed, BGT226 reduces cell proliferation, decreases survival, and inhibits angiogenesis by reducing the expression of vascular endothelial growth factor (VEGF) and transcription factor HIF1α, suggesting that this drug is a promising candidate for hepatocarcinoma therapy (207). BGT226 exerts significant cytotoxic effect towards pancreatic cancer cells via mTOR signaling. L-leucine promotes proliferation of pancreatic cancer cells and enhances expression of Sestrin2 and p-mTOR proteins, suggesting that sestrin2 endorses the progression of pancreatic cancer through PAM signaling. Notably, sestrin2 overexpression increases glycolysis of pancreatic cancer cells and engenders their proliferation, but this effect is eliminated by BGT226-mediated PI3K/mTOR inhibition (208). Moreover, combination of BGT226 with gefitinib shows synergistic anticancer effects in non-small-cell lung cancer (NSCLC) cell lines. Indeed, suppression of PAM signalling and induction of apoptosis are intensified by this combination. Additionally, remarkable cancer growth suppression is observed in a xenograft model with this combination (209).

**Bimiralisib** (PQR309)

Bimiralisib exhibits anti-lymphoma activity *in vitro*, both singly, and in combination with either ibrutinib, or venetoclax, or rituximab, or panobinostat, or lenalidomide, or marizomib, or ARV-825. In addition, this drug exerts anticancer activity in cells with primary or secondary resistance to idelalisib. Interestingly, sensitivity to bimiralisib is particularly associated with high expression of transcripts coding for the B-cell receptor pathway. Thus, bimiralisib can be considered a potential drug that is worth developing to treat lymphoma (210). Moreover, bimiralisib reduces proliferation, causes G1-phase cell cycle arrest, and enhances apoptosis in human glioblastoma cells. Accordingly, the expression of AKT, p‑AKT, and cyclin D1 decreases, whereas the expression of BAX, BAD, cleaved caspase‑3, Bcl‑2, and Bcl‑xL increases. Furthermore, migration and invasion of glioma cells show a remarkable reduction after treatment with bimiralisib, providing significant evidence for further development of this compound (211). Interestingly, glioblastoma cell lines with increased level of active p-AKT, decreased level of phosphorylated inactive protein translation repressor EIF4E-BP1 (p4E-BP1), and enhanced level of (Ser9)-phosphorylated inactive GSK3β (p-GSK3β), are more responsive to bimiralisib. Accordingly, the anticancer activity of bimiralisib is synergistically potentiated by either direct pharmacological inhibition of AKT or silencing of AKT gene (212). Studies in preclinical models suggest that bimiralisib can exert antitumor activity in HNSCC, and its efficacy has been found to be enhanced with inactivating NOTCH1 mutations (213). More importantly, bimiralisib is able to cross the BBB, displaying efficient pharmacokinetic parameters in rats and mice, and reduction of proliferation in cancer cell lines, as well as rat xenograft models. Besides, this drug shows favourable tolerability, and is thus a potential clinical candidate for the treatment of primary brain cancers and central nervous system metastasis (214). Moreover, bimiralisib singly exerts *in vivo* antitumor activity in an orthotopic LN-229 glioma xenografts in nude mice. In addition, combining bimiralisib with direct AKT pharmacological inhibition or AKT gene silencing, synergistically enhances the anticancer efficacy in these glioma xenograft models (212). Clinical studies with acceptable safety profile of bimiralisib monotherapy have shown anticancer activity in advanced solid tumors, but with limited efficacy in lymphoma (215) (216) (**SUPPLEMENTARY TABLE 1**).

**Dactolisib** (BEZ-235, NVP-BEZ235)

Dactolisib is an imidazo [4,5-c] quinoline derivative drug that inhibits PI3K and mTOR kinase catalytic activities by binding to their ATP-binding cleft (217). Dactolisib stimulates mutant p53 degradation to exert anticancer effects on TNBC cells. In fact, dactolisib inhibits growth, migration and formation of colonies in TNBC cells. Moreover, dactolisib treatment leads to degradation of mutp53 in MDA-MB-231 and MDA-MB-468 TNBC cell lines. Additionally, dactolisib induces autophagy through repression of the PAM signaling pathway. Furthermore, there is positive feedback between mutp53 and autophagy in TNBC cells (218). Dactolisib also inhibits thyroid cancer cell growth by p53-dependent and p53-independent p21 upregulation. Besides, the anticancer effects of dactolisib are more prominent in p53 wild-type thyroid cancer cells, in comparison to p53 mutant thyroid cancer cells. Additionally, GSK3β/β-catenin signaling suppression is implicated in the p21-induced G0/G1 cell cycle arrest in both p53 wild type thyroid cancer cells and mutant thyroid cancer cells treated with dactolisib (219). Dactolisib singly shows significant anticancer activity by inducing cytotoxicity and apoptosis in glioblastoma cell lines (220). Combination of dactolisib with diosmin determines synergistic anti-proliferative effects, enhances caspase-3-induced apoptosis, reduces angiogenesis, and thus inhibits the signaling transduction of PAM pathway in HCT-116 CRC cell line (221). In addition, combination of dactolisib with multi-kinase inhibitor regorafenib exercises synergistic antitumor effects in hepatocellular carcinoma. Indeed, this combination treatment augments the suppression of cell proliferation and enhances cleaved caspase-3 and cleaved PARP expression in hepatocellular carcinoma cells. Additionally, this combination inhibits cell migration, suppresses cell invasion, and reduces the expression of epithelial-mesenchymal transition (EMT)-related genes such as vimentin, slug, and MMP9/2. Besides, suppression of AKT, mTOR, S6K, and 4EBP1 phosphorylation has also been confirmed (222). Moreover, combination of dactolisib with temozolomide plus simultaneous radiotherapy, more significantly reduces cell viability, enhances pro-apoptotic effects, inhibits migration and invasion, increases p27 expression, and decreases Bcl-2 expression, compared to temozolomide plus radiotherapy, in glioblastoma cells (220). Dactolisib has exhibited adequate anticancer effects in preclinical studies in various cancers, such as colorectal cancer (223) (224), renal cancer (225), prostate cancer (226), lung cancer (227), TNBC (228) (229), melanoma (230), lymphoma (231), ovarian cancer (232), thyroid cancer (219), and glioblastoma (220). However, dactolisib monotherapy has been reported to exert only modest antitumor clinical activity with inconsistent pharmacokinetic characteristics in solid tumors (233) (234), and in combination, for instance with abiraterone acetate, has been shown to produce mild anticancer clincial activity, mainly due to intolerability in solid tumor patients (235). Clinical studies with acceptable safety profile have demonstrated anticancer activity of dactolisib monotherapy in a variety of advanced solid tumors (236) (237) (**SUPPLEMENTARY TABLE 1**).

**DS-7423** (DS7423)

DS-7423, a small molecule dual PI3K/mTORi, exerts anticancer activity and promotes p53-dependent apoptosis in ovarian clear cell adenocarcinoma (OCCA). In addition, a reduction in S phase and an enhancement of sub-G1 phase of the cell cycle treatment is detected after DS-7423 in majority of OCCA cell lines. Moreover, DS-7423 treatment induces more apoptosis in cell lines without TP53, compared to others with *TP53* mutations. In line with this, concurrent increased level of p53 phosphorylation at Ser46, as well as reduced phosphorylation level of (p53 inhibitor) MDM2, is determined by DS-7423 treatment, in *TP53* wild-type cell lines. Besides, DS-7423 causes upregulation of genes that mediate p53-dependent apoptosis, including PUMA and P53AIP1. Furthermore, DS-7423 significantly reduces cancer growth of ovarian clear cell adenocarcinoma in a dose-dependent manner in mouse xenograft models, suggesting that DS-7423-induced dual PI3K/mTOR inhibition can represent a strategic molecular target therapy for ovarian clear cell adenocarcinoma (238). Interestingly, dual inhibition of PAM pathway by DS-7423 and MDM2 by RG7112 exerts synergistic antiproliferative effect in ovarian clear cell carcinoma cell lines without *TP53* mutations. This combination more significantly induces pro-apoptotic protein PUMA and cleaved PARP with augmented sub-G1 population and apoptotic cells, compared to either agent singly. In addition, this combination remarkably decreases cancer volume in mice, and robustly reduces vascularity and cell proliferation, with higher apoptotic cell death in xenograft tumors (239). In other preclinical studies, DS-7423 remarkably has reduced the growth of glioma cancer cell lines and glioma-initiating cell lines. Importantly, cell lines with PI3K gene alterations and *PTEN* mutations are particularly responsive, through inhibition of cell growth, to DS-7423 treatment. DS-7423 demonstrates robust capacity to cross the BBB in mice, leading to significant suppression of PI3K signaling biomarkers in the brain. Additionally, DS-7423 displays steady survival benefit and stable efficacy in the orthotopic models of glioblastoma multiforme. Besides, DS-7423 treatment increases the anticancer potency of temozolomide against glioblastoma multiforme in glioma models, emphasising that dual inhibition of PI3K and mTOR can enhance cytotoxicity mediated by alkylating drug, and thus, suggesting a novel regimen treatment for glioblastoma multiforme, which can be appropriate for patients characterised by PI3K-activated and/or *PTEN*-altered tumors (240). A clinical study with acceptable safety profile has shown anticancer activity of DS-7423 monotherapy in advanced solid tumors (241) (**SUPPLEMENTARY TABLE 1**).

**Gedatolisib** (PF-05212384, PKI-587)

Gedatolisib is known to repress an mTORC2-induced negative feedback loop, resulting in MEK/ERK pathway overactivation in pancreatic cancer cells (242). Moreover, gedatolisib provokes a remarkable reduction of cell cycle with G0/G1 blockage, and activation of apoptosis in neuroendocrine tumor cells (243). Gedatolisib determines growth suppression (244) and enhances radio-sensitization in several HNSCC cell lines (245). Furthermore, overexpression of ATP-binding cassette drug transporters, particularly ABCB1 and ABCG2 transporters, which represent one of the most common mechanism for developing multidrug resistance, is reported to reduce the potency of gedatolisib in colorectal cancer cells. Besides, gedatolisib is a drug substrate of both ABCB1 and ABCG2. Therefore, combination of gedatolisib with ATP-binding cassette transporter inhibitors is possibly necessary to improve the overall efficacy toward colon cancer cells (246). Gedatolisib enhances chemosensitivity of oxaliplatin through hindering DNA damage repair pathways such as homologous recombination (HR) and non-homologous end joining (NHEJ), and inhibiting the PAM signaling pathway, in SK-Hep1 and HepG2 hepatocellular carcinoma cell lines. Indeed, combination of gedatolisib and oxaliplatin more significantly reduces proliferation, augments apoptosis, increases the G0/G1 phase arrest of the cell cycle, and enhances the number of γ-H2AX/cells, compared to oxaliplatin treatment singly (247). Interestingly, gedatolisib potently sensitizes and eliminates quiescent breast cancer cells seeded in organotypic bone marrow cultures to chemotherapy (248). A *PTEN*-deficient patient-derived tumor xenograft study has shown that combination of gedatolisib with either paclitaxel, or cisplatin, or dacomitinib, enhances chemotherapy efficacy in TNBC and low-grade serous ovarian cancer models, whereas no improvement is detected in *KRAS* and *TP53* mutant lung adenocarcinoma models (249). Besides, gedatolisib significantly enhances the radiosensitization in preclinical non-metastatic HNSCC models (245). Gedatolisib has also demonstrated antitumor activity by promoting apoptosis, without toxicity, in ovarian cancer xenograft models (250). Moreover, combination of gedatolisib with oxaliplatin significantly reduces cancer growth *in vivo*, through antitumor effects associated with induction of mitochondrial apoptosis, and suppression of AKT/mTOR phosphorylation, as well as γ-H2AX phosphorylation (247). Clinical studies with favourable or acceptable safety profile have displayed anticancer activity of gedatolisib, both singly and in combination, in several advanced solid tumors (251) (252) (253) (253) (254) (255) (**SUPPLEMENTARY TABLE 1**).

**Omipalisib** (GSK2126458, GSK458)

Omipalisib, a potent highly selective small-molecule ATP-competitive dual PI3K/mTORCi, can significantly hamper ovarian cancer carcinogenesis and metastasis. Indeed, omipalisib potently inhibits cell proliferation, and strongly reduces cell migration in ovarian cancer and patient-derived ovarian primary cancer cell lines. Importantly, the efficacy of omipalisib-mediated inibition on cell proliferation and migration is mostly the same as that of paclitaxel. In addition, the anti-cancer activity of omipalisib is associated with inactivation of AKT and mTOR, and activation of cell cycle arrest at the G0/G1 phase (256). Ompalisib hampers NHEJ and sensitizes different types of tumor cells to chemotherapy and radiotherapy. In fact, omipalisib enhances DNA damage-induced cell death, and impedes ionizing radiation- and doxorubicin-induced phosphorylation of DNA-PKcs, thereby leading to suppression of the NHEJ pathway (257). Ompalisib significantly inhibits proliferation and invasion of pancreatic cancer cells, highlighting the importance of PAM signaling pathway in the onset and progression of pancreatic cancer (258). Macrocyclization is a novel promising strategy used in drug discovery, since the semi-rigid character of these structures can determine improved selectivity, potency, and favorable pharmacokinetic features. Notably, this approach permits access to new chemical space leading to attaining a more suitable property position. A series of novel small molecule macrocycles (MCXs) based on omipalisib exhibit potent biochemical and cellular dual PI3K/mTOR inhibition, and show remarkable anticancer effects in human tumor cell lines. Therefore, these *in vitro* experiments reveal omipalisib as an appropriate candidate for future *in vivo* pharmacokinetic and pharmacodynamic studies in mouse tumor models (259). Moreover, combination of omipalisib with dual PI3K/mTORCi gedatolisib displays suppression of cancer progression and enhances radiosensitivity in nasopharyngeal carcinoma *in vitro*. Indeed, this combination remarkably decreases cell proliferation and motility in nasopharyngeal carcinoma cells, and inhibits phosphorylation of AKT, mTOR, S6, and 4E-BP1 proteins. Additionally, this combination sensitizes nasopharyngeal carcinoma cells to ionizing radiation by increasing DNA damage, inducing apoptosis, and increasing the G2-M cell cycle delay (260). Omipalisib exhibits significant antitumor activity in solid renal tumors through abrogation of cell proliferation and enhancement of apoptosis in genetically engineered TSC2^+/-^ mice. In fact, omipalisib significantly decreases the number and size of solid renal tumors, and reduces the number and size of solid, cystic, and papillary lesions. Additionally, as expected, omipalisib exerts a strong inhibition of PI3K and mTOR in renal tumors (261). Furthermore, omipalisib significantly reduces ovarian cancer growth and metastasis in SKOV3 cell intraperitoneally-engrafted nude mice or patient-derived tumor cell xenografts. Omipalisib anti-cancer activity is correlated to activation of G0/G1 phase arrest of the cell cycle, and inhibition of AKT and mTOR. Notably, the inhibitory effect of omipalisib is statistically comparable to that of paclitaxel, further emphasising the potent antitumor activity of this agent (256). In addition, combination of omipalisib with gedatolisib plus ionizing radiation significantly induces apoptosis, suppresses the phosphorylation of AKT, mTOR, and 4E-BP1, and reduces cancer growth *in vivo*, suggesting that this combination can represent a promising therapeutic strategy for nasopharyngeal carcinoma (260). A clinical study with acceptable safety profile has shown moderate anticancer activity of omipalisib monotherapy in advanced solid tumors (262) (**SUPPLEMENTARY TABLE 1**).

**Panulisib** (P7170)

Panulisib, a potent and selective imidazoquinoline-based PI3K inhibitor, significantly reduces phosphorylation of AKT, S6 and 4E-BP1 level by 80-100%, and decreases tumor growth in human NSCLC cells. Besides, panulisib decreases colony formation, and induces apoptosis in NSCLC patient cancer-derived NSCLC cell lines (263). Panulisib displays a strong anticancer activity in endocrine-sensitive and endocrine-resistant estrogen receptor positive (ER^+^) breast cancer. Indeed, panulisib significantly increases apoptosis, remarkably inhibits PI3K at a dose of ≥ 200 nM, and reduces mTOR activity at a dose of ≤ 25 nM in cell lines (264). Moreover, panulisib treatment shows strong anticancer efficacy and reduced ALK1-induced angiogenic activity. In fact, panulisib remarkably inhibits phosphorylation of p-AKT (S473), as well as S6K (T389), and significantly reduces proliferation in colon, ovarian, renal, and prostate cancer cell lines. Additionally, panulisib promotes blockage of G1 phase-S phase cell cycle transition in these cell lines. Moreover, *in vivo* matrigel plug, *in vitro* human umbilical vein endothelial cell (HUVEC) tube formation, and rat aorta ring assays proves that panulisib exert significant antiangiogenic activity. Furthermore, knockdown of *ALK1* in HUVECs, demonstrates that panulisib-induced antiangiogenic activity is achieved through the inhibition of ALK1. Besides, panulisib also exhibits a remarkable cancer growth inhibition, in a dose-depended manner, in several human cancers when given orally at doses ranging from 10 mg/kg to 20 mg/kg in mouse xenografts (265). In addition, panulisib at 20 mg/kg daily, remarkably suppresses the growth of human NSCLC xenografts, independently of *PIK3CA*, *KRAS*, or epidermal growth factor receptor (*EGFR*) mutations, or erlotinib treatment response (263). Also, panulisib significantly inhibits cancer growth, suppresses growth of fulvestrant-resistant cancers, and reduces tumor cell proliferation in mice bearing endocrine-sensitive and endocrine-resistant ER^+^ breast cancer xenografts, suggesting that the major mechanism of panulisib anticancer efficacy occurs through the inhibition of mTOR signaling (264).

**Paxalisib** (GDC-0084, RG7666)

Paxalisib significantly reduces cutaneous squamous cell carcinoma (cSCC) cell growth *in vitro*. Indeed, paxalisib remarkably decreases proliferation and survival of established SCL-1, SCC-13, A431 cell lines, as well as primary human cSCC cells. Moreover, paxalisib triggers apoptosis and causes cell cycle blockage in primary human cSCC cells. Additionally, particularly in A431 cells and primary human cSCC cells, paxalisib suppresses phosphorylation of crucial PAM pathway proteins, such as p85, AKT, S6, and S6K1. Besides, paxalisib also reduces the activation of DNA-PKcs, the catalytic subunit of a nuclear DNA-dependent Ser/Thr protein kinase DNA-PK, in primary human cSCC cells, which results in paxalisib-induced cell death and apoptosis in A431 cells (266). Importantly, paxalisib is a brain-penetrant small molecule that has shown a remarkable dose-dependent anticancer activity *in vitro*. Indeed, paxalisib significantly reduces cell viability, enhances apoptosis, cell-cycle arrest, and decreases phosphorylation of AKT and p70S6 kinase, in breast cancer brain metastatic cell lines harbouring a *PIK3CA* mutation. Conversely, paxalisib causes only growth inhibition in breast cancer brain metastatic cell lines with *PIK3CA* wild type (267). *In vivo*, daily administration of paxalisib robustly reduces A431 cell line-xenograft cancer growth in cSCC mice. Also, in paxalisib-treated cancer tissues, activation of PI3K, AKT, mTOR, and DNA-PKcs are remarkably inhibited (266). Moreover, paxalisib treatment potently suppresses growth of *PIK3CA*-mutant, but not growth of *PIK3CA* wild-type brain tumors, in breast cancer brain metastasis xenograft mouse models. Paxalisib can therefore be considered a promising treatment for breast cancer brain metastases with altered PI3K and mTOR signaling determined by activating *PIK3CA* mutations (267). Furthermore, paxalisib at steady-state concentration of 45 mg/once daily determines enduring anticancer activity in glioma xenograft models (268). A clinical study with acceptable safety profile has demonstrated anticancer activity of paxalisib monotherapy in high-grade glioma (269) (**SUPPLEMENTARY TABLE 1**).

**PF-04691502** (1013101-36-4)

PF-04691502, a potent ATP competitor inhibitor, decreases phosphorylation of AKT and S6 ribosomal protein thereby inhibiting cell proliferation in *PI3KCA*-mutated and *PTEN*-deleted cancer cell lines (270). PF-04691502 also exhibits significant activity through its activation of G1 cell cycle blockage and apoptosis in numerous aggressive B-cell non-Hodgkin lymphomas (NHL) cell lines (271). PF-04691502 enhances apoptosis and disrupts micro-environmental signaling in chronic lymphocytic leukemia *in vitro* (272). Combination of PF-04691502 with radiation exerts synergistic anticancer effects on HNSCC cells. However, this combination is ineffective in HNSCC cells characterised by a higher mutational burden and multiple variants in the PAM pathway, highlighting the need for alternative therapeutic strategies in non-responsive HNSCCs (273). Combination of PF-04691502 with radiotherapy enhances radiosensitization in NETs. Indeed, exposure to PF-04691502 two days after radiotherapy more significantly enhances apoptosis, in comparison to either PF-04691502 or radiotherapy singly, in neuroendocrine tumor (NET) cell lines. Thus, schedule-dependent administration of PF-04691502 in combination with radiotherapy can increase cytotoxicity by engendering the radiosensitivity of NET cells, suggesting that this combination may potentially represent a suitable therapeutic regimen for gastroenteropancreatic NET patients (274). PF-04691502 significantly inhibits cell growth in cutaneous T-cell lymphoma (CTCL) cell lines. Moreover, PF-502 also exhibits anticancer activity in patient-derived CTCL cells (275). Preclinical studies of PF-04691502 have shown that PF-04691502-induced PI3K/mTOR suppression can increase the expression of *TP53*/*TP73*, and consequently, reduces tumor growth in murine knockout models and human xenografts of HNSCC (276). Furthermore, in a *PTEN*-deficient patient-derived tumor xenograft, PF-04691502 combined either with paclitaxel, or cisplatin, or dacomitinib, increases the chemotherapy efficacy in TNBC and low-grade serous ovarian cancer models, whereas no improvement is detected in *KRAS* and *TP53* mutant lung adenocarcinoma model (249). PF-04691502 demonstrates antitumor activity by promoting apoptosis in ovarian cancer xenograft models, but however, minimally affects proliferation markers such as Ki67 and phospho-histone H3. Besides, no toxicity is detected during this treatment (250). Moreover, PF-04691502 treatment of tumor-bearing animals leads to a transient lymphocytosis, followed by a decrease of tumor in blood, spleen, bone marrow, and lymph nodes (272). Furthermore, PF-502 also displays anticancer activity in a patient-derived CTCL xenograft mouse model, by increasing survival and inducing apoptosis in treated mice (275).

**PI103** (PI-103)

PI-103 is a small organic heterotricyclic PI3K inhibitor molecule that selectively inhibits recombinant PI3K isoforms p110α, p110β, and p110δ, and blocks mTOR and DNA-PK, by targeting the ATP binding sites of PI3K and mTOR (277). Indeed, PI103 remarkably inhibits constitutive- and growth factor-induced activation of PI3K/mTORC1, blocks cell proliferation, and drives G1 phase arrest of the cell cycle in human leukemia cell lines (278). Interestingly, combination of PI103 and histone-lysine N-methyltransferase enzyme EZH2 inhibitor EPZ-6438 synergistically decreases invasion, angiogenesis, and stemness potential in glioblastoma multiforme U-87 cells (279). PI103 is also able to chemosensitise glioblastoma cell lines, as well as glioblastoma stem cells, to apoptosis by hampering the mechanism of DNA repair. In fact, combined treatment of PI103 with DNA-damaging agents, particularly doxorubicin, remarkably enhances apoptosis, and decreases colony formation, compared to chemotherapy treatment singularly. The molecular mechanism explaining this chemosensitisation is demonstrated by the suppression of PI3K, mTOR, and DNA-PK. Notably, inactivation of PI3K or DNA-PK, but not mTOR, delays the resolution of doxorubicin-mediated DNA damage and concurrently enhanced apoptosis (280). Interestingly, PI103 treatment exhibits a significant decrease of proliferation in human glioma cell lines, as well as reduction of tumor growth, invasion, angiogenesis, and metastasis in human glioma xenograft tumors, thereby highlighting a potential efficacy due to combined inhibition of PI3K and mTOR in malignant glioma. Besides, the safety profile of PI103 yields favorable results since there is no detectable toxicity (281). Moreover, PI103 primes neuroblastoma cells for TRAIL-mediated apoptosis by changing the balance toward pro-apoptotic Bcl-2 family members, enhances mitochondrial apoptosis, and suppresses clonogenic survival *in vitro*. Additionally, PI103 significantly decreases cancer growth in a neuroblastoma *in vivo* model. Therefore, PI103 is a novel promising agent to increase the efficacy of TRAIL-based treatments in neuroblastoma (282). Combination of PI103 with PARP inhibitor olaparib significantly increases radiation-induced apoptosis in BRCA-proficient TNBC cells, and as a result, remarkably decreases tumor volume in xenografts. Thus, targeting the PAM signaling pathway with concurrent PARP inhibition maybe a novel strategy to augment the efficacy of radiation in BRCA-proficient TNBC patients (283). Combination of PI103 with chemotherapic doxorubicin synergistically activates apoptosis in neuroblastoma cells, and decreases cancer growth in neuroblastoma *in vivo* models. Indeed, this combination activates caspase 3, increases BID cleavage, induces BAX, suppresses cell proliferation, and determines G1 phase arrest of the cell cycle in neuroblastoma cells *in vitro*. Induction of caspase 3 is also detected in neuroblastoma xenografts in nude mice. In addition, this combinatorial treatment significantly hampers the growth of established neuroblastoma tumors and improves the survival of mice *in vivo* (284).

**Samotolisib** (LY3023414, GTPL8918)

Samotolisib has been tested in several cancer cell lines. Interestingly, an *in vitro* study has shown that overexpression of ATP-binding cassette drug transporters, which is notably a common mechanism for developing tumor cell multidrug resistance, remarkably hampers the efficiency of samotolisib in cancer cells. In fact, intracellular accumulation of samotolisib in cancer cells is robustly decreased by the drug efflux function of both ABCB1 and ABCG2, two well-known ATP-binding cassette drug transporters. As a result, samotolisib displays no significant efficacy to suppress PAM signaling pathway and determine blockage of G0/G1 cell cycle phase in tumor cells overexpressing ABCB1 or ABCG2 (285). Bone metastasis, which often leads to bone destruction, is rather frequent in many advanced cancers. Interestingly, samotolisib inhibits both osteogenesis and osteoclastogenesis through the PAM signalling pathway, which is often activated in osteosarcoma, in murine preosteoblast cells and bone marrow-derived macrophage cells. Indeed, samotolisib significantly reduces PI3K/AKT/GSK3-dependent activation of β-catenin during osteogenesis, and concurrently, decreases NFATc1 during osteoclastogenesis. Accordingly, siRNA-mediated gene knockdown of AKT isoforms downregulates osteoclastogenesis emphasising that AKT1 and AKT2 act synergistically. Thus, this study may contribute to a better understanding of the potential benefits of samotolisib in future cancer-related clinical applications (286). Clinical studies with favourable or acceptable safety profile have shown anticancer activity of samotolisib, both singly and in combination, in different advanced solid tumors (287) (288) (289) (290) (291) (292) (**SUPPLEMENTARY TABLE 1**).

**Voxtalisib** (XL765, SAR245409)

Voxtalisib significantly decreases proliferation, suppresses generation of PIP3 on the plasma membrane, and remarkably reduces phosphorylation of AKT, S6K, and S6, in a variety of cancer cell lines (293). Besides, voxtalisib exerts significant antiproliferative effects, mainly due to decreased cyclin B1/cyclin D1/cyclin E, CDK2/CDK4/CDK6, and concurrent increase of cell cycle regulators p21/p27 in prostate cancer cell lines. Antitumor activity of voxtalisib is correlated to an enhancement of nuclear localization of FOXO1a, with consequent induction of apoptosis. Additionally, voxtalisib treatment shows superior antitumor efficacy in prostate cancer cells, when compared to either PI3K inhibitor voxtalisib treatment or mTOR inhibitor rapamycin treatment, carried out in the same cell lines (294). Notably, voxtalisib inhibits cell growth by inducing endoplasmic reticulum stress-dependent apoptosis, occurring through the activation of CHOP/DR5 pathway, in T98G, U87MG, and A172 glioblastoma cell lines. Moreover, endoplasmic reticulum stress is determined by voxtalisib-induced suppression of mTOR signal, and not PI3K signal. In addition, combination of voxtalisib with temozolomide, a common glioblastoma chemotherapy, remarkably improves the antitumor efficacy in A172 glioblastoma cell line xenograft mouse model (295). Furthermore, oral administration of voxtalisib robustly inhibits AKT, S6K, and S6 phosphorylation in a dose-dependent manner, exerting a 24-hour active duration, in mouse xenograft models. Also, repeated dosing of voxtalisib leads to a remarkable cancer growth suppression in numerous human xenograft models in nude mice, which correlated to proapoptotic, antiproliferative, and antiangiogenic effects (293). Clinical studies with favourable or acceptable safety profile have displayed anticancer activity of voxtalisib, both singly and in combination, in a variety of advanced solid tumors, as well as B-cell malignancies (296) (297) (298) (299) (300) (301) (301) (302) (**SUPPLEMENTARY TABLE 1**).

**VS-5584** (SB2343)

VS-5584 exhibits a 30-fold higher efficacy in reducing survival and proliferation of cancer stem cells, compared to cancer cells in solid tumor cell populations (303). VS-5584 treatment determines a BIM-induced significant growth inhibition in multiple myeloma cell lines. Notably, VS-5584 displays an upregulation of class II tumor suppressor gene RARRES3, which contributes to induction of apoptosis, enhancement of cyclin D2 expression, and blockage of cell cycle, in myeloma patient cells. In addition, combination of VS-5584 with either velcade, dexamethasone, or panobinostat, exerts synergistic effects in several myeloma cell lines, and in CD138+ plasma cells isolated from different myeloma patients (304). Nowadays, VS-5584 can be considered a promising antitumor drug in application along with chemotherapy in PAM-indeced human osteosarcoma. Indeed, in a dose-depended manner, VS-5584 treatment significantly reduces growth of MG-63 and U2OS human osteosarcoma cells, by enhancing CDC2-, Cyclin B1-, p21-, and p27-induced G1-phase arrest. Moreover, VS-5584 treatment displays a robust suppression of the PAM signaling pathway. Furthermore, VS-5584 completely blocks cell migration and tube formation of HUVECs (305). Interestingly, VS-5584 remarkably increases the apoptotic effect, and consequently augments the anti-proliferative effect, of subtoxic dose arsenic trioxide through the suppression of NF-κB activity, a downstream protein of PAM pathway, in B cell precursor-acute lymphoblastic leukemia. Accordingly, this combination also leads to lower expression of NF-κB target genes involved in cell survival and proliferation (306). VS-5584 is reported to increase the anticancer efficacy of multi-tyrosine kinase inhibitor ponatinib in blasts and Philadelphia -negative leukemia stem cells in chronic myeloid leukemia (CML). In fact, this combination exerts synergistic antileukemic effects *in vitro*, since VS-5584 lowers the dose concentration of ponatinib necessary to target leukemic stem cells. Moreover, this combination treatment suppresses the PAM pathway more robustly, through decreasing p-AKT, p-mTOR, p-S6K, and p-PRAS40, compared to each agent singly, in leukemic stem cells. Consequently, the synergistic effects of this combination lead to subsequent downstream effects such as increased C/EBP transcriptional activity, as well as reduced activity of NFκB, STAT3, E2F/DP1, CREB, Myc/Max, Elk-1/SRF, and AP-1, in leukemic stem cells (307). Moreover, VS5584 synergistically enhances the antitumor activity of ATP-competitive and AKT2 selective inhibitor CCT128930 in human osteosarcoma cells. Indeed, combination of VS5584 and CCT128930 determines a more significant PAM pathway- and MAPK pathway-mediated apoptosis, shown through increased PARP cleavage and caspase-3 activation, compared to each drug singly, in human osteosarcoma cells (305). VS-5584 significantly inhibits PAM activation in established 786-O and A498 cell lines, and primary renal cell carcinoma cells, thereby reducing cell proliferation, cell survival, cell cycle progression, and cell migration. Additionally, VS-5584 determines a remarkable enhancement of apoptosis in renal cell carcinoma cells. Interestingly, VS-5584 treatment in renal cell carcinoma cells and 786-O tumor xenografts lead to feedback upregulation of BRD4. Accordingly, BRD4 complete knockout, or BRD4 knockdown, or JQ1-induced BRD4 inhibition, strongly enhances VS-5584-induced renal cell carcinoma cell death and apoptosis. Conversely, induced overexpression of BRD4 mitigates the cytotoxicity of VS-5584 in established 786-O cells. These results emphasise that VS-5584 anticancer activity is robustly potentiated by possibly co-targeting the inhibition of BRD4. Moreover, orally administered VS-5584 in a single daily dose of 20 mg/kg exerts a remarkable inhibition of established renal carcinoma 786-O tumor growth *in vivo* (308). Also, VS-5584 exerts significant anti-cancer efficacy by decreasing proliferation, causing G0/G1 phase arrest of the cell cycle, and suppressing the PAM pathway, in neuroblastomas cells. In addition, VS-5584 reduces the expression of p-S6K1, p-Rb, p-CDK2, and cyclin E1, and augments the expression of p21 and p27 in neuroblastoma cells. Furthermore, VS-5584 remarkably hampers tumor growth and downregulates the expression of p-mTOR and p-S6K1 in mouse neuroblastoma tissues (309). Importantly, VS-5584 also decreases cancer stem cell level in numerous mouse xenograft models of human tumor. In line with this, VS-5584 treatment *ex vivo* remarkably decreases cancer stem cells in resected ovarian and breast cancer patients. Contrary, cisplatin or paclitaxel chemotherapy displays minor efficacy in targeting cancer stem cells than bulk tumor cells. Moreover, VS-5584 arrests cancer regrowth after chemotherapy in small-cell lung cancer (SCLC) mouse xenograft models (303). Besides, VS-5584 remarkably decreases cancer volume and weight in multiple myeloma mouse xenografts (304).

**AKT INHIBITORS**

Overexpression of AKT has been detected in several cancers and is often associated with increased tumor cell survival and proliferation. Thus, targeting AKT could provide a strategic approach for cancer therapy. Numerous drugs can specifically inhibit AKT proteins, thereby impeding overactivation of downstream proteins in PAM signalling pathway (**FIGURE 10**). Herein, we included the major AKT inhibitors that are currently in different stages of human clinical trials. A summary of the most representative studies on AKT inhibitors is shown in **SUPPLEMENTARY TABLE 2**.

**Afuresertib** (GSK2110183)

Afuresertib is a competitive pan-AKT kinase domain inhibitor (310). Interestingly, ALK+ inhibitor ceritinib has been found to enhace the *in vitro* efficacy of afuresertib. In fact, this combination more significantly reduces proliferation, increases apoptosis, and decreases p-AKT as well as p-GSK3β levels, compared to each agent singly, in gastric cancer cell lines and gastric cancer patient-derived cells (311). Auresertib increases apoptosis, and enhances caspase-3 as well as caspase-7 activities in malignant pleural mesothelioma cells. Besides, afuresertib remarkably causes G_1_ phase arrest of the cell cycle, and accordingly, increases the expression of p21^WAF1/CIP1^ in these cells. In addition, afuresertib reduces phosphorylation of AKT substrates GSK3β and FOXO, suggesting that drug-mediated expression of p21 determines G1-phase cell cycle arrest by promoting FOXO transcriptional activity malignant pleural mesothelioma cells. Moreover, addition of afuresertib remarkably improves cisplatin-induced cytotoxicity in malignant pleural mesothelioma cells (312). Interestingly, the anticancer effects of phytochemical curcumin, mainly due to cycle arrest and enhanced apoptosis, are significantly augmented by afuresertib, in ML‑2 and OCI‑AML5 acute myeloid leukemia cells. In line with this, combination of afuresertib and curcumin synergistically inhibits the engraftment, proliferation, and survival of acute myeloid leukemia cells in an acute myeloid leukemia xenograft mouse model (313). Furthermore, combination of afuresertib and ALK+ inhibitor ceritinib has been reported to exert a strong synergistic enhancement of tumor regression in a mouse MKN45 gastric cancer model (311). Clinical studies with favourable or acceptable safety profile have shown anticancer activity of afuresertib monotherapy in advanced hematological malignancies, and in combination with paclitaxel plus carboplatin in platinum-resistant ovarian cancer (310) (314) (315) (316) (**SUPPLEMENTARY TABLE 2**).

**ARQ-751** (ARQ751, MK-4440)

ARQ 751 is an allosteric pan-AKT inhibitor, which reduces AKT activation by dephosphorylating its membrane-associated active conformation, and by impeding its inactive conformation from relocalizing to the plasma membrane. ARQ 751 decreases proliferation across numerous cancer types, including CRC, leukemia, endometrial cancer, and breast cancer cell lines. Besides, preclinical studies have shown sensitivity of *PIK3CA*, *PIK3R1*, *AKT1^E17K^*, or *PTEN*-mutated cancer cells towards ARQ 751. Moreover, ARQ 751 demonstrates significant anti-tumor activity in *AKT1^E17K^*-mutated endometrial patient-derived xenografts models, and other PAM pathway-activated cancer models (317). ARQ 751 significantly inhibits NHL cell proliferation, but however, exhibits modest effect on the viability of primary B-cells (318). Combination of ARQ 751 with first-line KIT inhibitor imatinib mesylate shows synergistic effects in imatinib mesylate-sensitive and -resistant gastrointestinal stromal tumor cell lines. Accordingly, this treatment combination significantly upregulates tumor suppressor PDCD4, and enhances cell death. Furthermore, *in vivo* studies demonstrate superior efficacy of this combination in an imatinib mesylate-sensitive preclinical model of gastrointestinal stromal tumor compared to each agent singly (319).

**BAY1125976** (BAY-1125976)

BAY 1125976 is an allosteric AKT1 and AKT2 inhibitor that exerts activity in several preclinical cancer models such as *AKT^E17K^*-mutant prostate cancer xenografts and *PIK3CA^H1074R^*-mutant breast cancer xenografts (320). A phase 1 study of BAY 1125976 has shown clinical benefit rate (CBR) of 27.9% in advanced solid cancer patients treated at the recommended phase 2 dose (RP2D) of 60 mg/twice daily. Nonetheless, despite the inhibition of AKT1 and AKT2, genetic analyses report additional mutations that may promote cancer cell growth. BAY 1125976, whose tolerability is satisfactory, inhibits AKT1 and AKT2 signaling; but however, results in no clinical cancer responses, suggesting that its monotherapy inhibitory activity is moderate towards AKT (321).

**Capivasertib** (AZD5363)

Capivasertib is a potent selective inhibitor of all three AKT isoforms (322). A study performed with breast cancer patient-derived xenografts (PDXs) to identify response biomarkers, and thus, elucidate mechanisms of resistance to capivasertib, has shown that mutations in *PIK3CA*/*AKT1* and the absence of mTORC1-activating alterations, such as in mTOR or TSC1, are linked with sensitivity to capivasertib monotherapy. In addition, resistant PDX models display low baseline pAKT (S473) and residual pS6 (S235) after capivasertib treatment, highlighting that parallel pathways can bypass AKT/S6K1 signaling. Moreover, loss of AKT1 p.E17K and cyclin D1 overexpression are identified as the two main mechanisms of acquired resistance to capivasertib (323). Notably, in a recent phase 3 CAPItello-291 study, combination of capivasertib and fulvestrant doubled PFS compared to fulvestrant singly in hormone receptor positive (HR^+^), HER2^-^ breast cancer patients who have developed resistance to aromatase inhibitors and CDK4/CDK6 inhibitors (324). Thus, capivasertib represents a new valuable treatment option for these patients and is expected to receive FDA approval in due time. Clinical studies with favourable or acceptable safety profile have demonstrated anticancer activity of capivasertib, both singly and in combination, in a variety of advanced solid tumors, particularly breast cancer (325) (326) (327) (328) (329) (330) (331) (332) (333) (322) (334) (335) (336) (337) (324) (**SUPPLEMENTARY TABLE 2**).

**CCT128930** (CCT-128930)

ATP-competitive and AKT2 selective inhibitor CCT128930 treatment demonstrates significant G1 phase arrest of the cell cycle, due to Cyclin B1, Cyclin D1, p27, and CDC2 dysfunctioning, and increase in apoptosis, due to enhanced PARP cleavage and caspase-3 activation, in U2OS and MG63 human osteosarcoma cell lines. Besides, CCT128930 remarkably suppresses p-AKT and p-p70S6K expression. Additionally, CCT128930 augments the antitumor activity of dual PI3K/mTORC inhibitor VS5584 in U2OS and MG63 human osteosarcoma cells, since this combination more robustly enhanced apoptosis by increasing PARP cleavage and caspase-3 activation, compared to each agent singly (338). In a three-dimensional cell culture study, combination of CCT128930 with paclitaxel shows a more efficient anticancer activity in reducing the viability of HeyA8 and SKOV3 ovarian cancer cells compared to each agent singularly. In fact, CCT128930 inhibits the expression of pS6 (S235/S236) and pS6 (S240/S244), in HeyA8 and SKOV3 cells, which counterbalanced paclitaxel-mediated upregulation of pS6 (S235/S236) and pS6 (S240/S244) in the same cell lines, occurring after paclitaxel single treatment (339). Cisplatin (CDDP)-resistant and radiation-resistant neuroblastoma cells often display an enhancement of AKT2/mTOR pathway and MAPK pathway. Interestingly, suppressing these two pathways with AKT2 inhibitor CCT128930 and MEK inhibitor PD98059 significantly reduces cell proliferation, angiogenesis, and cell migration in cisplatin/radiation-resistant cancer stem-like neuroblastoma cells. Moreover, the tumor-sphere formation of these cells is more sensitive to this combination, compared to each agent singly. Importantly, this combined treatment approach may represent an effective novel therapeutic strategy to attain a better clinical outcome in neuroblastoma patients (340). The major difficulty in targeting a cancer stem cell (CSC) subpopulation within a tumor is due to their peculiar transformation process, whereby CSCs are capable of differentiating to non-CSCs, and viceversa. Interestingly, CCT128930-induced suppression of AKT2 remarkably precludes non-CSC conversion through MET, thereby decreasing invasion and colony-forming capability of MDA-MB-231 breast CSCs in an ALDH1A1 expression-driven fluorescent CSC model. Furthermore, suppression of AKT2 decreases CSC viability in low attachment culture conditions. In line with this, marked AKT2 expression is often observed in circulating tumor cells in orthotopic tumor mouse models, highlighting AKT2 as a promising target for future antitumor therapies to prevent cancer recurrence through the inhibition of CSC metastatic potential (341).

**Ipatasertib** (GDC-0068)

Ipatasertib is a novel ATP-competitive drug that increase pro-apoptotic protein PUMA induction by FOXO3a and NF-κB, leading to colon cancer growth suppression. Indeed, ipatasertib treatment results in p53-independent PUMA activation by inhibiting AKT, thereby inducing both FoxO3a and NF-κB, which directly bind to PUMA promoter, resulting in increased PUMA transcription and BAX-mediated intrinsic mitochondrial apoptosis (342). Ipatasertib inhibits cancer cell proliferation and metastasis, as well as cytokine regulation and PD-L1 expression. Consequently, since AKT can be used to control breast cancer progression and immunosuppression, ipatasertib is currently undergoing clinical investigation to treat TNBC patients (343). Combination of ipatasertib with anti-HER2 monoclonal antibody trastuzumab significantly increases the anticancer activity in HER2^+^ gastric cancer cells. Indeed, this combination determines a remarkable decrease of proliferation and migration in HER2^+^ N87, OE19, and OE33 cell lines. Additionally, particularly in OE19 and OE33 cell lines, this combination suppresses the activation of proteins downstream of HER2 and AKT, highlighting that targeting HER2 and AKT can lead to an improved anticancer activity in HER2^+^ gastric cancer cells (344). Ipatasertib also suppresses colon cancer growth, in terms of weight and volume *in vivo* by inducing PUMA-dependent (but not p53-dependent) endogenous apoptosis, and showing direct antitumor activity through inhibition of AKT and its downstream pathways in xenograft mouse models (342). Clinical studies with favourable or acceptable safety profile have shown anticancer activity of ipatasertib, both singly and in combination, in several advanced solid tumors, especially breras cancer (345) (346) (347) (348) (349) (350) (351) (352) (353) (**SUPPLEMENTARY TABLE 2**).

**M2698** (MSC2363318A)

M2698, previously known as MSC2363318A, is a potent dual inhibitor of both AKT and S6K1 that induces a remarkable PAM pathway inhibition (354). M2698 displays cancer growth inhibition, in a dose-dependent manner, in mouse xenograft models gained from human HER2-expressing TNBC cell lines with dysregulated PAM pathway. Moreover, M2698 can improve survival, and decrease brain tumor burden in U251 glioblastoma cell orthotopically-implanted mice (355). M2698 demonstrates therapeutic efficiency in several preclinical models of uterine cancer and ovarian cancer, supporting its further clinical development. Indeed, M2698 reduces cancer growth and metastases in murine orthotopic models of uterine cancer and ovarian cancer by decreasing proliferation and angiogenesis and enhancing cell death. Notably, combination of M2698 and paclitaxel in the endometrioid uterine tumor mouse model significantly prolongs the overall survival (356). Clinical studies with favourable safety profile have displayed anticancer activity of M2698 monotherapy in advanced solid tumors, and in combination with either trastuzumab or tamoxifen in advanced breast cancer (357) (354) (**SUPPLEMENTARY TABLE 2**).

**Miransertib** (ARQ-092, MK-7075)

Allosteric pan-AKT inhibitor miransertib inhibits AKT activation by dephosphorylation of its membrane-associated active conformation, as well as by averting its inactive conformation from moving into plasma membrane. In several tumor cell lines miransertib inhibits proliferation but shows most potency in colorectal cancer, leukemia, breast, and endometrial cell lines. Additionally, the efficacy of miransertib is more significant in *PIK3R1*- or *PIK3CA*-mutated tumor cell lines, in comparison to those with wt-*PIK3R1*, or wt-*PIK3CA*, or *PTEN* alterations (317). Combination of miransertib with ARQ 087, a pan-FGFR inhibitor demonstrates synergistic antitumor efficacy in ovarian and endometrial cancer cell lines. Indeed, pathway analysis reveals that this combinatorial strategy augments the suppression of PAM pathway and fibroblast growth factor receptor (FGFR) pathway in these cell lines. Notably, this combination also determines a strong anticancer activity in mouse models with endometrial cancer cells as well as patient-derived tumors (358). Combination of miransertib with mTOR inhibitor rapamycin synergistically decreases cell proliferation in NHLs, including indolent subtype follicular lymphoma, as well as aggressive DLBCL, and primary effusion lymphoma *in vitro*. Furthermore, results obtained *in vivo* are consistent with the *in vitro* studies, suggesting that targeting the PAM pathway with a combination of AKT and mTOR inhibitors can represent a potential therapeutic approach in NHLs (318). Interestingly, in endometrial PDX models harboring mutant *AKT1^E17K^*, miransertib monotherapy shows remarkable anticancer activity. Besides, combination of miransertib with paclitaxel demonstrates a strong enhancement of cancer inhibition in breast cancer models in vivo (317). Additionally, combination of miransertib and sorafenib endows the inhibition of cancer progression in a cirrhotic rat model of hepatocellular carcinoma, suggesting their possible use as combined drug treatment for patients with advanced hepatocellular carcinoma (359).

**MK‑2206** (1032349-93-1)

MK‑2206 is an allosteric AKT1/AKT2/AKT3 inhibitor which exhibits synergism with lapatinib in breast cancer cell lines, and with erlotinib in NSCLC cell lines (360). In preclinical studies, combination of MK-2206 with anti-oestrogens displays synergism in oestrogen-sensitive breast cancer cell lines (361). MK-2206 inhibits AKT phosphorylation and cell proliferation in a dose-related manner in breast cancer cell lines. Moreover, combination of MK-2206 with mTOR inhibitor rapamycin inhibits phosphorylation of AKT and S6, synergistically enhances apoptosis, and reduces proliferation with stronger efficacy, suggesting that this combination exerts higher antitumor activity in breast cancer cells, and possibly results in a convenient approach to treat breast cancer patients (362). MK-2206 enhances apoptosis and reduces proliferation of Panc-1 and MIA PaCa-2 pancreatic cancer cell lines. Furthermore, combination of MK-2206 with gemcitabine increases the cytotoxic efficiency of gemcitabine, and inhibits AKT phosphorylation (363). Additionally, combination of MK-2206 with COX2 inhibitor celecoxib suppresses cell growth more significantly in *PTEN*-deficient mouse embryonic fibroblasts compared to their isogenic counterparts. In line with this, loss of *PTEN* results in enhanced COX2 expression in an AKT-independent manner, and *PTEN* deficiency leads to the transcription of COX2 through upregulation of the KLF5 transcription factor. Therefore, combining MK-2206 with COX2 may represent an effective novel strategy to treat PTEN deficiency-related tumors (364). In a 3D co-culture *in vitro* model with ER+ breast tumor cells and bone marrow mesenchymal stem cells representing disseminated tumors cells in a bone marrow niche, combination of MK-2206 with thioredoxin reductase (TrxR) inhibitor D9 significantly reduces breast cancer cells without changing the survival of bone marrow stromal cells. Moreover, this combination suppresses the formation of new metastases more effectively than tamoxifen in mice with disseminated ER+ human breast cancer, suggesting that this approach may efficiently eliminate disseminated tumors cells, thereby reducing the risk of tumor recurrence (365). Clinical studies with favourable or acceptable safety profile have shown anticancer activity of MK‑2206, both singularly and in combination, in different advanced solid tumors, particularly breast cancer. Besides, triple combination of MK‑2206 with bendamustine and rituximab has exhibited significant antitumor activity in CLL (366) (367) (368) (361) (369) (361) (370) (371) (372) (373) (374) (375) (376) (377) (**SUPPLEMENTARY TABLE 2**).

**Perifosine** (D-21266, KRX-0401, NSC 639966)

Lactic acidosis has been reported to interfere with the efficacy of perifosine to treat colorectal cancer spheroids. In fact, cytotoxicity of perifosine in 3D cultures is reduced in acidic environments at pH levels below 6.7, emphasising that this drug, and possibly other AKT inhibitors in CRC cells, are actively dependent on the effect of pH, which should be taken in consideration in pre-clinical and clinical testing of AKT-targeted tumor therapy (378). Interestingly, combination of perifosine and vitamin D can promote apoptotic and non-apoptotic (paraptotic, necrotic and autophagic) cell death in endometrial cancer cells, suggesting that this combination can represent a preferred regimen for the treatment of endometrial cancer (379). UCHL3 is a deubiquitinase overexpressed in TNBC that is associated with poor prognosis. Biochemically, UCHL3 deubiquitinates RAD51, allowing the recruitment of RAD51 to DNA damage sites and enhancing DNA repair. Thus, UCHL3 overexpression renders cancer cells resistant to chemotherapy- and radiotherapy-induced DNA damage. Therefore, targeting UCHL3 can consequently sensitize TNBC cells to treatment. In line with this, low dose (50 nM) perifosine has been shown to inhibit UCHL3 deubiquitination activity. Moreover, perifosine increases olaparib-induced growth inhibition in TNBC cells. Indeed, perifosine determines RAD51 ubiquitination, thereby blocking the RAD51/BRCA2 interaction, which subsequently reduces ionizing radiation-induced foci (IRIF) of Rad51, and consequently, HR-mediated DNA double strand break (DSB) repair. In addition, perifosine combined with olaparib also displays synergistic anticancer activity in vivo in TNBC xenograft models (380). Besides, perifosine has been found to decrease telomerase activity in 66.6% of chronic lymphocytic leukemia patients, suggesting that perifosine could be used as a novel approach to target telomerase in cancer (381). Clinical studies with favourable or acceptable safety profile have demonstrated anticancer activity of perifosine, both singly and in combination, in solid tumors, and in combination with other drugs in multiple myeloma (382) (383) (384) (385) (386) (**SUPPLEMENTARY TABLE 2**).

**TAS-117** (TAS 117)

TAS-117 is a strong allosteric AKT inhibitor that also downregulates EZH2, a histone-lysine N-methyltransferase enzyme involved in histone methylation, and thus, transcriptional repression of PRC2, a protein complex which cross-talks with downstream PAM signaling pathway, and consequently, represents a key therapeutic target for multiple myeloma. Interestingly, combination of TAS117 with dual EZH1/EZH2 inhibitor UNC1999 synergistically increases TAS-117 anticancer efficacy, resulting in apoptosis in myeloma cells. Besides, TAS-117 treatment induces activation of FOXO3, leading to its nuclear accumulation, and upregulation of its downstream targets, among which are several pro-apoptotic genes. In fact, TAS-117 treatment can cause direct binding of FOXO3 to EZH1 promoter, thereby enhancing FOXO3 shuttling from the cytoplasm into the nucleus (387). TAS117 also demonstrates a remarkable downregulation of p-AKT and growth inhibition in multiple myeloma cell lines. Moreover, TAS117 exerts cytotoxicity in patient-derived multiple myeloma cells, but not in normal peripheral blood mononuclear cells (PBMCs). Additionally, TAS-117 enhances bortezomib-induced apoptotic signaling and endoplasmic reticulum stress in multiple myeloma cells, and improves bortezomib-induced cytotoxicity *in vivo*, leading to prolonged host survival. Besides, combination of TAS-117 with carfilzomib also determines increased cytotoxicity towards multiple myeloma cells, compared to either singular drug treatment. Furthermore, oral administration of TAS-117 significantly suppresses human multiple myeloma cell growth, enhances apoptosis, and promotes endoplasmic reticulum stress response in murine xenograft models. Likewise, TAS-117 improves bortezomib-induced cytotoxicity *in vivo*, leading to prolonged host survival (388). A clinical study with acceptable safety profile has shown limited anticancer activity of TAS-117 monotherapy in PI3K-mutated and/or AKT-mutated solid tumors refractory to chemotherapy (389) (**SUPPLEMENTARY TABLE 2**).

**Uprosertib** (GSK2141795)

Uprosertib is potent ATP-competitive AKT inhibitor, which acts equally on AKT1, AKT2 and AKT3 isoforms. Interestingly, lactic acidosis enhances resistance of colon cancer cells to uprosertib, mainly caused by reduction of apoptosis and increased of cell survival. Notably, inhibition of lactate transport or oxidative metabolism reverses this lactic acidosis-induced resistance, thereby enhancing the efficacy of uprosertib treatment on colon cancer cells (390). Uprosertib has also been found to inhibit cancer cell proliferation and metastasis, as well as cytokine regulation and PD-L1 expression. As a result, since AKT is involved in the regulation of breast tumor progression and immuno-suppression, uprosertib is currently undergoing clinical investigation to improve treatment in TNBC patients (343). A clinical study with favourable safety profile have displayed efficacious anticancer activity of uprosertib monotherapy in advanced solid tumors (391) (**SUPPLEMENTARY TABLE 2**).

**mTOR INHIBITORS**

mTOR inhibitors were the first PAM-targeting drugs to advance to the clinic (392). Induction of mTORC1 enhances the formation of proteins, lipids, nucleotides, and decreases autophagy, resulting in cell survival, proliferation, and growth; whereas activation of mTORC2 regulates protein kinases, including AKT, leading to cell survival, and proliferation (393). Therefore, both mTORC1 and mTORC2 functions provide an important rationale for targeting mTOR complexes in tumor, although the effectiveness of some mTORC inhibitors may be compromised by a compensatory feedback loop leading to AKT activation (355). Only two mTOR inhibitors (temsirolimus and everolimus) have been approved by the FDA for cancer treatment (**SUPPLEMENTARY FIGURE 1**). There are three types of mTOR inhibitors: allosteric (non-competitive) mTOR inhibitors (allosteric mTORi), which inhibit mTORC1 only; ATP-competitive mTOR inhibitors (ATP-competitive mTORi), which suppress both mTORC1 and mTORC2; and bi-steric mTOR inhibitors (bi-steric mTORi), which inhibit mTORC1 only (**FIGURE 10**).

**ALLOSTERIC mTOR INHIBITORS**

Allosteric (non-competitive) mTORi act against mTORC1. Since allosteric inhibitors can only exert their function towards mTORC1, they cannot avoid the feedback loop-based induction of AKT determined by the suppression of mTORC1. Besides, allosteric mTORi can modestly reduce p4E-BP1 levels through the inhibition of (4E-BP1) phosphorylation, and consequently, cannot effectively restrain eIF4E-mediated cap-dependent translation initiation in cancer (394). Therefore, these agents exert a weaker PAM signalling inhibition compared to ATP-competitive mTORi, resulting in decreased antitumor activity. The first generation of allosteric mTOR inhibitors includes rapamycin and its analogues, commonly known as rapalogs, which only exert a specifical inhibition towards mTORC1 (395). Herein, we included the major allosteric mTORi that are currently in different stages of human clinical trials. A summary of the most representative studies on these inhibitors is shown in **SUPPLEMENTARY TABLE 3**.

**Everolimus** (RAD001, SDZ-RAD)

Combination of everolimus with metformin results in more significant additive effects, in terms of colony reduction and antiproliferation, when compared to everolimus singly. Mechanistically, this co-treatment successfully inhibits mTOR signaling and mitochondrial respiration in breast cancer cells, suggesting its further *in vivo* investigation as antitumor therapy (396). Combination of everolimus with VEGFR/RET/EGFR inhibitor vandetanib, displays synergistic anticancer effects in ACVR1-mutant diffuse intrinsic pontine glioma cells. In fact, apart from mTOR, everolimus suppresses ABCB1 and ABCG2, whereas vandetanib specifically targets ACVR1. Additionally, this combined treatment is well tolerated *in vivo*, due to its effect on increasing survival and reducing cancer burden, as observed in an orthotopic ACVR1-mutant patient-derived diffuse intrinsic pontine glioma xenograft model (397). Everolimus has exhibited remarkable clinical benefits in several types of cancers. Accordingly, everolimus has been FDA-approved as monotherapy in NET patients, TSC-associated subependymal giant cell astrocytoma (SEGA) patients, renal TSC-associated angiomyolipoma adult patients, and advanced-stage renal cell carcinoma (RCC) patients, as well as in combination with lenvatinib in advanced-stage RCC patients who have received a prior antiangiogenic therapy, and in combination with exemestane in postmenopausal HR^+^/HER2^­^ breast cancer patients with recurrence or progression following prior therapy with letrozole or anastrozole (398) (399) (**SUPPLEMENTARY FIGURE 1**). Clinical studies with favourable or acceptable safety profile have shown anticancer activity of everolimus, both singularly and in combination, in a variety of advanced solid tumors. In particular, double or triple combination of everolimus with other drugs has exhibited antitumor activity in breast cancer (400) (96) (401) (402) (403) (404) (405) (399) (406) (407) (408) (409) (410) (411) (412) (413) (414) (415) (416) (417) (418) (419) (**SUPPLEMENTARY TABLE 3**).

**Nab-sirolimus** (Nab-rapamycin, ABI-009)

Preclinical studies have shown that nanoparticle albumin-bound (nab)-sirolimus is a potent drug with a favorable safety profile. Indeed, nab-sirolimus significantly decreases cell viability and reduces downstream signaling in numerous xenograft tumor models, such as colorectal (420), and multiple myeloma (421). Moreover, in human breast xenograft models nab-sirolimus determines 75% inhibition of cancer growth, and its anticancer activity is further improved when given in combination with doxorubicin (a topoisomerase inhibitor), perifosine (an AKT inhibitor), SAHA (an HDAC inhibitor), and erlotinib (an EGFR inhibitor), with high tolerability in the combination regimen (422). Clinical studies with favourable or acceptable safety profile have demonstrated anticancer activity of nab-sirolimus monotherapy, in advanced non-hematological malignancies (423) (424) (**SUPPLEMENTARY TABLE 3**).

**Rapamycin** (Sirolimus, AY-22989, I-2190A)

Rapamycin, also known as sirolimus, is a natural macrolide compound produced by the bacterium Streptomyces hygroscopicus, and has immunosuppressive and antiproliferative properties (425). Generally, rapamycin monotherapy shows no remarkable cytotoxicity towards tumor cells (426). In cells, rapamycin interacts with the intracellular immunophilin FKBP12 to generate an immunosuppressive complex that binds to and blocks the activation of mTOR. In fact, this immunosuppressive complex inhibits the interaction of mTOR with Raptor, thereby disrupting the coupling of mTORC1 with its substrates. Therefore, this natural compound is a specific allosteric inhibitor of mTORC1 (427). Moreover, rapamycin treatment in combination with chemotherapeutic agents significantly induces apoptosis in various tumor cell lines (428). Combination of rapamycin with ALK/IGF1R inhibitor AZD3463 significantly reduces survival, enhances apoptosis, and increases the G0/G1 phase of the cell cycle arrest, compared to treatment using individual agent, in MCF7 breast cancer cells. Additionally, this combination displays a decrease of *PRKCB* and *PIK3CG* gene expression (429). A recent nanomedicine study has reported that cellular uptake of rapamycin liposomes (particle size of 100 ± 5.5 nm) by colorectal cancer cells is remarkably higher compared to free rapamycin, without liposomes. Indeed, the capability of rapamycin liposomes to suppress cancer proliferation and migration, as well as enhance apoptosis in cancer cells, is superior compared to free natural rapamycin. In addition, *in vivo* studies have shown that rapamycin liposomes lead to a greater increase in tumor compared to free natural rapamycin, and consequently, display stronger anticancer efficacy in HCT-116 xenograft mice. Notably, liposome-delivered rapamycin more significantly augments the chemotherapy efficacy of 5-fluorouracil in CRC, both *in vitro* and *in vivo*, in comparison to free natural rapamycin combined with 5-fluorouracil, emphasising the potential of liposome-based delivery features in future clinical studies (430). Combination of rapamycin with MEK inhibitor trametinib results in a robust synergistic anticancer effect, leading to apoptosis, and G1-phase arrest of the cell cycle in NSCLC cells. Additionally, this combination, significantly suppresses phosphorylation of AKT, mTOR, ERK, and 4E-BP1. Moreover, co-administration of rapamycin and trametinib exhibits a decrease in cancer growth without displaying drug toxicity in a NSCLC xenograft mouse model (431). Importantly, since rapamycin itself do not possess ideal pharmacological properties, novel drugs of rapamycin analogs, commonly known as rapalogs, with improved pharmacokinetic properties and decreased immunosuppressive effect, have been developed during the last decades (432). In particular, as its interactions with both mTOR and FKBP12 proteins have been preserved, rapalogs compounds share the same mechanism of action as rapamycin (433). Nevertheless, clinical studies with favourable or acceptable safety profile have shown anticancer activity of rapamycin, both singly and in combination, in several advanced solid tumors (434) (435) (435) (**SUPPLEMENTARY TABLE 3**).

**Ridaforolimus** (AP23573, MK-8669, Deforolimus)

TRIM28 is the transcriptional activator of the mutant TERT promoter in bladder cancer and is inhibited upon interaction with TRIM24. Mechanistically, mTORC1-mediated phosphorylation of TRIM28 releases it from TRIM24, thereby inducing hTERT transcription, and promoting cell growth of bladder cancer. Interestingly, ridaforolimus significantly reduces TRIM28 phosphorylation, hTERT expression, and cell viability, in bladder cancer, both *in vitro* and *in vivo* (436). Moreover, triple combination of ridaforolimus, with IGF1R inhibitor dalotuzumab, and aromatase inhibitor letrozole, shows synergism in ER^+^ hormone-sensitive breast cancer, by significantly suppressing xenograft cancer growth (437). Clinical studies with favourable or acceptable safety profile have displayed anticancer activity of ridaforolimus, both singly and in combination, in different advanced solid tumors (438) (439) (440) (441) (442) (443) (444) (445) (**SUPPLEMENTARY TABLE 3**).

**Temsirolimus** (CCI-779, Torisel)

Temsirolimus, an ester of sirolimus (rapamycin), is an intravenous-administered drug that establishes a complex with FKBP1A, which is then integrated into mTORC1, but not mTORC2, thereby inhibiting mTOR (446), and consequently suppressing the generation of proteins implicated in cell cycle (447) and angiogenesis (448). Temsirolimus significantly reduces survival, growth, and migration of retinoblastoma cells without interfering with normal retinal cells, both *in vitro* and in xenograft mouse models. Additionally, temsirolimus remarkably decreases retinal angiogenesis through targeting retinal endothelial cell biological functions. Notably, temsirolimus-induced inhibition of retinoblastoma and angiogenesis is achieved by suppressing mTOR signalling and proangiogenic cytokines (449). Combination of temsirolimus and MEK inhibitor trametinib can radiosensitize A549 NSCLC cells by reducing clonogenic survival, increasing apoptosis, and prolonging γ-H2AX expression, as well as G2/M cell cycle arrest. Moreover, *in vivo* studies have demonstrated that co-treatment of these two agents significantly sensitize lung cancer xenografts to radiotherapy (450). Temsirolimus has shown significant clinical benefits as monotherapy in RCC patients, and thus has been FDA-approved for use in this cohort (451) (**SUPPLEMENTARY FIGURE 1**). Clinical studies with acceptable safety profile have exhibited anticancer activity of temsirolimus, both singly and in combination, in advanced solid tumors, and hematological malignancies (383) (452) (453) (454) (455) (456) (457) (458) (392) (459) (**SUPPLEMENTARY TABLE 3**).

**ATP-COMPETITIVE mTOR INHIBITORS**

ATP-competitive mTORi, also known as active-site mTORi, act against both mTORC1 and mTORC2, avoiding the feedback loop-based induction of AKT determined by the suppression of mTORC1. Notably, ATP-competitive mTORi remarkably reduce p4E-BP1 levels through the inhibition of (4E-BP1) phosphorylation, and thus, can effectively avoid eIF4E-mediated cap-dependent translation initiation in cancer. Thence, ATP-competitive mTORi actively promote dephosphorylation on Thr46 (4E-BP1), thereby re-establishing its endogenous functions of growth suppression and pro-apoptosis (394). As a result, these inhibitors may induce a stronger PAM signalling inhibition, compared to allosteric mTOR inhibitors, leading to enhanced antitumor activity (395). This difference is mainly due to the fact that allosteric mTORi, which represent the first-generation mTORi, suppress mTORC1 indirectly by binding to FKBP12; while ATP-competitive mTORi, which represents the second-generation mTORi, suppress both mTORC1 and mTORC2 by inhibiting the mTOR kinase directly (460). In fact, in regard to allosteric mTORi, there is a catalytic cleft within the FKBP12-rapamycin-binding (FRB) domain of mTOR, enabling limited access to 4E-BP1 as a substrate; whereas ATP-competitive mTORi are able to bind deeper inside the catalytic cleft, thereby abolishing the capacity to phosphorylate 4E-BP1 (394). Herein, we included the major ATP-competitive mTORi currently in different stages of human clinical trials. A summary of the most representative studies on these inhibitors is shown in **SUPPLEMENTARY TABLE 3**.

**AZD8055** (R339J08R6U)

An *in vitro* study reported that AZD8055 exerts strong efficacy against the proliferation of MCF-7/R cells due to down-regulation of HSPB8 expression levels in ER^+^ breast cancer cells. Interestingly, AZD8055 overcomes tamoxifen resistance in breast cancer cells by inducing significant anti-proliferative activity and G1-phase arrest of the cell cycle. Therefore, the efficacy of AZD8055 in ER^+^ breast cancer is pertinent to the expression of HSPB8, suggesting that this feature merits further investigation (461). AZD8055 significantly increases the inhibition of radiation-induced cell growth, colony formation, G2/M arrest, and enhances apoptosis, in nasopharyngeal carcinoma cell lines. Therefore, AZD8055 can also be considered a radiosensitizer for nasopharyngeal carcinoma (462). AZD8055 also exerts cell cycle blockage, enhances apoptosis, and induce cytotoxicity in colon cancer cells. In addition, AZD8055 exhibits anticancer efficacy in mice. Besides, AZD8055 reduces the overall mTOR signaling pathway, both *in vitro* and *in vivo* (463). Dual mTORC1/mTORC2 inhibition with AZD8055 blocks AKT/mTOR signalling, suppresses cancer cell viability, partly due to cell cycle arrest, and reduces tumor growth in mouse xenograft model more significantly compared to mTORC1-only inhibition in TFE3-fusion RCC (464). AZD8055 partially modulates its antitumor effect by PUMA-dependent apoptosis through the intrinsic mitochondrial pathway in colorectal cancer cells. Additionally, AZD8055 significantly suppresses CRC tumor growth in mice. Accordingly, deletion of PUMA enhances resistance towards AZD8055, suggesting that PUMA mediates tumorigenesis both *in vitro* and *in vivo* (465). Recent studies have shown that AZD8055 synergistically enhances the antitumor efficacy of EGFR inhibitor afatinib by reducing cell viability in chordoma cell lines. Also, this drug combination thoroughly inhibits cancer growth *in vivo*, displaying a significant improvement of tumor control, suggesting a novel therapeutic strategy for chordoma patients (466). Clinical studies with acceptable safety profile have demonstrated anticancer activity of AZD8055 monotherapy in a variety of advanced solid tumors (467) (468) (**SUPPLEMENTARY TABLE 3**).

**Onatasertib** (CC-223, ATG-008)

Onatasertib exhibits antitumor efficacy toward pancreatic cancer cells by inhibiting mTOR signaling. Indeed, onatasertib impedes Sestrin2 overexpression-induced enhancement of glycolysis, which promotes tumor development through mTOR pathway in pancreatic cancer cells. In fact, onatasertib exerts cytotoxic effects, detected by reduced proliferation, invasion and migration, against pancreatic cancer cells (208). Onatasertib abolishes mTORC1/mTORC2 activation and induces remarkable anti-proliferative and cytotoxic activities against established human hepatocellular carcinoma cell lines, as well as primary human hepatocellular carcinoma cells. Importantly, onatasertib induces apoptosis, activation of caspase-3/caspase-9, enhancement of mitochondrial permeability transition pore opening, and augmentation of ROS production in several hepatocellular carcinoma cell types (469). Onatasertib significantly inhibits survival, growth, proliferation and cell cycle advancement in primary human oral cavity carcinoma cells, and causes activation of apoptosis in human HNSCC cells, emphasising that onatasertib-targeted mTOR kinase inhibits human HNSCC cell growth *in vitro*. Besides, oral administration of onatasertib significantly reduces HNSCC SCC-9 xenograft tumor growth in severe immunodeficient mice. Moreover, activation of both mTORC1 and mTORC2 is remarkably decreased in onatasertib-treated cancer tissues. Thus, onatasertib can be considered a potential valuable treatment option for HNSCC (470). Additionally, oral administration of onatasertib strongly reduces human hepatocellular carcinoma xenograft growth, and significantly blocks mTORC1/mTORC2 activation, in severe immunodeficient mice (469). Clinical studies with acceptable safety profile have shown anticancer activity of onatasertib monotherapy in several advanced solid tumors (471) (472) (**SUPPLEMENTARY TABLE 3**).

**OSI‑027** (ASP4786, CERC 006, AEVI-006)

Catastrophic macropinocytosis is a process whereby several water-filled vacuoles form, merge, and ultimately rupture, thereby killing the cell. Interestingly, a study has reported that OSI-027 can induce cancer catastrophic macropinocytosis in breast cancer, skin cancer, lung cancer, cervix cancer, and rhabdomyosarcoma cells. Importantly, OSI‑027-mediated inhibition of mTORC1/mTORC2 is confirmed as the underpinning mechanism for macropinocytosis (473). OSI-027 demonstrates significant antitumor activity by increasing PUMA-dependent apoptosis through the intrinsic mitochondrial pathway in colorectal cancer (465). In line with this, OSI-027 suppresses the oncogenesis of colon cancer by mediating the c-Myc/FOXO3a/PUMA axis. Indeed, OSI-027 decreases colon cancer cell survival dose-dependently by activating cell apoptosis. Moreover, OSI-027 promotes apoptosis of colon cancer cells by upregulating PUMA, via the activation of FOXO3a (474). OSI-027 decreases proliferation of MKN-45 and AGS gastric cancer cells by arresting the G0/G1 phase of the cell cycle. Moreover, OSI-027 inhibits mTORC1/mTORC2 activation, reduces p-AKT, p-p70S6K, p-4EBP1, and suppresses cyclin D1 and CDK4. Interestingly, OSI-027 displays synergistic cytotoxicity in combination with oxaliplatin. Indeed, OSI-027 downregulates P-gp, resulting in more significant oxaliplatin-induced apoptosis, and consequently, decreases multidrug resistance (475). OSI-027 significantly inhibits proliferation, suppresses cell cycle in G0/G1 phase, and downregulates mTORC1, mTORC2, p-AKT, p-S6K, p-4EBP1, cyclin D1, and CDK4, in BxPC-3, CFPAC-1, and Panc-1 pancreatic ductal adenocarcinoma cell lines. Furthermore, OSI-027 augments gemcitabine-induced apoptosis in the aforementioned cell lines. Additionally, OSI-027 in combination with gemcitabine exerts synergistic anticancer efficacy *in vitro* and *in vivo* in pancreatic ductal adenocarcinoma (476). OSI-027 decreases rhabdomyosarcoma tumor growth in a murine xenograft model. Also, in the residual xenograft tumors, OSI-027 treatment results in lower expression of cell survival and proliferation markers, as well as proteins involved in the EMT, emphasising that dual mTORC1/mTORC2 inhibition has the potential to be used for the treatment of recurrent or refractory rhabdomyosarcoma (473). Moreover, co-administration of OSI-027 with IAP antagonist AT406, significantly potentiates the suppression of HepG2 tumor growth in hepatocellular carcinoma nude mice (477). A clinical study with acceptable safety profile has displayed only moderate anticancer activity of OSI‑027 monotherapy in advanced solid tumors, and advanced lymphomas (478) (**SUPPLEMENTARY TABLE 3**).

**Sapanisertib** (TAK-228, MLN0128, INK128)

Sapanisertib determines a dose-dependent decrease in cell proliferation, associated with reduced levels of p-RPS6^S235/236^ and p-4EBP1^T37/46^ in glioma cells (479). Moreover, combination of sapanisertib with trastuzumab displays a more significant antitumor efficacy in reversing the acquired resistance, compared to each agent singly, in several HER2^+^ breast cancer cell lines with trastuzumab resistance. Indeed, this combined treatment remarkably enhances apoptosis, and augments cell cycle arrest in G0/G1 phase, in most cell lines (480). Combination of sapanisertib with MEK inhibitor trametinib synergistically decreases cell survival, induces apoptosis, and induces cell cycle arrest, in canine mucosal melanomas cell lines, compared to monotherapy with each drug. Additionally, sapanisertib combined with daily trametinib can reduce primary mucosal melanoma xenograft growth in mice, and cancer dissemination in a metastasis model, minimizing renal and hematologic side effects. Moreover, this combination downregulates respective signaling targets and abolishes pathway reciprocal crosstalk, suggesting that targeting PAM and Ras/MAPK signal transduction axes could be a rational strategy for treating human mucosal melanomas (481). Sapanisertib has reached clinical testing in a variety of tumours and in several types of combinations. Particularly, sapanisertib is currently administered to patients with advanced-stage cancers harbouring *TSC1* or *TSC2* mutations (482). Clinical studies with favourable or acceptable safety profile have shown anticancer activity of sapanisertib, both singly and in combination, in different advanced solid tumors. Besides, a clinical study with acceptable safety profile displayed significant efficacy of sapanisertib monotherapy in hematological malignancies (483) (484) (485) (486) (486) (487) (114) (488) (489) (490) (491) (**SUPPLEMENTARY TABLE 3**).

**Torkinib** (PP242)

Torkinib is a specific mTORC1 and mTORC2 inhibitor (492), although an *in vitro* study has also reported that torkinib can inhibit PKCα, PKCβ, JAK2, and RET (493). Notably, torkinib suppresses proliferation of primary cells more efficiently compared to rapamycin. Interestingly, mTORC2 itself is not the basis for this increased activity; in fact, torkinib is rather unexpectedly a more efficient mTORC1 inhibitor than rapamycin. Indeed, proliferative ability of *Sin1^−/−^* mouse embryonic fibroblasts is more sensitive to torkinib compared to rapamycin. This highlights that rapamycin-resistant functions of mTORC1, such as the translation initiation, are crucial for the antiproliferative effects of torkinib. Besides, suppression of translational control and the antiproliferative effects of torkinib requires the inhibition of 4E-BP1 phosphorylation and eIF4E activity (492). However, in multiple myeloma cell lines, the main anticancer activity of torkinib is due to its additional inhibitory effects on mTORC2 (494). Torkinib significantly reduces cell proliferation, metastasis, and angiogenesis through inhibition of the PAM signaling pathway in human endothelial tumor cells and gastric cancer cells (495). In addition, torkinib counteracts proliferation, migration, invasiveness and stemness properties through the suppression of mTORC2/AKT in glioblastoma cell lines (496). Moreover, torkinib significantly reduces bladder cancer cell proliferation and migration through deactivation of the mTORC2/AKT1 signaling pathway. Furthermore, torkinib potently decreases the phosphorylation of AKT1 and mTORC2 (497). Activation of the PAM pathway and overexpression of histone deacetylases are common alterations in ovarian cancer. Interestingly, combination of torkinib with histone deacetylase inhibitor suberoylanilide hydroxamic acid synergistically determines an inhibition of growth, an enhancement of apoptosis, and an increase of autophagy, compared to each mono-agent therapy in ovarian cancer cells. Additionally, this combination displays stronger inhibition on cancer growth, and remarkably prolonged the survival time of mice, compared to each treatment singly (498). Combination of torkinib with PDK1 inhibitor GSK2334470 shows stronger anticancer efficacy compared to torkinib monotherapy *in vitro*, in multiple myeloma xenograft models, and in immunodeficient mice (499). Recent metabolic studies have shown that a 3-week torkinib treatment exhibits a reduction in cancer size and weight without any serious toxicity. Metabolic changes because of torkinib are not significant in plasma. Conversely, metabolic changes in cancer tissues are significant in torkinib-treated xenografts compared to untreated xenografts, empasising that energy and lipid metabolism are mainly altered by torkinib (500). Torkinib significantly reduces proliferation and induces apoptosis in glioblastoma cells. Interestingly, this increase in apoptosis results not only from torkinib-induced autophagy through on-target suppression of mTOR, but also, from off-target co-operative inhibition of PKCα and JAK2. Accordingly, a triple combination of torkinib with either EGFR inhibitors osimertinib or erlotinib, which can block PKCα, plus JAK2 inhibitor AZD1480, is able to significantly induce apoptosis in glioblastoma tumors in patient-derived orthotopic xenograft models, suggesting that this co-administration treatment can represent a valuable preclinical rationale to test analogous combinations in glioblastoma patients (479). Combination of torkinib with anti-EGFR monoclonal antibody cetuximab exerts significant anticancer effects by inhibiting proliferation and enhancing apoptosis, as well as cell cycle arrest, in CaCo-2 and HT-29 CRC cell lines. Besides, this dual-targeted molecular therapy inhibits tumor growth in mouse xenograft models of colorectal cancer (501). These studies strongly suggest that this promising new drug can complement rapamycin in elucidating the role in mTOR in human cancer.

**Vistusertib** (AZD2014)

Vistusertib is a mTORC1/mTORC2 inhibitor currently being investigated in several phase 1 and phase 2 trials, especially in meningioma, glioblastoma, and prostate cancer patients, as well as in patients with tumours harbouring *TSC* mutations, and tumors harbouring *RICTOR* amplifications, which are known to lead to mTORC1 and/or mTORC2 hyperactivation (502). *In vitro*, vistusertib can radiosensitize oral squamous cell carcinoma cells by inhibiting PAM signaling, and inducing cell cycle arrest in G1/G2/M phases (503). Besides, vistusertib increases the radiosensitivity of glioblastoma stem-like cells, both in vitro and in vivo, suggesting that this mechanism involves an inhibition of DNA repair (504). Vistusertib exerts stronger anticancer activity, due to inhibition of proliferation, enhancement of apoptosis, and suppression of G1 phase arrest of the cell cycle, compared to rapamycin monotherapy, in both docetaxel-sensitive and docetaxel-resistant castration resistant prostate cancer (CRPC) cells. Moreover, vistusertib more significantly inhibits AKT phosphorylation, as well as 4E-BP1 phosphorylation, and reduces migration, invasion and EMT, compared to rapamycin singly (505). Vistusertib treatment remarkably inhibits mTOR signaling, as well as proliferation, and enhances apoptosis, along with G1-phase arrest of cell cycle, in ovarian cancer cells. Also, vistusertib limits cancer growth and prolongs survival in tumor-bearing mice. Notably, vistusertib specifically decreases myeloid-derived suppressor cell migration and accumulation in epithelial ovarian cancer peritoneal fluid, but not in the spleen. In addition, subsequent vistusertib treatment after cisplatin chemotherapy significantly delays epithelial ovarian cancer recurrence (506). Combination of vistusertib with paclitaxel exerts additive effects, in term of decreased cell growth, in numerous cancer cell lines. Interestingly, in cisplatin-resistant cells, combination of vistusertib with paclitaxel decreases p-S6 and p-AKT levels. Likewise, this treatment combination remarkably reduces cancer volume in cisplatin-resistant xenograft models compared to vistusertib or paclitaxel singly. Furthermore, there is a significant enhancement of apoptosis *in vivo* due to high levels of cleaved PARP. Besides, treatment of ovarian cancer patients with paclitaxel induces an increase in p-AKT levels in platelet-rich plasma, but interestingly, co-treatment with vistusertib significantly abrogates the p-AKT increase, emphasising that this combination can represent a valuable therapy (507). Clinical studies with favourable or acceptable safety profile have demonstrated anticancer activity of vistusertib, in combination with other drugs, in several advanced solid tumors. In addition, a clinical study with acceptable safety profile exhibited limited efficacy of vistusertib monotherapy in diffuse large B-cell lymphoma (508) (509) (510) (511) (512) (**SUPPLEMENTARY TABLE 3**).

**BI-STERIC mTOR INHIBITORS**

Induction of mTORC1 triggers protein synthesis through phosphorylation of 4EBP proteins, relieving their suppression of cap-dependent translation. First-generation mTORi rapalogs bind to the immunophilin FKBP12, and subsequently, this rapalog/FKBP12 complex binds to the FRB domain of mTOR, thereby allosterically inhibiting phosphorylation of the substrate S6K. However, this rapalog/FKBP12 complex bound to the FRB domain of mTOR suppresses modestly 4EBP1 phosphorylation, and thus, determines minor effects on mTORC. As a result, rapalogs have shown only moderate clinical anticancer activity. Consequently, to tackle these issues, second-generation ATP-competitive mTORi, which robustly suppress both mTORC1 and mTORC2, were produced; however their clinical efficacy still remains minimal. Thus, these first and second-generation mTORi have not yielded a clear therapeutic advantage at tolerated doses. In addition, the clinical benefits of rapalogs and ATP-competitive mTORi have been obstacled by toxicity. Hence, a third-generation mTORi named bi-steric mTORi (also known as RapaLinks) that selectively inhibit mTORC1 and not mTORC2 has recently been designed (513). These inhibitors, which contain a rapamycin-like core moiety covalently linked to an mTOR active-site inhibitor (514), are termed bi-steric due to their simultaneous engagement of the allosteric FRB domain and orthosteric catalytic domain of mTOR in order to deepen the suppression of mTORC1 while also retaining selectivity for mTORC1 over mTORC2 (513). Importantly, bi-steric mTORi have demonstrated potent and selective inhibition of 4E-BP1 phosphorylation leading to reduced cell proliferation *in vitro* and tumor regression *in vivo*. Moreover, these compounds cause less relief of AKT-dependent feedback inhibition of RTK expression, which notoriously results in RTK receptor reactivation-induced adaptive resistance, and toxicity in comparison to ATP-competitive mTORi. Also, bi-steric mTORi display a longer dwell-time on target compared to other types of mTORi, and thus can be regularly dosed intermittently (513) (515). Herein, we included the major bi-steric mTORi currently used in pre-clincial studies and in human clinical trials. A summary of the most representative studies on these inhibitors is shown in **SUPPLEMENTARY TABLE 3**.

**RMC-4627**

Bi-steric mTORi RMC-4627 consists of a rapamycin monomer covalently linked to the mTOR active-site inhibitor PP242 (513). RMC-4627 has shown significant anticancer activity both *in vitro* and *in vivo*. Indeed, RMC-4627 potently and selectively inhibits 4E-BP1 phosphorylation in B-cell acute lymphoblastic leukemia cell lines, without suppressing mTORC2 activity. RMC-4627 effectively reduces cell survival, decreases cell cycle progression, enhances apoptosis, and augments cytotoxicity of tyrosine kinase inhibitor dasatinib in these B-cell acute lymphoblastic leukemia cell lines. Besides, RMC-4627 has a longer dwell-time on target compared to other types of mTORi, and as a result can be conveniently dosed intermittently (515). This prolonged duration of action can be attributed to binding of the rapamycin moiety to abundant cellular FKBP12 (514). Moreover, once/weekly intraperitoneal administration of RMC-4627 decreases leukemic burden and augments the anticancer activity of dasatinib in a B-cell acute lymphoblastic leukemia xenograft model. Notably, RMC-4627 treatment is well tolerated in these animal models. These results suggest that intermittent dosing of RMC-4627 or other bi-steric mTORi has therapeutic potential as a component of leukemia regimens (515).

**RMC-5552**

The bi-steric mTORC1-selective inhibitor RMC-5552 is a potent activator of 4EBP1, thereby suppressing initiation of protein translation. This novel therapeutic moiety tackles a significant impediment of rapalogs, which cannot properly inhibit phosphorylation of 4EBP1. RMC-5552 displays remarkable anticancer activity in human xenograft model of *PIK3CA*-mutated breast cancer in mice (516). Mutations in Ras genes can frequently co-occur with aberrant of mTORC1, hampering the anticancer activity of targeted Ras inhibitors. Interestingly, combination of RMC-5552 and Ras inhibitors show a stronger anticancer activity in a series of pre-clinical models of *KRAS*-mutated NSCLC compared to each agent singly. This suggests that mTORC1 induction contribute to resistance to *KRAS* inhibitors, which can be overcome by combination with RMC-5552. A recent phase 1/1b clinical study with favourable safety profile has exhibited anticancer activity of RMC-5552 in combination with Ras inhibitors in relapsed or refractory Ras-mutated solid tumor patients. At doses of 6 mg and higher the overall response rate (ORR) is 20% and the stable disease (SD) is 60%. The dose of 6 mg/weekly of RMC-5552 administered intravenously is well tolerated. The most frequent (> 25%) adverse events are stomatitis/mucositis (43%) and reduced appetite (29%), whereas the the most frequent dose-limiting grade 3 adverse events are stomatitis/mucositis observed in dose levels ≥ 10 mg (21%) (517) (**SUPPLEMENTARY TABLE 3**).

**RMC-6272**

Bi-steric mTORC1-selective inhibitor RMC-6272 is another robust activator of 4EBP1. Indeed, administration of a single intraperitoneal injection of RMC-6272 displays a dose-dependent, potent, and durable inhibition of p4EBP1 levels in human breast cancer cell line-derived xenograft models in mice. As previously mentioned, abnormal induction of mTORC1 can also co-occur with mutations in Ras genes, hindering the anticancer activity of targeted Ras inhibitors. In line with this, combination of RMC-6272 and *KRAS^G12C^* inhibitor sotorasib determines a stronger apoptosis and cancer regression in a xenograft model of *KRAS^G12C^* mutant NSCLC bearing a loss-of-function mutation in *STK11*, compared to sotorasib singly, emphasising that mTORC1 induction contribute to resistance to *KRAS^G12C^* inhibitors, which can be overcome by combination with RMC-6272. Besides, RMC-6272 exhibits an acceptable tolerability profile in these animal models (516).

**PDK1 INHIBITORS**

PDK1, also known as PDPK1, is a crucial regulator of PAM signalling pathway due to its phosphorylation on AKT. Indeed, PDK1 can exert a potential role in developping chemoresistance in various types of malignancy (518). Thus, it is reasonable to suggest that PDK1 inhibition, singly or in combination with other PAM inhibitors, could contribute to the enhancement of antitumor efficacy in different types of human cancer. The major PDK1 inhibitors are shown in **FIGURE 10**.

**GSK2334470** (GSK-470)

PDGSK2334470, a novel and specific small molecule PDK1 inhibitor, remarkably decreases cancer-specific cell growth and synergizes docetaxel sensitivity in DU145 and PC3 prostate cancer cells. This study emphasises that PDK1 mediates cell survival through SGK3 signalling and suggests that inactivation of the PDK1/SGK3 axis merits consideration for future treatment of prostate cancer (519). GSK2334470 significantly induces antiproliferative effects in mantle cell lymphoma-derived cell lines. This inhibition of cell growth is induced by enhanced apoptosis, and G2/M-phase arrest of the cell cycle. Notably, suppression of PDK1 displays no effects on AKT, but conversely, results in the inactivation of effector kinase RSK2, highlighting the importance of the PDK1/RSK2 axis in the survival and proliferation of mantle cell lymphoma cells (520). GSK2334470 potently determines cytotoxicity in multiple myeloma cell lines, but not in normal human cells. GSK2334470 remarkably downregulates phosphorylation of AKT at Thr308, and consequently inhibits downstream mTORC1 activity. Interestingly, short hairpin RNA (shRNA)-induced *PTEN* knockout leads to partial reversion of GSK2334470-mediated cell growth inhibition. Conversely, PTEN overexpression strongly increases myeloma cell sensitivity to GSK2334470, emphasising that sensitivity to GSK2334470 is closely associated with the expression of PTEN in multiple myeloma cells. Moreover, combination of GSK2334470 with dual mTORC1/mTORC2 inhibitor torkinib shows stronger anticancer efficacy compared to mono-agent therapies, both *in vitro* and in multiple myeloma xenograft models, in immunodeficient mice (499). Accordingly, GSK2334470 suppresses growth of multiple myeloma cell lines and induces apoptosis associated with the induction of intrinsic mitochondrial pathway, as well as extrinsic death receptor pathway. Furthermore, combination of GSK2334470 with proteasome inhibitor MG‑132 shows synergistic antitumor effects, by further reducing cell growth and proliferation. Importantly, this combination, almost completely inhibits phosphorylation of AKT, mTORC1, and mTORC2. Additionally, this drug combination upregulates PTEN, resulting in decreased AKT downstream signaling in PAM pathway. These results emphasise the importance of co-inhibition of PDK1 and proteasome to improve outcomes in multiple myeloma patients (521). Combination of GSK2334470 with CDK4/CDK6 inhibitor ribociclib, synergistically reduces proliferation and enhances apoptosis in several ER^+^ breast cancer cell lines. It is demonstrated that resistant cells display upregulated expression of cyclin A, cyclin E, and cyclin D1, as well as activated phosphorylation of AKT at S477/T479; and conversely, GSK2334470 treatment is capable of reversing these reactions, thereby restoring the sensitivity of cells to ribociclib. Moreover, GSK2334470 combined with ribociclib, suppresses xenograft cancer growth more efficiently than each agent singly, highlighting an important role for PDK1/AKT signaling along PAM pathway in controlling cancer cell resistance to CDK4/CDK6 inhibitors (522).

**MP7** (PDK1 Inhibitor)

MP7 is another PDK1-specific inhibitor capable of inhibiting PAM pathway through the PDK1/AKT axis. MP7 owns the unique ability to induce PDK1 t-loop dephosphorylation, decreasing basal phosphorylation of AKT. As a result, MP7 has been found to reduce soft agar colony formation of cancer cell lines, as well as primary patient-derived cancer xenograft cell lines (523). Furthermore, combination of MP7 and Aurora kinase A inhibitor alisertib shows significant reduction of proliferation, and suppression of tumorsphere-forming capacity in glioblastoma multiforme cells compared to mono-agent therapies (524). MP7 significantly decreases the number of AsPC-1 and HPAF-II pancreatic ductal adenocarcinoma cells compared to untreated control cells. Besides, MP7-treated AsPC-1 and HPAF-II cells show remarkably decrease in their anchorage-independent growth on soft agar. Moreover, MP7 treatment (75 mg/kg) of subcutaneously HPAF-II cell-injected mice significantly reduces cancer growth *in vivo*, emphasising that pharmacological targeting of PDK1 can effectively decrease pancreatic ductal adenocarcinoma cell progression in xenografts. Importantly, no body weight loss is detected during MP7 treatment, indicating that MP7 is tolerable in this study (525).

**2-O-Bn-InsP5** (2-O-benzyl-myo-inositol 1,3,4,5,6-pentakisphosphate)

2-O-Bn-InsP5 demonstrates remarkable anticancer activity and pro-apoptotic efficiency while concurrently rendering tumor cells more susceptible to curcumin and tamoxifen (526). 2-O-Bn-InsP5 acts by binding to the PH domain of PDK1, leading to retention of the PDK1 in the cytosol, and thus, impeding phosphorylation and activation of AKT on Thr308 in cell lines (527). Indeed, 2-O-Bn-InsP5 specifically suppresses PDK1, and inhibits mTOR in vitro. Additionally, 2-O-Bn-InsP5 remarkably induces proapoptotic effects in cancer cells, and significantly augments the effects of antitumor drugs *in vitro*. Thus, 2-O-Bn-InsP5 may potentially be a novel inhibitor of the PAM pathway (526). Interestingly, 2-O-Bn-InsP5 interacts with the pleckstrin homology domain of PDK1 to hamper the constitution of PDK1/PLCγ1 complex, whose activation plays an important function in signalling resulting in carcinogenesis and metastasis dissemination, thereby impeding PLCγ1-dependent cellular functions in tumor cells. Indeed, 2-O-Bn-InsP5 can inhibit EGF-induced PLCγ1 phosphorylation and downstream activity, leading to hindered tumor cell migration and invasion. Furthermore, 2-O-Bn-InsP5 also suppresses tumor cell dissemination in zebrafish xenotransplants, emphasising that this PDK1/PLCγ1 complex-inhibiting agent holds potential as an anti-metastatic drug (528).

**2-Oxindole** (Oxindole, 2-Indolinone)

PDK1 inhibitor 2-oxindole derivative treatment has been shown to disrupt the PDK1/AKT pathway and exerts significant antitumor activity in NSCLC. Indeed, 2-oxindole (compound 1a) exerts antiproliferative activity at low micromolar concentration and remarkably promotes cell apoptotic-like characteristics such as nuclear condensation, nuclear fragmentation, cell shrinkage, and cleavage of cells into debris, in adenocarcinomic human alveolar basal epithelial A549 cells. Moreover, 2-oxindole significantly inhibits phosphorylation of AKT on Thr308 and Ser473, and blocks cell cycle at G1/S phase, emphasising an important cytostatic activity, thereby holding potential as a novel antitumor drug for the treatment of NSCLC (529). Notably, 2-oxindole suppresses PDK1, and therefore proteins downstream in the PAM pathway, including GSK3α, GSK3β, and Chk1, which play critical roles in glioblastoma multiforme-derived stem cell survival and growth. In fact, 2-oxindole (compound 3) shows significant anticancer activity by targeting PDK1 signaling in glioblastoma multiforme-derived stem cell self-renewal. Indeed, this remarkable antitumor activity is achieved by reducing cell viability, triggering apoptosis, and inducing differentiation of the stem cell subpopulation in two *PTEN*-mutated (U118MG and ANGMS-CSS) human glioblastoma cell lines poorly responsive to conventional chemotherapies, including temozolomide and alkylating agents normally used for the treatment of glioblastoma multiforme. Therefore, antitumor efficacy of 2-oxindole derivatives is a good starting point for the discovery and development of more efficient targeted therapies for glioblastoma multiforme (530).

## SA16 (SA-16)

SA16, a novel drug ligand that displays simultaneous inhibition of two kinases, PDK1 and Aurora kinase A, significantly decreases cell proliferation and hampers tumor invasiveness, while concurrently inducing differentiation, and subsequent apoptosis in glioblastoma multiforme stem-like cells. Thus, SA16 effectively reduces glioblastoma multiforme stem-like cell populations, suggesting its potential in overcoming chemoresistance in glioblastoma multiforme (524). In line with this, SA16 is reported to inhibit cell proliferation and clonogenic potential in pancreatic cancer cells, and it produces no toxicity at micromolar range to normal human pancreatic cells. However, its activity is limited in vivo, suggesting that further improvement of its pharmacokinetic properties is required (531).

**ABBREVIATIONS**

**ABC-DLBCL**: Activated B-cell-like diffuse large B-cell lymphoma

**BBB**: Blood-brain barrier

**BTK**: Bruton tyrosine kinase

**CBR**: Clinical benefit rate

**CLL**: Chronic lymphocytic leukemia

**CML**: Chronic myeloid leukemia

**CMML**: Chronic myelomonocytic leukemia

**CRC**: Colorectal cancer

**CRPC**: Castration resistant prostate cancer

**CSC**: Cancer stem cell

**cSCC**: Cutaneous squamous cell carcinoma

**CTCL**: Cutaneous T-cell lymphoma

**DLT**: Dose-limiting toxicity

**DSB**: Double strand break

**EGFR**: Epidermal growth factor receptor (ErbB-1) (HER1)

**EMT**: Epithelial-mesenchymal transition

**ER^+^**: Estrogen receptor positive

**FDA**: Food and Drug Administration

**FGFR**: Fibroblast growth factor receptor

**FL**: Follicular lymphoma

**FRB**: FKBP12-rapamycin-binding

**GIST**: Gastrointestinal stromal tumor

**HNSCC**: Head and neck squamous cell cancer

**HR**: Homologous recombination

**HR^+^**: Hormone receptor positive

**HUVEC**: Human umbilical vein endothelial cell

**IFN-γ**: Interferon gamma

**MCL**: Mantle cell lymphoma

**MDR**: Multi drug resistance

**MDSCs**: Myeloid-derived suppressor cells

**MZL**: Marginal zone lymphoma

**NET**: Neuroendocrine tumor

**NHEJ**: Non-homologous end joining

**NHL**: Non-Hodgkin lymphomas

**NSCLC**: Non-small-cell lung cancer

**OCCA**: Ovarian clear cell adenocarcinoma

**ORR**: Overall response rate

**PAM**: PI3K/AKT/mTORC

**PBMCs**: Peripheral blood mononuclear cells

**PDXs**: Patient-derived xenografts

**RCC**: Renal cell carcinoma

**ROS**: Reactive oxygen species

**RP2D**: Recommended phase 2 dose

**SCLC**: Small-cell lung cancer

**SD**: Stable disease

**SEGA**: Subependymal giant cell astrocytoma

**shRNA**: Short hairpin RNA

**SLL**: Small lymphocytic lymphoma

**TAMs**: Tumor-associated macrophages

**TNBC**: Triple-negative breast cancer

**Tregs**: Regulatory T-cells

**VEGF**: Vascular endothelial growth factor

**REFERENCES**

1. Liu R, Chen Y, Liu G, Li C, Song Y, Cao Z, et al. PI3K/AKT pathway as a key link modulates the multidrug resistance of cancers. Cell Death Dis. 2020;11(9):797.

2. Hillmann P, Fabbro D. PI3K/mTOR Pathway Inhibition: Opportunities in Oncology and Rare Genetic Diseases. Int J Mol Sci. 2019;20(22).

3. Liu P, Cheng H, Roberts TM, Zhao JJ. Targeting the phosphoinositide 3-kinase pathway in cancer. Nat Rev Drug Discov. 2009;8(8):627-44.

4. Maira SM, Pecchi S, Huang A, Burger M, Knapp M, Sterker D, et al. Identification and characterization of NVP-BKM120, an orally available pan-class I PI3-kinase inhibitor. Mol Cancer Ther. 2012;11(2):317-28.

5. Nur Husna SM, Tan HT, Mohamud R, Dyhl-Polk A, Wong KK. Inhibitors targeting CDK4/6, PARP and PI3K in breast cancer: a review. Ther Adv Med Oncol. 2018;10:1758835918808509.

6. Yang S, Li X, Guan W, Qian M, Yao Z, Yin X, et al. NVP-BKM120 inhibits colon cancer growth via FoxO3a-dependent PUMA induction. Oncotarget. 2017;8(47):83052-62.

7. Bavelloni A, Focaccia E, Piazzi M, Orsini A, Ramazzotti G, Cocco L, et al. Therapeutic potential of nvp-bkm120 in human osteosarcomas cells. J Cell Physiol. 2019;234(7):10907-17.

8. Fiedler M, Schulz D, Piendl G, Brockhoff G, Eichberger J, Menevse AN, et al. Buparlisib modulates PD-L1 expression in head and neck squamous cell carcinoma cell lines. Exp Cell Res. 2020;396(1):112259.

9. Ragon BK, Kantarjian H, Jabbour E, Ravandi F, Cortes J, Borthakur G, et al. Buparlisib, a PI3K inhibitor, demonstrates acceptable tolerability and preliminary activity in a phase I trial of patients with advanced leukemias. Am J Hematol. 2017;92(1):7-11.

10. Bendell JC, Rodon J, Burris HA, de Jonge M, Verweij J, Birle D, et al. Phase I, dose-escalation study of BKM120, an oral pan-Class I PI3K inhibitor, in patients with advanced solid tumors. J Clin Oncol. 2012;30(3):282-90.

11. Ando Y, Inada-Inoue M, Mitsuma A, Yoshino T, Ohtsu A, Suenaga N, et al. Phase I dose-escalation study of buparlisib (BKM120), an oral pan-class I PI3K inhibitor, in Japanese patients with advanced solid tumors. Cancer Sci. 2014;105(3):347-53.

12. McKay RR, De Velasco G, Werner L, Bellmunt J, Harshman L, Sweeney C, et al. A phase 1 study of buparlisib and bevacizumab in patients with metastatic renal cell carcinoma progressing on vascular endothelial growth factor-targeted therapies. Cancer. 2016;122(15):2389-98.

13. McRee AJ, Marcom PK, Moore DT, Zamboni WC, Kornblum ZA, Hu Z, et al. A Phase I Trial of the PI3K Inhibitor Buparlisib Combined With Capecitabine in Patients With Metastatic Breast Cancer. Clin Breast Cancer. 2018;18(4):289-97.

14. Ma CX, Luo J, Naughton M, Ademuyiwa F, Suresh R, Griffith M, et al. A Phase I Trial of BKM120 (Buparlisib) in Combination with Fulvestrant in Postmenopausal Women with Estrogen Receptor-Positive Metastatic Breast Cancer. Clin Cancer Res. 2016;22(7):1583-91.

15. Owonikoko TK, Harvey RD, Carthon B, Chen Z, Lewis C, Collins H, et al. A Phase I Study of Safety, Pharmacokinetics, and Pharmacodynamics of Concurrent Everolimus and Buparlisib Treatment in Advanced Solid Tumors. Clin Cancer Res. 2020;26(11):2497-505.

16. Stewart CM, Michaud L, Whiting K, Nakajima R, Nichols C, De Frank S, et al. Phase I/Ib Study of the Efficacy and Safety of Buparlisib and Ibrutinib Therapy in MCL, FL, and DLBCL with Serial Cell-Free DNA Monitoring. Clin Cancer Res. 2022;28(1):45-56.

17. Goodwin R, Jonker D, Chen E, Kennecke H, Cabanero M, Tsao MS, et al. A phase Ib study of a PI3Kinase inhibitor BKM120 in combination with panitumumab in patients with KRAS wild-type advanced colorectal cancer. Invest New Drugs. 2020;38(4):1077-84.

18. Bedard PL, Tabernero J, Janku F, Wainberg ZA, Paz-Ares L, Vansteenkiste J, et al. A phase Ib dose-escalation study of the oral pan-PI3K inhibitor buparlisib (BKM120) in combination with the oral MEK1/2 inhibitor trametinib (GSK1120212) in patients with selected advanced solid tumors. Clin Cancer Res. 2015;21(4):730-8.

19. Zambrano CC, Schuler MH, Machiels J, Hess D, Paz-Ares L, Awada A, et al. Phase lb study of buparlisib (BKM120) plus either paclitaxel (PTX) in advanced solid tumors (aST) or PTX plus trastuzumab (TZ) in HER2+ breast cancer (BC). (Abstract). J Clin Oncol. 2014;32(15).

20. Lu YS, Lee KS, Chao TY, Tseng LM, Chitapanarux I, Chen SC, et al. A Phase Ib Study of Alpelisib or Buparlisib Combined with Tamoxifen Plus Goserelin in Premenopausal Women with HR-Positive HER2-Negative Advanced Breast Cancer. Clin Cancer Res. 2021;27(2):408-17.

21. Hyman DM, Snyder AE, Carvajal RD, Gerecitano JF, Voss MH, Ho AL, et al. Parallel phase Ib studies of two schedules of buparlisib (BKM120) plus carboplatin and paclitaxel (q21 days or q28 days) for patients with advanced solid tumors. Cancer Chemother Pharmacol. 2015;75(4):747-55.

22. Shih KC, Chowdhary SA, Becker KP, Baehring JM, Liggett WH, Burris HA, et al. A phase II study of the combination of BKM120 (buparlisib) and bevacizumab in patients with relapsed/refractory glioblastoma multiforme (GBM). (Abstract). J Clin Oncol. 2015;33(15).

23. Brisson RJ, Dekker A, De Souza JA, Saloura V, Vokes EE, Seiwert TY. A pilot study of the pan-class I PI3K inhibitor buparlisib in combination with cetuximab in patients with recurrent/metastatic head and neck cancer. (Abstract). J Clin Oncol. 2017;35(15).

24. Younes A, Salles G, Martinelli G, Bociek RG, Barrigon DC, Barca EG, et al. Pan-phosphatidylinositol 3-kinase inhibition with buparlisib in patients with relapsed or refractory non-Hodgkin lymphoma. Haematologica. 2017;102(12):2104-12.

25. Garrido-Castro AC, Saura C, Barroso-Sousa R, Guo H, Ciruelos E, Bermejo B, et al. Phase 2 study of buparlisib (BKM120), a pan-class I PI3K inhibitor, in patients with metastatic triple-negative breast cancer. Breast Cancer Res. 2020;22(1):120.

26. Amaral T, Niessner H, Sinnberg T, Thomas I, Meiwes A, Garbe C, et al. An open-label, single-arm, phase II trial of buparlisib in patients with melanoma brain metastases not eligible for surgery or radiosurgery-the BUMPER study. Neurooncol Adv. 2020;2(1):vdaa140.

27. Kim HR, Kang HN, Yun MR, Ju KY, Choi JW, Jung DM, et al. Mouse-human co-clinical trials demonstrate superior anti-tumour effects of buparlisib (BKM120) and cetuximab combination in squamous cell carcinoma of head and neck. Br J Cancer. 2020;123(12):1720-9.

28. Tanaka H, Yoshida M, Tanimura H, Fujii T, Sakata K, Tachibana Y, et al. The selective class I PI3K inhibitor CH5132799 targets human cancers harboring oncogenic PIK3CA mutations. Clin Cancer Res. 2011;17(10):3272-81.

29. Ohwada J, Ebiike H, Kawada H, Tsukazaki M, Nakamura M, Miyazaki T, et al. Discovery and biological activity of a novel class I PI3K inhibitor, CH5132799. Bioorg Med Chem Lett. 2011;21(6):1767-72.

30. Blagden S, Omlin A, Olmin A, Josephs D, Stavraka C, Zivi A, et al. First-in-human study of CH5132799, an oral class I PI3K inhibitor, studying toxicity, pharmacokinetics, and pharmacodynamics, in patients with metastatic cancer. Clin Cancer Res. 2014;20(23):5908-17.

31. Liu N, Rowley BR, Bull CO, Schneider C, Haegebarth A, Schatz CA, et al. BAY 80-6946 is a highly selective intravenous PI3K inhibitor with potent p110α and p110δ activities in tumor cell lines and xenograft models. Mol Cancer Ther. 2013;12(11):2319-30.

32. Yan J, Yang S, Tian H, Zhang Y, Zhao H. Copanlisib promotes growth inhibition and apoptosis by modulating the AKT/FoxO3a/PUMA axis in colorectal cancer. Cell Death Dis. 2020;11(11):943.

33. García-Valverde A, Rosell J, Serna G, Valverde C, Carles J, Nuciforo P, et al. Preclinical Activity of PI3K Inhibitor Copanlisib in Gastrointestinal Stromal Tumor. Mol Cancer Ther. 2020;19(6):1289-97.

34. Tarantelli C, Lange M, Gaudio E, Cascione L, Spriano F, Kwee I, et al. Copanlisib synergizes with conventional and targeted agents including venetoclax in B- and T-cell lymphoma models. Blood Adv. 2020;4(5):819-29.

35. Markham A. Copanlisib: First Global Approval. Drugs. 2017;77(18):2057-62.

36. Patnaik A, Appleman LJ, Tolcher AW, Papadopoulos KP, Beeram M, Rasco DW, et al. First-in-human phase I study of copanlisib (BAY 80-6946), an intravenous pan-class I phosphatidylinositol 3-kinase inhibitor, in patients with advanced solid tumors and non-Hodgkin's lymphomas. Ann Oncol. 2016;27(10):1928-40.

37. Doi T, Fuse N, Yoshino T, Kojima T, Bando H, Miyamoto H, et al. A Phase I study of intravenous PI3K inhibitor copanlisib in Japanese patients with advanced or refractory solid tumors. Cancer Chemother Pharmacol. 2017;79(1):89-98.

38. Qualls D, Lam HYJ, Whiting K, Kumar A, Matasar MJ, Owens C, et al. A phase 1 trial of copanlisib plus ibrutinib in relapsed/refractory mantle cell lymphoma. Blood Adv. 2022.

39. Kim RD, Alberts SR, Peña C, Genvresse I, Ajavon-Hartmann A, Xia C, et al. Phase I dose-escalation study of copanlisib in combination with gemcitabine or cisplatin plus gemcitabine in patients with advanced cancer. Br J Cancer. 2018;118(4):462-70.

40. Ramanathan RK, Von Hoff DD, Eskens F, Blumenschein G, Richards D, Genvresse I, et al. Phase Ib Trial of the PI3K Inhibitor Copanlisib Combined with the Allosteric MEK Inhibitor Refametinib in Patients with Advanced Cancer. Target Oncol. 2020;15(2):163-74.

41. Yhim HY, Kim T, Kim SJ, Shin HJ, Koh Y, Kim JS, et al. Combination treatment of copanlisib and gemcitabine in relapsed/refractory PTCL (COSMOS): an open-label phase I/II trial. Ann Oncol. 2021;32(4):552-9.

42. Dreyling M, Morschhauser F, Bouabdallah K, Bron D, Cunningham D, Assouline SE, et al. Phase II study of copanlisib, a PI3K inhibitor, in relapsed or refractory, indolent or aggressive lymphoma. Ann Oncol. 2017;28(9):2169-78.

43. Lenz G, Hawkes E, Verhoef G, Haioun C, Thye Lim S, Seog Heo D, et al. Single-agent activity of phosphatidylinositol 3-kinase inhibition with copanlisib in patients with molecularly defined relapsed or refractory diffuse large B-cell lymphoma. Leukemia. 2020;34(8):2184-97.

44. Dreyling M, Santoro A, Mollica L, Leppä S, Follows GA, Lenz G, et al. Phosphatidylinositol 3-Kinase Inhibition by Copanlisib in Relapsed or Refractory Indolent Lymphoma. J Clin Oncol. 2017;35(35):3898-905.

45. Damodaran S, Zhao F, Deming DA, Mitchell EP, Wright JJ, Gray RJ, et al. Phase II Study of Copanlisib in Patients With Tumors With PIK3CA Mutations: Results From the NCI-MATCH ECOG-ACRIN Trial (EAY131) Subprotocol Z1F. J Clin Oncol. 2022;40(14):1552-61.

46. Tan ES, Cao B, Kim J, Al-Toubah TE, Mehta R, Centeno BA, et al. Phase 2 study of copanlisib in combination with gemcitabine and cisplatin in advanced biliary tract cancers. Cancer. 2021;127(8):1293-300.

47. Matasar MJ, Capra M, Özcan M, Lv F, Li W, Yañez E, et al. Copanlisib plus rituximab versus placebo plus rituximab in patients with relapsed indolent non-Hodgkin lymphoma (CHRONOS-3): a double-blind, randomised, placebo-controlled, phase 3 trial. Lancet Oncol. 2021;22(5):678-89.

48. Matasar MJ, Dreyling M, Leppä S, Santoro A, Pedersen M, Buvaylo V, et al. Feasibility of Combining the Phosphatidylinositol 3-Kinase Inhibitor Copanlisib With Rituximab-Based Immunochemotherapy in Patients With Relapsed Indolent B-cell Lymphoma. Clin Lymphoma Myeloma Leuk. 2021;21(11):e886-e94.

49. Enzenmüller S, Gonzalez P, Karpel-Massler G, Debatin KM, Fulda S. GDC-0941 enhances the lysosomal compartment via TFEB and primes glioblastoma cells to lysosomal membrane permeabilization and cell death. Cancer Lett. 2013;329(1):27-36.

50. Zheng L, Yang W, Zhang C, Ding WJ, Zhu H, Lin NM, et al. GDC-0941 sensitizes breast cancer to ABT-737 in vitro and in vivo through promoting the degradation of Mcl-1. Cancer Lett. 2011;309(1):27-36.

51. Usman MW, Gao J, Zheng T, Rui C, Li T, Bian X, et al. Macrophages confer resistance to PI3K inhibitor GDC-0941 in breast cancer through the activation of NF-κB signaling. Cell Death Dis. 2018;9(8):809.

52. Li X, Zhang Y, Walana W, Zhao F, Li F, Luo F. GDC-0941 and CXCL8 (3-72) K11R/G31P combination therapy confers enhanced efficacy against breast cancer. Future Oncol. 2020;16(14):911-21.

53. Liang C, Yu X, Xiong N, Zhang Z, Sun Z, Dong Y. Pictilisib Enhances the Antitumor Effect of Doxorubicin and Prevents Tumor-Mediated Bone Destruction by Blockade of PI3K/AKT Pathway. Front Oncol. 2020;10:615146.

54. O'Brien C, Wallin JJ, Sampath D, GuhaThakurta D, Savage H, Punnoose EA, et al. Predictive biomarkers of sensitivity to the phosphatidylinositol 3' kinase inhibitor GDC-0941 in breast cancer preclinical models. Clin Cancer Res. 2010;16(14):3670-83.

55. Sarker D, Ang JE, Baird R, Kristeleit R, Shah K, Moreno V, et al. First-in-human phase I study of pictilisib (GDC-0941), a potent pan-class I phosphatidylinositol-3-kinase (PI3K) inhibitor, in patients with advanced solid tumors. Clin Cancer Res. 2015;21(1):77-86.

56. Leong S, Moss RA, Bowles DW, Ware JA, Zhou J, Spoerke JM, et al. A Phase I Dose-Escalation Study of the Safety and Pharmacokinetics of Pictilisib in Combination with Erlotinib in Patients with Advanced Solid Tumors. Oncologist. 2017;22(12):1491-9.

57. Schöffski P, Cresta S, Mayer IA, Wildiers H, Damian S, Gendreau S, et al. A phase Ib study of pictilisib (GDC-0941) in combination with paclitaxel, with and without bevacizumab or trastuzumab, and with letrozole in advanced breast cancer. Breast Cancer Res. 2018;20(1):109.

58. Soria JC, Adjei AA, Bahleda R, Besse B, Ferte C, Planchard D, et al. A phase IB dose-escalation study of the safety and pharmacokinetics of pictilisib in combination with either paclitaxel and carboplatin (with or without bevacizumab) or pemetrexed and cisplatin (with or without bevacizumab) in patients with advanced non-small cell lung cancer. Eur J Cancer. 2017;86:186-96.

59. Schmid P, Pinder SE, Wheatley D, Macaskill J, Zammit C, Hu J, et al. Phase II Randomized Preoperative Window-of-Opportunity Study of the PI3K Inhibitor Pictilisib Plus Anastrozole Compared With Anastrozole Alone in Patients With Estrogen Receptor-Positive Breast Cancer. J Clin Oncol. 2016;34(17):1987-94.

60. Jiang Z, Gong T, Wei H. CDKL5 promotes proliferation, migration, and chemotherapeutic drug resistance of glioma cells via activation of the PI3K/AKT signaling pathway. FEBS Open Bio. 2020;10(2):268-77.

61. Bechter OE, Dumez H, Costermans J, Punie K, Liu L, Jiang J, et al. Phase I safety and pharmacokinetic (PK) dose escalation study with SAR245408 (S) polymorph E, a PI3K inhibitor, in patients (pts) with solid tumors or lymphoma. (Abstract). J Clin Oncol. 2015;33(15).

62. Shapiro GI, Rodon J, Bedell C, Kwak EL, Baselga J, Braña I, et al. Phase I safety, pharmacokinetic, and pharmacodynamic study of SAR245408 (XL147), an oral pan-class I PI3K inhibitor, in patients with advanced solid tumors. Clin Cancer Res. 2014;20(1):233-45.

63. Soria JC, LoRusso P, Bahleda R, Lager J, Liu L, Jiang J, et al. Phase I dose-escalation study of pilaralisib (SAR245408, XL147), a pan-class I PI3K inhibitor, in combination with erlotinib in patients with solid tumors. Oncologist. 2015;20(3):245-6.

64. Wheler J, Mutch D, Lager J, Castell C, Liu L, Jiang J, et al. Phase I Dose-Escalation Study of Pilaralisib (SAR245408, XL147) in Combination with Paclitaxel and Carboplatin in Patients with Solid Tumors. Oncologist. 2017;22(4):377-e37.

65. Blackwell K, Burris H, Gomez P, Lynn Henry N, Isakoff S, Campana F, et al. Phase I/II dose-escalation study of PI3K inhibitors pilaralisib or voxtalisib in combination with letrozole in patients with hormone-receptor-positive and HER2-negative metastatic breast cancer refractory to a non-steroidal aromatase inhibitor. Breast Cancer Res Treat. 2015;154(2):287-97.

66. Tolaney S, Burris H, Gartner E, Mayer IA, Saura C, Maurer M, et al. Phase I/II study of pilaralisib (SAR245408) in combination with trastuzumab or trastuzumab plus paclitaxel in trastuzumab-refractory HER2-positive metastatic breast cancer. Breast Cancer Res Treat. 2015;149(1):151-61.

67. Matulonis U, Vergote I, Backes F, Martin LP, McMeekin S, Birrer M, et al. Phase II study of the PI3K inhibitor pilaralisib (SAR245408; XL147) in patients with advanced or recurrent endometrial carcinoma. Gynecol Oncol. 2015;136(2):246-53.

68. Qin AC, Li Y, Zhou LN, Xing CG, Lu XS. Dual PI3K-BRD4 Inhibitor SF1126 Inhibits Colorectal Cancer Cell Growth in Vitro and in Vivo. Cell Physiol Biochem. 2019;52(4):758-68.

69. Singh AR, Joshi S, Burgoyne AM, Sicklick JK, Ikeda S, Kono Y, et al. Single Agent and Synergistic Activity of the "First-in-Class" Dual PI3K/BRD4 Inhibitor SF1126 with Sorafenib in Hepatocellular Carcinoma. Mol Cancer Ther. 2016;15(11):2553-62.

70. Goldin AN, Singh A, Joshi S, Jamieson C, Durden DL. Augmented Antitumor Activity for Novel Dual PI3K/BDR4 Inhibitors, SF2523 and SF1126 in Ewing Sarcoma. J Pediatr Hematol Oncol. 2021;43(3):e304-e11.

71. Harder BG, Peng S, Sereduk CP, Sodoma AM, Kitange GJ, Loftus JC, et al. Inhibition of phosphatidylinositol 3-kinase by PX-866 suppresses temozolomide-induced autophagy and promotes apoptosis in glioblastoma cells. Mol Med. 2019;25(1):49.

72. Hong DS, Bowles DW, Falchook GS, Messersmith WA, George GC, O'Bryant CL, et al. A multicenter phase I trial of PX-866, an oral irreversible phosphatidylinositol 3-kinase inhibitor, in patients with advanced solid tumors. Clin Cancer Res. 2012;18(15):4173-82.

73. Bowles DW, Ma WW, Senzer N, Brahmer JR, Adjei AA, Davies M, et al. A multicenter phase 1 study of PX-866 in combination with docetaxel in patients with advanced solid tumours. Br J Cancer. 2013;109(5):1085-92.

74. Pitz MW, Eisenhauer EA, MacNeil MV, Thiessen B, Easaw JC, Macdonald DR, et al. Phase II study of PX-866 in recurrent glioblastoma. Neuro Oncol. 2015;17(9):1270-4.

75. Hotte SJ, Chi KN, Joshua AM, Tu D, Macfarlane RJ, Gregg RW, et al. A Phase II Study of PX-866 in Patients With Recurrent or Metastatic Castration-resistant Prostate Cancer: Canadian Cancer Trials Group Study IND205. Clin Genitourin Cancer. 2019;17(3):201-8.e1.

76. Namatame N, Tamaki N, Yoshizawa Y, Okamura M, Nishimura Y, Yamazaki K, et al. Antitumor profile of the PI3K inhibitor ZSTK474 in human sarcoma cell lines. Oncotarget. 2018;9(80):35141-61.

77. Zhou Q, Chen Y, Zhang L, Zhong Y, Zhang Z, Wang R, et al. Antiproliferative effect of ZSTK474 alone or in combination with chemotherapeutic drugs on HL60 and HL60/ADR cells. Oncotarget. 2017;8(24):39064-76.

78. Wang Y, Liu J, Qiu Y, Jin M, Chen X, Fan G, et al. ZSTK474, a specific class I phosphatidylinositol 3-kinase inhibitor, induces G1 arrest and autophagy in human breast cancer MCF-7 cells. Oncotarget. 2016;7(15):19897-909.

79. Jasek-Gajda E, Jurkowska H, Jasińska M, Lis GJ. Targeting the MAPK/ERK and PI3K/AKT Signaling Pathways Affects NRF2, Trx and GSH Antioxidant Systems in Leukemia Cells. Antioxidants (Basel). 2020;9(7).

80. Tran KB, Kolekar S, Jabed A, Jaynes P, Shih JH, Wang Q, et al. Diverse mechanisms activate the PI 3-kinase/mTOR pathway in melanomas: implications for the use of PI 3-kinase inhibitors to overcome resistance to inhibitors of BRAF and MEK. BMC Cancer. 2021;21(1):136.

81. Engelman JA. Targeting PI3K signalling in cancer: opportunities, challenges and limitations. Nat Rev Cancer. 2009;9(8):550-62.

82. Fritsch C, Huang A, Chatenay-Rivauday C, Schnell C, Reddy A, Liu M, et al. Characterization of the novel and specific PI3Kα inhibitor NVP-BYL719 and development of the patient stratification strategy for clinical trials. Mol Cancer Ther. 2014;13(5):1117-29.

83. Kim KJ, Kim JW, Sung JH, Suh KJ, Lee JY, Kim SH, et al. PI3K-targeting strategy using alpelisib to enhance the antitumor effect of paclitaxel in human gastric cancer. Sci Rep. 2020;10(1):12308.

84. Narayan P, Prowell TM, Gao JJ, Fernandes LL, Li E, Jiang X, et al. FDA Approval Summary: Alpelisib Plus Fulvestrant for Patients with HR-positive, HER2-negative, PIK3CA-mutated, Advanced or Metastatic Breast Cancer. Clin Cancer Res. 2021;27(7):1842-9.

85. Juric D, Rodon J, Tabernero J, Janku F, Burris HA, Schellens JHM, et al. Phosphatidylinositol 3-Kinase α-Selective Inhibition With Alpelisib (BYL719) in PIK3CA-Altered Solid Tumors: Results From the First-in-Human Study. J Clin Oncol. 2018;36(13):1291-9.

86. Ando Y, Iwasa S, Takahashi S, Saka H, Kakizume T, Natsume K, et al. Phase I study of alpelisib (BYL719), an α-specific PI3K inhibitor, in Japanese patients with advanced solid tumors. Cancer Sci. 2019;110(3):1021-31.

87. Jain S, Santa-Maria CA, Rademaker A, Giles FJ, Cristofanilli M, Gradishar WJ. Phase I study of Alpelisib (BYL-719) and T-DM1 in HER2-positive metastatic breast cancer after trastuzumab and taxane therapy. (Abstract). J Clin Oncol. 2017;35(15).

88. Jhaveri K, Drago JZ, Shah PD, Wang R, Pareja F, Ratzon F, et al. A Phase I Study of Alpelisib in Combination with Trastuzumab and LJM716 in Patients with PIK3CA-Mutated HER2-Positive Metastatic Breast Cancer. Clin Cancer Res. 2021;27(14):3867-75.

89. Juric D, Janku F, Rodón J, Burris HA, Mayer IA, Schuler M, et al. Alpelisib Plus Fulvestrant in PIK3CA-Altered and PIK3CA-Wild-Type Estrogen Receptor-Positive Advanced Breast Cancer: A Phase 1b Clinical Trial. JAMA Oncol. 2019;5(2):e184475.

90. Mayer IA, Abramson VG, Formisano L, Balko JM, Estrada MV, Sanders ME, et al. A Phase Ib Study of Alpelisib (BYL719), a PI3Kα-Specific Inhibitor, with Letrozole in ER+/HER2- Metastatic Breast Cancer. Clin Cancer Res. 2017;23(1):26-34.

91. Batalini F, Xiong N, Tayob N, Polak M, Eismann J, Cantley LC, et al. Phase 1b Clinical Trial with Alpelisib plus Olaparib for Patients with Advanced Triple-Negative Breast Cancer. Clin Cancer Res. 2022;28(8):1493-9.

92. Konstantinopoulos PA, Barry WT, Birrer M, Westin SN, Cadoo KA, Shapiro GI, et al. Olaparib and α-specific PI3K inhibitor alpelisib for patients with epithelial ovarian cancer: a dose-escalation and dose-expansion phase 1b trial. Lancet Oncol. 2019;20(4):570-80.

93. Pantaleo MA, Heinrich MC, Italiano A, Valverde C, Schöffski P, Grignani G, et al. A multicenter, dose-finding, phase 1b study of imatinib in combination with alpelisib as third-line treatment in patients with advanced gastrointestinal stromal tumor. BMC Cancer. 2022;22(1):511.

94. Hyman DM, Tran B, Jaime JC, Garralda E, Machiels JPH, Schellens JHM, et al. Phase Ib study of BGJ398 in combination with BYL719 in patients (pts) with select advanced solid tumors. (Abstract). J Clin Oncol. 2016;34(15).

95. Juric D, Soria JC, Sharma S, Banerji U, Azaro A, Desai J, et al. A phase 1b dose-escalation study of BYL719 plus binimetinib (MEK162) in patients with selected advanced solid tumors. (Abstract). J Clin Oncol. 2014;32(15).

96. Curigliano G, Martin M, Jhaveri K, Beck JT, Tortora G, Fazio N, et al. Alpelisib in combination with everolimus ± exemestane in solid tumours: Phase Ib randomised, open-label, multicentre study. Eur J Cancer. 2021;151:49-62.

97. Dunn LA, Riaz N, Fury MG, McBride SM, Michel L, Lee NY, et al. A Phase 1b Study of Cetuximab and BYL719 (Alpelisib) Concurrent with Intensity Modulated Radiation Therapy in Stage III-IVB Head and Neck Squamous Cell Carcinoma. Int J Radiat Oncol Biol Phys. 2020;106(3):564-70.

98. van Geel RMJM, Tabernero J, Elez E, Bendell JC, Spreafico A, Schuler M, et al. A Phase Ib Dose-Escalation Study of Encorafenib and Cetuximab with or without Alpelisib in Metastatic. Cancer Discov. 2017;7(6):610-9.

99. Sharma P, Abramson VG, O'Dea A, Nye L, Mayer I, Pathak HB, et al. Clinical and Biomarker Results from Phase I/II Study of PI3K Inhibitor Alpelisib plus Nab-paclitaxel in HER2-Negative Metastatic Breast Cancer. Clin Cancer Res. 2021;27(14):3896-904.

100. Razavi P, Dickler MN, Shah PD, Toy W, Brown DN, Won HH, et al. Alterations in PTEN and ESR1 promote clinical resistance to alpelisib plus aromatase inhibitors. Nat Cancer. 2020;1(4):382-93.

101. PN M, EP H, LG E, RH DB, Mayer, IA, et al. Ph IB study of LEE011 and BYL719 in combination with letrozole in ER+, HER2- breast cancer. (Abstract). J Clin Oncol. 2014;32(26).

102. Savas P, Lo LL, Luen SJ, Blackley EF, Callahan J, Moodie K, et al. Alpelisib Monotherapy for PI3K-Altered, Pretreated Advanced Breast Cancer: A Phase II Study. Cancer Discov. 2022;12(9):2058-73.

103. Turner S, Chia S, Kanakamedala H, Hsu WC, Park J, Chandiwana D, et al. Effectiveness of Alpelisib + Fulvestrant Compared with Real-World Standard Treatment Among Patients with HR+, HER2-, PIK3CA-Mutated Breast Cancer. Oncologist. 2021;26(7):e1133-e42.

104. Mayer IA, Prat A, Egle D, Blau S, Fidalgo JAP, Gnant M, et al. A Phase II Randomized Study of Neoadjuvant Letrozole Plus Alpelisib for Hormone Receptor-Positive, Human Epidermal Growth Factor Receptor 2-Negative Breast Cancer (NEO-ORB). Clin Cancer Res. 2019;25(10):2975-87.

105. Tabernero J, Van Geel R, Guren TK, Yaeger RD, Spreafico A, Faris JE, et al. Phase 2 results: Encorafenib (ENCO) and cetuximab (CETUX) with or without Alpelisib (ALP) in patients with advanced BRAF-mutant colorectal cancer (BRAFm CRC). (Abstract). J Clin Oncol. 2016;34(15).

106. André F, Ciruelos E, Rubovszky G, Campone M, Loibl S, Rugo HS, et al. Alpelisib for PIK3CA-Mutated, Hormone Receptor-Positive Advanced Breast Cancer. N Engl J Med. 2019;380(20):1929-40.

107. André F, Ciruelos EM, Juric D, Loibl S, Campone M, Mayer IA, et al. Alpelisib plus fulvestrant for PIK3CA-mutated, hormone receptor-positive, human epidermal growth factor receptor-2-negative advanced breast cancer: final overall survival results from SOLAR-1. Ann Oncol. 2021;32(2):208-17.

108. Erdmann T, Klener P, Lynch JT, Grau M, Vočková P, Molinsky J, et al. Sensitivity to PI3K and AKT inhibitors is mediated by divergent molecular mechanisms in subtypes of DLBCL. Blood. 2017;130(3):310-22.

109. Wu YH, Huang YF, Chen CC, Huang CY, Chou CY. Comparing PI3K/Akt Inhibitors Used in Ovarian Cancer Treatment. Front Pharmacol. 2020;11:206.

110. Carnevalli LS, Sinclair C, Taylor MA, Gutierrez PM, Langdon S, Coenen-Stass AML, et al. PI3Kα/δ inhibition promotes anti-tumor immunity through direct enhancement of effector CD8. J Immunother Cancer. 2018;6(1):158.

111. Hernández-Prat A, Rodriguez-Vida A, Juanpere-Rodero N, Arpi O, Menéndez S, Soria-Jiménez L, et al. Novel Oral mTORC1/2 Inhibitor TAK-228 Has Synergistic Antitumor Effects When Combined with Paclitaxel or PI3Kα Inhibitor TAK-117 in Preclinical Bladder Cancer Models. Mol Cancer Res. 2019;17(9):1931-44.

112. Patel CG, Rangachari L, Patti M, Griffin C, Shou Y, Venkatakrishnan K. Characterizing the Sources of Pharmacokinetic Variability for TAK-117 (Serabelisib), an Investigational Phosphoinositide 3-Kinase Alpha Inhibitor: A Clinical Biopharmaceutics Study to Inform Development Strategy. Clin Pharmacol Drug Dev. 2019;8(5):637-46.

113. Juric D, de Bono JS, LoRusso PM, Nemunaitis J, Heath EI, Kwak EL, et al. A First-in-Human, Phase I, Dose-Escalation Study of TAK-117, a Selective PI3Kα Isoform Inhibitor, in Patients with Advanced Solid Malignancies. Clin Cancer Res. 2017;23(17):5015-23.

114. Starks DC, Rojas-Espaillat L, Meissner T, Williams CB. Phase I dose escalation study of dual PI3K/mTOR inhibition by Sapanisertib and Serabelisib in combination with paclitaxel in patients with advanced solid tumors. Gynecol Oncol. 2022;166(3):403-9.

115. Jhaveri K, Chang MT, Juric D, Saura C, Gambardella V, Melnyk A, et al. Phase I Basket Study of Taselisib, an Isoform-Selective PI3K Inhibitor, in Patients with PIK3CA-Mutant Cancers. Clin Cancer Res. 2021;27(2):447-59.

116. Filho OM, Goel S, Barry WT, Hamilton EP, Tolaney SM, Yardley DA, et al. A mouse-human phase I co-clinical trial of taselisib in combination with TDM1 in advanced HER2-positive breast cancer (MBC). (Abstract). J Clin Oncol. 2017;35(15).

117. Baird RD, van Rossum AGJ, Oliveira M, Beelen K, Gao M, Schrier M, et al. POSEIDON Trial Phase 1b Results: Safety, Efficacy and Circulating Tumor DNA Response of the Beta Isoform-Sparing PI3K Inhibitor Taselisib (GDC-0032) Combined with Tamoxifen in Hormone Receptor Positive Metastatic Breast Cancer Patients. Clin Cancer Res. 2019;25(22):6598-605.

118. Lopez JS, Selvi Miralles M, Ameratunga M, Minchom A, Pascual J, Banerji U, et al. PIPA: A phase Ib study of selective ß-isoform sparing phosphatidylinositol 3-kinase (PI3K) inhibitor taselisib (T) plus palbociclib (P) in patients (pts) with advanced solid cancers—Safety, tolerability, pharmacokinetic (PK), and pharmacodynamic (PD) analysis of the doublet combination. (Abstract). J Clin Oncol. 2019;37(15).

119. Pascual J, Lim JSJ, Macpherson IR, Armstrong AC, Ring A, Okines AFC, et al. Triplet Therapy with Palbociclib, Taselisib, and Fulvestrant in PIK3CA-Mutant Breast Cancer and Doublet Palbociclib and Taselisib in Pathway-Mutant Solid Cancers. Cancer Discov. 2021;11(1):92-107.

120. Krop IE, Jegede OA, Grilley-Olson JE, Lauring JD, Mitchell EP, Zwiebel JA, et al. Phase II Study of Taselisib in PIK3CA-Mutated Solid Tumors Other Than Breast and Squamous Lung Cancer: Results From the NCI-MATCH ECOG-ACRIN Trial (EAY131) Subprotocol I. JCO Precis Oncol. 2022;6:e2100424.

121. Saura C, Hlauschek D, Oliveira M, Zardavas D, Jallitsch-Halper A, de la Peña L, et al. Neoadjuvant letrozole plus taselisib versus letrozole plus placebo in postmenopausal women with oestrogen receptor-positive, HER2-negative, early-stage breast cancer (LORELEI): a multicentre, randomised, double-blind, placebo-controlled, phase 2 trial. Lancet Oncol. 2019;20(9):1226-38.

122. Dickler MN, Saura C, Richards DA, Krop IE, Cervantes A, Bedard PL, et al. Phase II Study of Taselisib (GDC-0032) in Combination with Fulvestrant in Patients with HER2-Negative, Hormone Receptor-Positive Advanced Breast Cancer. Clin Cancer Res. 2018;24(18):4380-7.

123. Dent S, Cortés J, Im YH, Diéras V, Harbeck N, Krop IE, et al. Phase III randomized study of taselisib or placebo with fulvestrant in estrogen receptor-positive, PIK3CA-mutant, HER2-negative, advanced breast cancer: the SANDPIPER trial. Ann Oncol. 2021;32(2):197-207.

124. Kater AP, Tonino SH, Spiering M, Chamuleau MED, Liu R, Adewoye AH, et al. Final results of a phase 1b study of the safety and efficacy of the PI3Kδ inhibitor acalisib (GS-9820) in relapsed/refractory lymphoid malignancies. Blood Cancer J. 2018;8(2):16.

125. Owusu-Brackett N, Zhao M, Akcakanat A, Evans KW, Yuca E, Dumbrava EI, et al. Targeting PI3Kβ alone and in combination with chemotherapy or immunotherapy in tumors with PTEN loss. Oncotarget. 2020;11(11):969-81.

126. Marqués M, Tranchant R, Risa-Ebrí B, Suárez-Solís ML, Fernández LC, Carrillo-de-Santa-Pau E, et al. Combined MEK and PI3K/p110β Inhibition as a Novel Targeted Therapy for Malignant Mesothelioma Displaying Sarcomatoid Features. Cancer Res. 2020;80(4):843-56.

127. Herrick WG, Kilpatrick CL, Hollingshead MG, Esposito D, O'Sullivan Coyne G, Gross AM, et al. Isoform- and Phosphorylation-specific Multiplexed Quantitative Pharmacodynamics of Drugs Targeting PI3K and MAPK Signaling in Xenograft Models and Clinical Biopsies. Mol Cancer Ther. 2021;20(4):749-60.

128. Choudhury AD, Higano CS, de Bono JS, Cook N, Rathkopf DE, Wisinski KB, et al. A Phase I Study Investigating AZD8186, a Potent and Selective Inhibitor of PI3Kβ/δ, in Patients with Advanced Solid Tumors. Clin Cancer Res. 2022;28(11):2257-69.

129. De Bono JS, Hansen A, Choudhury AD, Cook N, Heath EI, Higano C, et al. AZD8186, a potent and selective inhibitor of PI3Kβ/δ, as monotherapy and in combination with abiraterone acetate plus prednisone (AAP), in patients (pts) with metastatic castrate-resistant prostate cancer (mCRPC). (Abstract). Ann Oncol. 2018;29(8):291-2.

130. Uribe-Alvarez C, Guerrero-Rodríguez SL, Rhodes J, Cannon A, Chernoff J, Araiza-Olivera D. Targeting effector pathways in RAC1. Small GTPases. 2021;12(4):273-81.

131. Peng W, Williams LJ, Xu C, Melendez B, McKenzie JA, Chen Y, et al. Anti-OX40 Antibody Directly Enhances The Function of Tumor-Reactive CD8. Clin Cancer Res. 2019;25(21):6406-16.

132. Sarker D, Dawson NA, Aparicio AM, Dorff TB, Pantuck AJ, Vaishampayan UN, et al. A Phase I, Open-Label, Dose-Finding Study of GSK2636771, a PI3Kβ Inhibitor, Administered with Enzalutamide in Patients with Metastatic Castration-Resistant Prostate Cancer. Clin Cancer Res. 2021.

133. Arkenau HT, Mateo J, Lemech CR, Infante JR, Burris HA, Bang YJ, et al. A phase I/II, first-in-human dose-escalation study of GSK2636771 in patients (pts) with PTEN-deficient advanced tumors. (Abstract). J Clin Oncol. 2014;32(15).

134. Dumbrava EE, Burton EM, Subudhi SK, Milton DR, Aparicio A, Yap TA, et al. Phase I/II study of the selective PI3Kβ inhibitor GSK2636771 in combination with pembrolizumab in patients (pts) with metastatic castration-resistant prostate cancer (mCRPC) and PTEN loss. Journal of Clinical Oncology. 2022;40(16):5052-.

135. Jung M, Kim C, Kim H, Lee C, Lee H, Bae W, et al. SO-10 An open-label, multi-centre, phase Ib/II study of PI3Kβ selective inhibitor GSK2636771 administered in combination with paclitaxel in patients with advanced gastric cancer having alterations in PI3K/Akt pathway. Annals of Oncology. 2021;32(3).

136. Bonnevaux H, Lemaitre O, Vincent L, Levit MN, Windenberger F, Halley F, et al. Concomitant Inhibition of PI3Kβ and BRAF or MEK in PTEN-Deficient/BRAF-Mutant Melanoma Treatment: Preclinical Assessment of SAR260301 Oral PI3Kβ-Selective Inhibitor. Mol Cancer Ther. 2016;15(7):1460-71.

137. Bédard PL, Davies MA, Kopetz S, Juric D, Shapiro GI, Luke JJ, et al. First-in-human trial of the PI3Kβ-selective inhibitor SAR260301 in patients with advanced solid tumors. Cancer. 2018;124(2):315-24.

138. Dong S, Guinn D, Dubovsky JA, Zhong Y, Lehman A, Kutok J, et al. IPI-145 antagonizes intrinsic and extrinsic survival signals in chronic lymphocytic leukemia cells. Blood. 2014;124(24):3583-6.

139. Patel VM, Balakrishnan K, Douglas M, Tibbitts T, Xu EY, Kutok JL, et al. Duvelisib treatment is associated with altered expression of apoptotic regulators that helps in sensitization of chronic lymphocytic leukemia cells to venetoclax (ABT-199). Leukemia. 2017;31(9):1872-81.

140. Blair HA. Duvelisib: First Global Approval. Drugs. 2018;78(17):1847-53.

141. Horwitz SM, Koch R, Porcu P, Oki Y, Moskowitz A, Perez M, et al. Activity of the PI3K-δ,γ inhibitor duvelisib in a phase 1 trial and preclinical models of T-cell lymphoma. Blood. 2018;131(8):888-98.

142. Izutsu K, Kato K, Kiyoi H, Yamamoto G, Shimada K, Akashi K. Phase I study of duvelisib in Japanese patients with relapsed or refractory lymphoma. Int J Hematol. 2020;112(4):504-9.

143. Flinn IW, O'Brien S, Kahl B, Patel M, Oki Y, Foss FF, et al. Duvelisib, a novel oral dual inhibitor of PI3K-δ,γ, is clinically active in advanced hematologic malignancies. Blood. 2018;131(8):877-87.

144. Flinn IW, Cherry MA, Maris MB, Matous JV, Berdeja JG, Patel M. Combination trial of duvelisib (IPI-145) with rituximab or bendamustine/rituximab in patients with non-Hodgkin lymphoma or chronic lymphocytic leukemia. Am J Hematol. 2019;94(12):1325-34.

145. Davids MS, Fisher DC, Tyekucheva S, McDonough M, Hanna J, Lee B, et al. A phase 1b/2 study of duvelisib in combination with FCR (DFCR) for frontline therapy for younger CLL patients. Leukemia. 2021;35(4):1064-72.

146. Zheng Z, Gao Y, Song Y, Qian Y, Jing H, Liu T, et al. Efficacy and safety of duvelisib, a phosphoinositide 3 kinase (PI3K) δ and γ inhibitor, in Chinese patients (pts) with relapsed/refractory follicular lymphoma (R/R FL): A single-arm, open-label, multicenter, phase Ⅱ clinical trial. Journal of Clinical Oncology. 2021;39(15).

147. Flinn IW, Miller CB, Ardeshna KM, Tetreault S, Assouline SE, Mayer J, et al. DYNAMO: A Phase II Study of Duvelisib (IPI-145) in Patients With Refractory Indolent Non-Hodgkin Lymphoma. J Clin Oncol. 2019;37(11):912-22.

148. Flinn IW, Hillmen P, Montillo M, Nagy Z, Illés Á, Etienne G, et al. The phase 3 DUO trial: duvelisib vs ofatumumab in relapsed and refractory CLL/SLL. Blood. 2018;132(23):2446-55.

149. Davids MS, Kuss BJ, Hillmen P, Montillo M, Moreno C, Essell J, et al. Efficacy and Safety of Duvelisib Following Disease Progression on Ofatumumab in Patients with Relapsed/Refractory CLL or SLL in the DUO Crossover Extension Study. Clin Cancer Res. 2020;26(9):2096-103.

150. Berta GN, Di Scipio F, Yang Z, Oberto A, Abbadessa G, Romano F, et al. Chemical Oral Cancerogenesis Is Impaired in PI3Kγ Knockout and Kinase-Dead Mice. Cancers (Basel). 2021;13(16).

151. De Vera AA, Gupta P, Lei Z, Liao D, Narayanan S, Teng Q, et al. Immuno-oncology agent IPI-549 is a modulator of P-glycoprotein (P-gp, MDR1, ABCB1)-mediated multidrug resistance (MDR) in cancer: In vitro and in vivo. Cancer Lett. 2019;442:91-103.

152. Jiang M, He K, Qiu T, Sun J, Liu Q, Zhang X, et al. Tumor-targeted delivery of silibinin and IPI-549 synergistically inhibit breast cancer by remodeling the microenvironment. Int J Pharm. 2020;581:119239.

153. Sullivan RJ, Hong DS, Tolcher AW, Patnaik A, Shapiro G, Chmielowski B, et al. Initial results from first-in-human study of IPI-549, a tumor macrophage-targeting agent, combined with nivolumab in advanced solid tumors. Journal of Clinical Oncology. 2018;36(15).

154. Tomczak P, Popovic L, Barthelemy P, Janicic A, Sevillano Fernandez E, Borchiellini D, et al. Preliminary analysis of a phase II, multicenter, randomized, active-control study to evaluate the efficacy and safety of eganelisib (IPI 549) in combination with nivolumab compared to nivolumab monotherapy in patients with advanced urothelial carcinoma. Journal of Clinical Oncology. 2021;39(6).

155. Locatelli SL, Careddu G, Serio S, Consonni FM, Maeda A, Viswanadha S, et al. Targeting Cancer Cells and Tumor Microenvironment in Preclinical and Clinical Models of Hodgkin Lymphoma Using the Dual PI3Kδ/γ Inhibitor RP6530. Clin Cancer Res. 2019;25(3):1098-112.

156. Huen A, Haverkos BM, Zain J, Radhakrishnan R, Lechowicz MJ, Devata S, et al. Phase I/Ib Study of Tenalisib (RP6530), a Dual PI3K δ/γ Inhibitor in Patients with Relapsed/Refractory T-Cell Lymphoma. Cancers (Basel). 2020;12(8).

157. Spriano F, Tarantelli C, Gaudio E, Gerlach MM, Priebe V, Cascione L, et al. Single and combined BTK and PI3Kδ inhibition with acalabrutinib and ACP-319 in pre-clinical models of aggressive lymphomas. Br J Haematol. 2019;187(5):595-601.

158. Niemann CU, Mora-Jensen HI, Dadashian EL, Krantz F, Covey T, Chen SS, et al. Combined BTK and PI3Kδ Inhibition with Acalabrutinib and ACP-319 Improves Survival and Tumor Control in CLL Mouse Model. Clin Cancer Res. 2017;23(19):5814-23.

159. Lanasa MC, Glenn M, Mato AR, Allgood SD, Wong S, Amore B, et al. First-in-human study of AMG 319, a highly selective, small molecule inhibitor of PI3Kδ, in adult patients with relapsed or refractory lymphoid malignancies. (Abstract). Blood. 2013;122(21):678.

160. Meadows SA, Vega F, Kashishian A, Johnson D, Diehl V, Miller LL, et al. PI3Kδ inhibitor, GS-1101 (CAL-101), attenuates pathway signaling, induces apoptosis, and overcomes signals from the microenvironment in cellular models of Hodgkin lymphoma. Blood. 2012;119(8):1897-900.

161. Arita A, Hanlon K, Chkourko H, Lannutti BJ, Johnson DM, Gabrilove JL, et al. Effect of phosphotidylinositol 3-kinase-delta inhibitor idelalisib (GS-1101) on signaling in primary non-Hodgkin lymphoma cells: Correlative studies from NCT01306643. (Abstract). J Clin Oncol. 2013;31(15).

162. George JA, Alshebli Z, Alneyadi A, Al Mukhaini N, Al-Salam S, Sudhadevi M, et al. Idelalisib induces apoptosis in the lymphoid tissues and impairs lung function in mice. J Chemother. 2020;32(2):88-97.

163. Serrat N, Guerrero-Hernández M, Matas-Céspedes A, Yahiaoui A, Valero JG, Nadeu F, et al. PI3Kδ inhibition reshapes follicular lymphoma-immune microenvironment cross talk and unleashes the activity of venetoclax. Blood Adv. 2020;4(17):4217-31.

164. Miller BW, Przepiorka D, de Claro RA, Lee K, Nie L, Simpson N, et al. FDA approval: idelalisib monotherapy for the treatment of patients with follicular lymphoma and small lymphocytic lymphoma. Clin Cancer Res. 2015;21(7):1525-9.

165. Shah A, Mangaonkar A. Idelalisib: A Novel PI3Kδ Inhibitor for Chronic Lymphocytic Leukemia. Ann Pharmacother. 2015;49(10):1162-70.

166. Brown JR, Byrd JC, Coutre SE, Benson DM, Flinn IW, Wagner-Johnston ND, et al. Idelalisib, an inhibitor of phosphatidylinositol 3-kinase p110δ, for relapsed/refractory chronic lymphocytic leukemia. Blood. 2014;123(22):3390-7.

167. Kahl BS, Spurgeon SE, Furman RR, Flinn IW, Coutre SE, Brown JR, et al. A phase 1 study of the PI3Kδ inhibitor idelalisib in patients with relapsed/refractory mantle cell lymphoma (MCL). Blood. 2014;123(22):3398-405.

168. Flinn IW, Kahl BS, Leonard JP, Furman RR, Brown JR, Byrd JC, et al. Idelalisib, a selective inhibitor of phosphatidylinositol 3-kinase-δ, as therapy for previously treated indolent non-Hodgkin lymphoma. Blood. 2014;123(22):3406-13.

169. Coutre SE, Flinn IW, de Vos S, Barrientos JC, Schreeder MT, Wagner-Johnson ND, et al. Idelalisib in Combination With Rituximab or Bendamustine or Both in Patients With Relapsed/Refractory Chronic Lymphocytic Leukemia. Hemasphere. 2018;2(3):e39.

170. Fukuhara N, Kinoshita T, Yamamoto K, Nagai H, Izutsu K, Yamamoto G, et al. Phase 1b study to investigate the safety and tolerability of idelalisib in Japanese patients with relapsed/refractory follicular lymphoma and chronic lymphocytic leukemia. Jpn J Clin Oncol. 2020;50(12):1395-402.

171. Danilov AV, Herbaux C, Walter HS, Hillmen P, Rule SA, Kio EA, et al. Phase Ib Study of Tirabrutinib in Combination with Idelalisib or Entospletinib in Previously Treated Chronic Lymphocytic Leukemia. Clin Cancer Res. 2020;26(12):2810-8.

172. Gopal AK, Fanale MA, Moskowitz CH, Shustov AR, Mitra S, Ye W, et al. Phase II study of idelalisib, a selective inhibitor of PI3Kδ, for relapsed/refractory classical Hodgkin lymphoma. Ann Oncol. 2017;28(5):1057-63.

173. Gopal AK, Kahl BS, de Vos S, Wagner-Johnston ND, Schuster SJ, Jurczak WJ, et al. PI3Kδ inhibition by idelalisib in patients with relapsed indolent lymphoma. N Engl J Med. 2014;370(11):1008-18.

174. Fjordén K, Ekberg S, Kuric N, Smedby KE, Lagerlöf I, Larsen TS, et al. Idelalisib in relapsed/refractory diffuse large B-cell lymphoma: results from a Nordic Lymphoma Group phase II trial. Br J Haematol. 2021.

175. O'Brien SM, Lamanna N, Kipps TJ, Flinn I, Zelenetz AD, Burger JA, et al. A phase 2 study of idelalisib plus rituximab in treatment-naïve older patients with chronic lymphocytic leukemia. Blood. 2015;126(25):2686-94.

176. Tomowiak C, Poulain S, Herbaux C, Perrot A, Mahé B, Morel P, et al. Obinutuzumab and idelalisib in symptomatic patients with relapsed/refractory Waldenström macroglobulinemia. Blood Adv. 2021;5(9):2438-46.

177. Furman RR, Sharman JP, Coutre SE, Cheson BD, Pagel JM, Hillmen P, et al. Idelalisib and rituximab in relapsed chronic lymphocytic leukemia. N Engl J Med. 2014;370(11):997-1007.

178. Sharman JP, Coutre SE, Furman RR, Cheson BD, Pagel JM, Hillmen P, et al. Final Results of a Randomized, Phase III Study of Rituximab With or Without Idelalisib Followed by Open-Label Idelalisib in Patients With Relapsed Chronic Lymphocytic Leukemia. J Clin Oncol. 2019;37(16):1391-402.

179. Ghia P, Pluta A, Wach M, Lysak D, Kozak T, Simkovic M, et al. ASCEND: Phase III, Randomized Trial of Acalabrutinib Versus Idelalisib Plus Rituximab or Bendamustine Plus Rituximab in Relapsed or Refractory Chronic Lymphocytic Leukemia. J Clin Oncol. 2020;38(25):2849-61.

180. Jones JA, Robak T, Brown JR, Awan FT, Badoux X, Coutre S, et al. Efficacy and safety of idelalisib in combination with ofatumumab for previously treated chronic lymphocytic leukaemia: an open-label, randomised phase 3 trial. Lancet Haematol. 2017;4(3):e114-e26.

181. Zelenetz AD, Barrientos JC, Brown JR, Coiffier B, Delgado J, Egyed M, et al. Idelalisib or placebo in combination with bendamustine and rituximab in patients with relapsed or refractory chronic lymphocytic leukaemia: interim results from a phase 3, randomised, double-blind, placebo-controlled trial. Lancet Oncol. 2017;18(3):297-311.

182. Eyre TA, Preston G, Kagdi H, Islam A, Nicholson T, Smith HW, et al. A retrospective observational study to evaluate the clinical outcomes and routine management of patients with chronic lymphocytic leukaemia treated with idelalisib and rituximab in the UK and Ireland (RETRO-idel). Br J Haematol. 2021;194(1):69-77.

183. Jiang B, Qi J, Song Y, Li Z, Tu M, Ping L, et al. Phase 1 clinical trial of the PI3Kδ inhibitor YY-20394 in patients with B-cell hematological malignancies. J Hematol Oncol. 2021;14(1):130.

184. Jin J, Cen H, Zhou K, Xu X, Li F, Wu T, et al. A phase Ib study of a PI3Kδ inhibitor Linperlisib in patients with relapsed or refractory peripheral T-cell lymphoma. Journal of Clinical Oncology. 2021;39(15).

185. Moreno O, Wood J. Absorption, Distribution, and Binding Profile of ME-401, a Potent and Selective Oral Small-Molecule Inhibitor of Phosphatidylinositol 3-Kinase δ (PI3Kδ) in Animal and B-Cell Lymphoma Models. Target Oncol. 2019;14(5):603-11.

186. Moreno O, Butler T, Zann V, Willson A, Leung P, Connor A. Safety, Pharmacokinetics, and Pharmacodynamics of ME-401, an Oral, Potent, and Selective Inhibitor of Phosphatidylinositol 3-Kinase P110δ, Following Single Ascending Dose Administration to Healthy Volunteers. Clin Ther. 2018;40(11):1855-67.

187. Zelenetz AD, Soumerai JD, Jagadeesh D, Reddy N, Stathis A, Asch AS, et al. Preliminary safety and efficacy results with an intermittent schedule of the PI3kδ inhibitor ME-401 alone or in combination with rituximab for B-cell malignancies. (Abstract). Blood. 2018;132(1):2893.

188. Shin N, Stubbs M, Koblish H, Yue EW, Soloviev M, Douty B, et al. Parsaclisib Is a Next-Generation Phosphoinositide 3-Kinase. J Pharmacol Exp Ther. 2020;374(1):211-22.

189. Naing A, Powderly JD, Nemunaitis JJ, Luke JJ, Mansfield AS, Messersmith WA, et al. Exploring the safety, effect on the tumor microenvironment, and efficacy of itacitinib in combination with epacadostat or parsaclisib in advanced solid tumors: a phase I study. J Immunother Cancer. 2022;10(3).

190. Fukuhara N, Suehiro Y, Kato H, Kusumoto S, Coronado C, Rappold E, et al. Parsaclisib in Japanese patients with relapsed or refractory B-cell lymphoma (CITADEL-111): A phase Ib study. Cancer Sci. 2022;113(5):1702-11.

191. Forero-Torres A, Ramchandren R, Yacoub A, Wertheim MS, Edenfield WJ, Caimi P, et al. Parsaclisib, a potent and highly selective PI3Kδ inhibitor, in patients with relapsed or refractory B-cell malignancies. Blood. 2019;133(16):1742-52.

192. Coleman M, Belada D, Casasnovas RO, Gressin R, Lee HP, Mehta A, et al. Phase 2 study of parsaclisib (INCB050465), a highly selective, next-generation PI3Kδ inhibitor, in relapsed or refractory diffuse large B-cell lymphoma (CITADEL-202). Leuk Lymphoma. 2021;62(2):368-76.

193. Maharaj K, Powers JJ, Achille A, Mediavilla-Varela M, Gamal W, Burger KL, et al. The dual PI3Kδ/CK1ε inhibitor umbralisib exhibits unique immunomodulatory effects on CLL T cells. Blood Adv. 2020;4(13):3072-84.

194. Villaume MT, Arrate MP, Ramsey HE, Sunthankar KI, Jenkins MT, Moyo TK, et al. The delta isoform of phosphatidylinositol-3-kinase predominates in chronic myelomonocytic leukemia and can be targeted effectively with umbralisib and ruxolitinib. Exp Hematol. 2021;97:57-65.e5.

195. Dhillon S, Keam SJ. Umbralisib: First Approval. Drugs. 2021;81(7):857-66.

196. Burris HA, Flinn IW, Patel MR, Fenske TS, Deng C, Brander DM, et al. Umbralisib, a novel PI3Kδ and casein kinase-1ε inhibitor, in relapsed or refractory chronic lymphocytic leukaemia and lymphoma: an open-label, phase 1, dose-escalation, first-in-human study. Lancet Oncol. 2018;19(4):486-96.

197. Nastoupil LJ, Lunning MA, Vose JM, Schreeder MT, Siddiqi T, Flowers CR, et al. Tolerability and activity of ublituximab, umbralisib, and ibrutinib in patients with chronic lymphocytic leukaemia and non-Hodgkin lymphoma: a phase 1 dose escalation and expansion trial. Lancet Haematol. 2019;6(2):e100-e9.

198. Davids MS, Kim HT, Nicotra A, Savell A, Francoeur K, Hellman JM, et al. Umbralisib in combination with ibrutinib in patients with relapsed or refractory chronic lymphocytic leukaemia or mantle cell lymphoma: a multicentre phase 1-1b study. Lancet Haematol. 2019;6(1):e38-e47.

199. Lunning M, Vose J, Nastoupil L, Fowler N, Burger JA, Wierda WG, et al. Ublituximab and umbralisib in relapsed/refractory B-cell non-Hodgkin lymphoma and chronic lymphocytic leukemia. Blood. 2019;134(21):1811-20.

200. Mato AR, Ghosh N, Schuster SJ, Lamanna N, Pagel JM, Flinn IW, et al. Phase 2 study of the safety and efficacy of umbralisib in patients with CLL who are intolerant to BTK or PI3Kδ inhibitor therapy. Blood. 2021;137(20):2817-26.

201. Chavez JC, Goldschmidt N, Samaniego F, Wrobel T, Cavallo F, Fonseca G, et al. The Combination of Umbralisib Plus Ublituximab Is Active in Patients with Relapsed or Refractory Marginal Zone Lymphoma (MZL): Results from the Phase 2 Global Unity-NHL Trial. (Abstract). Blood. 2021;138(45).

202. Fowler NH, Samaniego F, Jurczak W, Ghosh N, Derenzini E, Reeves JA, et al. Umbralisib, a Dual PI3Kδ/CK1ε Inhibitor in Patients With Relapsed or Refractory Indolent Lymphoma. J Clin Oncol. 2021;39(15):1609-18.

203. Jang DK, Lee YG, Chan Chae Y, Lee JK, Paik WH, Lee SH, et al. GDC-0980 (apitolisib) treatment with gemcitabine and/or cisplatin synergistically reduces cholangiocarcinoma cell growth by suppressing the PI3K/Akt/mTOR pathway. Biochem Biophys Res Commun. 2020;529(4):1242-8.

204. Rahmani M, Nkwocha J, Hawkins E, Pei X, Parker RE, Kmieciak M, et al. Cotargeting BCL-2 and PI3K Induces BAX-Dependent Mitochondrial Apoptosis in AML Cells. Cancer Res. 2018;78(11):3075-86.

205. Dolly SO, Wagner AJ, Bendell JC, Kindler HL, Krug LM, Seiwert TY, et al. Phase I Study of Apitolisib (GDC-0980), Dual Phosphatidylinositol-3-Kinase and Mammalian Target of Rapamycin Kinase Inhibitor, in Patients with Advanced Solid Tumors. Clin Cancer Res. 2016;22(12):2874-84.

206. Powles T, Lackner MR, Oudard S, Escudier B, Ralph C, Brown JE, et al. Randomized Open-Label Phase II Trial of Apitolisib (GDC-0980), a Novel Inhibitor of the PI3K/Mammalian Target of Rapamycin Pathway, Versus Everolimus in Patients With Metastatic Renal Cell Carcinoma. J Clin Oncol. 2016;34(14):1660-8.

207. Simioni C, Cani A, Martelli AM, Zauli G, Alameen AA, Ultimo S, et al. The novel dual PI3K/mTOR inhibitor NVP-BGT226 displays cytotoxic activity in both normoxic and hypoxic hepatocarcinoma cells. Oncotarget. 2015;6(19):17147-60.

208. Guo Y, Zhu H, Weng M, Zhang H, Wang C, Sun L. CC-223, NSC781406, and BGT226 Exerts a Cytotoxic Effect Against Pancreatic Cancer Cells via mTOR Signaling. Front Pharmacol. 2020;11:580407.

209. Katanasaka Y, Kodera Y, Yunokawa M, Kitamura Y, Tamura T, Koizumi F. Synergistic anti-tumor effects of a novel phosphatidyl inositol-3 kinase/mammalian target of rapamycin dual inhibitor BGT226 and gefitinib in non-small cell lung cancer cell lines. Cancer Lett. 2014;347(2):196-203.

210. Tarantelli C, Gaudio E, Arribas AJ, Kwee I, Hillmann P, Rinaldi A, et al. PQR309 Is a Novel Dual PI3K/mTOR Inhibitor with Preclinical Antitumor Activity in Lymphomas as a Single Agent and in Combination Therapy. Clin Cancer Res. 2018;24(1):120-9.

211. Yang K, Tang XJ, Xu FF, Liu JH, Tan YQ, Gao L, et al. PI3K/mTORC1/2 inhibitor PQR309 inhibits proliferation and induces apoptosis in human glioblastoma cells. Oncol Rep. 2020;43(3):773-82.

212. von Achenbach C, Weller M, Kaulich K, Gramatzki D, Zacher A, Fabbro D, et al. Synergistic growth inhibition mediated by dual PI3K/mTOR pathway targeting and genetic or direct pharmacological AKT inhibition in human glioblastoma models. J Neurochem. 2020;153(4):510-24.

213. V. S, L. S, P. T, T. M, C. P, J.N. M, et al. Identification of NOTCH1 inactivating mutation as a therapeutic vulnerability to PI3K/mTOR pathway inhibition in head and neck squamous cell carcinoma (HNSCC). (Abstract). Cancer Res. 2017;77(13).

214. Beaufils F, Cmiljanovic N, Cmiljanovic V, Bohnacker T, Melone A, Marone R, et al. 5-(4,6-Dimorpholino-1,3,5-triazin-2-yl)-4-(trifluoromethyl)pyridin-2-amine (PQR309), a Potent, Brain-Penetrant, Orally Bioavailable, Pan-Class I PI3K/mTOR Inhibitor as Clinical Candidate in Oncology. J Med Chem. 2017;60(17):7524-38.

215. Wicki A, Brown N, Xyrafas A, Bize V, Hawle H, Berardi S, et al. First-in human, phase 1, dose-escalation pharmacokinetic and pharmacodynamic study of the oral dual PI3K and mTORC1/2 inhibitor PQR309 in patients with advanced solid tumors (SAKK 67/13). Eur J Cancer. 2018;96:6-16.

216. Collins GP, Eyre TA, Schmitz-Rohmer D, Townsend W, Popat R, Giulino-Roth L, et al. A Phase II Study to Assess the Safety and Efficacy of the Dual mTORC1/2 and PI3K Inhibitor Bimiralisib (PQR309) in Relapsed, Refractory Lymphoma. Hemasphere. 2021;5(11):e656.

217. Maira SM, Stauffer F, Brueggen J, Furet P, Schnell C, Fritsch C, et al. Identification and characterization of NVP-BEZ235, a new orally available dual phosphatidylinositol 3-kinase/mammalian target of rapamycin inhibitor with potent in vivo antitumor activity. Mol Cancer Ther. 2008;7(7):1851-63.

218. Cai J, Xia J, Zou J, Wang Q, Ma Q, Sun R, et al. The PI3K/mTOR dual inhibitor NVP-BEZ235 stimulates mutant p53 degradation to exert anti-tumor effects on triple-negative breast cancer cells. FEBS Open Bio. 2020;10(4):535-45.

219. Ruan B, Liu W, Chen P, Cui R, Li Y, Ji M, et al. NVP-BEZ235 inhibits thyroid cancer growth by p53- dependent/independent p21 upregulation. Int J Biol Sci. 2020;16(4):682-93.

220. Shi F, Zhang J, Liu H, Wu L, Jiang H, Wu Q, et al. The dual PI3K/mTOR inhibitor dactolisib elicits anti-tumor activity in vitro and in vivo. Oncotarget. 2018;9(1):706-17.

221. Helmy MW, Ghoneim AI, Katary MA, Elmahdy RK. The synergistic anti-proliferative effect of the combination of diosmin and BEZ-235 (dactolisib) on the HCT-116 colorectal cancer cell line occurs through inhibition of the PI3K/Akt/mTOR/NF-κB axis. Mol Biol Rep. 2020;47(3):2217-30.

222. Yu CC, Huang SY, Chang SF, Liao KF, Chiu SC. The Synergistic Anti-Cancer Effects of NVP-BEZ235 and Regorafenib in Hepatocellular Carcinoma. Molecules. 2020;25(10).

223. Yang X, Niu B, Wang L, Chen M, Kang X, Ji Y, et al. Autophagy inhibition enhances colorectal cancer apoptosis induced by dual phosphatidylinositol 3-kinase/mammalian target of rapamycin inhibitor NVP-BEZ235. Oncol Lett. 2016;12(1):102-6.

224. Alqurashi N, Hashimi SM, Alowaidi F, Ivanovski S, Wei MQ. Dual mTOR/PI3K inhibitor NVP‑BEZ235 arrests colorectal cancer cell growth and displays differential inhibition of 4E‑BP1. Oncol Rep. 2018;40(2):1083-92.

225. Carlo MI, Molina AM, Lakhman Y, Patil S, Woo K, DeLuca J, et al. A Phase Ib Study of BEZ235, a Dual Inhibitor of Phosphatidylinositol 3-Kinase (PI3K) and Mammalian Target of Rapamycin (mTOR), in Patients With Advanced Renal Cell Carcinoma. Oncologist. 2016;21(7):787-8.

226. Liu G, Jin Z, Lu X. Differential Targeting of Gr-MDSCs, T Cells and Prostate Cancer Cells by Dactolisib and Dasatinib. Int J Mol Sci. 2020;21(7).

227. Wu YY, Wu HC, Wu JE, Huang KY, Yang SC, Chen SX, et al. The dual PI3K/mTOR inhibitor BEZ235 restricts the growth of lung cancer tumors regardless of EGFR status, as a potent accompanist in combined therapeutic regimens. J Exp Clin Cancer Res. 2019;38(1):282.

228. Wang Y, Yu Q, He X, Romigh T, Altemus J, Eng C. Activation of AR sensitizes breast carcinomas to NVP-BEZ235's therapeutic effect mediated by PTEN and KLLN upregulation. Mol Cancer Ther. 2014;13(2):517-27.

229. Chen L, Jin T, Zhu K, Piao Y, Quan T, Quan C, et al. PI3K/mTOR dual inhibitor BEZ235 and histone deacetylase inhibitor Trichostatin A synergistically exert anti-tumor activity in breast cancer. Oncotarget. 2017;8(7):11937-49.

230. Calero R, Morchon E, Martinez-Argudo I, Serrano R. Synergistic anti-tumor effect of 17AAG with the PI3K/mTOR inhibitor NVP-BEZ235 on human melanoma. Cancer Lett. 2017;406:1-11.

231. Au KM, Wang AZ, Park SI. Pretargeted delivery of PI3K/mTOR small-molecule inhibitor-loaded nanoparticles for treatment of non-Hodgkin's lymphoma. Sci Adv. 2020;6(14):eaaz9798.

232. Kudoh A, Oishi T, Itamochi H, Sato S, Naniwa J, Shimada M, et al. Dual inhibition of phosphatidylinositol 3'-kinase and mammalian target of rapamycin using NVP-BEZ235 as a novel therapeutic approach for mucinous adenocarcinoma of the ovary. Int J Gynecol Cancer. 2014;24(3):444-53.

233. Massard C, Chi KN, Castellano D, de Bono J, Gravis G, Dirix L, et al. Phase Ib dose-finding study of abiraterone acetate plus buparlisib (BKM120) or dactolisib (BEZ235) in patients with castration-resistant prostate cancer. Eur J Cancer. 2017;76:36-44.

234. Rodon J, Pérez-Fidalgo A, Krop IE, Burris H, Guerrero-Zotano A, Britten CD, et al. Phase 1/1b dose escalation and expansion study of BEZ235, a dual PI3K/mTOR inhibitor, in patients with advanced solid tumors including patients with advanced breast cancer. Cancer Chemother Pharmacol. 2018;82(2):285-98.

235. Wei XX, Hsieh AC, Kim W, Friedlander T, Lin AM, Louttit M, et al. A Phase I Study of Abiraterone Acetate Combined with BEZ235, a Dual PI3K/mTOR Inhibitor, in Metastatic Castration Resistant Prostate Cancer. Oncologist. 2017;22(5):503-e43.

236. Bendell JC, Kurkjian C, Infante JR, Bauer TM, Burris HA, Greco FA, et al. A phase 1 study of the sachet formulation of the oral dual PI3K/mTOR inhibitor BEZ235 given twice daily (BID) in patients with advanced solid tumors. Invest New Drugs. 2015;33(2):463-71.

237. Toyoda M, Watanabe K, Amagasaki T, Natsume K, Takeuchi H, Quadt C, et al. A phase I study of single-agent BEZ235 special delivery system sachet in Japanese patients with advanced solid tumors. Cancer Chemother Pharmacol. 2019;83(2):289-99.

238. Kashiyama T, Oda K, Ikeda Y, Shiose Y, Hirota Y, Inaba K, et al. Antitumor activity and induction of TP53-dependent apoptosis toward ovarian clear cell adenocarcinoma by the dual PI3K/mTOR inhibitor DS-7423. PLoS One. 2014;9(2):e87220.

239. Makii C, Ikeda Y, Oda K, Uehara Y, Nishijima A, Koso T, et al. Anti-tumor activity of dual inhibition of phosphatidylinositol 3-kinase and MDM2 against clear cell ovarian carcinoma. Gynecol Oncol. 2019;155(2):331-9.

240. Koul D, Wang S, Wu S, Saito N, Zheng S, Gao F, et al. Preclinical therapeutic efficacy of a novel blood-brain barrier-penetrant dual PI3K/mTOR inhibitor with preferential response in PI3K/PTEN mutant glioma. Oncotarget. 2017;8(13):21741-53.

241. Yokota T, Bendell J, LoRusso P, Tsushima T, Desai V, Kenmotsu H, et al. Impact of race on dose selection of molecular-targeted agents in early-phase oncology trials. Br J Cancer. 2018;118(12):1571-9.

242. Soares HP, Ming M, Mellon M, Young SH, Han L, Sinnet-Smith J, et al. Dual PI3K/mTOR Inhibitors Induce Rapid Overactivation of the MEK/ERK Pathway in Human Pancreatic Cancer Cells through Suppression of mTORC2. Mol Cancer Ther. 2015;14(4):1014-23.

243. Freitag H, Christen F, Lewens F, Grass I, Briest F, Iwaszkiewicz S, et al. Inhibition of mTOR's Catalytic Site by PKI-587 Is a Promising Therapeutic Option for Gastroenteropancreatic Neuroendocrine Tumor Disease. Neuroendocrinology. 2017;105(1):90-104.

244. Wilson GD, Wilson TG, Hanna A, Dabjan M, Buelow K, Torma J, et al. Dacomitinib and gedatolisib in combination with fractionated radiation in head and neck cancer. Clin Transl Radiat Oncol. 2021;26:15-23.

245. Leiker AJ, DeGraff W, Choudhuri R, Sowers AL, Thetford A, Cook JA, et al. Radiation Enhancement of Head and Neck Squamous Cell Carcinoma by the Dual PI3K/mTOR Inhibitor PF-05212384. Clin Cancer Res. 2015;21(12):2792-801.

246. Liu C, Xing W, Yu H, Zhang W, Si T. ABCB1 and ABCG2 restricts the efficacy of gedatolisib (PF-05212384), a PI3K inhibitor in colorectal cancer cells. Cancer Cell Int. 2021;21(1):108.

247. Zhang Y, Xie C, Li A, Liu X, Xing Y, Shen J, et al. PKI-587 enhances chemosensitivity of oxaliplatin in hepatocellular carcinoma through suppressing DNA damage repair pathway (NHEJ and HR) and PI3K/AKT/mTOR pathway. Am J Transl Res. 2019;11(8):5134-49.

248. Shor RE, Dai J, Lee SY, Pisarsky L, Matei I, Lucotti S, et al. The PI3K/mTOR inhibitor Gedatolisib eliminates dormant breast cancer cells in organotypic culture, but fails to prevent metastasis in preclinical settings. Mol Oncol. 2021.

249. Brana I, Pham NA, Kim L, Sakashita S, Li M, Ng C, et al. Novel combinations of PI3K-mTOR inhibitors with dacomitinib or chemotherapy in PTEN-deficient patient-derived tumor xenografts. Oncotarget. 2017;8(49):84659-70.

250. Langdon SP, Kay C, Um IH, Dodds M, Muir M, Sellar G, et al. Evaluation of the dual mTOR/PI3K inhibitors Gedatolisib (PF-05212384) and PF-04691502 against ovarian cancer xenograft models. Sci Rep. 2019;9(1):18742.

251. Shapiro GI, Bell-McGuinn KM, Molina JR, Bendell J, Spicer J, Kwak EL, et al. First-in-Human Study of PF-05212384 (PKI-587), a Small-Molecule, Intravenous, Dual Inhibitor of PI3K and mTOR in Patients with Advanced Cancer. Clin Cancer Res. 2015;21(8):1888-95.

252. Radovich M, Solzak JP, Wang CJ, Hancock BA, Badve SS, Althouse SK, et al. Initial phase I safety study of gedatolisib plus cofetuzumab pelidotin for patients with metastatic triple-negative breast cancer. Clin Cancer Res. 2022.

253. Wainberg ZA, Alsina M, Soares HP, Braña I, Britten CD, Del Conte G, et al. A Multi-Arm Phase I Study of the PI3K/mTOR Inhibitors PF-04691502 and Gedatolisib (PF-05212384) plus Irinotecan or the MEK Inhibitor PD-0325901 in Advanced Cancer. Target Oncol. 2017;12(6):775-85.

254. Colombo I, Genta S, Martorana F, Guidi M, Frattini M, Samartzis EP, et al. Phase I Dose-Escalation Study of the Dual PI3K-mTORC1/2 Inhibitor Gedatolisib in Combination with Paclitaxel and Carboplatin in Patients with Advanced Solid Tumors. Clin Cancer Res. 2021;27(18):5012-9.

255. Del Campo JM, Birrer M, Davis C, Fujiwara K, Gollerkeri A, Gore M, et al. A randomized phase II non-comparative study of PF-04691502 and gedatolisib (PF-05212384) in patients with recurrent endometrial cancer. Gynecol Oncol. 2016;142(1):62-9.

256. Xiao Y, Yu Y, Jiang P, Li Y, Wang C, Zhang R. The PI3K/mTOR dual inhibitor GSK458 potently impedes ovarian cancer tumorigenesis and metastasis. Cell Oncol (Dordr). 2020;43(4):669-80.

257. Du J, Chen F, Yu J, Jiang L, Zhou M. The PI3K/mTOR Inhibitor Ompalisib Suppresses Nonhomologous End Joining and Sensitizes Cancer Cells to Radio- and Chemotherapy. Mol Cancer Res. 2021.

258. Feng Y, Jiang Y, Hao F. GSK2126458 has the potential to inhibit the proliferation of pancreatic cancer uncovered by bioinformatics analysis and pharmacological experiments. J Transl Med. 2021;19(1):373.

259. Álvarez RM, García AB, Riesco-Fagundo C, Martín JI, Varela C, Rodríguez Hergueta A, et al. Omipalisib inspired macrocycles as dual PI3K/mTOR inhibitors. Eur J Med Chem. 2021;211:113109.

260. Liu T, Sun Q, Li Q, Yang H, Zhang Y, Wang R, et al. Dual PI3K/mTOR inhibitors, GSK2126458 and PKI-587, suppress tumor progression and increase radiosensitivity in nasopharyngeal carcinoma. Mol Cancer Ther. 2015;14(2):429-39.

261. Narov K, Yang J, Samsel P, Jones A, Sampson JR, Shen MH. The dual PI3K/mTOR inhibitor GSK2126458 is effective for treating solid renal tumours in Tsc2+/- mice through suppression of cell proliferation and induction of apoptosis. Oncotarget. 2017;8(35):58504-12.

262. Munster P, Aggarwal R, Hong D, Schellens JH, van der Noll R, Specht J, et al. First-in-Human Phase I Study of GSK2126458, an Oral Pan-Class I Phosphatidylinositol-3-Kinase Inhibitor, in Patients with Advanced Solid Tumor Malignancies. Clin Cancer Res. 2016;22(8):1932-9.

263. Venkatesha VA, Joshi A, Venkataraman M, Sonawane V, Bhatia D, Tannu P, et al. P7170, a novel inhibitor of mTORC1/mTORC2 and Activin receptor-like Kinase 1 (ALK1) inhibits the growth of non small cell lung cancer. Mol Cancer. 2014;13:259.

264. Bean JR, Hosford SR, Symonds LK, Owens P, Dillon LM, Yang W, et al. The PI3K/mTOR dual inhibitor P7170 demonstrates potent activity against endocrine-sensitive and endocrine-resistant ER+ breast cancer. Breast Cancer Res Treat. 2015;149(1):69-79.

265. Jalota-Badhwar A, Bhatia DR, Boreddy S, Joshi A, Venkatraman M, Desai N, et al. P7170: A Novel Molecule with Unique Profile of mTORC1/C2 and Activin Receptor-like Kinase 1 Inhibition Leading to Antitumor and Antiangiogenic Activity. Mol Cancer Ther. 2015;14(5):1095-106.

266. Ding LT, Zhao P, Yang ML, Lv GZ, Zhao TL. GDC-0084 inhibits cutaneous squamous cell carcinoma cell growth. Biochem Biophys Res Commun. 2018;503(3):1941-8.

267. Ippen FM, Alvarez-Breckenridge CA, Kuter BM, Fink AL, Bihun IV, Lastrapes M, et al. The dual PI3K/mTOR-pathway inhibitor GDC-0084 achieves antitumor activity in PIK3CA-mutant breast cancer brain metastases. Clin Cancer Res. 2019;25(11):3374-83.

268. Wen PY, Cloughesy TF, Olivero A, Lu X, Mueller L, Coimbra AF, et al. A first-in-human phase 1 study to evaluate the brain-penetrant PI3K/mTOR inhibitor GDC-0084 in patients with progressive or recurrent high-grade glioma. (Abstract). J Clin Oncol. 2016;34(15).

269. Wen PY, Cloughesy TF, Olivero AG, Morrissey KM, Wilson TR, Lu X, et al. First-in-Human Phase I Study to Evaluate the Brain-Penetrant PI3K/mTOR Inhibitor GDC-0084 in Patients with Progressive or Recurrent High-Grade Glioma. Clin Cancer Res. 2020;26(8):1820-8.

270. Yuan J, Mehta PP, Yin MJ, Sun S, Zou A, Chen J, et al. PF-04691502, a potent and selective oral inhibitor of PI3K and mTOR kinases with antitumor activity. Mol Cancer Ther. 2011;10(11):2189-99.

271. Chen D, Mao C, Zhou Y, Su Y, Liu S, Qi WQ. PF-04691502, a dual PI3K/mTOR inhibitor has potent pre-clinical activity by inducing apoptosis and G1 cell cycle arrest in aggressive B-cell non-Hodgkin lymphomas. Int J Oncol. 2016;48(1):253-60.

272. Blunt MD, Carter MJ, Larrayoz M, Smith LD, Aguilar-Hernandez M, Cox KL, et al. The PI3K/mTOR inhibitor PF-04691502 induces apoptosis and inhibits microenvironmental signaling in CLL and the Eµ-TCL1 mouse model. Blood. 2015;125(26):4032-41.

273. Tonlaar N, Galoforo S, Thibodeau BJ, Ahmed S, Wilson TG, Yumpo Cardenas P, et al. Antitumor activity of the dual PI3K/MTOR inhibitor, PF-04691502, in combination with radiation in head and neck cancer. Radiother Oncol. 2017;124(3):504-12.

274. Chow Z, Johnson J, Chauhan A, Izumi T, Cavnar M, Weiss H, et al. PI3K/mTOR Dual Inhibitor PF-04691502 Is a Schedule-Dependent Radiosensitizer for Gastroenteropancreatic Neuroendocrine Tumors. Cells. 2021;10(5).

275. Bresin A, Cristofoletti C, Caprini E, Cantonetti M, Monopoli A, Russo G, et al. Preclinical Evidence for Targeting PI3K/mTOR Signaling with Dual-Inhibitors as a Therapeutic Strategy against Cutaneous T-Cell Lymphoma. J Invest Dermatol. 2020;140(5):1045-53.e6.

276. Herzog A, Bian Y, Vander Broek R, Hall B, Coupar J, Cheng H, et al. PI3K/mTOR inhibitor PF-04691502 antitumor activity is enhanced with induction of wild-type TP53 in human xenograft and murine knockout models of head and neck cancer. Clin Cancer Res. 2013;19(14):3808-19.

277. Raynaud FI, Eccles S, Clarke PA, Hayes A, Nutley B, Alix S, et al. Pharmacologic characterization of a potent inhibitor of class I phosphatidylinositide 3-kinases. Cancer Res. 2007;67(12):5840-50.

278. Park S, Chapuis N, Bardet V, Tamburini J, Gallay N, Willems L, et al. PI-103, a dual inhibitor of Class IA phosphatidylinositide 3-kinase and mTOR, has antileukemic activity in AML. Leukemia. 2008;22(9):1698-706.

279. Mishra VS, Kumar N, Raza M, Sehrawat S. Amalgamation of PI3K and EZH2 blockade synergistically regulates invasion and angiogenesis: combination therapy for glioblastoma multiforme. Oncotarget. 2020;11(51):4754-69.

280. Westhoff MA, Kandenwein JA, Karl S, Vellanki SH, Braun V, Eramo A, et al. The pyridinylfuranopyrimidine inhibitor, PI-103, chemosensitizes glioblastoma cells for apoptosis by inhibiting DNA repair. Oncogene. 2009;28(40):3586-96.

281. Fan QW, Knight ZA, Goldenberg DD, Yu W, Mostov KE, Stokoe D, et al. A dual PI3 kinase/mTOR inhibitor reveals emergent efficacy in glioma. Cancer Cell. 2006;9(5):341-9.

282. Opel D, Naumann I, Schneider M, Bertele D, Debatin KM, Fulda S. Targeting aberrant PI3K/Akt activation by PI103 restores sensitivity to TRAIL-induced apoptosis in neuroblastoma. Clin Cancer Res. 2011;17(10):3233-47.

283. Jang NY, Kim DH, Cho BJ, Choi EJ, Lee JS, Wu HG, et al. Radiosensitization with combined use of olaparib and PI-103 in triple-negative breast cancer. BMC Cancer. 2015;15:89.

284. Geng X, Xie L, Xing H. PI3K Inhibitor Combined With Chemotherapy Can Enhance the Apoptosis of Neuroblastoma Cells In Vitro and In Vivo. Technol Cancer Res Treat. 2016;15(5):716-22.

285. Wu CP, Hung CY, Lusvarghi S, Huang YH, Tseng PJ, Hung TH, et al. Overexpression of ABCB1 and ABCG2 contributes to reduced efficacy of the PI3K/mTOR inhibitor samotolisib (LY3023414) in cancer cell lines. Biochem Pharmacol. 2020;180:114137.

286. Chen X, Chen W, Aung ZM, Han W, Zhang Y, Chai G. LY3023414 inhibits both osteogenesis and osteoclastogenesis through the PI3K/Akt/GSK3 signalling pathway. Bone Joint Res. 2021;10(4):237-49.

287. Zauderer MG, Alley EW, Bendell J, Capelletto E, Bauer TM, Callies S, et al. Phase 1 cohort expansion study of LY3023414, a dual PI3K/mTOR inhibitor, in patients with advanced mesothelioma. Invest New Drugs. 2021;39(4):1081-8.

288. Bendell JC, Varghese AM, Hyman DM, Bauer TM, Pant S, Callies S, et al. A First-in-Human Phase 1 Study of LY3023414, an Oral PI3K/mTOR Dual Inhibitor, in Patients with Advanced Cancer. Clin Cancer Res. 2018;24(14):3253-62.

289. Kondo S, Tajimi M, Funai T, Inoue K, Asou H, Ranka VK, et al. Phase 1 dose-escalation study of a novel oral PI3K/mTOR dual inhibitor, LY3023414, in patients with cancer. Invest New Drugs. 2020;38(6):1836-45.

290. Hong DS, Moore KN, Bendell JC, Karp DD, Wang JS, Ulahannan SV, et al. Preclinical Evaluation and Phase Ib Study of Prexasertib, a CHK1 Inhibitor, and Samotolisib (LY3023414), a Dual PI3K/mTOR Inhibitor. Clin Cancer Res. 2021;27(7):1864-74.

291. Sweeney CJ, Percent IJ, Babu S, Cultrera JL, Mehlhaff BA, Goodman OB, et al. Phase Ib/II Study of Enzalutamide with Samotolisib (LY3023414) or Placebo in Patients with Metastatic Castration-Resistant Prostate Cancer. Clin Cancer Res. 2022;28(11):2237-47.

292. Rubinstein MM, Hyman DM, Caird I, Won H, Soldan K, Seier K, et al. Phase 2 study of LY3023414 in patients with advanced endometrial cancer harboring activating mutations in the PI3K pathway. Cancer. 2020;126(6):1274-82.

293. Yu P, Laird AD, Du X, Wu J, Won KA, Yamaguchi K, et al. Characterization of the activity of the PI3K/mTOR inhibitor XL765 (SAR245409) in tumor models with diverse genetic alterations affecting the PI3K pathway. Mol Cancer Ther. 2014;13(5):1078-91.

294. Gravina GL, Mancini A, Scarsella L, Colapietro A, Jitariuc A, Vitale F, et al. Dual PI3K/mTOR inhibitor, XL765 (SAR245409), shows superior effects to sole PI3K [XL147 (SAR245408)] or mTOR [rapamycin] inhibition in prostate cancer cell models. Tumour Biol. 2016;37(1):341-51.

295. Zhao H, Chen G, Liang H. Dual PI3K/mTOR Inhibitor, XL765, suppresses glioblastoma growth by inducing ER stress-dependent apoptosis. Onco Targets Ther. 2019;12:5415-24.

296. Cloughesy TF, Mischel PS, Omuro AMP, Prados M, Wen PY, Wu B, et al. Tumor pharmacokinetics (PK) and pharmacodynamics (PD) of SAR245409 (XL765) and SAR245408 (XL147) administered as single agents to patients with recurrent glioblastoma (GBM): An Ivy Foundation early-phase clinical trials consortium study. (Abstract). J Clin Oncol. 2013;31(15).

297. Papadopoulos KP, Tabernero J, Markman B, Patnaik A, Tolcher AW, Baselga J, et al. Phase I safety, pharmacokinetic, and pharmacodynamic study of SAR245409 (XL765), a novel, orally administered PI3K/mTOR inhibitor in patients with advanced solid tumors. Clin Cancer Res. 2014;20(9):2445-56.

298. Mehnert JM, Edelman G, Stein M, Camisa H, Lager J, Dedieu JF, et al. A phase I dose-escalation study of the safety and pharmacokinetics of a tablet formulation of voxtalisib, a phosphoinositide 3-kinase inhibitor, in patients with solid tumors. Invest New Drugs. 2018;36(1):36-44.

299. Jänne PA, Cohen RB, Laird AD, Macé S, Engelman JA, Ruiz-Soto R, et al. Phase I safety and pharmacokinetic study of the PI3K/mTOR inhibitor SAR245409 (XL765) in combination with erlotinib in patients with advanced solid tumors. J Thorac Oncol. 2014;9(3):316-23.

300. Wen PY, Omuro A, Ahluwalia MS, Fathallah-Shaykh HM, Mohile N, Lager JJ, et al. Phase I dose-escalation study of the PI3K/mTOR inhibitor voxtalisib (SAR245409, XL765) plus temozolomide with or without radiotherapy in patients with high-grade glioma. Neuro Oncol. 2015;17(9):1275-83.

301. Awan FT, Gore L, Gao L, Sharma J, Lager J, Costa LJ. Phase Ib trial of the PI3K/mTOR inhibitor voxtalisib (SAR245409) in combination with chemoimmunotherapy in patients with relapsed or refractory B-cell malignancies. Br J Haematol. 2016;175(1):55-65.

302. Brown JR, Hamadani M, Hayslip J, Janssens A, Wagner-Johnston N, Ottmann O, et al. Voxtalisib (XL765) in patients with relapsed or refractory non-Hodgkin lymphoma or chronic lymphocytic leukaemia: an open-label, phase 2 trial. Lancet Haematol. 2018;5(4):e170-e80.

303. Kolev VN, Wright QG, Vidal CM, Ring JE, Shapiro IM, Ricono J, et al. PI3K/mTOR dual inhibitor VS-5584 preferentially targets cancer stem cells. Cancer Res. 2015;75(2):446-55.

304. Mustafa N, Ting Lee JX, Adina Nee HF, Bi C, Chung TH, Hart S, et al. VS-5584 mediates potent anti-myeloma activity via the upregulation of a class II tumor suppressor gene, RARRES3 and the activation of Bim. Oncotarget. 2017;8(60):101847-64.

305. Sun JY, Hou YJ, Cui HJ, Zhang C, Yang MF, Wang FZ, et al. VS-5584 Inhibits Human Osteosarcoma Cells Growth by Induction of G1- phase Arrest through Regulating PI3K/mTOR and MAPK Pathways. Curr Cancer Drug Targets. 2020;20(8):616-23.

306. Toosi B, Zaker F, Alikarami F, Kazemi A, Teremmahi Ardestanii M. VS-5584 as a PI3K/mTOR inhibitor enhances apoptotic effects of subtoxic dose arsenic trioxide via inhibition of NF-κB activity in B cell precursor-acute lymphoblastic leukemia. Biomed Pharmacother. 2018;102:428-37.

307. Kayabasi C, Yelken BO, Asik A, Okcanoglu TB, Sogutlu F, Gasimli R, et al. PI3K/mTOR dual-inhibition with VS-5584 enhances anti-leukemic efficacy of ponatinib in blasts and Ph-negative LSCs of chronic myeloid leukemia. Eur J Pharmacol. 2021;910:174446.

308. Xu M, Xu L, Wang Y, Dai G, Xue B, Liu YY, et al. BRD4 inhibition sensitizes renal cell carcinoma cells to the PI3K/mTOR dual inhibitor VS-5584. Aging (Albany NY). 2020;12(19):19147-58.

309. Chen Y, Tsai HW, Tsai YH, Tseng SH. VS-5584, a PI3K/mTOR dual inhibitor, exerts antitumor effects on neuroblastomas in vitro and in vivo. J Pediatr Surg. 2021;56(8):1441-8.

310. Spencer A, Yoon SS, Harrison SJ, Morris SR, Smith DA, Brigandi RA, et al. The novel AKT inhibitor afuresertib shows favorable safety, pharmacokinetics, and clinical activity in multiple myeloma. Blood. 2014;124(14):2190-5.

311. Wang J, Xu X, Wang T, Guo Q, Dai X, Guo H, et al. Ceritinib increases sensitivity of AKT inhibitors to gastric cancer. Eur J Pharmacol. 2021;896:173879.

312. Yamaji M, Ota A, Wahiduzzaman M, Karnan S, Hyodo T, Konishi H, et al. Novel ATP-competitive Akt inhibitor afuresertib suppresses the proliferation of malignant pleural mesothelioma cells. Cancer Med. 2017;6(11):2646-59.

313. Zhou H, Ning Y, Zeng G, Zhou C, Ding X. Curcumin promotes cell cycle arrest and apoptosis of acute myeloid leukemia cells by inactivating AKT. Oncol Rep. 2021;45(4).

314. Tolcher AW, Patnaik A, Papadopoulos KP, Rasco DW, Becerra CR, Allred AJ, et al. Phase I study of the MEK inhibitor trametinib in combination with the AKT inhibitor afuresertib in patients with solid tumors and multiple myeloma. Cancer Chemother Pharmacol. 2015;75(1):183-9.

315. Blagden SP, Hamilton AL, Mileshkin L, Wong S, Michael A, Hall M, et al. Phase IB Dose Escalation and Expansion Study of AKT Inhibitor Afuresertib with Carboplatin and Paclitaxel in Recurrent Platinum-resistant Ovarian Cancer. Clin Cancer Res. 2019;25(5):1472-8.

316. Chen CI, Paul H, Le LW, Wei EN, Snitzler S, Wang T, et al. A phase 2 study of ofatumumab (Arzerra®) in combination with a pan-AKT inhibitor (afuresertib) in previously treated patients with chronic lymphocytic leukemia (CLL). Leuk Lymphoma. 2019;60(1):92-100.

317. Yu Y, Savage RE, Eathiraj S, Meade J, Wick MJ, Hall T, et al. Targeting AKT1-E17K and the PI3K/AKT Pathway with an Allosteric AKT Inhibitor, ARQ 092. PLoS One. 2015;10(10):e0140479.

318. Rivera-Soto R, Yu Y, Dittmer DP, Damania B. Combined Inhibition of Akt and mTOR Is Effective Against Non-Hodgkin Lymphomas. Front Oncol. 2021;11:670275.

319. Kozinova M, Joshi S, Ye S, Belinsky MG, Sharipova D, Farma JM, et al. Combined Inhibition of AKT and KIT Restores Expression of Programmed Cell Death 4 (PDCD4) in Gastrointestinal Stromal Tumor. Cancers (Basel). 2021;13(15).

320. Politz O, Siegel F, Bärfacker L, Bömer U, Hägebarth A, Scott WJ, et al. BAY 1125976, a selective allosteric AKT1/2 inhibitor, exhibits high efficacy on AKT signaling-dependent tumor growth in mouse models. Int J Cancer. 2017;140(2):449-59.

321. Schneeweiss A, Hess D, Joerger M, Varga A, Moulder S, Tsimberidou AM, et al. Phase 1 Dose Escalation Study of the Allosteric AKT Inhibitor BAY 1125976 in Advanced Solid Cancer-Lack of Association between Activating AKT Mutation and AKT Inhibition-Derived Efficacy. Cancers (Basel). 2019;11(12).

322. Jones RH, Casbard A, Carucci M, Cox C, Butler R, Alchami F, et al. Fulvestrant plus capivasertib versus placebo after relapse or progression on an aromatase inhibitor in metastatic, oestrogen receptor-positive breast cancer (FAKTION): a multicentre, randomised, controlled, phase 2 trial. Lancet Oncol. 2020;21(3):345-57.

323. Gris-Oliver A, Palafox M, Monserrat L, Brasó-Maristany F, Òdena A, Sánchez-Guixé M, et al. Genetic Alterations in the PI3K/AKT Pathway and Baseline AKT Activity Define AKT Inhibitor Sensitivity in Breast Cancer Patient-derived Xenografts. Clin Cancer Res. 2020;26(14):3720-31.

324. Turner N. Capivasertib Doubles PFS in Some Breast Cancers. Cancer Discov. 2023;13(2):250.

325. Robertson JFR, Coleman RE, Cheung KL, Evans A, Holcombe C, Skene A, et al. Proliferation and AKT Activity Biomarker Analyses after Capivasertib (AZD5363) Treatment of Patients with ER. Clin Cancer Res. 2020;26(7):1574-85.

326. Banerji U, Dean EJ, Pérez-Fidalgo JA, Batist G, Bedard PL, You B, et al. A Phase I Open-Label Study to Identify a Dosing Regimen of the Pan-AKT Inhibitor AZD5363 for Evaluation in Solid Tumors and in PIK3CA-Mutated Breast and Gynecologic Cancers. Clin Cancer Res. 2018;24(9):2050-9.

327. Kalinsky K, Hong F, McCourt CK, Sachdev JC, Mitchell EP, Zwiebel JA, et al. Effect of Capivasertib in Patients With an AKT1 E17K-Mutated Tumor: NCI-MATCH Subprotocol EAY131-Y Nonrandomized Trial. JAMA Oncol. 2021;7(2):271-8.

328. Smyth LM, Tamura K, Oliveira M, Ciruelos EM, Mayer IA, Sablin MP, et al. Capivasertib, an AKT Kinase Inhibitor, as Monotherapy or in Combination with Fulvestrant in Patients with AKT1 E17K-Mutant, ER-Positive Metastatic Breast Cancer. Clin Cancer Res. 2020;26(15):3947-57.

329. Smyth LM, Batist G, Meric-Bernstam F, Kabos P, Spanggaard I, Lluch A, et al. Selective AKT kinase inhibitor capivasertib in combination with fulvestrant in PTEN-mutant ER-positive metastatic breast cancer. NPJ Breast Cancer. 2021;7(1):44.

330. Yap TA, Kristeleit R, Michalarea V, Pettitt SJ, Lim JSJ, Carreira S, et al. Phase I Trial of the PARP Inhibitor Olaparib and AKT Inhibitor Capivasertib in Patients with BRCA1/2- and Non- BRCA1/2-Mutant Cancers. Cancer Discov. 2020;10(10):1528-43.

331. Shore ND, Mellado B, Shah S, Hauke RJ, Costin D, Morris T, et al. A phase I study of capivasertib in combination with abiraterone acetate in patients with metastatic castration-resistant prostate cancer. Journal of Clinical Oncology. 2021;39(6).

332. Kolinsky MP, Rescigno P, Bianchini D, Zafeiriou Z, Mehra N, Mateo J, et al. A phase I dose-escalation study of enzalutamide in combination with the AKT inhibitor AZD5363 (capivasertib) in patients with metastatic castration-resistant prostate cancer. Ann Oncol. 2020;31(5):619-25.

333. Westin SN, Labrie M, Litton JK, Blucher A, Fang Y, Vellano CP, et al. Phase 1b dose expansion and translational analyses of olaparib in combination with capivasertib in recurrent endometrial, triple negative breast, and ovarian cancer. Clin Cancer Res. 2021.

334. Howell SJ, Casbard A, Carucci M, Ingarfield K, Butler R, Morgan S, et al. Fulvestrant plus capivasertib versus placebo after relapse or progression on an aromatase inhibitor in metastatic, oestrogen receptor-positive, HER2-negative breast cancer (FAKTION): overall survival, updated progression-free survival, and expanded biomarker analysis from a randomised, phase 2 trial. Lancet Oncol. 2022.

335. Turner NC, Alarcón E, Armstrong AC, Philco M, López Chuken YA, Sablin MP, et al. BEECH: a dose-finding run-in followed by a randomised phase II study assessing the efficacy of AKT inhibitor capivasertib (AZD5363) combined with paclitaxel in patients with estrogen receptor-positive advanced or metastatic breast cancer, and in a PIK3CA mutant sub-population. Ann Oncol. 2019;30(5):774-80.

336. Schmid P, Abraham J, Chan S, Wheatley D, Brunt AM, Nemsadze G, et al. Capivasertib Plus Paclitaxel Versus Placebo Plus Paclitaxel As First-Line Therapy for Metastatic Triple-Negative Breast Cancer: The PAKT Trial. J Clin Oncol. 2020;38(5):423-33.

337. Crabb SJ, Griffiths G, Marwood E, Dunkley D, Downs N, Martin K, et al. Pan-AKT Inhibitor Capivasertib With Docetaxel and Prednisolone in Metastatic Castration-Resistant Prostate Cancer: A Randomized, Placebo-Controlled Phase II Trial (ProCAID). J Clin Oncol. 2021;39(3):190-201.

338. Sun JY, Hou YJ, Yin YB, Wang FZ, Yang MF, Zhang YY, et al. CCT128930 induces G1-phase arrest and apoptosis and synergistically enhances the anticancer efficiency of VS5584 in human osteosarcoma cells. Biomed Pharmacother. 2020;130:110544.

339. Choi JI, Park SH, Lee HJ, Lee DW, Lee HN. Inhibition of Phospho-S6 Kinase, a Protein Involved in the Compensatory Adaptive Response, Increases the Efficacy of Paclitaxel in Reducing the Viability of Matrix-Attached Ovarian Cancer Cells. PLoS One. 2016;11(5):e0155052.

340. Kim KW, Kim JY, Qiao J, Clark RA, Powers CM, Correa H, et al. Dual-Targeting AKT2 and ERK in cancer stem-like cells in neuroblastoma. Oncotarget. 2019;10(54):5645-59.

341. Gener P, Rafael D, Seras-Franzoso J, Perez A, Pindado LA, Casas G, et al. Pivotal Role of AKT2 during Dynamic Phenotypic Change of Breast Cancer Stem Cells. Cancers (Basel). 2019;11(8).

342. Sun L, Huang Y, Liu Y, Zhao Y, He X, Zhang L, et al. Ipatasertib, a novel Akt inhibitor, induces transcription factor FoxO3a and NF-κB directly regulates PUMA-dependent apoptosis. Cell Death Dis. 2018;9(9):911.

343. Jabbarzadeh Kaboli P, Salimian F, Aghapour S, Xiang S, Zhao Q, Li M, et al. Akt-targeted therapy as a promising strategy to overcome drug resistance in breast cancer - A comprehensive review from chemotherapy to immunotherapy. Pharmacol Res. 2020;156:104806.

344. Laterza MM, Ciaramella V, Facchini BA, Franzese E, Liguori C, De Falco S, et al. Enhanced Antitumor Effect of Trastuzumab and Duligotuzumab or Ipatasertib Combination in HER-2 Positive Gastric Cancer Cells. Cancers (Basel). 2021;13(10).

345. Saura C, Roda D, Roselló S, Oliveira M, Macarulla T, Pérez-Fidalgo JA, et al. A First-in-Human Phase I Study of the ATP-Competitive AKT Inhibitor Ipatasertib Demonstrates Robust and Safe Targeting of AKT in Patients with Solid Tumors. Cancer Discov. 2017;7(1):102-13.

346. Isakoff SJ, Tabernero J, Molife LR, Soria JC, Cervantes A, Vogelzang NJ, et al. Antitumor activity of ipatasertib combined with chemotherapy: results from a phase Ib study in solid tumors. Ann Oncol. 2020;31(5):626-33.

347. Shapiro GI, LoRusso P, Cho DC, Musib L, Yan Y, Wongchenko M, et al. A phase Ib open-label dose escalation study of the safety, pharmacokinetics, and pharmacodynamics of cobimetinib (GDC-0973) and ipatasertib (GDC-0068) in patients with locally advanced or metastatic solid tumors. Invest New Drugs. 2021;39(1):163-74.

348. Shi Z, Wulfkuhle J, Nowicka M, Gallagher RI, Saura C, Nuciforo PG, et al. Functional Mapping of AKT Signaling and Biomarkers of Response from the FAIRLANE Trial of Neoadjuvant Ipatasertib plus Paclitaxel for Triple-Negative Breast Cancer. Clin Cancer Res. 2022;28(5):993-1003.

349. Kim SB, Dent R, Im SA, Espié M, Blau S, Tan AR, et al. Ipatasertib plus paclitaxel versus placebo plus paclitaxel as first-line therapy for metastatic triple-negative breast cancer (LOTUS): a multicentre, randomised, double-blind, placebo-controlled, phase 2 trial. Lancet Oncol. 2017;18(10):1360-72.

350. Dent R, Oliveira M, Isakoff SJ, Im SA, Espié M, Blau S, et al. Final results of the double-blind placebo-controlled randomized phase 2 LOTUS trial of first-line ipatasertib plus paclitaxel for inoperable locally advanced/metastatic triple-negative breast cancer. Breast Cancer Res Treat. 2021;189(2):377-86.

351. De Bono JS, De Giorgi U, Rodrigues DN, Massard C, Bracarda S, Font A, et al. Randomized Phase II Study Evaluating Akt Blockade with Ipatasertib, in Combination with Abiraterone, in Patients with Metastatic Prostate Cancer with and without PTEN Loss. Clin Cancer Res. 2019;25(3):928-36.

352. Turner N, Dent RA, O'Shaughnessy J, Kim SB, Isakoff SJ, Barrios C, et al. Ipatasertib plus paclitaxel for PIK3CA/AKT1/PTEN-altered hormone receptor-positive HER2-negative advanced breast cancer: primary results from cohort B of the IPATunity130 randomized phase 3 trial. Breast Cancer Res Treat. 2022;191(3):565-76.

353. Sweeney C, Bracarda S, Sternberg CN, Chi KN, Olmos D, Sandhu S, et al. Ipatasertib plus abiraterone and prednisolone in metastatic castration-resistant prostate cancer (IPATential150): a multicentre, randomised, double-blind, phase 3 trial. Lancet. 2021;398(10295):131-42.

354. Tsimberidou AM, Shaw JV, Juric D, Verschraegen C, Weise AM, Sarantopoulos J, et al. Phase 1 study of M2698, a p70S6K/AKT dual inhibitor, in patients with advanced cancer. J Hematol Oncol. 2021;14(1):127.

355. Machl A, Wilker EW, Tian H, Liu X, Schroeder P, Clark A, et al. M2698 is a potent dual-inhibitor of p70S6K and Akt that affects tumor growth in mouse models of cancer and crosses the blood-brain barrier. Am J Cancer Res. 2016;6(4):806-18.

356. Previs RA, Armaiz-Pena GN, Ivan C, Dalton HJ, Rupaimoole R, Hansen JM, et al. Role of YAP1 as a Marker of Sensitivity to Dual AKT and P70S6K Inhibition in Ovarian and Uterine Malignancies. J Natl Cancer Inst. 2017;109(7).

357. Tsimberidou AM, CVerschraegen CF, Weise AM, Sarantopoulos J, Lopes G, Nemunaitis JJ, et al. Precision oncology: Results of a phase I study of M2698, a p70S6K/AKT targeted agent in patients with advanced cancer and tumor PI3K/AKT/mTOR (PAM) pathway abnormalities. Journal of Clinical Oncology. 2018;36(15).

358. Yu Y, Hall T, Eathiraj S, Wick MJ, Schwartz B, Abbadessa G. In-vitro and in-vivo combined effect of ARQ 092, an AKT inhibitor, with ARQ 087, a FGFR inhibitor. Anticancer Drugs. 2017;28(5):503-13.

359. Jilkova ZM, Kuyucu AZ, Kurma K, Ahmad Pour ST, Roth GS, Abbadessa G, et al. Combination of AKT inhibitor ARQ 092 and sorafenib potentiates inhibition of tumor progression in cirrhotic rat model of hepatocellular carcinoma. Oncotarget. 2018;9(13):11145-58.

360. Hirai H, Sootome H, Nakatsuru Y, Miyama K, Taguchi S, Tsujioka K, et al. MK-2206, an allosteric Akt inhibitor, enhances antitumor efficacy by standard chemotherapeutic agents or molecular targeted drugs in vitro and in vivo. Mol Cancer Ther. 2010;9(7):1956-67.

361. Ma CX, Sanchez C, Gao F, Crowder R, Naughton M, Pluard T, et al. A Phase I Study of the AKT Inhibitor MK-2206 in Combination with Hormonal Therapy in Postmenopausal Women with Estrogen Receptor-Positive Metastatic Breast Cancer. Clin Cancer Res. 2016;22(11):2650-8.

362. Woo SU, Sangai T, Akcakanat A, Chen H, Wei C, Meric-Bernstam F. Vertical inhibition of the PI3K/Akt/mTOR pathway is synergistic in breast cancer. Oncogenesis. 2017;6(10):e385.

363. Wang Z, Luo G, Qiu Z. Akt inhibitor MK-2206 reduces pancreatic cancer cell viability and increases the efficacy of gemcitabine. Oncol Lett. 2020;19(3):1999-2004.

364. Zhang L, Wu Y, Wu J, Zhou M, Li D, Wan X, et al. KLF5-mediated COX2 upregulation contributes to tumorigenesis driven by PTEN deficiency. Cell Signal. 2020;75:109767.

365. Buschhaus JM, Humphries BA, Eckley SS, Robison TH, Cutter AC, Rajendran S, et al. Targeting disseminated estrogen-receptor-positive breast cancer cells in bone marrow. Oncogene. 2020;39(34):5649-62.

366. Yap TA, Yan L, Patnaik A, Fearen I, Olmos D, Papadopoulos K, et al. First-in-man clinical trial of the oral pan-AKT inhibitor MK-2206 in patients with advanced solid tumors. J Clin Oncol. 2011;29(35):4688-95.

367. Gupta S, Argilés G, Munster PN, Hollebecque A, Dajani O, Cheng JD, et al. A Phase I Trial of Combined Ridaforolimus and MK-2206 in Patients with Advanced Malignancies. Clin Cancer Res. 2015;21(23):5235-44.

368. Hudis C, Swanton C, Janjigian YY, Lee R, Sutherland S, Lehman R, et al. A phase 1 study evaluating the combination of an allosteric AKT inhibitor (MK-2206) and trastuzumab in patients with HER2-positive solid tumors. Breast Cancer Res. 2013;15(6):R110.

369. Brana I, Berger R, Golan T, Haluska P, Edenfield J, Fiorica J, et al. A parallel-arm phase I trial of the humanised anti-IGF-1R antibody dalotuzumab in combination with the AKT inhibitor MK-2206, the mTOR inhibitor ridaforolimus, or the NOTCH inhibitor MK-0752, in patients with advanced solid tumours. Br J Cancer. 2014;111(10):1932-44.

370. Chien AJ, Cockerill A, Fancourt C, Schmidt E, Moasser MM, Rugo HS, et al. A phase 1b study of the Akt-inhibitor MK-2206 in combination with weekly paclitaxel and trastuzumab in patients with advanced HER2-amplified solid tumor malignancies. Breast Cancer Res Treat. 2016;155(3):521-30.

371. Larsen JT, Shanafelt TD, Leis JF, LaPlant B, Call T, Pettinger A, et al. Akt inhibitor MK-2206 in combination with bendamustine and rituximab in relapsed or refractory chronic lymphocytic leukemia: Results from the N1087 alliance study. Am J Hematol. 2017;92(8):759-63.

372. Xing Y, Lin NU, Maurer MA, Chen H, Mahvash A, Sahin A, et al. Phase II trial of AKT inhibitor MK-2206 in patients with advanced breast cancer who have tumors with PIK3CA or AKT mutations, and/or PTEN loss/PTEN mutation. Breast Cancer Res. 2019;21(1):78.

373. Oki Y, Fanale M, Romaguera J, Fayad L, Fowler N, Copeland A, et al. Phase II study of an AKT inhibitor MK2206 in patients with relapsed or refractory lymphoma. Br J Haematol. 2015;171(4):463-70.

374. Ramanathan RK, McDonough SL, Kennecke HF, Iqbal S, Baranda JC, Seery TE, et al. Phase 2 study of MK-2206, an allosteric inhibitor of AKT, as second-line therapy for advanced gastric and gastroesophageal junction cancer: A SWOG cooperative group trial (S1005). Cancer. 2015;121(13):2193-7.

375. Stover EH, Xiong N, Myers AP, Tayob N, Engvold V, Polak M, et al. A phase II study of MK-2206, an AKT inhibitor, in uterine serous carcinoma. Gynecol Oncol Rep. 2022;40:100974.

376. Lara PN, Longmate J, Mack PC, Kelly K, Socinski MA, Salgia R, et al. Phase II Study of the AKT Inhibitor MK-2206 plus Erlotinib in Patients with Advanced Non-Small Cell Lung Cancer Who Previously Progressed on Erlotinib. Clin Cancer Res. 2015;21(19):4321-6.

377. Chien AJ, Tripathy D, Albain KS, Symmans WF, Rugo HS, Melisko ME, et al. MK-2206 and Standard Neoadjuvant Chemotherapy Improves Response in Patients With Human Epidermal Growth Factor Receptor 2-Positive and/or Hormone Receptor-Negative Breast Cancers in the I-SPY 2 Trial. J Clin Oncol. 2020;38(10):1059-69.

378. Pavlatovská B, Machálková M, Brisudová P, Pruška A, Štěpka K, Michálek J, et al. Lactic Acidosis Interferes With Toxicity of Perifosine to Colorectal Cancer Spheroids: Multimodal Imaging Analysis. Front Oncol. 2020;10:581365.

379. Karagul MI, Aktas S, Yilmaz SN, Yetkin D, Celikcan HD, Cevik OS. Perifosine and vitamin D combination induces apoptotic and non-apoptotic cell death in endometrial cancer cells. EXCLI J. 2020;19:532-46.

380. Song Z, Tu X, Zhou Q, Huang J, Chen Y, Liu J, et al. A novel UCHL3 inhibitor, perifosine, enhances PARP inhibitor cytotoxicity through inhibition of homologous recombination-mediated DNA double strand break repair. Cell Death Dis. 2019;10(6):398.

381. Holohan B, Hagiopian MM, Lai TP, Huang E, Friedman DR, Wright WE, et al. Perifosine as a potential novel anti-telomerase therapy. Oncotarget. 2015;6(26):21816-26.

382. Kushner BH, Cheung NV, Modak S, Becher OJ, Basu EM, Roberts SS, et al. A phase I/Ib trial targeting the Pi3k/Akt pathway using perifosine: Long-term progression-free survival of patients with resistant neuroblastoma. Int J Cancer. 2017;140(2):480-4.

383. Kaley TJ, Panageas KS, Pentsova EI, Mellinghoff IK, Nolan C, Gavrilovic I, et al. Phase I clinical trial of temsirolimus and perifosine for recurrent glioblastoma. Ann Clin Transl Neurol. 2020;7(4):429-36.

384. Jakubowiak AJ, Richardson PG, Zimmerman T, Alsina M, Kaufman JL, Kandarpa M, et al. Perifosine plus lenalidomide and dexamethasone in relapsed and relapsed/refractory multiple myeloma: a Phase I Multiple Myeloma Research Consortium study. Br J Haematol. 2012;158(4):472-80.

385. Richardson PG, Wolf J, Jakubowiak A, Zonder J, Lonial S, Irwin D, et al. Perifosine plus bortezomib and dexamethasone in patients with relapsed/refractory multiple myeloma previously treated with bortezomib: results of a multicenter phase I/II trial. J Clin Oncol. 2011;29(32):4243-9.

386. Bendell JC, Nemunaitis J, Vukelja SJ, Hagenstad C, Campos LT, Hermann RC, et al. Randomized placebo-controlled phase II trial of perifosine plus capecitabine as second- or third-line therapy in patients with metastatic colorectal cancer. J Clin Oncol. 2011;29(33):4394-400.

387. Rizk M, Rizq O, Oshima M, Nakajima-Takagi Y, Koide S, Saraya A, et al. Akt inhibition synergizes with polycomb repressive complex 2 inhibition in the treatment of multiple myeloma. Cancer Sci. 2019;110(12):3695-707.

388. Mimura N, Hideshima T, Shimomura T, Suzuki R, Ohguchi H, Rizq O, et al. Selective and potent Akt inhibition triggers anti-myeloma activities and enhances fatal endoplasmic reticulum stress induced by proteasome inhibition. Cancer Res. 2014;74(16):4458-69.

389. Lee JB, Jung M, Beom SH, Kim GM, Kim HR, Choi HJ, et al. Phase 2 study of TAS-117, an allosteric akt inhibitor in advanced solid tumors harboring phosphatidylinositol 3-kinase/v-akt murine thymoma viral oncogene homolog gene mutations. Invest New Drugs. 2021;39(5):1366-74.

390. Barnes EME, Xu Y, Benito A, Herendi L, Siskos AP, Aboagye EO, et al. Lactic acidosis induces resistance to the pan-Akt inhibitor uprosertib in colon cancer cells. Br J Cancer. 2020;122(9):1298-308.

391. Burris HA, Siu LL, Infante JR, Wheler JJ, Kurkjian C, Opalinska J, et al. Safety, pharmacokinetics (PK), pharmacodynamics (PD), and clinical activity of the oral AKT inhibitor GSK2141795 (GSK795) in a phase I first-in-human study. (Abstract). J Clin Oncol. 2011;29(15).

392. Hudes G, Carducci M, Tomczak P, Dutcher J, Figlin R, Kapoor A, et al. Temsirolimus, interferon alfa, or both for advanced renal-cell carcinoma. N Engl J Med. 2007;356(22):2271-81.

393. Sarbassov DD, Guertin DA, Ali SM, Sabatini DM. Phosphorylation and regulation of Akt/PKB by the rictor-mTOR complex. Science. 2005;307(5712):1098-101.

394. Wang Z, Feng X, Molinolo AA, Martin D, Vitale-Cross L, Nohata N, et al. 4E-BP1 Is a Tumor Suppressor Protein Reactivated by mTOR Inhibition in Head and Neck Cancer. Cancer Res. 2019;79(7):1438-50.

395. Hsu PP, Kang SA, Rameseder J, Zhang Y, Ottina KA, Lim D, et al. The mTOR-regulated phosphoproteome reveals a mechanism of mTORC1-mediated inhibition of growth factor signaling. Science. 2011;332(6035):1317-22.

396. Ariaans G, Jalving M, Vries EG, Jong S. Anti-tumor effects of everolimus and metformin are complementary and glucose-dependent in breast cancer cells. BMC Cancer. 2017;17(1):232.

397. Carvalho DM, Richardson PJ, Olaciregui N, Stankunaite R, Lavarino C, Molinari V, et al. Repurposing Vandetanib plus Everolimus for the Treatment of ACVR1-Mutant Diffuse Intrinsic Pontine Glioma. Cancer Discov. 2022;12(2):416-31.

398. Janku F, Yap TA, Meric-Bernstam F. Targeting the PI3K pathway in cancer: are we making headway? Nat Rev Clin Oncol. 2018;15(5):273-91.

399. Motzer RJ, Hutson TE, Glen H, Michaelson MD, Molina A, Eisen T, et al. Lenvatinib, everolimus, and the combination in patients with metastatic renal cell carcinoma: a randomised, phase 2, open-label, multicentre trial. Lancet Oncol. 2015;16(15):1473-82.

400. Ballhausen A, Wheler JJ, Karp DD, Piha-Paul SA, Fu S, Pant S, et al. Phase I Study of Everolimus, Letrozole, and Trastuzumab in Patients with Hormone Receptor-positive Metastatic Breast Cancer or Other Solid Tumors. Clin Cancer Res. 2021;27(5):1247-55.

401. Bautista F, Paoletti X, Rubino J, Brard C, Rezai K, Nebchi S, et al. Phase I or II Study of Ribociclib in Combination With Topotecan-Temozolomide or Everolimus in Children With Advanced Malignancies: Arms A and B of the AcSé-ESMART Trial. J Clin Oncol. 2021;39(32):3546-60.

402. Bardia A, Hurvitz SA, DeMichele A, Clark AS, Zelnak A, Yardley DA, et al. Phase I/II Trial of Exemestane, Ribociclib, and Everolimus in Women with HR. Clin Cancer Res. 2021;27(15):4177-85.

403. Schmid P, Sablin MP, Bergh J, Im SA, Lu YS, Martínez N, et al. A phase Ib/II study of xentuzumab, an IGF-neutralising antibody, combined with exemestane and everolimus in hormone receptor-positive, HER2-negative locally advanced/metastatic breast cancer. Breast Cancer Res. 2021;23(1):8.

404. Lau DK, Tay RY, Yeung YH, Chionh F, Mooi J, Murone C, et al. Phase II study of everolimus (RAD001) monotherapy as first-line treatment in advanced biliary tract cancer with biomarker exploration: the RADiChol Study. Br J Cancer. 2018;118(7):966-71.

405. Escudier B, Molinie V, Bracarda S, Maroto P, Szczylik C, Nathan P, et al. Open-label phase 2 trial of first-line everolimus monotherapy in patients with papillary metastatic renal cell carcinoma: RAPTOR final analysis. Eur J Cancer. 2016;69:226-35.

406. Jerusalem G, de Boer RH, Hurvitz S, Yardley DA, Kovalenko E, Ejlertsen B, et al. Everolimus Plus Exemestane vs Everolimus or Capecitabine Monotherapy for Estrogen Receptor-Positive, HER2-Negative Advanced Breast Cancer: The BOLERO-6 Randomized Clinical Trial. JAMA Oncol. 2018;4(10):1367-74.

407. Fan Y, Sun T, Shao Z, Zhang Q, Ouyang Q, Tong Z, et al. Effectiveness of Adding Everolimus to the First-line Treatment of Advanced Breast Cancer in Premenopausal Women Who Experienced Disease Progression While Receiving Selective Estrogen Receptor Modulators: A Phase 2 Randomized Clinical Trial. JAMA Oncol. 2021:e213428.

408. Slomovitz BM, Jiang Y, Yates MS, Soliman PT, Johnston T, Nowakowski M, et al. Phase II study of everolimus and letrozole in patients with recurrent endometrial carcinoma. J Clin Oncol. 2015;33(8):930-6.

409. Jun T, Hahn NM, Sonpavde G, Albany C, MacVicar GR, Hauke R, et al. Phase II Clinical and Translational Study of Everolimus ± Paclitaxel as First-Line Therapy in Cisplatin-Ineligible Advanced Urothelial Carcinoma. Oncologist. 2022;27(6):432-e52.

410. Soliman PT, Westin SN, Iglesias DA, Fellman BM, Yuan Y, Zhang Q, et al. Everolimus, Letrozole, and Metformin in Women with Advanced or Recurrent Endometrioid Endometrial Cancer: A Multi-Center, Single Arm, Phase II Study. Clin Cancer Res. 2020;26(3):581-7.

411. Yao JC, Shah MH, Ito T, Bohas CL, Wolin EM, Van Cutsem E, et al. Everolimus for advanced pancreatic neuroendocrine tumors. N Engl J Med. 2011;364(6):514-23.

412. Yao JC, Fazio N, Singh S, Buzzoni R, Carnaghi C, Wolin E, et al. Everolimus for the treatment of advanced, non-functional neuroendocrine tumours of the lung or gastrointestinal tract (RADIANT-4): a randomised, placebo-controlled, phase 3 study. Lancet. 2016;387(10022):968-77.

413. Fazio N, Buzzoni R, Delle Fave G, Tesselaar ME, Wolin E, Van Cutsem E, et al. Everolimus in advanced, progressive, well-differentiated, non-functional neuroendocrine tumors: RADIANT-4 lung subgroup analysis. Cancer Sci. 2018;109(1):174-81.

414. Motzer RJ, Escudier B, Oudard S, Hutson TE, Porta C, Bracarda S, et al. Efficacy of everolimus in advanced renal cell carcinoma: a double-blind, randomised, placebo-controlled phase III trial. Lancet. 2008;372(9637):449-56.

415. Bissler JJ, Kingswood JC, Radzikowska E, Zonnenberg BA, Frost M, Belousova E, et al. Everolimus for angiomyolipoma associated with tuberous sclerosis complex or sporadic lymphangioleiomyomatosis (EXIST-2): a multicentre, randomised, double-blind, placebo-controlled trial. Lancet. 2013;381(9869):817-24.

416. Franz DN, Belousova E, Sparagana S, Bebin EM, Frost M, Kuperman R, et al. Efficacy and safety of everolimus for subependymal giant cell astrocytomas associated with tuberous sclerosis complex (EXIST-1): a multicentre, randomised, placebo-controlled phase 3 trial. Lancet. 2013;381(9861):125-32.

417. Baselga J, Campone M, Piccart M, Burris HA, Rugo HS, Sahmoud T, et al. Everolimus in postmenopausal hormone-receptor-positive advanced breast cancer. N Engl J Med. 2012;366(6):520-9.

418. Tesch H, Stoetzer O, Decker T, Kurbacher CM, Marmé F, Schneeweiss A, et al. Efficacy and safety of everolimus plus exemestane in postmenopausal women with hormone receptor-positive, human epidermal growth factor receptor 2-negative locally advanced or metastatic breast cancer: Results of the single-arm, phase IIIB 4EVER trial. Int J Cancer. 2019;144(4):877-85.

419. Toi M, Shao Z, Hurvitz S, Tseng LM, Zhang Q, Shen K, et al. Efficacy and safety of everolimus in combination with trastuzumab and paclitaxel in Asian patients with HER2+ advanced breast cancer in BOLERO-1. Breast Cancer Res. 2017;19(1):47.

420. Kennecke H, Rahman M, Yip S, Woods R, Schaeffer D, Tai I. Effect of nab-rapamycin versus rapamycin in colorectal cancer cell lines and associations with KRAS and PI3K mutations (Abstract). J Clin Oncol. 2011;29(15).

421. Cirstea D, Hideshima T, Rodig S, Santo L, Pozzi S, Vallet S, et al. Dual inhibition of akt/mammalian target of rapamycin pathway by nanoparticle albumin-bound-rapamycin and perifosine induces antitumor activity in multiple myeloma. Mol Cancer Ther. 2010;9(4):963-75.

422. Desai N, D’Cruz O, Trieu V. Combination regimens of nab-rapamycin (ABI-009) effective against MDA-MB-231 breast-tumor xenografts. (Abstract). Cancer Res. 2009;69(24).

423. Gonzalez-Angulo AM, Meric-Bernstam F, Chawla S, Falchook G, Hong D, Akcakanat A, et al. Weekly nab-Rapamycin in patients with advanced nonhematologic malignancies: final results of a phase I trial. Clin Cancer Res. 2013;19(19):5474-84.

424. Wagner AJ, Ravi V, Riedel RF, Ganjoo K, Van Tine BA, Chugh R, et al. nab-Sirolimus for Patients With Malignant Perivascular Epithelioid Cell Tumors. J Clin Oncol. 2021;39(33):3660-70.

425. Li J, Kim SG, Blenis J. Rapamycin: one drug, many effects. Cell Metab. 2014;19(3):373-9.

426. Hosoi H, Dilling MB, Shikata T, Liu LN, Shu L, Ashmun RA, et al. Rapamycin causes poorly reversible inhibition of mTOR and induces p53-independent apoptosis in human rhabdomyosarcoma cells. Cancer Res. 1999;59(4):886-94.

427. Oshiro N, Yoshino K, Hidayat S, Tokunaga C, Hara K, Eguchi S, et al. Dissociation of raptor from mTOR is a mechanism of rapamycin-induced inhibition of mTOR function. Genes Cells. 2004;9(4):359-66.

428. Ishibashi Y, Nakamura O, Yamagami Y, Nishimura H, Fukuoka N, Yamamoto T. Chloroquine Enhances Rapamycin-induced Apoptosis in MG63 Cells. Anticancer Res. 2019;39(2):649-54.

429. Ozates NP, Soğutlu F, Lerminoglu F, Demir B, Gunduz C, Shademan B, et al. Effects of rapamycin and AZD3463 combination on apoptosis, autophagy, and cell cycle for resistance control in breast cancer. Life Sci. 2021;264:118643.

430. Chen YQ, Zhu WT, Lin CY, Yuan ZW, Li ZH, Yan PK. Delivery of Rapamycin by Liposomes Synergistically Enhances the Chemotherapy Effect of 5-Fluorouracil on Colorectal Cancer. Int J Nanomedicine. 2021;16:269-81.

431. Sun CY, Li YZ, Cao D, Zhou YF, Zhang MY, Wang HY. Rapamycin and trametinib: a rational combination for treatment of NSCLC. Int J Biol Sci. 2021;17(12):3211-23.

432. Rizzieri DA, Feldman E, Dipersio JF, Gabrail N, Stock W, Strair R, et al. A phase 2 clinical trial of deforolimus (AP23573, MK-8669), a novel mammalian target of rapamycin inhibitor, in patients with relapsed or refractory hematologic malignancies. Clin Cancer Res. 2008;14(9):2756-62.

433. Hartford CM, Ratain MJ. Rapamycin: something old, something new, sometimes borrowed and now renewed. Clin Pharmacol Ther. 2007;82(4):381-8.

434. Cloughesy TF, Yoshimoto K, Nghiemphu P, Brown K, Dang J, Zhu S, et al. Antitumor activity of rapamycin in a Phase I trial for patients with recurrent PTEN-deficient glioblastoma. PLoS Med. 2008;5(1):e8.

435. Cohen EE, Wu K, Hartford C, Kocherginsky M, Eaton KN, Zha Y, et al. Phase I studies of sirolimus alone or in combination with pharmacokinetic modulators in advanced cancer patients. Clin Cancer Res. 2012;18(17):4785-93.

436. Agarwal N, Rinaldetti S, Cheikh BB, Zhou Q, Hass EP, Jones RT, et al. TRIM28 is a transcriptional activator of the mutant TERT promoter in human bladder cancer. Proc Natl Acad Sci U S A. 2021;118(38).

437. Becker MA, Hou X, Tienchaianada P, Haines BB, Harrington SC, Weroha SJ, et al. Ridaforolimus (MK-8669) synergizes with Dalotuzumab (MK-0646) in hormone-sensitive breast cancer. BMC Cancer. 2016;16(1):814.

438. Pearson AD, Federico SM, Aerts I, Hargrave DR, DuBois SG, Iannone R, et al. A phase 1 study of oral ridaforolimus in pediatric patients with advanced solid tumors. Oncotarget. 2016;7(51):84736-47.

439. Di Cosimo S, Sathyanarayanan S, Bendell JC, Cervantes A, Stein MN, Braña I, et al. Combination of the mTOR inhibitor ridaforolimus and the anti-IGF1R monoclonal antibody dalotuzumab: preclinical characterization and phase I clinical trial. Clin Cancer Res. 2015;21(1):49-59.

440. Piha-Paul SA, Munster PN, Hollebecque A, Argilés G, Dajani O, Cheng JD, et al. Results of a phase 1 trial combining ridaforolimus and MK-0752 in patients with advanced solid tumours. Eur J Cancer. 2015;51(14):1865-73.

441. Chon HS, Kang S, Lee JK, Apte SM, Shahzad MM, Williams-Elson I, et al. Phase I study of oral ridaforolimus in combination with paclitaxel and carboplatin in patients with solid tumor cancers. BMC Cancer. 2017;17(1):407.

442. Mita MM, Poplin E, Britten CD, Tap WD, Rubin EH, Scott BB, et al. Phase I/IIa trial of the mammalian target of rapamycin inhibitor ridaforolimus (AP23573; MK-8669) administered orally in patients with refractory or advanced malignancies and sarcoma. Ann Oncol. 2013;24(4):1104-11.

443. Colombo N, McMeekin DS, Schwartz PE, Sessa C, Gehrig PA, Holloway R, et al. Ridaforolimus as a single agent in advanced endometrial cancer: results of a single-arm, phase 2 trial. Br J Cancer. 2013;108(5):1021-6.

444. Seiler M, Ray-Coquard I, Melichar B, Yardley DA, Wang RX, Dodion PF, et al. Oral ridaforolimus plus trastuzumab for patients with HER2+ trastuzumab-refractory metastatic breast cancer. Clin Breast Cancer. 2015;15(1):60-5.

445. Demetri GD, Chawla SP, Ray-Coquard I, Le Cesne A, Staddon AP, Milhem MM, et al. Results of an international randomized phase III trial of the mammalian target of rapamycin inhibitor ridaforolimus versus placebo to control metastatic sarcomas in patients after benefit from prior chemotherapy. J Clin Oncol. 2013;31(19):2485-92.

446. Harding MW. Immunophilins, mTOR, and pharmacodynamic strategies for a targeted cancer therapy. Clin Cancer Res. 2003;9(8):2882-6.

447. Hay N, Sonenberg N. Upstream and downstream of mTOR. Genes Dev. 2004;18(16):1926-45.

448. Pantuck AJ, Zeng G, Belldegrun AS, Figlin RA. Pathobiology, prognosis, and targeted therapy for renal cell carcinoma: exploiting the hypoxia-induced pathway. Clin Cancer Res. 2003;9(13):4641-52.

449. Chen Z, Yang H, Li Z, Xia Q, Nie Y. Temsirolimus as a dual inhibitor of retinoblastoma and angiogenesis via targeting mTOR signalling. Biochem Biophys Res Commun. 2019;516(3):726-32.

450. Kim SY, Jeong EH, Lee TG, Kim HR, Kim CH. The Combination of Trametinib, a MEK Inhibitor, and Temsirolimus, an mTOR Inhibitor, Radiosensitizes Lung Cancer Cells. Anticancer Res. 2021;41(6):2885-94.

451. Kwitkowski VE, Prowell TM, Ibrahim A, Farrell AT, Justice R, Mitchell SS, et al. FDA approval summary: temsirolimus as treatment for advanced renal cell carcinoma. Oncologist. 2010;15(4):428-35.

452. Trivedi ND, Armstrong S, Wang H, Hartley M, Deeken J, Ruth He A, et al. A phase I trial of the mTOR inhibitor temsirolimus in combination with capecitabine in patients with advanced malignancies. Cancer Med. 2021;10(6):1944-54.

453. Tasian SK, Silverman LB, Whitlock JA, Sposto R, Loftus JP, Schafer ES, et al. Temsirolimus combined with cyclophosphamide and etoposide for pediatric patients with relapsed/refractory acute lymphoblastic leukemia: a Therapeutic Advances in Childhood Leukemia Consortium trial (TACL 2014-001). Haematologica. 2022.

454. Inwards DJ, Fishkin PA, LaPlant BR, Drake MT, Kurtin PJ, Nikcevich DA, et al. Phase I trial of rituximab, cladribine, and temsirolimus (RCT) for initial therapy of mantle cell lymphoma. Ann Oncol. 2014;25(10):2020-4.

455. Major A, Kline J, Karrison TG, Fishkin PAS, Kimball AS, Petrich AM, et al. Phase I/II clinical trial of temsirolimus and lenalidomide in patients with relapsed and refractory lymphomas. Haematologica. 2021.

456. Korfel A, Schlegel U, Herrlinger U, Dreyling M, Schmidt C, von Baumgarten L, et al. Phase II Trial of Temsirolimus for Relapsed/Refractory Primary CNS Lymphoma. J Clin Oncol. 2016;34(15):1757-63.

457. Hobday TJ, Qin R, Reidy-Lagunes D, Moore MJ, Strosberg J, Kaubisch A, et al. Multicenter Phase II Trial of Temsirolimus and Bevacizumab in Pancreatic Neuroendocrine Tumors. J Clin Oncol. 2015;33(14):1551-6.

458. Mascarenhas L, Chi YY, Hingorani P, Anderson JR, Lyden ER, Rodeberg DA, et al. Randomized Phase II Trial of Bevacizumab or Temsirolimus in Combination With Chemotherapy for First Relapse Rhabdomyosarcoma: A Report From the Children's Oncology Group. J Clin Oncol. 2019;37(31):2866-74.

459. Dreyling M, Jurczak W, Jerkeman M, Silva RS, Rusconi C, Trneny M, et al. Ibrutinib versus temsirolimus in patients with relapsed or refractory mantle-cell lymphoma: an international, randomised, open-label, phase 3 study. Lancet. 2016;387(10020):770-8.

460. Laplante M, Sabatini DM. mTOR signaling in growth control and disease. Cell. 2012;149(2):274-93.

461. Shi JJ, Chen SM, Guo CL, Li YX, Ding J, Meng LH. The mTOR inhibitor AZD8055 overcomes tamoxifen resistance in breast cancer cells by down-regulating HSPB8. Acta Pharmacol Sin. 2018;39(8):1338-46.

462. Chang L, Huang Z, Li S, Yao Z, Bao H, Wang Z, et al. A low dose of AZD8055 enhances radiosensitivity of nasopharyngeal carcinoma cells by activating autophagy and apoptosis. Am J Cancer Res. 2019;9(9):1922-37.

463. Chen Y, Lee CH, Tseng BY, Tsai YH, Tsai HW, Yao CL, et al. AZD8055 Exerts Antitumor Effects on Colon Cancer Cells by Inhibiting mTOR and Cell-cycle Progression. Anticancer Res. 2018;38(3):1445-54.

464. Kauffman EC, Lang M, Rais-Bahrami S, Gupta GN, Wei D, Yang Y, et al. Preclinical efficacy of dual mTORC1/2 inhibitor AZD8055 in renal cell carcinoma harboring a TFE3 gene fusion. BMC Cancer. 2019;19(1):917.

465. Wang H, Liu Y, Ding J, Huang Y, Liu J, Liu N, et al. Targeting mTOR suppressed colon cancer growth through 4EBP1/eIF4E/PUMA pathway. Cancer Gene Ther. 2020;27(6):448-60.

466. Zhao T, Siu IM, Williamson T, Zhang H, Ji C, Burger PC, et al. AZD8055 enhances in vivo efficacy of afatinib in chordomas. J Pathol. 2021;255(1):72-83.

467. Naing A, Aghajanian C, Raymond E, Olmos D, Schwartz G, Oelmann E, et al. Safety, tolerability, pharmacokinetics and pharmacodynamics of AZD8055 in advanced solid tumours and lymphoma. Br J Cancer. 2012;107(7):1093-9.

468. Asahina H, Nokihara H, Yamamoto N, Yamada Y, Tamura Y, Honda K, et al. Safety and tolerability of AZD8055 in Japanese patients with advanced solid tumors; a dose-finding phase I study. Invest New Drugs. 2013;31(3):677-84.

469. Xie Z, Wang J, Liu M, Chen D, Qiu C, Sun K. CC-223 blocks mTORC1/C2 activation and inhibits human hepatocellular carcinoma cells in vitro and in vivo. PLoS One. 2017;12(3):e0173252.

470. Wang JY, Jin X, Zhang X, Li XF. CC-223 inhibits human head and neck squamous cell carcinoma cell growth. Biochem Biophys Res Commun. 2018;496(4):1191-6.

471. Bendell JC, Kelley RK, Shih KC, Grabowsky JA, Bergsland E, Jones S, et al. A phase I dose-escalation study to assess safety, tolerability, pharmacokinetics, and preliminary efficacy of the dual mTORC1/mTORC2 kinase inhibitor CC-223 in patients with advanced solid tumors or multiple myeloma. Cancer. 2015;121(19):3481-90.

472. Wolin E, Mita A, Mahipal A, Meyer T, Bendell J, Nemunaitis J, et al. A phase 2 study of an oral mTORC1/mTORC2 kinase inhibitor (CC-223) for non-pancreatic neuroendocrine tumors with or without carcinoid symptoms. PLoS One. 2019;14(9):e0221994.

473. Srivastava RK, Li C, Khan J, Banerjee NS, Chow LT, Athar M. Combined mTORC1/mTORC2 inhibition blocks growth and induces catastrophic macropinocytosis in cancer cells. Proc Natl Acad Sci U S A. 2019;116(49):24583-92.

474. Lou J, Lv JX, Zhang YP, Liu ZJ. OSI-027 inhibits the tumorigenesis of colon cancer through mediation of c-Myc/FOXO3a/PUMA axis. Cell Biol Int. 2022.

475. Xu E, Zhu H, Wang F, Miao J, Du S, Zheng C, et al. OSI-027 alleviates oxaliplatin chemoresistance in gastric cancer cells by suppressing P-gp induction. Curr Mol Med. 2020.

476. Zhi X, Chen W, Xue F, Liang C, Chen BW, Zhou Y, et al. OSI-027 inhibits pancreatic ductal adenocarcinoma cell proliferation and enhances the therapeutic effect of gemcitabine both in vitro and in vivo. Oncotarget. 2015;6(28):26230-41.

477. Zhen MC, Wang FQ, Wu SF, Zhao YL, Liu PG, Yin ZY. Identification of mTOR as a primary resistance factor of the IAP antagonist AT406 in hepatocellular carcinoma cells. Oncotarget. 2017;8(6):9466-75.

478. Mateo J, Olmos D, Dumez H, Poondru S, Samberg NL, Barr S, et al. A first in man, dose-finding study of the mTORC1/mTORC2 inhibitor OSI-027 in patients with advanced solid malignancies. Br J Cancer. 2016;114(8):889-96.

479. Wong RA, Luo X, Lu M, An Z, Haas-Kogan DA, Phillips JJ, et al. Cooperative Blockade of PKCα and JAK2 Drives Apoptosis in Glioblastoma. Cancer Res. 2020;80(4):709-18.

480. Sanz-Álvarez M, Martín-Aparicio E, Luque M, Zazo S, Martínez-Useros J, Eroles P, et al. The Novel Oral mTORC1/2 Inhibitor TAK-228 Reverses Trastuzumab Resistance in HER2-Positive Breast Cancer Models. Cancers (Basel). 2021;13(11).

481. Wei BR, Hoover SB, Peer CJ, Dwyer JE, Adissu HA, Shankarappa P, et al. Efficacy, Tolerability, and Pharmacokinetics of Combined Targeted MEK and Dual mTORC1/2 Inhibition in a Preclinical Model of Mucosal Melanoma. Mol Cancer Ther. 2020;19(11):2308-18.

482. Iyer G, Hanrahan AJ, Milowsky MI, Al-Ahmadie H, Scott SN, Janakiraman M, et al. Genome sequencing identifies a basis for everolimus sensitivity. Science. 2012;338(6104):221.

483. Ghobrial IM, Siegel DS, Vij R, Berdeja JG, Richardson PG, Neuwirth R, et al. TAK-228 (formerly MLN0128), an investigational oral dual TORC1/2 inhibitor: A phase I dose escalation study in patients with relapsed or refractory multiple myeloma, non-Hodgkin lymphoma, or Waldenström's macroglobulinemia. Am J Hematol. 2016;91(4):400-5.

484. Shimizu T, Kuboki Y, Lin CC, Yonemori K, Yanai T, Faller DV, et al. A Phase 1 Study of Sapanisertib (TAK-228) in East Asian Patients with Advanced Nonhematological Malignancies. Target Oncol. 2022;17(1):15-24.

485. Voss MH, Gordon MS, Mita M, Rini B, Makker V, Macarulla T, et al. Phase 1 study of mTORC1/2 inhibitor sapanisertib (TAK-228) in advanced solid tumours, with an expansion phase in renal, endometrial or bladder cancer. Br J Cancer. 2020;123(11):1590-8.

486. Moore KN, Bauer TM, Falchook GS, Chowdhury S, Patel C, Neuwirth R, et al. Phase I study of the investigational oral mTORC1/2 inhibitor sapanisertib (TAK-228): tolerability and food effects of a milled formulation in patients with advanced solid tumours. ESMO Open. 2018;3(2):e000291.

487. Coleman N, Naing A, Zhang S, Piha-Paul SA, Tsimberidou AM, Janku F, et al. Phase I study of mTORC1/2 inhibitor sapanisertib (TAK-228) in combination with metformin in patients (pts) with mTOR/AKT/PI3K pathway alterations and advanced solid malignancies. Journal of Clinical Oncology. 2021;39(15).

488. Lim B, Potter DA, Salkeni MA, Silverman P, Haddad TC, Forget F, et al. Sapanisertib Plus Exemestane or Fulvestrant in Women with Hormone Receptor-Positive/HER2-Negative Advanced or Metastatic Breast Cancer. Clin Cancer Res. 2021;27(12):3329-38.

489. McGregor BA, Xie W, Adib E, Stadler WM, Zakharia Y, Alva A, et al. Biomarker-Based Phase II Study of Sapanisertib (TAK-228): An mTORC1/2 Inhibitor in Patients With Refractory Metastatic Renal Cell Carcinoma. JCO Precis Oncol. 2022;6:e2100448.

490. Koca E, Niravath PA, Ensor J, Patel TA, Li X, Hemati P, et al. ANETT: PhAse II trial of NEoadjuvant TAK-228 plus Tamoxifen in patients with hormone receptor-positive breast cancer. Breast Cancer Res Treat. 2021;188(2):433-9.

491. García-Sáenz J, Martínez-Jáñez N, Cubedo R, Jerez Y, Lahuerta A, González-Santiago S, et al. Sapanisertib plus Fulvestrant in Postmenopausal Women with Estrogen Receptor-Positive/HER2-Negative Advanced Breast Cancer after Progression on Aromatase Inhibitor. Clin Cancer Res. 2022;28(6):1107-16.

492. Feldman ME, Apsel B, Uotila A, Loewith R, Knight ZA, Ruggero D, et al. Active-site inhibitors of mTOR target rapamycin-resistant outputs of mTORC1 and mTORC2. PLoS Biol. 2009;7(2):e38.

493. Apsel B, Blair JA, Gonzalez B, Nazif TM, Feldman ME, Aizenstein B, et al. Targeted polypharmacology: discovery of dual inhibitors of tyrosine and phosphoinositide kinases. Nat Chem Biol. 2008;4(11):691-9.

494. Hoang B, Frost P, Shi Y, Belanger E, Benavides A, Pezeshkpour G, et al. Targeting TORC2 in multiple myeloma with a new mTOR kinase inhibitor. Blood. 2010;116(22):4560-8.

495. Xing X, Zhang L, Wen X, Wang X, Cheng X, Du H, et al. PP242 suppresses cell proliferation, metastasis, and angiogenesis of gastric cancer through inhibition of the PI3K/AKT/mTOR pathway. Anticancer Drugs. 2014;25(10):1129-40.

496. Mecca C, Giambanco I, Bruscoli S, Bereshchenko O, Fioretti B, Riccardi C, et al. PP242 Counteracts Glioblastoma Cell Proliferation, Migration, Invasiveness and Stemness Properties by Inhibiting mTORC2/AKT. Front Cell Neurosci. 2018;12:99.

497. Zhang Z, Zhang G, Kong C, Gong D. PP242 suppresses bladder cancer cell proliferation and migration through deactivating the mammalian target of rapamycin complex 2/AKT1 signaling pathway. Mol Med Rep. 2016;13(1):333-8.

498. Qin Y, Zhao X, Fang Y. PP242 synergizes with suberoylanilide hydroxamic acid to inhibit growth of ovarian cancer cells. Int J Gynecol Cancer. 2014;24(8):1373-80.

499. Yang C, Huang X, Liu H, Xiao F, Wei J, You L, et al. PDK1 inhibitor GSK2334470 exerts antitumor activity in multiple myeloma and forms a novel multitargeted combination with dual mTORC1/C2 inhibitor PP242. Oncotarget. 2017;8(24):39185-97.

500. Rashid MM, Lee H, Jung BH. Evaluation of the antitumor effects of PP242 in a colon cancer xenograft mouse model using comprehensive metabolomics and lipidomics. Sci Rep. 2020;10(1):17523.

501. Kong L, Zhang Q, Mao J, Cheng L, Shi X, Yu L, et al. A dual-targeted molecular therapy of PP242 and cetuximab plays an anti-tumor effect through EGFR downstream signaling pathways in colorectal cancer. J Gastrointest Oncol. 2021;12(4):1625-42.

502. Cheng H, Zou Y, Ross JS, Wang K, Liu X, Halmos B, et al. RICTOR Amplification Defines a Novel Subset of Patients with Lung Cancer Who May Benefit from Treatment with mTORC1/2 Inhibitors. Cancer Discov. 2015;5(12):1262-70.

503. Yu CC, Huang HB, Hung SK, Liao HF, Lee CC, Lin HY, et al. AZD2014 Radiosensitizes Oral Squamous Cell Carcinoma by Inhibiting AKT/mTOR Axis and Inducing G1/G2/M Cell Cycle Arrest. PLoS One. 2016;11(3):e0151942.

504. Kahn J, Hayman TJ, Jamal M, Rath BH, Kramp T, Camphausen K, et al. The mTORC1/mTORC2 inhibitor AZD2014 enhances the radiosensitivity of glioblastoma stem-like cells. Neuro Oncol. 2014;16(1):29-37.

505. Li S, Sheng J, Liu Z, Fan Y, Zhang C, Lv T, et al. Potent antitumour of the mTORC1/2 dual inhibitor AZD2014 in docetaxel-sensitive and docetaxel-resistant castration-resistant prostate cancer cells. J Cell Mol Med. 2021;25(5):2436-49.

506. Pi R, Yang Y, Hu X, Li H, Shi H, Liu Y, et al. Dual mTORC1/2 inhibitor AZD2014 diminishes myeloid-derived suppressor cells accumulation in ovarian cancer and delays tumor growth. Cancer Lett. 2021.

507. Wong Te Fong AC, Thavasu P, Gagrica S, Swales KE, Leach MO, Cosulich SC, et al. Evaluation of the combination of the dual m-TORC1/2 inhibitor vistusertib (AZD2014) and paclitaxel in ovarian cancer models. Oncotarget. 2017;8(69):113874-84.

508. Lapointe S, Mason W, MacNeil M, Harlos C, Tsang R, Sederias J, et al. A phase I study of vistusertib (dual mTORC1/2 inhibitor) in patients with previously treated glioblastoma multiforme: a CCTG study. Invest New Drugs. 2020;38(4):1137-44.

509. Basu B, Krebs MG, Sundar R, Wilson RH, Spicer J, Jones R, et al. Vistusertib (dual m-TORC1/2 inhibitor) in combination with paclitaxel in patients with high-grade serous ovarian and squamous non-small-cell lung cancer. Ann Oncol. 2018;29(9):1918-25.

510. Heudel P, Frenel JS, Dalban C, Bazan F, Joly F, Arnaud A, et al. Safety and Efficacy of the mTOR Inhibitor, Vistusertib, Combined With Anastrozole in Patients With Hormone Receptor-Positive Recurrent or Metastatic Endometrial Cancer: The VICTORIA Multicenter, Open-label, Phase 1/2 Randomized Clinical Trial. JAMA Oncol. 2022.

511. Eyre TA, Hildyard C, Hamblin A, Ali AS, Houlton A, Hopkins L, et al. A phase II study to assess the safety and efficacy of the dual mTORC1/2 inhibitor vistusertib in relapsed, refractory DLBCL. Hematol Oncol. 2019;37(4):352-9.

512. Schmid P, Zaiss M, Harper-Wynne C, Ferreira M, Dubey S, Chan S, et al. Fulvestrant Plus Vistusertib vs Fulvestrant Plus Everolimus vs Fulvestrant Alone for Women With Hormone Receptor-Positive Metastatic Breast Cancer: The MANTA Phase 2 Randomized Clinical Trial. JAMA Oncol. 2019;5(11):1556-64.

513. Lee BJ, Boyer JA, Burnett GL, Thottumkara AP, Tibrewal N, Wilson SL, et al. Selective inhibitors of mTORC1 activate 4EBP1 and suppress tumor growth. Nat Chem Biol. 2021;17(10):1065-74.

514. Rodrik-Outmezguine VS, Okaniwa M, Yao Z, Novotny CJ, McWhirter C, Banaji A, et al. Overcoming mTOR resistance mutations with a new-generation mTOR inhibitor. Nature. 2016;534(7606):272-6.

515. Lee BJ, Mallya S, Dinglasan N, Fung A, Nguyen T, Herzog LO, et al. Efficacy of a Novel Bi-Steric mTORC1 Inhibitor in Models of B-Cell Acute Lymphoblastic Leukemia. Front Oncol. 2021;11:673213.

516. Burnett GL, Yang YC, Aggen JB, Pitzen J, Gliedt MK, Semko CM, et al. Discovery of RMC-5552, a Selective Bi-Steric Inhibitor of mTORC1, for the Treatment of mTORC1-Activated Tumors. J Med Chem. 2023;66(1):149-69.

517. Burris HA, Ulahannan SV, Haura EB, Ignatius Ou SI, Capasso A, Munster PN, et al. The bi-steric mTORC1-selective inhibitor RMC-5552 in tumors with activation of mTOR signaling: Preclinical activity in combination with RAS(ON) inhibitors in RAS-addicted tumors, and initial clinical findings from a single agent phase 1/1b study. (Abstract). Journal of Clinical Oncology. 2022;40(16).

518. Emmanouilidi A, Falasca M. Targeting PDK1 for Chemosensitization of Cancer Cells. Cancers (Basel). 2017;9(10).

519. Nalairndran G, Hassan Abdul Razack A, Mai CW, Fei-Lei Chung F, Chan KK, Hii LW, et al. Phosphoinositide-dependent Kinase-1 (PDPK1) regulates serum/glucocorticoid-regulated Kinase 3 (SGK3) for prostate cancer cell survival. J Cell Mol Med. 2020;24(20):12188-98.

520. Maegawa S, Chinen Y, Shimura Y, Tanba K, Takimoto T, Mizuno Y, et al. Phosphoinositide-dependent protein kinase 1 is a potential novel therapeutic target in mantle cell lymphoma. Exp Hematol. 2018;59:72-81.e2.

521. Zhang J, Yang C, Zhou F, Chen X. PDK1 inhibitor GSK2334470 synergizes with proteasome inhibitor MG‑132 in multiple myeloma cells by inhibiting full AKT activity and increasing nuclear accumulation of the PTEN protein. Oncol Rep. 2018;39(6):2951-9.

522. Jansen VM, Bhola NE, Bauer JA, Formisano L, Lee KM, Hutchinson KE, et al. Kinome-Wide RNA Interference Screen Reveals a Role for PDK1 in Acquired Resistance to CDK4/6 Inhibition in ER-Positive Breast Cancer. Cancer Res. 2017;77(9):2488-99.

523. Nagashima K, Shumway SD, Sathyanarayanan S, Chen AH, Dolinski B, Xu Y, et al. Genetic and pharmacological inhibition of PDK1 in cancer cells: characterization of a selective allosteric kinase inhibitor. J Biol Chem. 2011;286(8):6433-48.

524. Daniele S, Sestito S, Pietrobono D, Giacomelli C, Chiellini G, Di Maio D, et al. Dual Inhibition of PDK1 and Aurora Kinase A: An Effective Strategy to Induce Differentiation and Apoptosis of Human Glioblastoma Multiforme Stem Cells. ACS Chem Neurosci. 2017;8(1):100-14.

525. Emmanouilidi A, Fyffe CA, Ferro R, Edling CE, Capone E, Sestito S, et al. Preclinical validation of 3-phosphoinositide-dependent protein kinase 1 inhibition in pancreatic cancer. J Exp Clin Cancer Res. 2019;38(1):191.

526. Falasca M, Chiozzotto D, Godage HY, Mazzoletti M, Riley AM, Previdi S, et al. A novel inhibitor of the PI3K/Akt pathway based on the structure of inositol 1,3,4,5,6-pentakisphosphate. Br J Cancer. 2010;102(1):104-14.

527. Komander D, Fairservice A, Deak M, Kular GS, Prescott AR, Peter Downes C, et al. Structural insights into the regulation of PDK1 by phosphoinositides and inositol phosphates. EMBO J. 2004;23(20):3918-28.

528. Raimondi C, Calleja V, Ferro R, Fantin A, Riley AM, Potter BV, et al. A Small Molecule Inhibitor of PDK1/PLCγ1 Interaction Blocks Breast and Melanoma Cancer Cell Invasion. Sci Rep. 2016;6:26142.

529. Nesi G, Sestito S, Mey V, Ricciardi S, Falasca M, Danesi R, et al. Synthesis of Novel 3,5-Disubstituted-2-oxindole Derivatives As Antitumor Agents against Human Nonsmall Cell Lung Cancer. ACS Med Chem Lett. 2013;4(12):1137-41.

530. Sestito S, Nesi G, Daniele S, Martelli A, Digiacomo M, Borghini A, et al. Design and synthesis of 2-oxindole based multi-targeted inhibitors of PDK1/Akt signaling pathway for the treatment of glioblastoma multiforme. Eur J Med Chem. 2015;105:274-88.

531. Casari I, Domenichini A, Sestito S, Capone E, Sala G, Rapposelli S, et al. Dual PDK1/Aurora Kinase A Inhibitors Reduce Pancreatic Cancer Cell Proliferation and Colony Formation. Cancers (Basel). 2019;11(11).

**SUPPLEMENTARY FIGURE 1 LEGEND**

**SUPPLEMENTARY FIGURE 1.** Timeline of FDA approvals for mTOR inhibitors (red rectangles), and PI3K inhibitors (green rectangles). A-NC mTORi: allosteric (non-competitive) mTOR inhibitor; Pan-PI3Ki: Pan-PI3K inhibitor; IS PI3Kαi: Isoform-Specific PI3Kα inhibitor; IS PI3Kγ/δi: Isoform-Specific PI3Kγ/δ inhibitor. Black arrows below the inhibitors: years in which the first FDA approval occurred. Grey squares at the top-left of each inhibitor: cancer type/s related to the first FDA approval of the corresponding inhibitor. RCC: Renal cell carcinoma; FL: Follicular lymphoma; CLL: Chronic lymphocytic leukemia; SLL: Acute lymphocytic leukemia; BR: Breast cancer. Circled capital letters at the top-right of each inhibitor: biopharmaceutical companies related to the first FDA approval of inhibitors. Ⓟ: Pfizer; Ⓝ: Novartis; Ⓖ: Gilead Sciences; Ⓑ = Bayer; Ⓢ: Secura Bio.

**SUPPLEMENTARY TABLE LEGENDS**

**SUPPLEMENTARY TABLE 1.** Summary of the most representative studies on pan-PI3K inhibitors, isoform-specific PI3K inhibitors, and dual-PI3K/mTOR inhibitors with favourable/acceptable safety profile in different stages of human clinical trials. ISP: inhibitor safety profile (F: favourable, A: acceptable or manageable or tolerable). C: complete; A, R: active recruiting; A, NR: active not recruiting; T: terminated; U: unknown. FP: first posted; RFP: results first posted; LUP: last update posted. NCT: national clinical trial. *: none; R: reference. *ORR*: overall response rate; *PR*: partial response; *CR*: complete response; *MR*: minimal response; *PFS*: Progression-free survival; *SD*: stable disease; *OS*: overall survival; *DOR*: duration of response; *CBR*: clinical benefit rate; *DCR*: disease control rate; *MTD*: maximum tolerated dose; *RP2D*: recommended phase 2 dose.

**SUPPLEMENTARY TABLE 2.** Summary of the most representative studies on AKT inhibitors with favourable/acceptable safety profile in different stages of human clinical trials. ISP: inhibitor safety profile (F: favourable, A: acceptable or manageable or tolerable). C: complete; A, R: active recruiting; A, NR: active not recruiting; T: terminated; U: unknown. FP: first posted; RFP: results first posted; LUP: last update posted. NCT: national clinical trial. *: none; R: reference. *ORR*: overall response rate; *PR*: partial response; *CR*: complete response; *MR*: minimal response; *PFS*: Progression-free survival; *SD*: stable disease; *OS*: overall survival; *DOR*: duration of response; *CBR*: clinical benefit rate; *DCR*: disease control rate; *MTD*: maximum tolerated dose; *RP2D*: recommended phase 2 dose.

**SUPPLEMENTARY TABLE 3.** Summary of the most representative studies on allosteric mTOR inhibitors, ATP-competitive mTOR inhibitors, and bi-steric mTOR inhibitors with favourable/acceptable safety profile in different stages of human clinical trials. ISP: inhibitor safety profile (F: favourable, A: acceptable or manageable or tolerable). C: complete; A, R: active recruiting; A, NR: active not recruiting; T: terminated; U: unknown. FP: first posted; RFP: results first posted; LUP: last update posted. NCT: national clinical trial. *: none; R: reference. *ORR*: overall response rate; *PR*: partial response; *CR*: complete response; *MR*: minimal response; *PFS*: Progression-free survival; *SD*: stable disease; *OS*: overall survival; *DOR*: duration of response; *CBR*: clinical benefit rate; *DCR*: disease control rate; *MTD*: maximum tolerated dose; *RP2D*: recommended phase 2 dose.
