# Supplementary material for: PI3K/AKT/mTOR signaling transduction pathway and targeted therapies in cancer
Source: Mol Cancer. 2023 Aug 18;22:138. doi: 10.1186/s12943-023-01827-6 (PMC10436543; doi:10.1186/s12943-023-01827-6)
Supplement: Supplementary file 4 — Additional file 4: Supplementary information 3. [file 12943_2023_1827_MOESM4_ESM.docx]

**SUPPLEMENTARY INFORMATION 3**

**RESISTANCE**

The PI3K/AKT/mTORC (PAM) signaling pathway involves numerous feedback loops, compensatory pathways, and crosstalk nodes with other signal transduction axes that hamper the inhibition of PI3K, AKT, mTORC1, mTORC2, and PDK1 in cancer. Indeed, recent studies have reported that short administration of drug inhibitor therapies can determine feedback loop inductions that consecutively reduce the overall response rate (ORR). Additionally, chronic administration of inhibitor-based therapies can lead to the accumulation of slow-cycling cells that can possibly gain genetic mutation contributing to drug resistance (1). The major mechanisms of resistance to PAM signalling-targeted inhibitors are revised below.

**MECHANISMS OF RESISTANCE TO PI3K INHIBITORS**

Even though PAM-targeted agents, particularly PI3K drug inhibitors, have demonstrated significant therapeutic activity in human cancer, acquired and intrinsic resistance has hindered their clinical efficacy. Thus, a rationale for alternative clinical strategies could be provided by an accurate understanding of the biochemical mechanisms whereby resistance to PI3Ki occur. Various targeted therapies can induce several possible mechanisms of resistance to PI3Ki as described below.

***Insulin signalling and PI3K reactivation***

Altered glucose metabolism is a typical characteristic of tumor cells, which feature an enhanced uptake of glucose, and a glycolytic transformation into lactate. The latter is an aerobic glycolysis, also known as the Warburg effect (2), which produces several intermediate metabolites necessary for important cellular functions, including protein production, and provides ATP-generated energy to tumor cells (3). Growth factor-induced insulin-like growth factor 1 receptor (IGF1R) and insulin receptor (InsR) lead to the successive phosphorylation of insulin receptor substrate (IRS) adaptor molecules, usually IRS1, which subsequently determines activation of PI3K. (Phosphorylated) insulin receptor substrate 1 (IRS1)-induced activation of PI3K occurs through binding to the two SH2 domains on the PI3K p85 subunit, alleviating its connection to the catalytic PI3K p110 subunit, thereby triggering the induction of PAM signaling axis (4). Coversely, when in surplus to the PI3K p110 subunit, PI3K p85 subunit also forms a sequestration complex with IRS1, which determines the transfer of phosphorylated IRS1 into the cytosol, distant from the site where PIP3 is produced on the cell membrane, thereby preventing IRS1-induced activation of PAM pathway (5). This mechanism can result in the inhibition of insulin signaling through the preservation of PI3K p85 subunit basal inhibition on PI3K p110 subunit, and concurrently, could further lead to insulin resistance if expressed in excess. Upon induction, the PI3K p110α subunit is then capable of mediating intracellular response-insulin stimulation, triggering growth and glucose homeostasis in the majority of tissues (6). In line with this, alterations to the *PIK3CA* gene-encoded PI3K p110 subunit are often linked to several types of cancer (7).

***Altered cell metabolism***

Recently a mechanism of resistance to dual PI3K/mTORi that depends on alterations of cell metabolism has been reported. A metabolic switch associated with mitochondrial DNA mutation has been shown as a mechanism of resistance to dactolisib in lung tumor cells. Indeed, these cancer cells present featues of increased glycolysis, including elevated level of glucose, glutamine, lactate, GLUT1 expression, glucose dependence, reduced levels of reactive oxygen species (ROS), and rates of oxygen consumption (8). Moreover, enhanced Notch activity associated with increased mitochondrial metabolism and fibroblast growth factor receptor 1 (FGFR1) signalling have been proposed as a mechanism of resistance to PI3K/mTOR inhibitors, occurring through the activation of a drug resistant cancer stem cell (CSC)-like population (9). Furthermore, it has been reported that patient-derived leukemia cells resistant to dual PI3K/mTORi exhibit substantial difference with regard to their metabolomic profile compared to sensitive cells. In fact, major alterations are detected in molecules mainly involved in energy, amino acid, and arachidonic acid metabolism (10).

***Interactions between PAM pathway and other pathways***

The PAM axis is highly interconnected with the Ras/MEK/ERK signaling pathway (11). Indeed, overexpression and mutation of Ras family protein *HRAS* decreases vulnerability to PI3K inhibitors, whereas its knockdown improves susceptibility to PI3K inhibitors (12). In addition, cooperations between MAP2K4 and NEK9 can play a mediating role in tumor cell proliferation as well as resistance to PI3K inhibitors (13). Interestingly, amplifications of both MYC and eIF4E have been found to mediate resistance to dual PI3K/mTORi (14). Indeed, MYC is known to affect proliferation, growth, differentiation, and tumor cell metabolism. Also, knockdown of MYC has been shown to reverse the resistance to dual PI3K/mTORi. Since eIF4E is a MYC-regulated target, their interactions may be implicated in the regulation of mechanisms that enhance dual PI3K/mTORi resistance (15). Suppression of PI3K with pan-PI3Ki pilaralisib or pictilisib, or dual PI3K/mTORi dactolisib, has been reported to enhance the expression of HER2/HER3, resulting in compensatory activation of the ERK signaling pathway (16) (17). Moreover, *PIK3CA* mutant cells are able to secrete factors activating Ras/MEK/ERK signaling, thereby endorsing cancer growth (18). Notably, MEK inhibitors can overcome resistance to dual PI3K/mTORi in human tumors (19) although the intolerability is the major problem in phase 1 clinical trials co-targeting these two signaling pathways (20) (21). Dysregulation of WNT/β-catenin signaling pathway has also been associated with dual PI3K/mTORi resistance (22) since β-catenin in the nucleus drives resistance to FOXO3a-induced apoptosis determined by PI3K inhibitors and AKT inhibitors (23). Notably, dual inhibition of PI3K/mTOR generates a positive feedback response that determines increased induction of JAK2/STAT5 and production of several cytokines including IL-8, thereby promoting PI3K inhibitor resistance (24). Suppression of PI3K/mTOR upregulates the JAK/STAT5 signaling pathway within 4 hours post-treatment, and for approximately 20 hours (25). Additionally, IL6-STAT3 loop has been found to mediate resistance to PI3K inhibitors by triggering epithelial-mesenchymal transition (EMT) and increasing cancer stem cell population in human breast cancer cells (26). Interestingly, JAK/STAT signaling pathway regulates PIM1 expression through direct binding of STAT3 and STAT5 to *PIM1* promoter, which consequently leads to its transcription (27). PIM1, which is notoriously overexpressed in multiple malignancies, can confer resistance to PI3K inhibitor by promoting the induction of downstream PI3K effectors in an AKT-independent manner (28). Moreover, PIM1 can regulate the activity of mTORC1 and eIF4B to increase NRF2/ARE activeness and reduce ROS production to diminish PI3K/AKT inhibitor-induced cytotoxic effects (29). Furthermore, at earlier time points IRS1 accumulation precedes JAK/STAT5 phosphorylation, suggesting that IRS1 could mediate the signalings of JAK/STAT, leading to a reduction in PI3K inhibitor susceptibility (25). Besides, dual suppression of PI3K/mTOR can also result in the induction of the Notch-MYC pathway (15) (30). Nonetheless, overexpression of HEY1, HEY2, or HES1, three NOTCH canonical target genes, displays no significant resistance to PI3K inhibitors (31). Dysregulation of hormone estrogen receptor (ER) signaling pathway has also been correlated with resistance to PI3K inhibitors. In fact, the majority of breast cancers express ER, which has been reported to remarkably correlate with mutations in *PIK3CA* (32). Increased PI3K activity, mainly due to its upregulation or hyperactive mutations, leads to endocrine therapy resistance (33). Indeed, PAM signaling pathway has been detected to strongly suppress ER-induced transcription through direct AKT1-mediated phosphorylation of KMT2D, thereby blocking its enzymatic activity and transcription. Upon drug-mediated PI3K suppression, induction of KMT2D endorses chromatin accessibility, which is necessary for ER activation, resulting in PI3K inhibitor resistance (34). Besides, serum and SGK1 protein, which is involved in phosphorylating consensus AKT motifs, downregulates KMT2D through a negative feedback loop. In fact, PI3K inhibition activates ER, which directly binds to SGK1 promoter, and thus, promotes its transcription. Consequently, SGK1 upregulation leads to phosphorylation/downregulation of KMT2D, thereby reducing and blocking ER signaling (35).

***Cellular plasticity***

Downregulation of some RTKs related to PAM signaling pathway can determine the production of genetically independent transcriptional programs, leading to cell populations tolerant to PI3K inhibitors or other inhibitory agents (36). Contrary to primary drug resistance, in this case cancer cells can transiently survive but are not capable to proliferate while drugged. These inhibitor-tolerant populations are able to escape initial therapy, but howbeit own no genetic mechanism to gain complete resistance necessary to consent cancer progression. Still, these populations can represent a source of carcinogenic cells with moderate cycling activity where a secondary genetic mechanism of acquired resistance to inhibitors may evolve from (37). Importantly, secondary resistance to targeted drug therapy and targeted immunotherapy is known to originate from these cancer cells (38). This protective feature is related to a phenotypic characteristic known as cell plasticity, where epithelial cancers gradually transit to a mesenchymal state (39) (1) (40) (41). Notably, this state can be reversed upon drug withdrawal, highlighting the absence of genetic mutations promoting this drug tolerance (37). Therefore, this EMT state has been closely related to chemoresistance by several studies during the last decades (42) (43). Nevertheless, upon PAM signaling pathway inhibition, there are still doubts regarding the initial mechanism/s inducing EMT reversal, since PI3K signalings are involved in both activating and inhibiting various EMT-related transcription factors. Further studies are necessary to unravel the individual factors to define the exact signaling network that regulates epigenome changes leading to EMT and chemoresistance (37). Notoriously, under selective pressure, tumor cells can further enter drug-tolerant state diapause, an environment-mediated changeful state occuring when the embryo is under hostile conditions (e.g. delayed blastocyte implantation). Interestingly, autophagy is enhanced prior to diapause initiation, and thus, is considered a crucial mediator of diapause. Notably, mTOR can inhibit autophagy (44), mainly by phosphorylation of ATG13 and ULK1, thereby preventing or interrupting its interaction with AMPK (45). Indeed, in patient-derived colorectal cancer models treated with ATP-competitive mTORi sapanisertib, cells enter a diapause drug-tolerant state, which is demonstrated by downregulation of mTOR and concurrent upregulation of autophagy. During discontinued treatment cells continue their proliferation, emphasizing the plasticity characteristics of these drug tolerant states. Interestingly, combination of autophagy inhibitors and chemotherapy significantly reduces cell survival, highlighting a possible novel therapy to contribute to targeting diapause-like state tumor cells. Importantly, both in EMT change-mediated drug-tolerant population, and in diapause-induced drug-tolerant population, cells cannot enter complete latency or dormancy, but instead continue in a moderate cycling-state, comparable to pluripotency. Therefore, using differentiation therapy, including leukaemia inhibitory factor (LIF) inhibitors or TGFβ inhibitors, may represent a beneficial potential strategy for future combinatorial targeted therapies with PAM signaling pathway inhibitors (46).

***Other mechanisms of resistance***

Since non-coding RNA (ncRNA) can modulate PAM signaling pathway and other parallel pathways including Ras/MEK/ERK (47), WNT/β-catenin (48), JAK/STAT (49), and Notch (50), ncRNA can also exert a critical role in PI3K inhibitor resistance (51) (52) (53). Indeed, several studies have emphasised that altered ncRNA expression is associated with cancer drug resistance (54) (55) (56) (57). Moreover, E3 ubiquitin ligase Skp2 correlates with the resistance of PI3K inhibition through the activation of AKT (25). Importantly, this resistance effect is reduced when either mTORC2 or PDK1 activity is suppressed (58), and results independent of PIP3 production, or PI3K activity. Since Skp2-induced ubiquitination of AKT is necessary for its recruitment to plasma membrane, Skp2 can thus increase the required localization of AKT, along with the cooperation of PDK1 and mTORC2, to result in its complete activation (59). Furthermore, macrophages in microenvironment also contribute to the resistance of PI3K inhibitors through the induction of NF-κB signaling pathway (60). Additionally, overexpression or amplification of RSK3 (61), RSK4 (61), PAK1 (62), CDK4 (63), CDK6 (63), MSK1 (64), IGFBP5 (65), and KDM6B (65), can contribute to PI3K inhibitor resistance. Likewise, upregulation of DNA methyltransferases, which decrease PPP2R2B and PTEN expression, is also considered another mechanism of acquired resistance towards dual PI3K/mTOR inhibitors (66).

**MECHANISMS OF RESISTANCE TO mTORC INHIBITORS**

***Other mechanisms of resistance to allosteric mTORi***

Rapalogs also determines activation of Ras/MEK/ERK signaling pathway through several mechanisms. Induction of ERK can occur via PI3K-mediated Rac/PAK1 activation, which consequently increases Raf induction, thereby promoting MEK/ERK overactivation (67). Alternatively, the adaptor protein GAB1 is recruited, leading to the activation of GRB2-SOS, and thus, resulting in Ras/Raf activation (68). Rapamycin, possibly through mTORC1/S6K inhibition, can activate c-Src, which trans-activates epidermal growth factor receptor (EGFR), leading to the induction of ERK pathway (69). Additionally, rapamycin-induced enhanced expression and phosphorylation of platelet-derived growth factor receptor-β (PDGFRβ) is also able to activate ERK signalling in hepatocellular carcinoma (70). Moreover, induction of ERK is often detected in aromatase inhibitor-resistant breast cancer cells displaying acquired everolimus resistance (71). mTORC2 contains mTOR kinase, as well as Rictor, GβL, and SIN1 subunits. S6K phosphorylation on Rictor reduces mTORC2 function, but conversely, mTORC1/S6K inhibition by rapamycin or similar agents can ultimately determine the re/activation of mTORC2 (72). Notably, mTORC2 phosphorylates and activates AKT (73), and as a result, loss of negative feedback on mTORC2 can produce induction of AKT, and upregulation of HIF (72) (74). Moreover, mutations in the FKBP12-rapamycin-binding (FRB) domain, including phenylalanine 2108 leucine substitution (F2108 L), and the alanine 2034 valine substitution (A2034 V), are involved in mediating rapalog resistance by obstructing cooperation between mTOR and FKBP12-rapamycin complex. Indeed, these types of mutations are generally present in tumor patients who relapsed during/after everolimus-based therapy (75).

***Other mechanisms of resistance to ATP-competitive mTORi***

In several tumor models, dual inhibition of mTORC1 and mTORC2 induces IGF1R/InsR-dependent PI3K activation, promotes AKT phosphorylation, and reorganises the integrin and focal adhesion kinase-mediated adhesomes, through an integrin/FAK/IGF1R-dependent signaling process (76). ATP-competitive mTORi can also activate a transcriptional response mediated by FOXO transcription factors that causes transcription of receptor tyrosine kinases RTKs. Indeed, AKT-induced phosphorylation of FOXO produces docking sites for 14-3-3 proteins, allowing the translocation of FOXO proteins from nucleus to cytoplasm. Conversely, inhibition of AKT induces nuclear localization of FOXO transcription factors, thereby promoting their transcriptional activity (77).

**ABBREVIATIONS**

**CSC**: Cancer stem cell

**EGFR**: Epidermal growth factor receptor (ErbB-1) (HER1)

**EMT**: Epithelial-mesenchymal transition

**ER**: Estrogen receptor

**FGFR1**: Fibroblast growth factor receptor 1

**FRB**: FKBP12-rapamycin-binding

**IGF1R**: Insulin-like growth factor 1 receptor

**InsR**: Insulin receptor

**IRS**: Insulin receptor substrate

**IRS1**: Insulin receptor substrate 1

**LIF**: Leukaemia inhibitory factor

**ncRNA**: Non-coding RNA

**ORR**: Overall response rate

**PAM**: PI3K/AKT/mTORC

**PDGFRβ**: Platelet-derived growth factor receptor beta

**ROS**: Reactive oxygen species

**REFERENCES**

1. Marine JC, Dawson SJ, Dawson MA. Non-genetic mechanisms of therapeutic resistance in cancer. Nat Rev Cancer. 2020;20(12):743-56.

2. Lunt SY, Vander Heiden MG. Aerobic glycolysis: meeting the metabolic requirements of cell proliferation. Annu Rev Cell Dev Biol. 2011;27:441-64.

3. Hoxhaj G, Manning BD. The PI3K-AKT network at the interface of oncogenic signalling and cancer metabolism. Nat Rev Cancer. 2020;20(2):74-88.

4. Khan KH, Wong M, Rihawi K, Bodla S, Morganstein D, Banerji U, et al. Hyperglycemia and Phosphatidylinositol 3-Kinase/Protein Kinase B/Mammalian Target of Rapamycin (PI3K/AKT/mTOR) Inhibitors in Phase I Trials: Incidence, Predictive Factors, and Management. Oncologist. 2016;21(7):855-60.

5. Barbour LA, Shao J, Qiao L, Leitner W, Anderson M, Friedman JE, et al. Human placental growth hormone increases expression of the p85 regulatory unit of phosphatidylinositol 3-kinase and triggers severe insulin resistance in skeletal muscle. Endocrinology. 2004;145(3):1144-50.

6. Goncalves MD, Hopkins BD, Cantley LC. Phosphatidylinositol 3-Kinase, Growth Disorders, and Cancer. N Engl J Med. 2018;379(21):2052-62.

7. Ando Y, Inada-Inoue M, Mitsuma A, Yoshino T, Ohtsu A, Suenaga N, et al. Phase I dose-escalation study of buparlisib (BKM120), an oral pan-class I PI3K inhibitor, in Japanese patients with advanced solid tumors. Cancer Sci. 2014;105(3):347-53.

8. Koh KX, Tan GH, Hui Low SH, Mohd Omar MF, Han MJ, Iacopetta B, et al. Acquired resistance to PI3K/mTOR inhibition is associated with mitochondrial DNA mutation and glycolysis. Oncotarget. 2017;8(66):110133-44.

9. Bhola NE, Jansen VM, Koch JP, Li H, Formisano L, Williams JA, et al. Treatment of Triple-Negative Breast Cancer with TORC1/2 Inhibitors Sustains a Drug-Resistant and Notch-Dependent Cancer Stem Cell Population. Cancer Res. 2016;76(2):440-52.

10. Nepstad I, Reikvam H, Brenner AK, Bruserud Ø, Hatfield KJ. Resistance to the Antiproliferative In Vitro Effect of PI3K-Akt-mTOR Inhibition in Primary Human Acute Myeloid Leukemia Cells Is Associated with Altered Cell Metabolism. Int J Mol Sci. 2018;19(2).

11. Castellano E, Downward J. RAS Interaction with PI3K: More Than Just Another Effector Pathway. Genes Cancer. 2011;2(3):261-74.

12. Ruicci KM, Pinto N, Khan MI, Yoo J, Fung K, MacNeil D, et al. ERK-TSC2 signalling in constitutively-active HRAS mutant HNSCC cells promotes resistance to PI3K inhibition. Oral Oncol. 2018;84:95-103.

13. Mundt F, Rajput S, Li S, Ruggles KV, Mooradian AD, Mertins P, et al. Mass Spectrometry-Based Proteomics Reveals Potential Roles of NEK9 and MAP2K4 in Resistance to PI3K Inhibition in Triple-Negative Breast Cancers. Cancer Res. 2018;78(10):2732-46.

14. Ilic N, Utermark T, Widlund HR, Roberts TM. PI3K-targeted therapy can be evaded by gene amplification along the MYC-eukaryotic translation initiation factor 4E (eIF4E) axis. Proc Natl Acad Sci U S A. 2011;108(37):E699-708.

15. Dey N, Leyland-Jones B, De P. MYC-xing it up with PIK3CA mutation and resistance to PI3K inhibitors: summit of two giants in breast cancers. Am J Cancer Res. 2015;5(1):1-19.

16. Chakrabarty A, Sánchez V, Kuba MG, Rinehart C, Arteaga CL. Feedback upregulation of HER3 (ErbB3) expression and activity attenuates antitumor effect of PI3K inhibitors. Proc Natl Acad Sci U S A. 2012;109(8):2718-23.

17. Serra V, Scaltriti M, Prudkin L, Eichhorn PJ, Ibrahim YH, Chandarlapaty S, et al. PI3K inhibition results in enhanced HER signaling and acquired ERK dependency in HER2-overexpressing breast cancer. Oncogene. 2011;30(22):2547-57.

18. Young CD, Zimmerman LJ, Hoshino D, Formisano L, Hanker AB, Gatza ML, et al. Activating PIK3CA Mutations Induce an Epidermal Growth Factor Receptor (EGFR)/Extracellular Signal-regulated Kinase (ERK) Paracrine Signaling Axis in Basal-like Breast Cancer. Mol Cell Proteomics. 2015;14(7):1959-76.

19. Mohan S, Vander Broek R, Shah S, Eytan DF, Pierce ML, Carlson SG, et al. MEK Inhibitor PD-0325901 Overcomes Resistance to PI3K/mTOR Inhibitor PF-5212384 and Potentiates Antitumor Effects in Human Head and Neck Squamous Cell Carcinoma. Clin Cancer Res. 2015;21(17):3946-56.

20. Mita M, Fu S, Piha-Paul SA, Janku F, Mita A, Natale R, et al. Phase I trial of MEK 1/2 inhibitor pimasertib combined with mTOR inhibitor temsirolimus in patients with advanced solid tumors. Invest New Drugs. 2017;35(5):616-26.

21. Schram AM, Gandhi L, Mita MM, Damstrup L, Campana F, Hidalgo M, et al. A phase Ib dose-escalation and expansion study of the oral MEK inhibitor pimasertib and PI3K/MTOR inhibitor voxtalisib in patients with advanced solid tumours. Br J Cancer. 2018;119(12):1471-6.

22. Park YL, Kim HP, Cho YW, Min DW, Cheon SK, Lim YJ, et al. Activation of WNT/β-catenin signaling results in resistance to a dual PI3K/mTOR inhibitor in colorectal cancer cells harboring PIK3CA mutations. Int J Cancer. 2019;144(2):389-401.

23. Tenbaum SP, Ordóñez-Morán P, Puig I, Chicote I, Arqués O, Landolfi S, et al. β-catenin confers resistance to PI3K and AKT inhibitors and subverts FOXO3a to promote metastasis in colon cancer. Nat Med. 2012;18(6):892-901.

24. Britschgi A, Andraos R, Brinkhaus H, Klebba I, Romanet V, Müller U, et al. JAK2/STAT5 inhibition circumvents resistance to PI3K/mTOR blockade: a rationale for cotargeting these pathways in metastatic breast cancer. Cancer Cell. 2012;22(6):796-811.

25. Clement E, Inuzuka H, Nihira NT, Wei W, Toker A. Skp2-dependent reactivation of AKT drives resistance to PI3K inhibitors. Sci Signal. 2018;11(521).

26. Yang L, Han S, Sun Y. An IL6-STAT3 loop mediates resistance to PI3K inhibitors by inducing epithelial-mesenchymal transition and cancer stem cell expansion in human breast cancer cells. Biochem Biophys Res Commun. 2014;453(3):582-7.

27. Bachmann M, Möröy T. The serine/threonine kinase Pim-1. Int J Biochem Cell Biol. 2005;37(4):726-30.

28. Le X, Antony R, Razavi P, Treacy DJ, Luo F, Ghandi M, et al. Systematic Functional Characterization of Resistance to PI3K Inhibition in Breast Cancer. Cancer Discov. 2016;6(10):1134-47.

29. Song JH, Singh N, Luevano LA, Padi SKR, Okumura K, Olive V, et al. Mechanisms Behind Resistance to PI3K Inhibitor Treatment Induced by the PIM Kinase. Mol Cancer Ther. 2018;17(12):2710-21.

30. Liu P, Cheng H, Santiago S, Raeder M, Zhang F, Isabella A, et al. Oncogenic PIK3CA-driven mammary tumors frequently recur via PI3K pathway-dependent and PI3K pathway-independent mechanisms. Nat Med. 2011;17(9):1116-20.

31. Muellner MK, Uras IZ, Gapp BV, Kerzendorfer C, Smida M, Lechtermann H, et al. A chemical-genetic screen reveals a mechanism of resistance to PI3K inhibitors in cancer. Nat Chem Biol. 2011;7(11):787-93.

32. Ciriello G, Gatza ML, Beck AH, Wilkerson MD, Rhie SK, Pastore A, et al. Comprehensive Molecular Portraits of Invasive Lobular Breast Cancer. Cell. 2015;163(2):506-19.

33. Zhou W, Slingerland JM. Links between oestrogen receptor activation and proteolysis: relevance to hormone-regulated cancer therapy. Nat Rev Cancer. 2014;14(1):26-38.

34. Toska E, Osmanbeyoglu HU, Castel P, Chan C, Hendrickson RC, Elkabets M, et al. PI3K pathway regulates ER-dependent transcription in breast cancer through the epigenetic regulator KMT2D. Science. 2017;355(6331):1324-30.

35. Toska E, Castel P, Chhangawala S, Arruabarrena-Aristorena A, Chan C, Hristidis VC, et al. PI3K Inhibition Activates SGK1 via a Feedback Loop to Promote Chromatin-Based Regulation of ER-Dependent Gene Expression. Cell Rep. 2019;27(1):294-306.e5.

36. Boumahdi S, de Sauvage FJ. The great escape: tumour cell plasticity in resistance to targeted therapy. Nat Rev Drug Discov. 2020;19(1):39-56.

37. Arozarena I, Wellbrock C. Phenotype plasticity as enabler of melanoma progression and therapy resistance. Nat Rev Cancer. 2019;19(7):377-91.

38. Rambow F, Rogiers A, Marin-Bejar O, Aibar S, Femel J, Dewaele M, et al. Toward Minimal Residual Disease-Directed Therapy in Melanoma. Cell. 2018;174(4):843-55.e19.

39. Vasan N, Baselga J, Hyman DM. A view on drug resistance in cancer. Nature. 2019;575(7782):299-309.

40. Ashrafizadeh M, Hushmandi K, Hashemi M, Akbari ME, Kubatka P, Raei M, et al. Role of microRNA/Epithelial-to-Mesenchymal Transition Axis in the Metastasis of Bladder Cancer. Biomolecules. 2020;10(8).

41. Hwang ST, Yang MH, Kumar AP, Sethi G, Ahn KS. Corilagin Represses Epithelial to Mesenchymal Transition Process Through Modulating Wnt/β-Catenin Signaling Cascade. Biomolecules. 2020;10(10).

42. Shibue T, Weinberg RA. EMT, CSCs, and drug resistance: the mechanistic link and clinical implications. Nat Rev Clin Oncol. 2017;14(10):611-29.

43. Cheng JT, Wang L, Wang H, Tang FR, Cai WQ, Sethi G, et al. Insights into Biological Role of LncRNAs in Epithelial-Mesenchymal Transition. Cells. 2019;8(10).

44. Lin JZ, Wang WW, Hu TT, Zhu GY, Li LN, Zhang CY, et al. FOXM1 contributes to docetaxel resistance in castration-resistant prostate cancer by inducing AMPK/mTOR-mediated autophagy. Cancer Lett. 2020;469:481-9.

45. Rehman SK, Haynes J, Collignon E, Brown KR, Wang Y, Nixon AML, et al. Colorectal Cancer Cells Enter a Diapause-like DTP State to Survive Chemotherapy. Cell. 2021;184(1):226-42.e21.

46. Pascual-García M, Bonfill-Teixidor E, Planas-Rigol E, Rubio-Perez C, Iurlaro R, Arias A, et al. LIF regulates CXCL9 in tumor-associated macrophages and prevents CD8. Nat Commun. 2019;10(1):2416.

47. Chen L, Feng P, Zhu X, He S, Duan J, Zhou D. Long non-coding RNA Malat1 promotes neurite outgrowth through activation of ERK/MAPK signalling pathway in N2a cells. J Cell Mol Med. 2016;20(11):2102-10.

48. Yu B, Ye X, Du Q, Zhu B, Zhai Q, Li XX. The Long Non-Coding RNA CRNDE Promotes Colorectal Carcinoma Progression by Competitively Binding miR-217 with TCF7L2 and Enhancing the Wnt/β-Catenin Signaling Pathway. Cell Physiol Biochem. 2017;41(6):2489-502.

49. Wang S, Liang K, Hu Q, Li P, Song J, Yang Y, et al. JAK2-binding long noncoding RNA promotes breast cancer brain metastasis. J Clin Invest. 2017;127(12):4498-515.

50. Reicher A, Foßelteder J, Kwong LN, Pichler M. Crosstalk between the Notch signaling pathway and long non-coding RNAs. Cancer Lett. 2018;420:91-6.

51. Garg M, Shanmugam MK, Bhardwaj V, Goel A, Gupta R, Sharma A, et al. The pleiotropic role of transcription factor STAT3 in oncogenesis and its targeting through natural products for cancer prevention and therapy. Med Res Rev. 2020.

52. Arora L, Kumar AP, Arfuso F, Chng WJ, Sethi G. The Role of Signal Transducer and Activator of Transcription 3 (STAT3) and Its Targeted Inhibition in Hematological Malignancies. Cancers (Basel). 2018;10(9).

53. Lee JH, Chiang SY, Nam D, Chung WS, Lee J, Na YS, et al. Capillarisin inhibits constitutive and inducible STAT3 activation through induction of SHP-1 and SHP-2 tyrosine phosphatases. Cancer Lett. 2014;345(1):140-8.

54. Wang BD, Ceniccola K, Hwang S, Andrawis R, Horvath A, Freedman JA, et al. Alternative splicing promotes tumour aggressiveness and drug resistance in African American prostate cancer. Nat Commun. 2017;8:15921.

55. Mishra S, Verma SS, Rai V, Awasthee N, Chava S, Hui KM, et al. Long non-coding RNAs are emerging targets of phytochemicals for cancer and other chronic diseases. Cell Mol Life Sci. 2019;76(10):1947-66.

56. Ma Z, Wang YY, Xin HW, Wang L, Arfuso F, Dharmarajan A, et al. The expanding roles of long non-coding RNAs in the regulation of cancer stem cells. Int J Biochem Cell Biol. 2019;108:17-20.

57. Pandya G, Kirtonia A, Sethi G, Pandey AK, Garg M. The implication of long non-coding RNAs in the diagnosis, pathogenesis and drug resistance of pancreatic ductal adenocarcinoma and their possible therapeutic potential. Biochim Biophys Acta Rev Cancer. 2020;1874(2):188423.

58. Chan CH, Li CF, Yang WL, Gao Y, Lee SW, Feng Z, et al. The Skp2-SCF E3 ligase regulates Akt ubiquitination, glycolysis, herceptin sensitivity, and tumorigenesis. Cell. 2012;149(5):1098-111.

59. Rexer BN, Chanthaphaychith S, Dahlman K, Arteaga CL. Direct inhibition of PI3K in combination with dual HER2 inhibitors is required for optimal antitumor activity in HER2+ breast cancer cells. Breast Cancer Res. 2014;16(1):R9.

60. Usman MW, Gao J, Zheng T, Rui C, Li T, Bian X, et al. Macrophages confer resistance to PI3K inhibitor GDC-0941 in breast cancer through the activation of NF-κB signaling. Cell Death Dis. 2018;9(8):809.

61. Serra V, Eichhorn PJ, García-García C, Ibrahim YH, Prudkin L, Sánchez G, et al. RSK3/4 mediate resistance to PI3K pathway inhibitors in breast cancer. J Clin Invest. 2013;123(6):2551-63.

62. Walsh K, McKinney MS, Love C, Liu Q, Fan A, Patel A, et al. PAK1 mediates resistance to PI3K inhibition in lymphomas. Clin Cancer Res. 2013;19(5):1106-15.

63. Vora SR, Juric D, Kim N, Mino-Kenudson M, Huynh T, Costa C, et al. CDK 4/6 inhibitors sensitize PIK3CA mutant breast cancer to PI3K inhibitors. Cancer Cell. 2014;26(1):136-49.

64. Wu S, Wang S, Zheng S, Verhaak R, Koul D, Yung WK. MSK1-Mediated β-Catenin Phosphorylation Confers Resistance to PI3K/mTOR Inhibitors in Glioblastoma. Mol Cancer Ther. 2016;15(7):1656-68.

65. Wang W, Lim KG, Feng M, Bao Y, Lee PL, Cai Y, et al. KDM6B Counteracts EZH2-Mediated Suppression of IGFBP5 to Confer Resistance to PI3K/AKT Inhibitor Treatment in Breast Cancer. Mol Cancer Ther. 2018;17(9):1973-83.

66. Qian XJ, Li YT, Yu Y, Yang F, Deng R, Ji J, et al. Inhibition of DNA methyltransferase as a novel therapeutic strategy to overcome acquired resistance to dual PI3K/mTOR inhibitors. Oncotarget. 2015;6(7):5134-46.

67. Ebi H, Costa C, Faber AC, Nishtala M, Kotani H, Juric D, et al. PI3K regulates MEK/ERK signaling in breast cancer via the Rac-GEF, P-Rex1. Proc Natl Acad Sci U S A. 2013;110(52):21124-9.

68. Borisov N, Aksamitiene E, Kiyatkin A, Legewie S, Berkhout J, Maiwald T, et al. Systems-level interactions between insulin-EGF networks amplify mitogenic signaling. Mol Syst Biol. 2009;5:256.

69. Chaturvedi D, Gao X, Cohen MS, Taunton J, Patel TB. Rapamycin induces transactivation of the EGFR and increases cell survival. Oncogene. 2009;28(9):1187-96.

70. Li QL, Gu FM, Wang Z, Jiang JH, Yao LQ, Tan CJ, et al. Activation of PI3K/AKT and MAPK pathway through a PDGFRβ-dependent feedback loop is involved in rapamycin resistance in hepatocellular carcinoma. PLoS One. 2012;7(3):e33379.

71. Kimura M, Hanamura T, Tsuboi K, Kaneko Y, Yamaguchi Y, Niwa T, et al. Acquired resistance to everolimus in aromatase inhibitor-resistant breast cancer. Oncotarget. 2018;9(30):21468-77.

72. Julien LA, Carriere A, Moreau J, Roux PP. mTORC1-activated S6K1 phosphorylates Rictor on threonine 1135 and regulates mTORC2 signaling. Mol Cell Biol. 2010;30(4):908-21.

73. Feldman ME, Apsel B, Uotila A, Loewith R, Knight ZA, Ruggero D, et al. Active-site inhibitors of mTOR target rapamycin-resistant outputs of mTORC1 and mTORC2. PLoS Biol. 2009;7(2):e38.

74. Petrossian K, Nguyen D, Lo C, Kanaya N, Somlo G, Cui YX, et al. Use of dual mTOR inhibitor MLN0128 against everolimus-resistant breast cancer. Breast Cancer Res Treat. 2018;170(3):499-506.

75. Wagle N, Grabiner BC, Van Allen EM, Amin-Mansour A, Taylor-Weiner A, Rosenberg M, et al. Response and acquired resistance to everolimus in anaplastic thyroid cancer. N Engl J Med. 2014;371(15):1426-33.

76. Yoon SO, Shin S, Karreth FA, Buel GR, Jedrychowski MP, Plas DR, et al. Focal Adhesion- and IGF1R-Dependent Survival and Migratory Pathways Mediate Tumor Resistance to mTORC1/2 Inhibition. Mol Cell. 2017;67(3):512-27.e4.

77. Yan Y, Serra V, Prudkin L, Scaltriti M, Murli S, Rodríguez O, et al. Evaluation and clinical analyses of downstream targets of the Akt inhibitor GDC-0068. Clin Cancer Res. 2013;19(24):6976-86.
