# Supplementary material for: PI3K/AKT/mTOR signaling transduction pathway and targeted therapies in cancer
Source: Mol Cancer. 2023 Aug 18;22:138. doi: 10.1186/s12943-023-01827-6 (PMC10436543; doi:10.1186/s12943-023-01827-6)
Supplement: Supplementary file 5 — Additional file 5: Supplementary table 1. [file 12943_2023_1827_MOESM5_ESM.docx]

| **PAN-PI3K INHIBITORS** | | | | | | | | | | | | |
| --- | --- | --- | --- | --- | --- | --- | --- | --- | --- | --- | --- | --- |
| **Buparlisib** (BKM120, NVP-BKM120) | | | | | | | | | | | | |
| **Phase** | **Treatment** | **Disease** | **Outcome** | **ISP** | **Status** | **FP** | **RFP** | **LUP** | **Sponsor** | **Collaborator** | **NCT Identifier** | **R** |
| 1 | Buparlisib | Advanced acute leukemia | OS: 75 days; reduction of p-S6K (65%); decrease of p-FOXO3 (93%). MTD: 80 mg/daily | F | C | 2011 | * | 2016 | M.D. Anderson Cancer Center | Novartis | NCT01396499 | 9 |
| 1 | Buparlisib | Advanced solid tumor | PR: 3.2% (triple-negative breast cancer with a KRAS mutation); SD: 52% for more than 6 weeks. MTD: 100 mg/daily | F | C | 2010 | * | 2020 | Novartis | * | NCT01068483 | 10 |
| 1 | Buparlisib | Advanced solid tumor | SD: 40%; DCR: 40%; progressive disease: 46.7%. RP2D: 100 mg/day | A | C | 2011 | * | 2020 | Novartis | * | NCT01283503 | 11 |
| 1 | Combination of buparlisib with bevacizumab | Metastatic renal cell carcinoma | PR: 13.3%, and SD: 50%, There was a significant maximal tumor shrinkage in 6.2% of patients. MTD: 80 mg/daily | A | C | 2011 | * | 2016 | Dana-Farber Cancer Institute | Beth Israel Deaconess Medical Center | NCT01283048 | 12 |
| 1 | Combination of buparlisib with capecitabine | Metastatic breast cancer | PR: 23.5%; CR: 5.8% | F | C | 2011 | * | 2020 | UNC Lineberger Comprehensive Cancer Center | Novartis | NCT01300962 | 13 |
| 1 | Combination of buparlisib with fulvestrant | ER+ metastatic breast cancer | CBR: 58.6%. Loss of PTEN, or progesterone receptor (PgR) expression, or mutation in TP53 was commoner in resistant cases. MTD: 100mg/once daily | A | C | 2011 | * | 2017 | Washington University School of Medicine | National Cancer Institute (NCI) | NCT01339442 | 14 |
| 1 | Combination of buparlisib with everolimus | Advanced solid tumor | PFS: 2.7 months; OS: 9 months; progressive disease: 11%; SD: 89%. MTD and RP2D: 60 mg/daily on a continuous schedule | F | C | 2011 | * | 2018 | Emory University | Novartis | NCT01470209 | 15 |
| 1, 1b | Combination of buparlisib with Ibrutinib | Relapsed or refractory B-cell lymphoma | ORR: 94%, and 33-month median PFS in mantle cell lymphoma. ORR: 31% in diffuse large B-cell lymphoma. ORR: 20% in follicular lymphoma. MTD: 100 mg (buparlisib) + 560 mg (ibrutinib). RP2D: 80 mg (buparlisib) + 560 mg (ibrutinib) | F | A, NR | 2016 | * | 2022 | Memorial Sloan Kettering Cancer Center | Janssen Scientific Affairs LLC, and  Novartis | NCT02756247 | 16 |
| 1b | Combination of buparlisib with panitumumab | Metastatic and advanced Ras wild-type colorectal cancer | PFS: 2 months; SD: 36.8%; progression disease: 47.3% | A | C | 2012 | * | 2020 | Canadian Cancer Trials Group | Pfizer | NCT01591421 | 17 |
| 1b | Combination of buparlisib with trametinib | Advanced solid tumor | PFS: 7 months, and ORR: 28.6% (ovarian cancer). PFS: 4 months, and ORR: 53% (non-small-cell lung carcinoma). PFS: 2 months (pancreatic cancer) | A | C | 2010 | * | 2020 | Novartis | * | NCT01155453 | 18 |
| 1b | Combination of buparlisib with paclitaxel ± trastuzumab | HER2+ breast cancer and advanced solid tumor | ORR: 17% with buparlisib + paclitaxel (advanced solid tumor). ORR: 27% with buparlisib + paclitaxel + trastuzumab (HER2+ breast cancer). MTD (for both combinations): 100 mg/day | F | C | 2011 | * | 2020 | Novartis | * | NCT01285466 | 19 |
| 1b | Triple combination of buparlisib with tamoxifen and goserelin | HR+, HER2- advanced breast cancer | PFS: 20.6 months. RP2D: 100 mg/once daily | F | C | 2014 | * | 2020 | Novartis | * | NCT02058381 | 20 |
| 1b | Triple combination of buparlisib with carboplatin and paclitaxel | Advanced solid tumor | ORR: 20% | F | C | 2011 | * | 2017 | Memorial Sloan Kettering Cancer Center | Novartis, and  Sai Life Sciences | NCT01297452 | 21 |
| 1, 2 | Combination of buparlisib with bevacizumab | Relapsed or refractory glioblastoma multiforme | PFS: 5.3 months; OS: 10.8 months; progressive disease: 50% | F | C | 2011 | 2018 | 2020 | SCRI Development Innovations, LLC | Novartis | NCT01349660 | 22 |
| 1, 2 | Combination of buparlisib with cetuximab | Recurrent or metastatic head and neck cancer | OS: 280 days; SD: 40% | F | C | 2013 | 2020 | 2021 | University of Chicago | National Cancer Institute (NCI) | NCT01816984 | 23 |
| 2 | Buparlisib | Relapsed or refractory non-Hodgkin lymphoma | ORR: 25%, PFS: 9.8 months, and OS: 12.1 months (follicular lymphoma). ORR: 11.5%, PFS: 1.8 months, and OS: 5.2 months (diffuse large B-cell lymphoma). ORR: 22.7%, PFS: 11.3 months, and OS: 8.2 months (mantle cell lymphoma) | A | C | 2012 | 2018 | 2018 | Novartis | * | NCT01693614 | 24 |
| 2 | Buparlisib | Metastatic triple-negative breast cancer | PFS: 1.8 months; OS: 11.2 months | F | C | 2012 | 2020 | 2020 | SOLTI Breast Cancer Research Group | Novartis, and Dana-Farber Cancer Institute | NCT01629615 | 25 |
| 2 | Buparlisib | Melanoma brain metastases | DOR: 117 days, PFS: 42 days, and OS: 5 months. Intracranial SD: 17.6% | F | U | 2015 | * | 2017 | University Hospital Tuebingen | University Hospital Dresden | NCT02452294 | 26 |
| 2 | Combination of buparlisib with cetuximab | Recurrent or metastatic head and neck squamous cell carcinoma | DCR: 91%, ORR: 18%, PFS: 111 days, and OS: 206 days with buparlisib + cetuximab vs DCR: 49%, ORR: 3%, PFS: 63 days, and OS: 143 days with buparlisib monotherapy | F | U | 2012 | * | 2012 | Yonsei University | * | NCT01527877 | 27 |
| **CH5132799** (CH5) | | | | | | | | | | | | |
| **Phase** | **Treatment** | **Disease** | **Outcome** | **ISP** | **Status** | **FP** | **RFP** | **LUP** | **Sponsor** | **Collaborator** | **NCT Identifier** | **R** |
| 1 | CH5132799 | Advanced solid tumour | Inhibition of p-AKT; SD: 21% up to 4 months (5.2% harboured a PIK3CA mutation). MTD: 48 mg/twice daily | F | C | 2010 | * | 2014 | Chugai Pharma Europe Ltd | * | NCT01222546 | 30 |
| **Copanlisib** (BAY 80-6946, Aliqopa) | | | | | | | | | | | | |
| **Phase** | **Treatment** | **Disease** | **Outcome** | **ISP** | **Status** | **FP** | **RFP** | **LUP** | **Sponsor** | **Collaborator** | **NCT Identifier** | **R** |
| 1 | Copanlisib | Non-Hodgkin lymphoma | CR: 16.6%, and PR: 83.3% (follicular lymphoma). MTD: 0.8 mg/kg (treatment on day 1, day 8, and day 15 of a month cycle) | F | C | 2009 | * | 2017 | Bayer | * | NCT00962611 | 36 |
| 1 | Copanlisib | Advanced or refractory solid tumor | DCR: 40%. MTD: 0.8 mg/kg | F | C | 2011 | * | 2017 | Bayer | * | NCT01404390 | 37 |
| 1 | Combination of copanlisib with ibrutinib | Relapsed or refractory mantle cell lymphoma | ORR: 87.5% with CR in 50% of patients, and PR in 37.5% of patients. Median PFS: 7.7 months. MTD: 45 mg (copanlisib) + 560 mg (ibrutinib) | A | A, NR | 2019 | * | 2022 | Memorial Sloan Kettering Cancer Center | Bayer | NCT03877055 | 38 |
| 1 | Combination of copanlisib with gemcitabine ± cisplatin | Advanced cancer | MTD and RP2D: 0.8 mg kg^−1^ on days 1, 8 and 15 of a 28-day treatment cycle, for both copanlisib + gemcitabine, and copanlisib + gemcitabine + cisplatin | A | C | 2011 | * | 2017 | Bayer | * | NCT01460537 | 39 |
| 1b | Combination of copanlisib with refametinib | Advanced cancer | SD: 32.8%; decreased tumor fluorodeoxyglucose (FDG) uptake. MTD: 0.4 mg/kg weekly | F | C | 2011 | * | 2015 | Bayer | * | NCT01392521 | 40 |
| 1, 2 | Combination of copanlisib with gemcitabine | Relapsed and refractory peripheral T-cell lymphoma | ORR: 72%; CR: 32%; PFS: 6.9 months; DOR: 8.2 months | F | C | 2017 | * | 2022 | Chonnam National University Hospital | Bayer, and Consortium for Improving Survival of Lymphoma | NCT03052933 | 41 |
| 2 | Copanlisib | Relapsed, indolent, or aggressive non-Hodgkin lymphoma | ORR: 43.7%, PFS: 294 days, and DOR: 390 days (relapsed, indolent, non-Hodgkin lymphoma). ORR: 27.1%, PFS: 70 days, and DOR: 166 days (relapsed, aggressive non-Hodgkin lymphoma) | A | A, NR | 2012 | 2018 | 2022 | Bayer | * | NCT01660451 | 42 |
| 2 | Copanlisib | Relapse or recurrent diffuse large B-cell lymphoma | ORR: 19.4%, PFS: 1.8 months, and DOR: 4.3 months. | A | C | 2015 | 2018 | 2019 | Bayer | * | NCT02391116 | 43 |
| 2 | Copanlisib | Relapsed or refractory indolent B-cell lymphoma | ORR: 59%; CR: 12%; PFS: 11.2 months; DOR: 22.6 months | A | A, NR | 2012 | 2018 | 2022 | Bayer | * | NCT01660451 | 44 |
| 2 | Copanlisib | Advanced refractory solid tumor with PIK3CA mutations | ORR: 16% in select tumors with PIK3CA mutation in the refractory setting | A | A, R | 2015 | * | 2022 | National Cancer Institute (NCI) | * | NCT02465060 | 45 |
| 2 | Triple combination of copanlisib with gemcitabine and cisplatin | Advanced biliary tract cancer | OS: 13.7 months, PFS: 6.2 months, and PFS rate at 6 months: 51%, PR: 31.6%, and SD: 57.9%. There was no significant difference in survival with the addition of copanlisib in comparison with gemcitabine + cisplatin | F | C | 2015 | 2020 | 2021 | H. Lee Moffitt Cancer Center and Research Institute | Bayer | NCT02631590 | 46 |
| 3 | Combination of copanlisib with rituximab | Relapsed indolent non-Hodgkin lymphoma | PFS: 21.5 months with copanlisib + rituximab vs PFS: 13.8 months with placebo + rituximab | A | A, NR | 2015 | 2022 | 2022 | Bayer | * | NCT02367040 | 47 |
| 3 | Combination of copanlisib with bendamustine | Relapsed Indolent B-cell lymphoma | ORR: 90%, CR: 50%, and PR: 40%. RP3D: 60 mg/weekly | F | A, NR | 2015 | * | 2022 | Bayer | * | NCT02626455 | 48 |
| 3 | Combination of copanlisib with rituximab, cyclophosphamide, doxorubicin, vincristine, and prednisone | Relapsed Indolent B-cell lymphoma | ORR: 100%, CR: 30%, and PR: 70%. RP3D: 60 mg/weekly | F | A, NR | 2015 | * | 2022 | Bayer | * | NCT02626455 | 48 |
| **Pictilisib** (GDC-0941, RG7321) | | | | | | | | | | | | |
| **Phase** | **Treatment** | **Disease** | **Outcome** | **ISP** | **Status** | **FP** | **RFP** | **LUP** | **Sponsor** | **Collaborator** | **NCT Identifier** | **R** |
| 1 | Pictilisib | Advanced solid tumor | Concentration-dependent reduction of pAKT in platelet-rich plasma; decrease of pS6 and pAKT in cancer biopsies. MTD and RP2D: 330 mg/once daily | F | C | 2009 | * | 2016 | Genentech, Inc | * | NCT00876122 | 55 |
| 1 | Combination of pictilisib with erlotinib | Advanced solid tumor | PR: 3.5%; SD: 33.3%. RP2D: 340 mg (5 days on/2 days off schedule) | A | C | 2009 | * | 2016 | Genentech, Inc | * | NCT00975182 | 56 |
| 1b | Combination of pictilisib with letrozole | Metastatic or locally recurrent breast cancer | PFS: 5.4 months | A | C | 2009 | * | 2016 | Genentech, Inc | * | NCT00960959 | 57 |
| 1b | Combination of pictilisib with paclitaxel ± bevacizumab or trastuzumab | Metastatic or locally recurrent breast cancer | PFS: 5 months with pictilisib + paclitaxel. PFS: 5.8 months with pictilisib + paclitaxel + bevacizumab. PFS: 14.8 months with pictilisib + paclitaxel + trastuzumab | A | C | 2009 | * | 2016 | Genentech, Inc | * | NCT00960960 | 57 |
| 1b | Triple combination of pictilisib with paclitaxel and carboplatin ± bevacizumab | Advanced non-small cell lung cancer (NSCLC) | PR: 50% and SD: 16.7% with pictilisib + paclitaxel + carboplatin. PR: 37.5% and SD: 37.5% with pictilisib + paclitaxel + carboplatin + bevacizumab | A | C | 2009 | * | 2016 | Genentech, Inc | * | NCT00974584 | 58 |
| 1b | Triple combination of pictilisib with pemetrexed and cisplatin ± bevacizumab | Advanced non-small cell lung cancer (NSCLC) | PR: 18.2% and SD: 45.5% with pictilisib + pemetrexed + cisplatin. PR: 69.2% and SD: 23.1% with pictilisib + pemetrexed + cisplatin + bevacizumab | A | C | 2009 | * | 2016 | Genentech, Inc | * | NCT00974584 | 58 |
| 2 | Combination of pictilisib with anastrozole | Estrogen receptor-positive, HER2 negative breast cancer | Suppression of Ki-67: 83.8% with pictilisib + anastrozole vs 66% with anastrozole singularly | F | C | 2013 | * | 2015 | Brighton & Sussex University Hospitals NHS Trust | * | ISRCTN26131497 | 59 |
| **Pilaralisib** (XL147, SAR-245408) | | | | | | | | | | | | |
| **Phase** | **Treatment** | **Disease** | **Outcome** | **ISP** | **Status** | **FP** | **RFP** | **LUP** | **Sponsor** | **Collaborator** | **NCT Identifier** | **R** |
| 1 | Pilaralisib | Advanced solid tumor | SD: 27.7%. RP2D: 600 mg/daily | F | C | 2013 | * | 2016 | Sanofi | * | NCT01943838 | 61 |
| 1 | Pilaralisib | Advanced solid tumor | PFS: 11.6% at 6 months; 40%-80% reduction in phosphorylation of AKT, S6, PRAS40, and 4EBP1. RP2D: 600 mg/once daily | F | C | 2007 | * | 2013 | Sanofi | * | NCT00486135 | 62 |
| 1 | Combination of pilaralisib with erlotinib | Advanced solid tumor | PR: 3.7%, and SD: 51.9%. MTD: 400 mg/once daily | F | C | 2008 | * | 2012 | Sanofi | * | NCT00692640 | 63 |
| 1 | Triple combination of pilaralisib with paclitaxel and carboplatin | Advanced solid tumor | PR: 13.5%. MTD: 200 mg/once daily. Pilaralisib did not significantly enhance the antitumor activity of paclitaxel + carboplatin | F | C | 2008 | * | 2013 | Sanofi | * | NCT00756847 | 64 |
| 1, 2 | Combination of pilaralisib with letrozole | HR+, HER2- metastatic breast cancer refractory to a non-steroidal aromatase inhibitor | ORR: 4%; PFS rate at 6 months: 17%; PFS: 8 weeks; PR: 4%; SD: 41.7%; progressive disease: 45.8%. MTD: 400 mg/once daily | A | C | 2010 | * | 2016 | Sanofi | * | NCT01082068 | 65 |
| 1, 2 | Combination of pilaralisib with trastuzumab ± paclitaxel | Trastuzumab-refractory HER2+ metastatic breast cancer | PFS: 11 weeks (5.3% of patients showed PFS for more than 24 weeks) with pilaralisib + trastuzumab. PFS: 21.1 weeks (40% of patients showed PFS for more than 24 weeks), SD: 55%, and PR: 20%, with pilaralisib + trastuzumab + paclitaxel. MTD (for both combinations): 400 mg/once daily | A | C | 2010 | * | 2016 | Sanofi | * | NCT01042925 | 66 |
| 2 | Pilaralisib | Previously treated advanced-stage endometrial carcinoma | ORR: 6%; 6-month PFS rate: 11.9% | F | C | 2009 | * | 2016 | Sanofi | * | NCT01013324 | 67 |
| **Sonolisib** (PX-866) | | | | | | | | | | | | |
| **Phase** | **Treatment** | **Disease** | **Outcome** | **ISP** | **Status** | **FP** | **RFP** | **LUP** | **Sponsor** | **Collaborator** | **NCT Identifier** | **R** |
| 1 | Sonolisib | Advanced solid tumour | SD: 53% (continuous schedule), and SD: 22% (intermittent schedule). MTD: 8 mg/once daily (continuous schedule), and 12 mg/once daily (intermittent schedule) | F | C | 2008 | * | 2018 | Cascadian Therapeutics, Inc | * | NCT00726583 | 72 |
| 1 | Combination of sonolisib with docetaxel | Advanced solid tumour | PR: 4.6% | F | C | 2010 | * | 2018 | Cascadian Therapeutics, Inc | * | NCT01204099 | 73 |
| 2 | Sonolisib | Glioblastoma multiforme at time of first relapse or progression | SD: 21%; PFS: 17% at 6 months | F | C | 2010 | 2020 | 2020 | NCIC Clinical Trials Group | Cascadian Therapeutics, Inc | NCT01259869 | 74 |
| 2 | Sonolisib | Recurrent or metastatic castration-resistant prostate cancer | PFS: 33% at 12 weeks; PR: 14.2%; confirmed prostate-specific antigen (PSA) response: 7.1% | F | C | 2011 | 2020 | 2020 | NCIC Clinical Trials Group | Oncothyreon Canada, Inc | NCT01331083 | 75 |
| **ISOFORM SPECIFIC-PI3K INHIBITORS** | | | | | | | | | | | | |
| **Alpelisib** (BYL719, Piqray) [PI3Kα Inhibitor] | | | | | | | | | | | | |
| **Phase** | **Treatment** | **Disease** | **Outcome** | **ISP** | **Status** | **FP** | **RFP** | **LUP** | **Sponsor** | **Collaborator** | **NCT Identifier** | **R** |
| 1 | Alpelisib | PIK3CA-altered advanced solid tumor | SD: 52.2%; PR: 5.2%; CR: 0.7%. MTD: 400 mg/daily, and 150 mg/twice daily | F | C | 2010 | * | 2020 | Novartis | * | NCT01219699 | 85 |
| 1 | Alpelisib | Advanced solid tumor | ORR: 3%; DCR: 57.6%; PFS: 3.4 months. Reduction of pAKT, pS6 and p4EBP1 in skin biopsy. RP2D: 350 mg/daily | F | C | 2011 | * | 2020 | Novartis | * | NCT01387321 | 86 |
| 1 | Combination of alpelisib with trastuzumab-emtansine | HER2+ metastatic breast cancer after trastuzumab and taxane therapy | PFS: 6 months; Median follow-up: 11.6 months. MTD: 250 mg/daily | F | C | 2014 | 2020 | 2020 | Northwestern University | Novartis, and National Cancer Institute (NCI) | NCT02038010 | 87 |
| 1 | Triple combination of alpelisib with trastuzumab and LJM716 | PIK3CA-mutated HER2+ metastatic breast cancer | PR: 5.8%, and SD: 82.3%. MTD: 350 mg (4 days on and 3 days off). mRNA profiling of pre- and on-treatment tissue demonstrated PIK3CA target engagement via induction of downstream signaling and feedback pathways | A | C | 2014 | * | 2021 | Memorial Sloan Kettering Cancer Center | Novartis | NCT02167854 | 88 |
| 1b | Combination of alpelisib with fulvestrant | Advanced ER+ breast cancer | PFS: 5.4 months. PFS with alpelisib (300 mg to 400 mg/once daily) + fulvestrant was prolonged in PIK3CA-mutated cancer (9.1 months) vs wild type cancer (4.7 months). ORR: 29% in the PIK3CA-mutated cancer vs no objective cancer responses in wild-type cancer. MTD: 400 mg/once daily. RP2D: 300 mg/once daily | A | C | 2010 | * | 2020 | Novartis | * | NCT01219699 | 89 |
| 1b | Combination of alpelisib with letrozole | ER+, HER2- metastatic breast cancer resistant to endocrine therapy | Anticancer activity: 44% on PIK3CA-mutated cancer, and 20% in PIK3CA wild-type cancer. PR or SD lasting ≥6 months in 80% of PIK3CA (H1047R)-mutated cancers, and in 28% of PIK3CA exon 9-mutated cancer | F | A, NR | 2013 | * | 2022 | Vanderbilt-Ingram Cancer Center | National Cancer Institute (NCI) | NCT01791478 | 90 |
| 1b | Combination of alpelisib with olaparib | Advanced triple-negative breast cancer | ORR: 18%, and DOR: 7.4 months | F | C | 2012 | * | 2021 | Dana-Farber Cancer Institute | Novartis, and AstraZeneca | NCT01623349 | 91 |
| 1b | Combination of alpelisib with olaparib | Epithelial ovarian cancer | PR: 36%; SD: 50%. MTD and RP2D: 200 mg/once daily | F | C | 2012 | * | 2021 | Dana-Farber Cancer Institute | Novartis, and AstraZeneca | NCT01623349 | 92 |
| 1b | Combination of alpelisib with imatinib | Advanced gastrointestinal stromal tumor | PR: 2.9%, and SD: 42.9%. PFS: 2 months. MTD: 350 mg/once daily for alpelisib when used in combination with 400 mg/once daily imatinib | A | C | 2012 | * | 2020 | Novartis Pharmaceuticals | * | NCT01735968 | 93 |
| 1b | Combination of alpelisib with BGJ398 | Advanced solid tumor with PIK3CA mutation | PR: 12.9%. MTD: 300 mg/day | F | C | 2013 | * | 2020 | Novartis | * | NCT01928459 | 94 |
| 1b | Combination of alpelisib with binimetinib | Advanced solid tumor | SD: 31% lasting over 6 weeks. PR: 75% in KRAS-mutated ovarian cancer. MTD: 200 mg/once daily. | F | C | 2011 | * | 2017 | Array BioPharma | * | NCT01449058 | 95 |
| 1b | Combination of alpelisib with everolimus ± exemestane | Advanced solid tumor | 16-week PFS rate: 52.4% (renal cell carcinoma) (triplet). MTD: 200 mg/daily (triplet) | A | C | 2014 | * | 2020 | Novartis | * | NCT02077933 | 96 |
| 1b | Combination of alpelisib with cetuximab plus intensity modulated radiation therapy (IMRT) | Stage III-IVB head and neck squamous cell carcinoma | CR: 100%, with 90.9% remaining disease-free. RP2D: 250 mg/daily | F | C | 2014 | * | 2021 | Memorial Sloan Kettering Cancer Center | Novartis | NCT02282371 | 97 |
| 1b | Triple combination of alpelisib with cetuximab and encorafenib | Metastatic BRAF-mutant colorectal cancer | ORR: 18% | F | C | 2012 | 2021 | 2021 | Pfizer | * | NCT01719380 | 98 |
| 1, 2 | Combination of alpelisib with nab-paclitaxel | HER2- metastatic breast cancer | ORR: 59% (CR: 7% and PR: 52%), 21% of whom had response lasting >12 months. PFS: 8.7 months. Patients with tumor/ctDNA mutation demonstrated better PFS (11.9 months) compared to those without mutation (7.5 months). Patients with normal metabolic status had longer PFS (12 months) compared to diabetic patients (7.5 months). RP2D: alpelisib 350 mg daily. | F | C | 2015 | 2021 | 2022 | University of Kansas Medical Center | Novartis | NCT02379247 | 99 |
| 1, 2 | Combination of alpelisib with letrozole or exemestane | PIK3CA-mutated, HR+ metastatic breast cancer | ORR: 19.3%, PR: 16.1%, and CR: 3.2%. CBR: 52% | F | C | 2013 | * | 2022 | Memorial Sloan Kettering Cancer Center | Novartis | NCT01870505 | 100 |
| 1b, 2 | Combination of alpelisib with letrozole | Advanced ER+ breast cancer | SD: 40% | A | A, NR | 2013 | * | 2022 | Novartis | * | NCT01872260 | 101 |
| 2 | Alpelisib | PI3K-altered, pretreated advanced ER+ breast cancer | ORR: 30%, and CBR: 36%. A reduction of PI3K pathway mutant circulating tumor DNA (ctDNA) levels (from baseline to week 8 while on therapy) was significantly associated with CBR, PR, and improved PFS | F | C | 2015 | * | 2022 | Peter MacCallum Cancer Centre, Australia | Novartis | NCT02506556 | 102 |
| 2 | Combination of alpelisib with fulvestrant | HR+, HER2-, PIK3CA-mutated breast cancer | PFS: 7.3 versus with alpelisib with fulvestrant vs PFS: 3.7 months in the real-world standard treatment cohort. 6-month PFS: 54.6% with alpelisib with fulvestrant vs 6-month PFS: 40.1% in the real-world standard treatment cohort | F | A, NR | 2017 | * | 2022 | Novartis | * | NCT03056755 | 103 |
| 2 | Combination of alpelisib with letrozole | HR+, HER2- breast cancer | Higher reduction in phosphorylated AKT in PIK3CA-mutant cancer with alpelisib + letrozole vs placebo + letrozole | A | C | 2013 | 2018 | 2018 | Novartis | * | NCT01923168 | 104 |
| 2 | Triple combination of alpelisib with cetuximab and encorafenib | Advanced BRAF-mutant colorectal cancer | PFS: 5.4 months with alpelisib + cetuximab + encorafenib vs PFD: 4.2 months with cetuximab + encorafenib. ORR: 22%, and OS: 15.2 month with the triple combination. ORR: 27%, and OS: none with cetuximab + encorafenib | A | C | 2012 | 2021 | 2021 | Pfizer | * | NCT01719380 | 105 |
| 3 | Combination of alpelisib with fulvestrant | HR+, HER2- advanced breast cancer previously treated with endocrine therapy | ORR: 26.6% with alpelisib + fulvestrant vs ORR: 12.8% with fulvestrant singularly. PFS at a median follow-up of 20 months: 11 months with alpelisib + fulvestrant vs 5.7 months with fulvestrant singularly in PIK3CA-mutated cancer | F | A, NR | 2015 | 2019 | 2022 | Novartis | * | NCT02437318 | 106 |
| 3 | Combination of alpelisib with fulvestrant | PIK3CA-mutated, HR+, HER2- advanced breast cancer that progressed on or after aromatase Inhibitor treatment | OS: 39.3 months with alpelisib + fulvestrant vs OS: 31.4 months with placebo + fulvestrant | F | A, NR | 2015 | 2019 | 2021 | Novartis | * | NCT02437318 | 107 |
| **Serabelisib** (TAK-117, MLN1117, INK1117) [PI3Kα Inhibitor] | | | | | | | | | | | | |
| **Phase** | **Treatment** | **Disease** | **Outcome** | **ISP** | **Status** | **FP** | **RFP** | **LUP** | **Sponsor** | **Collaborator** | **NCT Identifier** | **R** |
| 1 | Serabelisib | Advanced solid tumor | MTD: 150 mg for daily dosing schedule, and 900 mg for intermittent dosing schedules. Higher off-target toxicity with daily dosing schedule (21%) vs intermittent dosing schedules (10%). Lower on-target toxicity with daily dosing schedule (0%) vs intermittent dosing schedules (11%) | A | C | 2011 | 2017 | 2017 | Millennium Pharmaceuticals Inc | * | NCT01449370 | 113 |
| 1b | Triple combination of serabelisib with sapanisertib and paclitaxel | Advanced solid tumor | ORR: 47%, CBR: 73%, and PFS: 11 months. RP2D: 200 mg on days 2-4, 9-11, 16-18 and 23-25 | F | A, NR | 2017 | * | 2021 | Avera McKennan Hospital & University Health Center | * | NCT03154294 | 114 |
| **Taselisib** (GDC-0032, RG7604) [PI3Kα Inhibitor] | | | | | | | | | | | | |
| **Phase** | **Treatment** | **Disease** | **Outcome** | **ISP** | **Status** | **FP** | **RFP** | **LUP** | **Sponsor** | **Collaborator** | **NCT Identifier** | **R** |
| 1 | Taselisib | PIK3CA-mutated cancer | ORR: 9% | A | C | 2011 | * | 2021 | Genentech, Inc | * | NCT01296555 | 115 |
| 1b | Combination of taselisib with trastuzumab emtansine | Advanced HER2+ breast cancer | PFS: 7.6 months; CR: 4%; PR: 29%; SD: 50% | A | A, NR | 2015 | * | 2022 | Dana-Farber Cancer Institute | Genentech Inc | NCT02390427 | 116 |
| 1b | Combination of taselisib with tamoxifen | Wild type and PIK3CA-mutated HR+ metastatic breast cancer | ORR: 24%; median time to disease progression: 3.7 months; disease control for at least 6 months: 40%. RP2D: 4 mg/once daily in a continuous schedule | F | T | 2014 | * | 2022 | The Netherlands Cancer Institute | Genentech Inc,  EurocanPlatform, and  RATHER | NCT02285179 | 117 |
| 1b | Combination of taselisib with palbociclib | Advanced solid tumor | Reduction of pAKT, pGSK3β, and pRb in platelet rich plasma. R2PD: 2 mg/once daily | F | U | 2015 | * | 2019 | Royal Marsden NHS Foundation Trust | Institute of Cancer Research United Kingdom,  Roche Pharma AG, and Pfizer | NCT02389842 | 118 |
| 1b | Triple combination of taselisib with palbociclib and fulvestrant | Heavily pretreated PIK3CA-mutant, ER+, HER2- advanced breast cancer | ORR: 37.5% | F | U | 2015 | * | 2019 | Royal Marsden NHS Foundation Trust | Institute of Cancer Research UK,  Roche Pharma AG, and Pfizer | NCT02389842 | 119 |
| 2 | Taselisib | PIK3CA-mutated solid tumor, except breast cancer, squamous lung cancer | 6-month PFS: 19.9%, and median PFS: 3.1 months. 6-month OS: 60.7%, and median OS: 7.2 months | F | A, R | 2015 | * | 2022 | National Cancer Institute (NCI) | * | NCT02465060 | 120 |
| 2 | Combination of taselisib with letrozole | HR+, HER2- early stage breast cancer | In PIK3CA wild type cancer (ORR: 50% with taselisib + letrozole vs ORR: 39% with letrozole + placebo). In PIK3CA-mutated cancer (ORR: 56% with the combination vs ORR: 38% with letrozole + placebo) | A | C | 2014 | 2018 | 2018 | Genentech, Inc | SOLTI Breast Cancer Research Group,  Breast International Group, and Austrian Breast and Colorectal Cancer Group | NCT02273973 | 121 |
| 2 | Combination of taselisib with fulvestrant | HR+, HER2- advanced breast cancer | ORR: 38.5%, and CBR: 38.5% (tumor with PIK3CA-mutations). ORR: 14.3%, and CBR: 23.8% (tumor without PIK3CA mutations). ORR: 20%, and CBR: 30% (tumor with unknown PIK3CA-mutation status) | A | C | 2011 | * | 2021 | Genentech, Inc | * | NCT01296555 | 122 |
| 3 | Combination of taselisib with fulvestrant | ER+, PIK3CA-mutant, HER2-, advanced breast cancer | PFS: 7.4 months with taselisib + fulvestrant vs PFS: 5.4 months with placebo + fulvestrant (investigator assessed). ORR: 28%, CBR: 51.5%, and DOR: 8.7% with taselisib + fulvestrant vs ORR: 11.9%, CBR: 37.3%, and DOR: 7.2% with placebo + fulvestrant | A | T | 2015 | 2019 | 2022 | Hoffmann-La Roche | * | NCT02340221 | 123 |
| **Acalisib** (CAL-120, GS-9820) [PI3Kβ/δ Inhibitor] | | | | | | | | | | | | |
| **Phase** | **Treatment** | **Disease** | **Outcome** | **ISP** | **Status** | **FP** | **RFP** | **LUP** | **Sponsor** | **Collaborator** | **NCT Identifier** | **R** |
| 1b | Acalisib | Relapsed or refractory lymphoid malignancies | ORR: 42.1%, and PFS: 8.2 months. ORR: 53.3%, and PFS: 16.6 (chronic lymphocytic leukemia). ORR: 28.6%, and PFS: 4.0 months (non-Hodgkin’s lymphoma and Hodgkin’s lymphoma) | A | C | 2012 | * | 2016 | Gilead Sciences | * | NCT01705847 | 124 |
| **AZD8186** (1627494-13-6) [PI3Kβ Inhibitor] | | | | | | | | | | | | |
| **Phase** | **Treatment** | **Disease** | **Outcome** | **ISP** | **Status** | **FP** | **RFP** | **LUP** | **Sponsor** | **Collaborator** | **NCT Identifier** | **R** |
| 1 | AZD8186 | Advanced Solid Tumor | PR: 2.9%, and SD: 38.2%. RP2D: 60 mg/twice daily (5 days on and 2 days off). However, doses of 120 mg/twice daily (continuous, and 5 days on and 2 days off) were also considered tolerable | A | C | 2013 | * | 2020 | AstraZeneca | * | NCT01884285 | 128 |
| 1 | Combination of AZD8186 with abiraterone acetate | Metastatic castrate-resistant prostate cancer | PR: 1.9%; SD: 19.2%. 17.3% of patients showed a >30% decrease in prostate-specific antigen (PSA) | A | C | 2013 | * | 2020 | AstraZeneca | * | NCT01884285 | 129 |
| **GSK2636771** (GSK-2636771) [PI3Kβ Inhibitor] | | | | | | | | | | | | |
| **Phase** | **Treatment** | **Disease** | **Outcome** | **ISP** | **Status** | **FP** | **RFP** | **LUP** | **Sponsor** | **Collaborator** | **NCT Identifier** | **R** |
| 1 | Combination of GSK2636771 with enzalutamide | PTEN-deficient metastatic castration-resistant prostate cancer | 12-week non-progressive disease rate: 50%; radiographic PR lasting 36 weeks: 3%; prostate-specific antigen (PSA) reduction of ≥50%: 12% | A | C | 2014 | 2020 | 2020 | GlaxoSmithKline | * | NCT02215096 | 132 |
| 1, 2 | GSK2636771 | Advanced solid tumor with PTEN deficiency | SD: 24.5%; PR: 1.8%; reduction of pAKT: 33.9%. MTD and RP2D: 400 mg/once daily | A | C | 2011 | * | 2018 | GlaxoSmithKline | * | NCT01458067 | 133 |
| 1, 2 | Combination of GSK2636771 with pembrolizumab | Metastatic castration-resistant prostate cancer with PTEN deficiency | PR: 18.1%. PFS (> 12 months): 18.8 months. Prostate-specific antigen (PSA) > 50% reduction as compared to baseline. RP2D: 200 mg/once daily | A | C | 2011 | * | 2018 | GlaxoSmithKline | * | NCT01458067 | 134 |
| 1b, 2 | Combination of GSK2636771 with paclitaxel | Advanced gastric cancer with alterations in PI3K pathway | PFS: 12.1 weeks, OS: 33.4 weeks, ORR: 17.9%, and DCR: 67.9%. RP2D: 200 mg/once daily | A | C | 2015 | * | 2021 | Yonsei University | * | NCT02615730 | 135 |
| **Duvelisib** (IPI-145, INK-1197) [PI3Kγ/δ Inhibitor] | | | | | | | | | | | | |
| **Phase** | **Treatment** | **Disease** | **Outcome** | **ISP** | **Status** | **FP** | **RFP** | **LUP** | **Sponsor** | **Collaborator** | **NCT Identifier** | **R** |
| 1 | Duvelisib | Relapsed peripheral T-cell lymphoma, and cutaneous T-cell lymphoma | ORR: 50% (relapsed peripheral T-cell lymphoma). ORR: 31.6% (cutaneous T-cell lymphoma) | A | T | 2011 | * | 2021 | SecuraBio | * | NCT01476657 | 141 |
| 1 | Duvelisib | Relapsed or refractory lymphoma | ORR: 71.4%; CR: 14.2%; PR: 85.8% | F | C | 2015 | * | 2017 | AbbVie | Infinity Pharmaceuticals Inc | NCT02598570 | 142 |
| 1 | Duvelisib | Advanced hematologic malignancies | ORR: 58% (indolent non-Hodgkin lymphoma). ORR: 56% (relapsed or refractory chronic lymphocytic leukemia). ORR: 50% (peripheral T-cell lymphoma). ORR: 32% (cutaneous T-cell lymphoma) | A | T | 2011 | * | 2021 | SecuraBio | * | NCT01476657 | 143 |
| 1 | Triple combination of duvelisib with rituximab and bendamustine | Non-Hodgkin lymphoma and chronic lymphocytic leukemia | ORR: 71.8%; PFS: 13.7 months; 30-month OS probability: 62% | A | C | 2013 | * | 2016 | SCRI Development Innovations, LLC | Infinity Pharmaceuticals Inc | NCT01871675 | 144 |
| 1b, 2 | Tetra combination of duvelisib with fludarabine, cyclophosphamide, and rituximab | Chronic lymphocytic leukemia | 3-year PFS: 73%; 3-year OS: 93%; ORR by intention-to-treat analysis: 88% | A | A, NR | 2014 | 2018 | 2022 | Dana-Farber Cancer Institute | Verastem, Inc | NCT02158091 | 145 |
| 2 | Duvelisib | Relapsed or refractory follicular lymphoma | ORR: 83% (independent review committees assessment), CR: 26%, and PR: 56.5%. ORR: 78% (investigator assessment), and PR: 78.2% | A | U | 2021 | * | 2021 | CSPC ZhongQi Pharmaceutical Technology Co., Ltd | * | NCT04707079 | 146 |
| 2 | Duvelisib | Heavily pretreated, double-refractory indolent non-Hodgkin lymphoma | ORR: 47.3%; PFS: 9.5 months; DOR: 10 months; OS: 28.9 months; median time to response: 1.87 months | A | A, NR | 2013 | 2018 | 2021 | SecuraBio | * | NCT01882803 | 147 |
| 3 | Duvelisib | Relapsed chronic lymphocytic leukemia, and relapsed small lymphocytic lymphoma | PFS: 13.3 months, ORR: 74%, PR: 72.5%, and CR: 0.6% with duvelisib. PFS: 9.9 months, ORR: 45%, PR: 44.7%, and CR: 0.6% with ofatumumab | F | C | 2013 | 2019 | 2022 | SecuraBio | * | NCT02004522 | 148 |
| 3 | Duvelisib | Relapsed or refractory chronic lymphocytic leukemia and small lymphocytic lymphoma that progressed on ofatumumab | ORR: 77%, DOR: 14.9 months, and PFS: 15.7 months with duvelisib (after the crossover) vs ORR: 29%, DOR: 10.4 months, and PFS: 9.4 months with ofatumumab (prior to the crossover) | A | C | 2014 | * | 2021 | SecuraBio | * | NCT02049515 | 149 |
| **Eganelisib** (IPI-549) [PI3Kγ Inhibitor] | | | | | | | | | | | | |
| **Phase** | **Treatment** | **Disease** | **Outcome** | **ISP** | **Status** | **FP** | **RFP** | **LUP** | **Sponsor** | **Collaborator** | **NCT Identifier** | **R** |
| 1 | Combination of eganelisib with nivolumab | Advanced solid tumor | PR: 6.4% after a 8-week assessment. RP2D: 40 mg/once daily | F | A, NR | 2015 | * | 2022 | Infinity Pharmaceuticals Inc | * | NCT02637531 | 153 |
| 2 | Combination of eganelisib with nivolumab | Advanced urothelial carcinoma | ORR: 30.3% with eganelisib + nivolumab vs 25% with placebo + nivolumab. PFS: 9.1 months with eganelisib + nivolumab vs 8 months with placebo + nivolumab | A | A, NR | 2019 | * | 2022 | Infinity Pharmaceuticals Inc | Bristol-Myers Squibb | NCT03980041 | 154 |
| **Tenalisib** (RP-6530) [PI3Kγ/δ Inhibitor] | | | | | | | | | | | | |
| **Phase** | **Treatment** | **Disease** | **Outcome** | **ISP** | **Status** | **FP** | **RFP** | **LUP** | **Sponsor** | **Collaborator** | **NCT Identifier** | **R** |
| 1, 1b | Tenalisib | Relapsed or refractory peripheral and cutaneous T-cell lymphoma | ORR: 45.7%; CR: 9%; PR: 37%; DOR: 4.9 months. MTD: 800 mg/twice daily | A | C | 2015 | 2020 | 2020 | Rhizen Pharmaceuticals SA | * | NCT02567656 | 156 |
| **Idelalisib** (GS-1101, CAL-101) [PI3Kδ Inhibitor] | | | | | | | | | | | | |
| **Phase** | **Treatment** | **Disease** | **Outcome** | **ISP** | **Status** | **FP** | **RFP** | **LUP** | **Sponsor** | **Collaborator** | **NCT Identifier** | **R** |
| 1 | Idelalisib | Relapsed or refractory chronic lymphocytic leukemia | ORR: 72%; PR: 39%. PFS for all patients was 15.8 months. PFS: 32 months; reduction of pAKT T308, and increase of chronic lymphocytic leukemia-derived cytokines. RP2D: 150 mg/twice daily | A | C | 2008 | * | 2012 | Gilead Sciences | * | NCT00710528 | 166 |
| 1 | Idelalisib | Relapsed or refractory mantle cell lymphoma | ORR: 40%; PR: 35%; CR: 5%; SD: 47.5%; DOR: 2.7 months; PFS: 3.7 months; 1-year PFS: 22% | A | C | 2008 | * | 2012 | Gilead Sciences | * | NCT00710528 | 167 |
| 1 | Idelalisib | Relapsed or refractory indolent non-Hodgkin lymphoma | ORR: 47%; disease regression 85%; DOR: 18.4 months; PFS: 7.6 months; PR: 39%; CR: 1.6%; major response: 6%; SD: 39%. 46.4% of patients were progression free 48 weeks after treatment | F | C | 2008 | * | 2012 | Gilead Sciences | * | NCT00710528 | 168 |
| 1 | Combination of idelalisib with rituximab | Relapsed or refractory chronic lymphocytic leukemia | ORR: 89.5%, and PR: 89.5%. PFS: 36.8 months, and DOR: 34.9 months | A | C | 2010 | 2021 | 2021 | Gilead Sciences | * | NCT01088048 | 169 |
| 1 | Combination of idelalisib with bendamustine | Relapsed or refractory chronic lymphocytic leukemia | ORR: 77.8%, CR: 5.6%, and PR: 72.2%. PFS: 18.5 months, and DOR: 16.7 months | A | C | 2010 | 2021 | 2021 | Gilead Sciences | * | NCT01088048 | 169 |
| 1 | Triple combination of idelalisib with rituximab and bendamustine | Relapsed or refractory chronic lymphocytic leukemia | ORR: 86.7%, CR: 13.3%, and PR: 73.3%. PFS: 23 months, and DOR: 21.2 months | A | C | 2010 | 2021 | 2021 | Gilead Sciences | * | NCT01088048 | 169 |
| 1b | Idelalisib | Relapsed or refractory follicular lymphoma and chronic lymphocytic leukemia | PR: 83.3%. The median duration of PR was 14.5 months | F | C | 2014 | 2021 | 2021 | Gilead Sciences | * | NCT02242045 | 170 |
| 1b | Combination of idelalisib with tirabrutinib | Previously treated chronic lymphocytic leukemia | ORR: 93% with idelalisib + tirabrutinib vs ORR: 83% with tirabrutinib singularly. This small study did not significantly establish a superior efficacy of the combination over tirabrutinib alone | F | A, NR | 2015 | * | 2022 | Gilead Sciences | * | NCT02457598 | 171 |
| 2 | Idelalisib | Relapsed or refractory classical Hodgkin lymphoma | ORR: 20%; time to response: 2 months; PFS: 2.3 months; DOR: 8.4 months | F | C | 2011 | 2015 | 2018 | Gilead Sciences | * | NCT01393106 | 172 |
| 2 | Idelalisib | Relapsed indolent non-Hodgkin lymphoma | ORR: 57%; CR: 6%; PR: 50%; minor response: 1% | A | C | 2011 | 2014 | 2019 | Gilead Sciences | * | NCT01282424 | 173 |
| 2 | Idelalisib | Relapsed or refractory diffuse large B-cell lymphoma | ORR: 14%; DOR: 15.5 months. PFS: 0.8 months, and OS: 3.5 months in the germinal centre B-cell (GCB) subtype group. PFS: 1.3 months, and OS: 5.3 months in the non-GCB subtype group | A | C | 2018 | * | 2021 | Nordic Lymphoma Group | * | NCT03576443 | 174 |
| 2 | Combination of idelalisib with rituximab | Chronic lymphocytic leukemia | ORR: 97%; CR: 19%; ORR: 100% in cancer with del(17p)/TP53 mutations | F | T | 2010 | 2017 | 2018 | Gilead Sciences | * | NCT01203930 | 175 |
| 2 | Combination of idelalisib with obinutuzumab | Relapsed or refractory Waldenström’s macroglobulinemia | ORR: 71.4%, major response rate: 65.3%, PFS: 25.4 months. | A | A, NR | 2016 | * | 2022 | French Innovative Leukemia Organisation | * | NCT02962401 | 176 |
| 3 | Combination of idelalisib with rituximab | Chronic lymphocytic leukemia | ORR: 81%, PFS at 24 weeks: 93%, and 12-month OS: 92% with idelalisib + rituximab vs ORR: 13%, PFS at 24 weeks: 46%, and 12-month OS: 80% with placebo + rituximab | A | C | 2012 | 2014 | 2019 | Gilead Sciences | * | NCT01539512 | 177 |
| 3 | Combination of idelalisib with rituximab | Relapsed chronic lymphocytic leukemia | OS: 40.6 months, ORR: 83.6%, and PFS: 19.4 months with idelalisib + rituximab vs OS: 34.6 months, ORR: 15.5%, and PFS: 6.5 months with rituximab singularly | F | C | 2012 | 2014 | 2019 | Gilead Sciences | * | NCT01539512 | 178 |
| 3 | Combination of idelalisib with rituximab | Relapsed or refractory chronic lymphocytic leukemia | PFS: 15.8 months, and estimated PFS (at 12 months): 68%. | A | A, NR | 2016 | * | 2022 | Acerta Pharma BV | * | NCT02970318 | 179 |
| 3 | Combination of idelalisib with ofatumumab | Relapsed chronic lymphocytic leukaemia | PFS: 16.3 months with idelalisib + ofatumumab vs PFS: 8 months with ofatumumab singularly | A | T | 2012 | 2017 | 2019 | Gilead Sciences | * | NCT01659021 | 180 |
| 3 | Triple combination of idelalisib with rituximab and bendamustine | Relapsed or refractory chronic lymphocytic leukemia | PFS: 20.8 months, and ORR: 58% with triple combination vs PFS: 11.1 months, and ORR: 23% with placebo + bendamustine + rituximab | A | C | 2012 | 2018 | 2020 | Gilead Sciences | * | NCT01569295 | 181 |
| N/A | Combination of idelalisib with rituximab | Chronic lymphocytic leukaemia | ORR: 88.2%; event-free survival: 20.3 months; 3-year OS: 56.1% | A | C | 2018 | * | 2019 | Gilead Sciences | * | NCT03582098 | 182 |
| **Linperlisib** (YY-20394, PI3Kδ-IN-2) [PI3Kδ Inhibitor] | | | | | | | | | | | | |
| **Phase** | **Treatment** | **Disease** | **Outcome** | **ISP** | **Status** | **FP** | **RFP** | **LUP** | **Sponsor** | **Collaborator** | **NCT Identifier** | **R** |
| 1 | Linperlisib | B-cell hematologic malignancies | ORR: 64.0%, CR: 20%, PR: 44%, SD: 8%, and DCR: 72% | A | U | 2018 | * | 2018 | Shanghai YingLi Pharmaceutical Co., Ltd | * | NCT03757000 | 183 |
| 1b | Linperlisib | Relapsed or refractory peripheral T-cell lymphoma | ORR: 70.4%, CR: 25.9%, and PR: 44.4%. DCR: 100% | A | A, R | 2019 | * | 2021 | Shanghai YingLi Pharmaceutical Co., Ltd | * | NCT04108325 | 184 |
| **Parsaclisib** (INCB050465, IBI-376) [PI3Kδ Inhibitor] | | | | | | | | | | | | |
| **Phase** | **Treatment** | **Disease** | **Outcome** | **ISP** | **Status** | **FP** | **RFP** | **LUP** | **Sponsor** | **Collaborator** | **NCT Identifier** | **R** |
| 1 | Combination of parsaclisib with itacitinib | Advanced solid tumor | ORR: 7.1%, and SD: 26.2%. DOR: 6.3 months, and PFS: 2.1 months | A | T | 2015 | * | 2019 | Incyte Corporation | * | NCT02559492 | 189 |
| 1b | Parsaclisib | Relapsed or refractory B-cell lymphoma | ORR: 100% in follicular lymphoma and marginal zone lymphoma. ORR: 16.7% in diffuse large B-cell lymphoma | A | A, NR | 2017 | * | 2022 | Incyte Corporation | * | NCT03314922 | 190 |
| 1, 2 | Parsaclisib | Relapsed or refractory B-cell non-Hodgkin lymphoma | ORR: 78% (marginal zone lymphoma). ORR: 71% (follicular lymphoma). ORR: 67% (mantle cell lymphoma). ORR: 30% (diffuse large B-cell lymphoma) | A | C | 2013 | 2022 | 2022 | Incyte Corporation | * | NCT02018861 | 191 |
| 2 | Parsaclisib | Relapsed or refractory diffuse large B-cell lymphoma | ORR: 25.5% (BTK inhibitor naïve cancer). ORR: 20% (BTK inhibitor-pretreated cancer) | A | C | 2016 | 2020 | 2021 | Incyte Corporation | * | NCT02998476 | 192 |
| **Umbralisib** (TGR-1202, RP5264) [PI3Kδ Inhibitor] | | | | | | | | | | | | |
| **Phase** | **Treatment** | **Disease** | **Outcome** | **ISP** | **Status** | **FP** | **RFP** | **LUP** | **Sponsor** | **Collaborator** | **NCT Identifier** | **R** |
| 1 | Umbralisib | Relapsed or refractory hematologic malignancies | ORR: 37%; PR: 33%. ORR: 85%, and DOR: 13.4 months (chronic lymphocytic leukemia). ORR: 53%, and DOR: 9.3 months (follicular lymphoma). ORR: 31%, and DOR: 6.4 months (diffuse large B-cell lymphoma). MTD: 1200 mg/daily. RP2D: 800 mg/daily | F | C | 2013 | * | 2021 | TG Therapeutics Inc | SCRI Development Innovations, LLC | NCT01767766 | 196 |
| 1 | Triple combination of umbralisib with ublituximab and ibrutinib | Advanced non-Hodgkin lymphoma and chronic lymphocytic leukaemia | ORR: 84%; PFS: 38.2 months | A | C | 2013 | * | 2019 | TG Therapeutics Inc | * | NCT02006485 | 197 |
| 1, 1b | Combination of umbralisib with ibrutinib | Relapsed or refractory chronic lymphocytic leukaemia and relapsed or refractory mantle cell lymphoma | ORR: 90% (chronic lymphocytic leukaemia). ORR: 67% (mantle cell lymphoma). RP2D: 800 mg/daily | F | A, NR | 2014 | 2020 | 2022 | Dana-Farber Cancer Institute | TG Therapeutics Inc,  The Leukemia and Lymphoma Society, and  Blood Cancer Research Partnership | NCT02268851 | 198 |
| 1, 1b | Combination of umbralisib with ublituximab | Relapsed or refractory B-cell non-Hodgkin lymphoma and chronic lymphocytic leukemia | ORR: 46%; CR: 17%; DOR: 20 months | F | C | 2013 | * | 2019 | TG Therapeutics Inc | * | NCT02006485 | 199 |
| 2 | Umbralisib | Chronic lymphocytic leukemia intolerant to PI3Kδ or BTK inhibitors | PFS: 23.5 months | A | C | 2016 | * | 2022 | TG Therapeutics Inc | * | NCT02742090 | 200 |
| 2 | Combination of umbralisib with ublituximab | Relapsed or refractory marginal zone lymphoma | ORR: 68%, and CR: 19%. There was a 90%-reduction in disease burden from baseline | A | T | 2016 | * | 2022 | TG Therapeutics Inc | * | NCT02793583 | 201 |
| 2b | Umbralisib | Heavily pretreated relapsed or refractory indolent non-Hodgkin lymphoma | ORR: 47.1%, with cancer reduction present in 86.4% of patients; median time to response: from 2.7 months to 4.6 months. DOR: 18.3 months, and PFS: 20.9 months (small lymphocytic lymphoma). DOR: 11.1 months, and PFS: 10.6 months (follicular lymphoma). DOR: none, and PFS: none (marginal zone lymphoma). | A | T | 2016 | * | 2022 | TG Therapeutics Inc | * | NCT02793583 | 202 |
| **DUAL-PI3K/mTOR INHIBITORS** | | | | | | | | | | | | |
| **Apitolisib** (GDC-0980, RG7422, GNE 390) | | | | | | | | | | | | |
| **Phase** | **Treatment** | **Disease** | **Outcome** | **ISP** | **Status** | **FP** | **RFP** | **LUP** | **Sponsor** | **Collaborator** | **NCT Identifier** | **R** |
| 1 | Apitolisib | Advanced solid tumor | In the dose-escalation stage, PR: 7.1%, CR: 3.5%, and SD: 77%. MTD: 50 mg/once daily on a 21 days/28 days dosing schedule. In the dose-expansion stage, PR: 7.8%, and SD with cancer regression: 31.2%. R2PD: 40 mg/once daily on a 28 days/28 days dosing schedule | A | C | 2009 | * | 2016 | Genentech, Inc | * | NCT00854152 | 205 |
| 2 | Apitolisib | Metastatic renal cell carcinoma | ORR: 7.1%, PFS: 3.7 months, and OS: 16.5 months with apitolisib vs ORR: 11.6%, PFS: 6.1 months, and OS: 22.8 months with everolimus | A | C | 2011 | * | 2016 | Genentech, Inc | * | NCT01442090 | 206 |
| **Bimiralisib** (PQR309) | | | | | | | | | | | | |
| **Phase** | **Treatment** | **Disease** | **Outcome** | **ISP** | **Status** | **FP** | **RFP** | **LUP** | **Sponsor** | **Collaborator** | **NCT Identifier** | **R** |
| 1 | Bimiralisib | Advanced solid tumor | Inhibition of several PAM-associated phosphoproteins. MTD and RP2D: 80 mg/once daily in tumors with activating PIK3CA mutations | A | C | 2013 | * | 2017 | PIQUR Therapeutics AG | * | NCT01940133 | 215 |
| 2 | Bimiralisib | Relapsed or refractory lymphoma | ORR: 14%, CR: 4%, PR: 10% | A | C | 2017 | * | 2019 | PIQUR Therapeutics AG | University Hospital Basel (Switzerland),  and University Hospital Munich (Germany) | NCT03127020 | 216 |
| **Dactolisib** (BEZ-235, NVP-BEZ235) | | | | | | | | | | | | |
| **Phase** | **Treatment** | **Disease** | **Outcome** | **ISP** | **Status** | **FP** | **RFP** | **LUP** | **Sponsor** | **Collaborator** | **NCT Identifier** | **R** |
| 1 | Dactolisib | Advanced solid tumor | SD: 45.4%. MTD and R2PD: 300 mg/twice daily | A | C | 2011 | * | 2015 | SCRI Development Innovations, LLC | Novartis | NCT01343498 | 236 |
| 1 | Dactolisib | Advanced solid tumor | SD: 51.9%; reduced phosphorylation of ribosomal protein S6 (pS6). R2PD: 1000 mg/once daily | A | C | 2010 | * | 2020 | Novartis | * | NCT01195376 | 237 |
| **DS-7423** (DS7423) | | | | | | | | | | | | |
| **Phase** | **Treatment** | **Disease** | **Outcome** | **ISP** | **Status** | **FP** | **RFP** | **LUP** | **Sponsor** | **Collaborator** | **NCT Identifier** | **R** |
| 1 | DS-7423 | Advanced solid tumor | SD: 18.5% in Caucasian patients, and 20% in Asian patients. MTD and RP2D: 240 mg/daily for both Caucasian patients and Asian patients | A | C | 2011 | * | 2014 | Daiichi Sankyo, Inc | * | NCT01364844 | 241 |
| **Gedatolisib** (PF-05212384, PKI-587) | | | | | | | | | | | | |
| **Phase** | **Treatment** | **Disease** | **Outcome** | **ISP** | **Status** | **FP** | **RFP** | **LUP** | **Sponsor** | **Collaborator** | **NCT Identifier** | **R** |
| 1 | Gedatolisib | Heavily pretreated solid tumor | ORR: 2.6%; CBR: 13%; ≥ 6 month-enduring SD: 10.4%; reduction (10.6%) of pAKT Ser473. MTD and RP2D: 154 mg/once weekly | A | C | 2009 | 2018 | 2018 | Pfizer | * | NCT00940498 | 251 |
| 1 | Combination of gedatolisib with cofetuzumab pelidotin | Metastatic triple negative breast cancer | ORR: 16.7%, and SD: 16.7%. CBR at 18 months: 27.8%. PFS: 2 months | F | C | 2017 | * | 2021 | Indiana University | * | NCT03243331 | 252 |
| 1 | Combination of gedatolisib with irinotecan | Advanced solid tumor | ORR: 5%, CBR: 16%, and PFS: 2.8 months (colorectal cancer) | F | T | 2011 | 2018 | 2018 | Pfizer | * | NCT01347866 | 253 |
| 1 | Combination of gedatolisib with PD-0325901 | Advanced solid tumor | PR: 60%, and SD: 40% (ovarian cancer). PR: 100% (endometrial cancer) | F | T | 2011 | 2018 | 2018 | Pfizer | * | NCT01347866 | 253 |
| 1 | Triple combination of gedatolisib with paclitaxel and carboplatin | Advanced solid tumour | ORR: 65%. ORR: 80% (clear cell ovarian cancer). RP2D: 110 mg/m^2^ on days 1, 8, 15, and 22 | A | C | 2014 | * | 2019 | Oncology Institute of Southern Switzerland | * | NCT02069158 | 254 |
| 2 | Gedatolisib | Recurrent endometrial cancer | CBR: 53% in low stathmin-expressing tumor, and 26% in high stathmin-expressing tumor | A | T | 2011 | 2015 | 2019 | Pfizer | * | NCT01420081 | 255 |
| **Omipalisib** (GSK2126458, GSK458) | | | | | | | | | | | | |
| **Phase** | **Treatment** | **Disease** | **Outcome** | **ISP** | **Status** | **FP** | **RFP** | **LUP** | **Sponsor** | **Collaborator** | **NCT Identifier** | **R** |
| 1 | Omipalisib | Advanced solid tumor | Durable objective response. MTD: 2.5 mg/once daily | A | C | 2012 | * | 2019 | GlaxoSmithKline | * | NCT01725139 | 262 |
| **Paxalisib** (GDC-0084, RG7666) | | | | | | | | | | | | |
| **Phase** | **Treatment** | **Disease** | **Outcome** | **ISP** | **Status** | **FP** | **RFP** | **LUP** | **Sponsor** | **Collaborator** | **NCT Identifier** | **R** |
| 1 | Paxalisib | Progressive or recurrent high-grade glioma | SD: 40%. MTD: 45 mg/daily | A | C | 2012 | * | 2016 | Genentech, Inc | * | NCT01547546 | 269 |
| **Samotolisib** (LY3023414, GTPL8918) | | | | | | | | | | | | |
| **Phase** | **Treatment** | **Disease** | **Outcome** | **ISP** | **Status** | **FP** | **RFP** | **LUP** | **Sponsor** | **Collaborator** | **NCT Identifier** | **R** |
| 1 | Samotolisib | Advanced mesothelioma | PFS: 2.83 months; SD: 41%, ORR: 2.4%; DCR: 43%. | A | C | 2012 | * | 2022 | Eli Lilly and Company | * | NCT01655225 | 287 |
| 1 | Samotolisib | Advanced solud tumor | SD: 31.9%, and DCR: 34%. MTD: 325 mg/once daily or 200 mg/twice daily | A | C | 2012 | * | 2022 | Eli Lilly and Company | * | NCT01655225 | 288 |
| 1 | Samotolisib | Advanced solud tumor | DCR: 55.6% | F | C | 2015 | * | 2018 | Eli Lilly and Company | * | NCT02536586 | 289 |
| 1b | Combination of samotolisib with prexasertib | Advanced solud tumor | ORR: 15.4%; PR: 3.7% | A | C | 2014 | * | 2020 | Eli Lilly and Company | * | NCT02124148 | 290 |
| 1b, 2 | Combination of samotolisib with enzalutamide | Metastatic castration-resistant prostate cancer following tumor progression on abiraterone | PFS: 3.8 months with samotolisib + enzalutamide versus 2.8 months with placebo + enzalutamide. Radiographic PFS: 10.2 months with samotolisib + enzalutamide versus 5.5 months with placebo + enzalutamide | A | C | 2015 | 2021 | 2021 | Eli Lilly and Company | Sarah Cannon Development Innovations | NCT02407054 | 291 |
| 2 | Samotolisib | Pretreated-advanced endometrial cancer with mutations in the PAM pathway | ORR: 16%; CBR: 28%; PR: 16%; PFS: 2.5 months; OS: 9.2 months | A | C | 2015 | * | 2022 | Memorial Sloan Kettering Cancer Center | Eli Lilly and Company | NCT02549989 | 292 |
| **Voxtalisib** (XL765, SAR245409) | | | | | | | | | | | | |
| **Phase** | **Treatment** | **Disease** | **Outcome** | **ISP** | **Status** | **FP** | **RFP** | **LUP** | **Sponsor** | **Collaborator** | **NCT Identifier** | **R** |
| 1 | Voxtalisib | Recurrent glioma | Inhibition of PAM signaling pathway; reduction of pS6K1; decrease of Ki67 expression | A | C | 2010 | * | 2012 | Sanofi | * | NCT01240460 | 296 |
| 1 | Voxtalisib | Advanced solid tumor | SD: 48%; inhibition of PAM signaling pathway; reduction of pAKT(T308), pAKT(S473), and pEBP1 in paired cancer biopsises. MTD: 90 mg/once daily, and 50 mg/twice daily | A | C | 2007 | * | 2013 | Sanofi | * | NCT00485719 | 297 |
| 1 | Voxtalisib | Advanced solid tumor | SD: 29% in the once/daily conhort, and 50% in the twice/daily cohort. MTD: 60 mg/once daily, and 40 mg/twice daily | A | C | 2012 | * | 2020 | Sanofi | * | NCT01596270 | 298 |
| 1 | Combination of voxtalisib with erlotinib | Advanced solid tumor | SD: 37.5%; reduction of pAKT(T308), p4EBP1T70, pEGFRY1045, and pERKT202/Y204. RP2D: 90 mg/once daily, and 50 mg/twice daily. MTD: 70 mg/once daily, and 20 mg/twice daily | F | C | 2008 | * | 2012 | Sanofi | * | NCT00777699 | 299 |
| 1 | Combination of voxtalisib with temozolomide with/without radiotherapy | High-grade malignant glioma | SD: 68%; PR: 4%; suppression of PAM signaling pathway. MTD: 90 mg/once daily, and 40 mg/twice daily, with voxtalisib + temozolomide. MTD: not reached with voxtalisib + temozolomide + radiotherapy | F | C | 2008 | * | 2013 | Sanofi | * | NCT00704080 | 300 |
| 1b | Combination of voxtalisib with rituximab | Relapsed or refractory B-cell malignancies | PR: 33.3%, CR: 6.7%, SD: 46.7%, and PFS: 33.4 weeks. RP2D: 50 mg/twice daily | A | C | 2011 | * | 2016 | Sanofi | * | NCT01410513 | 301 |
| 1b | Triple combination of voxtalisib with rituximab and bendamustine | Relapsed or refractory B-cell malignancies | PR: 37.5%, CR: 12.5%, SD: 37.5%, and PFS: 33.4 weeks (indolent non-Hodgkin lymphoma or mantle cell lymphoma). PR: 41.7%, CR: 16.7%, SD: 33.3%, and PFS: 26.1 weeks (chronic lymphocytic leukaemia). RP2D: 50 mg/twice daily | A | C | 2011 | * | 2016 | Sanofi | * | NCT01410513 | 301 |
| 2 | Voxtalisib | Relapsed or refractory non-Hodgkin lymphoma or chronic lymphocytic leukaemia | ORR: 18.3%; CR: 5%; PR: 13.4%; SD: 33.5%; PFS: 14.4 weeks. ORR: 41.3% (follicular lymphoma); ORR: 11.9% (mantle cell lymphoma); ORR: 4.9% (diffuse large B-cell lymphoma); ORR: 11.4% (chronic lymphocytic leukaemia and small lymphocytic lymphoma) | A | C | 2011 | * | 2016 | Sanofi | * | NCT01403636 | 302 |
