# Supplementary material for: PI3K/AKT/mTOR signaling transduction pathway and targeted therapies in cancer
Source: Mol Cancer. 2023 Aug 18;22:138. doi: 10.1186/s12943-023-01827-6 (PMC10436543; doi:10.1186/s12943-023-01827-6)
Supplement: Supplementary file 6 — Additional file 6: Supplementary table 2. [file 12943_2023_1827_MOESM6_ESM.docx]

| **AKT INHIBITORS** | | | | | | | | | | | | |
| --- | --- | --- | --- | --- | --- | --- | --- | --- | --- | --- | --- | --- |
| **Afuresertib** (GSK2110183) | | | | | | | | | | | | |
| **Phase** | **Treatment** | **Disease** | **Outcome** | **ISP** | **Status** | **FP** | **RFP** | **LUP** | **Sponsor** | **Collaborator** | **NCT Identifier** | **R** |
| 1 | Afuresertib | Advanced haematological malignancy | PR: 9%, and MR: 9%. MTD: 125 mg/daily | F | C | 2009 | * | 2012 | Accenture | * | NCT00881946 | 310 |
| 1 | Combination of afuresertib with MEK inhibitor trametinib | Advanced solid tumour | ORR: 5%, and SD: 20%. MTD: 50 mg (days 1-10 every 28-day) with trametinib 1.5 mg/once daily | A | C | 2011 | * | 2017 | GlaxoSmithKline | * | NCT01476137 | 314 |
| 1b | Triple combination of afuresertib with paclitaxel plus carboplatin | Recurrent platinum-resistant ovarian cancer | ORR: 32% (RECIST 1.1 criteria) or 52% (GCIG CA125 criteria). PFS: 7.1 months. MTD: 125 mg/daily | A | C | 2012 | 2018 | 2018 | Accenture | * | NCT01653912 | 315 |
| 2 | Combination of afuresertib with ofatumumab | Previously-treated chronic lymphocytic leukemia | ORR: 50%, and CR: 3.6. PFS: 8.5 months, and OS: 34.8 months | F | C | 2012 | 2019 | 2019 | University Health Network, Toronto | Novartis | NCT01532700 | 316 |
| **Capivasertib** (AZD5363) | | | | | | | | | | | | |
| **Phase** | **Treatment** | **Disease** | **Outcome** | **ISP** | **Status** | **FP** | **RFP** | **LUP** | **Sponsor** | **Collaborator** | **NCT Identifier** | **R** |
| 1 | Capivasertib | ER+ invasive breast cancer | Reduction of AKT pathway key biomarkers pGSK3β and pPRAS40. Decrease of proliferation marker Ki67 | F | C | 2014 | * | 2017 | University of Nottingham | AstraZeneca,  Cancer Research UK, and National Cancer Research Network | NCT02077569 | 325 |
| 1 | Capivasertib | PIK3CA-mutant gynaecological cancer, and PIK3CA-mutant breast cancer | PR: 8% in PIK3CA-mutant gynaecological cancer, and PR: 4% in PIK3CA-mutant breast cancer. MTD: 320 mg for continuous schedule, 480 mg for 4/7 schedule, and 640 mg for 2/7 schedule | F | A, NR | 2010 | * | 2022 | AstraZeneca | * | NCT01226316 | 326 |
| 1 | Capivasertib | AKT1 E17K-mutated refractory tumor | ORR: 28.6%, CR: 2.8%, and PR: 25.7%. SD: 46%. PFS: 28.4 months. 6-month PFS rate: 50% | A | C | 2008 | 2020 | 2022 | Eastern Cooperative Oncology Group | National Cancer Institute (NCI) | NCT00700882 | 327 |
| 1 | Combination of capivasertib with fulvestrant | Previously-treated AKT1 E17K-mutant ER+ metastatic breast cancer | ORR: 36% with capivasertib + fulvestrant vs ORR: 20% with capivasertib. A ≥50% decrease in AKT1 E17K at cycle 2 day 1 was associated with improved PFS | A | A, NR | 2010 | * | 2022 | AstraZeneca | * | NCT01226316 | 328 |
| 1 | Combination of capivasertib with fulvestrant | PTEN-mutant ER+ metastatic breast cancer | 24-week CBR: 42% in fulvestrant-pretreated breast cancer vs 24-week CBR: 17% in untreated breast cancer. ORR: 21% in fulvestrant-pretreated breast cancer vs ORR: 8% in untreated breast cancer | A | A, NR | 2010 | * | 2022 | AstraZeneca | * | NCT01226316 | 329 |
| 1 | Combination of capivasertib with olaparib | BRCA1/BCRA2 wild-type cancer and BRCA1/BCRA2-mutated cancer | CBR: 44.6% in both types of cancer. RP2D: olaparib 300 mg twice/daily with either capivasertib 400 mg/twice daily (4 days on, 3 days off), or capivasertib 640 mg/twice daily (2 days on, 5 days off) | F | C | 2015 | * | 2020 | Royal Marsden NHS Foundation Trust | Institute of Cancer Research UK, and AstraZeneca | NCT02338622 | 330 |
| 1 | Combination of capivasertib with abiraterone acetate | Metastatic castration-resistant prostate cancer | Reduced (> 20%) PSA levels | A | C | 2019 | * | 2022 | AstraZeneca | Parexel | NCT04087174 | 331 |
| 1 | Combination of capivasertib with enzalutamide | Metastatic castration-resistant prostate cancer | 25% of patients met at least one response criteria (PSA decline ≥50%, radiological response, and CTC conversion) and completed the 12-week study. 8.3% of patients met at three response criteria and remained on treatment for 25 weeks. RP2D: 400 mg/twice daily. | A | U | 2015 | * | 2018 | Institute of Cancer Research UK | Royal Marsden NHS Foundation Trust | NCT02525068 | 332 |
| 1b | Combination of capivasertib with olaparib | Recurrent triple negative breast cancer, ovarian cancer, and endometrial cancer | PR: 19%. SD (> 4 months): 22%. RP2D: 400 mg/twice daily on a schedule of 4-day on and 3-day off. | F | A, NR | 2014 | * | 2022 | M.D. Anderson Cancer Center | National Cancer Institute (NCI) | NCT02208375 | 333 |
| 2 | Combination of capivasertib with fulvestrant | Metastatic ER+ breast cancer | PFS: 10.3 months with capivasertib + fulvestrant vs PFS: 4.8 months with placebo + fulvestrant | A | A, NR | 2013 | * | 2022 | Velindre NHS Trust | AstraZeneca,  Cenduit LLC,  Covance, and Cardiff and Vale University Health Board | NCT01992952 | 322 |
| 2 | Combination of capivasertib with fulvestrant | Aromatase inhibitor-resistant ER+, HER2- advanced breast cancer | PFS: 10.3 months with capivasertib + fulvestrant vs PFS: 4.8 months with placebo + fulvestrant. OS: 29.3 months with capivasertib + fulvestrant vs OS: 23.4 months with placebo + fulvestrant. In PI3K/AKT/PTEN pathway-altered cancer PFS was 12.8 months vs 4.6 months, and OS was 38.9 months vs 20 months | A | A, NR | 2013 | * | 2022 | Velindre NHS Trust | AstraZeneca,  Cenduit LLC,  Covance, and Cardiff and Vale University Health Board | NCT01992952 | 334 |
| 2 | Combination of capivasertib with paclitaxel | ER+ advanced or metastatic breast cancer | PFS: 10.9 months with capivasertib + paclitaxel vs PFS: 8.4 months with placebo + paclitaxel | A | A, NR | 2012 | 2019 | 2022 | AstraZeneca | * | NCT01625286 | 335 |
| 2 | Combination of capivasertib with paclitaxel | Metastatic triple-negative breast cancer | PFS: 5.9 months with capivasertib + paclitaxel vs PFS: 4.2 months with placebo + paclitaxel. OS: 19.1 months with capivasertib + paclitaxel vs OS: 12.6 months with placebo + paclitaxel. PFS: 9.3 months with capivasertib + paclitaxel vs PFS: 3.7 months with placebo + paclitaxel (PIK3CA/AKT1/PTEN-altered tumors) | A | U | 2015 | * | 2020 | Queen Mary University of London | AstraZeneca, and Cancer Research UK | NCT02423603 | 336 |
| 2 | Triple combination of capivasertib with docetaxel and prednisolone | Metastatic castration-resistant prostate cancer | OS: 31.1 months with capivasertib + docetaxel + prednisolone vs OS: 20.2 months with placebo + docetaxel + prednisolone. PFS: 7 months with capivasertib + docetaxel + prednisolone vs PFS: 6.7 months with placebo + docetaxel + prednisolone | A | C | 2014 | * | 2022 | University Hospital Southampton NHS Foundation Trust | AstraZeneca, and  Cancer Research UK | NCT02121639 | 337 |
| 3 | Combination of capivasertib with fulvestrant | HR+, HER2- breast cancer resistant to aromatase inhibitors and CDK4/CDK6 inhibitors | Combination of apivasertib + fulvestrant more than doubled PFS compared to placebo + fulvestrant | A | A, NR | 2020 | * | 2023 | * | AstraZeneca | NCT04305496 | 324 |
| **Ipatasertib** (GDC-0068) | | | | | | | | | | | | |
| **Phase** | **Treatment** | **Disease** | **Outcome** |  | **Status** | **FP** | **RFP** | **LUP** | **Sponsor** | **Collaborator** | **NCT Identifier** | **R** |
| 1 | Ipatasertib | Advanced solid tumour | Radiographic SD: 30%. Multiple targets (PRAS40, GSK3β, and mTOR) resulted inhibited in paired on-treatment biopsies | A | C | 2010 | * | 2016 | Genentech, Inc. | * | NCT01090960 | 345 |
| 1b | Combination of ipatasertib with chemotherapy | Advanced solid tumour | RP2D: 400 mg/daily (for days 1–21 of each 28-day cycle) in combination with paclitaxel. RP2D: 600 mg/daily in combination with mFOLFOX6 | F | C | 2011 | * | 2022 | Genentech, Inc. | * | NCT01362374 | 346 |
| 1b | Combination of ipatasertib with cobimetinib | Advanced or metastatic solid tumor | PR: 4.5%. RP2D: 300 mg/daily (for 21 days) in combination with intermittent cobimetinib (once-daily on days 1, 4, 8, 11, 15, and 18) | A | C | 2012 | 2016 | 2016 | Genentech, Inc. | * | NCT01562275 | 347 |
| 2 | Combination of ipatasertib with paclitaxel | Early-stage triple-negative breast cancer | Ipatasertib + paclitaxel determined a downregulation of AKT/mTORC1 signaling, especially among the tumors with PIK3CA/AKT1/PTEN alterations, or among the responders to the treatment | F | C | 2014 | 2018 | 2018 | Genentech, Inc. | SOLTI Breast Cancer Research Group | NCT02301988 | 348 |
| 2 | Combination of ipatasertib with paclitaxel | Advanced triple-negative breast cancer | PFS: 6.2 months with ipatasertib + paclitaxel vs PFS: 4.9 months with placebo + paclitaxel | A | C | 2014 | 2021 | 2021 | Genentech, Inc. | * | NCT02162719 | 349 |
| 2 | Combination of ipatasertib with paclitaxel | Advanced and metastatic triple-negative breast cancer | Median follow-up: 19 months with ipatasertib + paclitaxel vs median follow-up: 16 months with placebo + paclitaxel. OS: 83% with ipatasertib + paclitaxel vs OS: 68% with placebo + paclitaxel. OS was longer with the combination in the PTEN-low (23.1 vs 15.8 months) and PIK3CA/AKT1/PTEN-altered (25.8 vs 22.1 months) subgroups | A | C | 2014 | 2021 | 2021 | Genentech, Inc. | * | NCT02162719 | 350 |
| 2 | Combination of ipatasertib with abiraterone | Metatatic castration-resistant prostate cancer | Radiographic PFS was longer after ipatasertib + abiraterone vs placebo + abiraterone, especially in PTEN-loss cancers. | F | C | 2011 | * | 2022 | Genentech, Inc. | * | NCT01485861 | 351 |
| 3 | Combination of ipatasertib with paclitaxel | PIK3CA/AKT1/PTEN-altered HR+, HER2-, advanced breast cancer | PFS: 9.3 months with ipatasertib + paclitaxel vs PFS: 9.3 months with placebo + paclitaxel (investigator assessed). PFS: 9.2 months with ipatasertib + paclitaxel vs PFS: 8.5 months with placebo + paclitaxel (independent review committees assessed). ORR: 47% with ipatasertib + paclitaxel vs ORR: 47% with placebo + paclitaxel | A | A, NR | 2017 | * | 2022 | Hoffmann-La Roche | * | NCT03337724 | 352 |
| 3 | Triple combination of ipatasertib with abiraterone and prednisolone | Metastatic castration-resistant prostate cancer with PTEN-loss | Radiographic PFS: 18.5 months with ipatasertib + abiraterone vs radioghraphic PFS: 16.5 months with placebo + abiraterone. PFS: 19.2 months with ipatasertib + abiraterone vs PFS: 16.6 months with placebo + abiraterone | A | A, NR | 2017 | * | 2022 | Hoffmann-La Roche | * | NCT03072238 | 353 |
| **M2698** (MSC2363318A) | | | | | | | | | | | | |
| **Phase** | **Treatment** | **Disease** | **Outcome** | **ISP** | **Status** | **FP** | **RFP** | **LUP** | **Sponsor** | **Collaborator** | **NCT Identifier** | **R** |
| 1 | M2698 | Advanced cancer ± PAM pathway alterations | ORR: 40%, SD: 32%, and PFS: 2.4 months (all cancers). ORR: 41%, SD: 32%, and PFS: 2.8 months (PAM pathway-altered cancers). ORR: 46%, SD: 35%, and PFS: 2.8 months (PAM pathway-altered cancers without potential resistance markers such as EGFR, KRAS and AKT2) | F | C | 2013 | * | 2018 | EMD Serono | * | NCT01971515 | 357 |
| 1 | Combination of M2698 with trastuzumab or tamoxifen | Advanced breast cancer | SD at 12 weeks: 27.4% with monotherapy. PR: 7.6% with M2698 + trastuzumab cohort vs PR: 3.8% with M2698 + tamoxifen. PFS: 31 months with M2698 + trastuzumab vs PFS: 2.7 months with M2698 + tamoxifen. RP2D: 240 mg/once daily with monotherapy, 160 mg/once daily with M2698 + trastuzumab, and 160 mg/once daily-240 mg/once daily intermittent regimen with M2698 + tamoxifen. | F | C | 2013 | * | 2018 | EMD Serono | * | NCT01971515 | 354 |
| **MK‑2206** (1032349-93-1) | | | | | | | | | | | | |
| **Phase** | **Treatment** | **Disease** | **Outcome** | **ISF** | **Status** | **FP** | **RFP** | **LUP** | **Sponsor** | **Collaborator** | **NCT Identifier** | **R** |
| 1 | MK-2206 | Advanced solid tumor | 23.3% tumour shrinkage, and 60% reduced serum level of the tumour marker CA19.9 (pancreatic cancer with PTEN loss and KRAS G12D mutation) | F | C | 2008 | 2019 | 2019 | Merck Sharp & Dohme LLC | * | NCT00670488 | 366 |
| 1 | Combination of MK-2206 with ridaforolimus | Breast cancer | PR: 12.5% (investigator assessment). CR: 14.3% (central assessment). MTD: 90 mg/once weekly | F | C | 2011 | * | 2015 | Merck Sharp & Dohme LLC | * | NCT01295632 | 367 |
| 1 | Combination of MK-2206 with trastuzumab | Previously-treated HER2+ solid tumor | CR: 3.2% (breast cancer), PR: 3.2% (breast cancer), and SD (> 4 months): 16.1% (breast cancer). MTD: 60 mg for the every-other-day schedule, and 135 mg for the once-weekly schedule | F | T | 2009 | 2018 | 2018 | Merck Sharp & Dohme LLC | * | NCT00963547 | 368 |
| 1 | Combination of MK-2206 with anastrozole or fulvestrant | ER+ metastatic breast cancer | CBR: 42% and no progression within 6 months with both combinations. RPTD: 150 mg weekly with each combination | F | C | 2011 | * | 2018 | National Cancer Institute (NCI) | * | NCT01344031 | 361 |
| 1 | Combination of MK-2206 with dalotuzumab | Advanced solid tumour | SD: 13.3% | A | T | 2010 | 2017 | 2018 | Merck Sharp & Dohme LLC | * | NCT01243762 | 369 |
| 1 | Triple combination of MK-2206 with anastrozole and fulvestrant | ER+ metastatic breast cancer | CBR: 42% and no progression within 6 months. RPTD: 150 mg weekly | F | C | 2011 | * | 2018 | National Cancer Institute (NCI) | * | NCT01344031 | 361 |
| 1b | Triple combination of MK-2206 with paclitaxel and trastuzumab | Advanced HER2-overexpressing solid tumor malignancies | ORR: 62.5%, PR: 43.7%, and CR: 18.7%. DOR: 6 months | F | C | 2010 | 2013 | 2014 | University of California, San Francisco | Merck Sharp & Dohme LLC | NCT01235897 | 370 |
| 1, 2 | Triple combination of MK-2206 with bendamustine and rituximab | Relapsed or refractory chronic lymphocytic leukemia | ORR: 92%, and PFS: 16 months. MTD: 90 mg/once weekly | F | C | 2011 | 2016 | 2017 | National Cancer Institute (NCI) | * | NCT01369849 | 371 |
| 2 | MK-2205 | PIK3CA or AKT1 mutated-advanced breast cancer | ORR: 5.6%. MK-2206 treatment was associated with a significant decline in pAKT S473 and pAKT T308 in PBMC | A | C | 2011 | 2016 | 2018 | National Cancer Institute (NCI) | * | NCT01277757 | 372 |
| 2 | MK-2206 | Relapsed or refractory lymphoma | ORR: 14%, CR: 3.3%, and PR: 10.1%, with a median response duraction of 5.8 months. There was 49% reduction in cancer imaging measurements | F | C | 2010 | 2015 | 2020 | National Cancer Institute (NCI) | * | NCT01258998 | 373 |
| 2 | MK-2206 | Advanced gastric and gastroesophageal junction cancer | ORR: 1%, PFS: 1.8 months, and OS: 5.1 months | F | C | 2010 | 2014 | 2015 | National Cancer Institute (NCI) | * | NCT01260701 | 374 |
| 2 | MK-2206 | Uterine serous carcinoma | CBR: 14.3%, SD: 35.7%, PFS: 2 months, and OS: 6.4 months | F | C | 2011 | 2017 | 2022 | National Cancer Institute (NCI) | * | NCT01307631 | 375 |
| 2 | Combination of MK-2206 with erlotinib | Advanced non-small cell lung cancer (NSCLC) | ORR: 9%, SD (12 weeks): 40%, and PFS: 4.4 months (EGFR-mutated cancer). ORR: 3%, SD (12 weeks): 47%, and PFS: 4.6 months (EGFR wild type cancer) | A | C | 2011 | 2016 | 2016 | National Cancer Institute (NCI) | * | NCT01294306 | 376 |
| 2 | Combination of MK-2206 with standard neoadjuvant chemotherapy | HER2+ and/or HR- breast cancer | Pathologic CR: 48% vs pathologic CR: 29% in the control (HER2+ breast cancer). Pathologic CR: 46% in HR- cancer vs pathologic CR: 25.8% in the control (HR- cancer) | A | A, R | 2010 | * | 2022 | QuantumLeap Healthcare Collaborative | * | NCT01042379 | 377 |
| **Perifosine** (D-21266, KRX-0401, NSC 639966) | | | | | | | | | | | | |
| **Phase** | **Treatment** | **Disease** | **Outcome** | **ISP** | **Status** | **FP** | **RFP** | **LUP** | **Sponsor** | **Collaborator** | **NCT Identifier** | **R** |
| 1 | Perifosine | Neuroblastoma | Progression-free for 54 months: 33.3%. PR: 7.4%, and CR: 3.7% | F | C | 2008 | * | 2017 | Memorial Sloan Kettering Cancer Center | University of Wisconsin, Duke University, AEterna Zentaris | NCT00776867 | 382 |
| 1 | Combination of perifosine and temsirolimus | Recurrent glioblastoma | PR: 3.4%, SD: 44.8%, OS: 10.4 months, and PFS: 2.7 months MTD: 600 mg on day 1 (in 4 divided doses) followed by 100 mg/daily | A | C | 2010 | 2016 | 2021 | National Cancer Institute (NCI) | * | NCT01051557 | 383 |
| 1 | Triple combination of perifosine with lenalidomide and dexamethasone | Relapsed or refractory multiple myeloma | PR: 50%, OS: 30.6 months, and PFS: 10.8 months | F | C | 2006 | * | 2012 | AEterna Zentaris | * | NCT00415064 | 384 |
| 1, 2 | Combination of perifosine with bortezomib | Previously bortezomib-treated multiple myeloma | ORR: 41% (65% in a relapsed group and 32% in a refractory group). PFS: 6.4 months, and OS: 25 months | F | C | 2006 | * | 2018 | AEterna Zentaris | * | NCT00401011 | 385 |
| 2 | Combination of perifosine with capecitabine | Previously-treated metastatic colorectal cancer | ORR: 20% with perifosine + capecitabine vs ORR: 7% with placebo + capecitabine. OS: 17.7 months with the combination vs OS: 10.9 months with placebo + capecitabine. Median time to progression: 27.5 weeks with the combination vs 10.1 weeks with placebo + capecitabine | A | C | 2006 | * | 2018 | AEterna Zentaris | * | NCT00398879 | 386 |
| **TAS-117** (TAS 117) | | | | | | | | | | | | |
| **Phase** | **Treatment** | **Disease** | **Outcome** | **ISP** | **Status** | **FP** | **RFP** | **LUP** | **Sponsor** | **Collaborator** | **NCT Identifier** | **R** |
| 2 | TAS117 | PI3K-mutated and/or AKT-mutated solid tumors refractory to chemotherapy | ORR: 8%, DCR: 23%, PFS: 1.4 months, and OS: 4.8 months. Particular clinical efficacy was detected in PIK3CA E545K-mutated ovarian cancer, in PIK3CA H1047R-mutated breast cancer, and in AKT1 E17K-mutated breast cancer | A | C | 2017 | * | 2020 | Yonsei University | * | NCT03017521 | 389 |
| **Uprosertib** (GSK2141795) | | | | | | | | | | | | |
| **Phase** | **Treatment** | **Disease** | **Outcome** | **ISP** | **Status** | **FP** | **RFP** | **LUP** | **Sponsor** | **Collaborator** | **NCT Identifier** | **R** |
| 1 | Uprosertib | Advanced solid tumour | PR: 1.5% (anal cancer), SD (≥ 6 months): 3% (endometrial cancer), SD (≥ 6 months): 3% (prostate cancer) | F | C | 2009 | * | 2017 | GlaxoSmithKline | * | NCT00920257 | 391 |
