# Supplementary material for: PI3K/AKT/mTOR signaling transduction pathway and targeted therapies in cancer
Source: Mol Cancer. 2023 Aug 18;22:138. doi: 10.1186/s12943-023-01827-6 (PMC10436543; doi:10.1186/s12943-023-01827-6)
Supplement: Supplementary file 7 — Additional file 7: Supplementary table 3. [file 12943_2023_1827_MOESM7_ESM.docx]

| **ALLOSTERIC mTOR INHIBITORS** | | | | | | | | | | | | |
| --- | --- | --- | --- | --- | --- | --- | --- | --- | --- | --- | --- | --- |
| **Everolimus** (RAD001, SDZ-RAD) | | | | | | | | | | | | |
| **Phase** | **Treatment** | **Disease** | **Outcome** | **ISP** | **Status** | **FP** | **RFP** | **LUP** | **Sponsor** | **Collaborator** | **NCT Identifier** | **R** |
| 1 | Triple combination of everolimus with letrozole and trastuzumab | HR+ metastatic breast cancer | PR: 19.2%. RP2D: 10 mg/once daily | F | C | 2014 | * | 2020 | M.D. Anderson Cancer Center | National Cancer Institute (NCI) | NCT02152943 | 400 |
| 1b | Triple combination of everolimus with alpelisib and exemestane | Advanced solid tumor | 16-week PFS rate: 52.4% (renal cell carcinoma). MTD: 2.5 mg/daily | A | C | 2014 | * | 2020 | Novartis | * | NCT02077933 | 96 |
| 1, 2 | Combination of everolimus with ribociclib | Recurrent or refractory advanced malignancies | SD: 41.2%. RP2D: 2.5 mg/m^2^ once daily | F | A, R | 2016 | * | 2021 | Gustave Roussy, Cancer Campus, Paris | National Cancer Institute, France | NCT02813135 | 401 |
| 1, 2 | Triple combination of everolimus with exemestane and ribociclib | Advanced breast cancer after progression on CDK4/6 inhibitors | CBR (at week 24): 41.1%. RP2D: 2.5 mg/once daily | F | C | 2016 | 2021 | 2021 | Novartis | * | NCT02732119 | 402 |
| 1b, 2 | Triple combination of everolimus with exemestane and xentuzumab | HR+, HER2- locally advanced breast cancer | PFS: 7.3 months with the triple combination vs PFS: 5.6 months with everolimus + exemestane. MTD: 10 mg/daily, with exemestane 25 mg/daily, and xentuzumab 1000 mg/weekly | A | C | 2014 | * | 2022 | Boehringer Ingelheim | * | NCT02123823 | 403 |
| 2 | Everolimus | Advanced biliary tract cancer | DCR (at 12 weeks): 48%, PFS: 5.5 months, and OS: 9.5 months | F | U | 2009 | * | 2009 | Austin Health | * | NCT00973713 | 404 |
| 2 | Everolimus | Papillary metastatic renal cell carcinoma | PFS (at 6 months): 34%, SD: 65%, and OS: 21.4 months | F | C | 2008 | 2015 | 2016 | Novartis | * | NCT00688753 | 405 |
| 2 | Combination of everolimus with lenvatinib | Metastatic renal cell carcinoma | PFS: 14.6 months with everolimus + lenvatinib vs PFS: 5.5 months with everolimus alone | A | C | 2010 | 2019 | 2019 | Eisai Inc. | * | NCT01136733 | 399 |
| 2 | Combination of everolimus with exemestane | HR+, HER2- advanced breast cancer | PFS: 8.4 months with everolimus + exemestane vs PFS: 6.8 months with everolimus alone. The estimated reduction of disease progression or death with the combination was 26%, compared to everolimus alone | A | C | 2013 | 2021 | 2021 | Novartis | * | NCT01783444 | 406 |
| 2 | Combination of everolimus with letrozole | HR+, HER2- advanced breast cancer | PFS: 19.4 months with everolimus + letrozole vs PFS: 12.9 months with letrozole singularly | A | U | 2014 | * | 2014 | Chinese Academy of Medical Sciences | Novartis | NCT02313051 | 407 |
| 2 | Combination of everolimus with letrozole | Recurrent endometrial carcinoma | CBR: 40%, ORR: 32% | F | A, NR | 2010 | * | 2022 | M.D. Anderson Cancer Center | Novartis | NCT01068249 | 408 |
| 2 | Combination of everolimus with paclitaxel | Cisplatin-ineligible advanced urothelial carcinoma | CBR (at 4 months): 37.9% with everolimus + paclitaxel vs CBR (at 4 months): 0% with everolimus alone. PFS: 5.85 months with the combination vs PFS: 2.33 months with everolimus alone | A | T | 2010 | * | 2019 | Hoosier Cancer Research Network | Novartis | NCT01215136 | 409 |
| 2 | Triple combination of everolimus with letrozole and metformin | Advanced or recurrent endometrioid endometrial cancer | CBR: 50%, PR 28%, SD: 22%, PFS: 5.7 months, and OS: 19.6 months. Positive progesterone receptor expression was associated with CBR (89.5% vs 27.3%) | A | A, NR | 2013 | * | 2022 | M.D. Anderson Cancer Center | Novartis Pharmaceuticals, and National Cancer Institute (NCI) | NCT01797523 | 410 |
| 3 | Everolimus | Advanced pancreatic neuroendocrine tumor | PFS: 11 months with everolimus vs PFS: 4.6 months with placebo. Estimates of the proportion of patients who were alive and progression-free at 18 months were 34% with everolimus vs 9% with placebo | A | C | 2007 | 2011 | 2015 | Novartis Pharmaceuticals | * | NCT00510068 | 411 |
| 3 | Everolimus | Advanced, non-functional neuroendocrine tumours of lung or gastrointestinal tract | PFS: 11 months with everolimus vs PFS: 3.9 months with placebo. Everolimus was associated with a 52% reduction in the estimated risk of disease progression or death vs placebo (28%) | A | C | 2012 | 2016 | 2021 | Novartis Pharmaceuticals | * | NCT01524783 | 412 |
| 3 | Everolimus | Advanced, progressive, well-differentiated, non-functional neuroendocrine tumor | Tumor shrinkage: 58% with everolimus vs 13% with placebo. PFS: 9.2 months with everolimus arm vs PFS: 3.6 months with placebo | A | C | 2012 | 2016 | 2021 | Novartis Pharmaceuticals | * | NCT01524783 | 413 |
| 3 | Everolimus | Previously-treated advanced metastatic renal cell carcinoma | PFS: 4 months with everolimus vs PFS: 1.9 months with placebo | A | C | 2006 | 2013 | 2013 | Novartis Pharmaceuticals | * | NCT00410124 | 414 |
| 3 | Everolimus | Renal TSC-associated angiomyolipoma | ORR: 42% with everolimus vs ORR: 0% with placebo | A | C | 2008 | 2012 | 2017 | Novartis Pharmaceuticals | * | NCT00790400 | 415 |
| 3 | Everolimus | TSC-associated SEGA | 50% reduction in the volume of SEGA with everolimus vs 0% reduction in the volume of SEGA with placebo | A | C | 2008 | 2012 | 2016 | Novartis Pharmaceuticals | * | NCT00789828 | 416 |
| 3 | Combination of everolimus with exemestane | Previously-treated advanced HR+, HER2- breast cancer | ORR: 9.5% with everolimus + aromatase exemestane vs ORR: 0.5% with placebo + exemestane. PFS: 6.9 months with the combination vs PFS: 2.8 months with placebo + exemestane (assessments by local investigators). PFS: 10.6 months with the combination vs PFS: 4.1 months with placebo + exemestane (central assessment) | A | C | 2009 | 2012 | 2017 | Novartis Pharmaceuticals | * | NCT00863655 | 417 |
| 3 | Combination of everolimus with exemestane | HR+, HER2- locally advanced or metastatic breast cancer | ORR: 8.9%, and PFS: 5.6 months | A | C | 2012 | * | 2017 | Novartis | * | NCT01626222 | 418 |
| 3 | Triple combination of everolimus with paclitaxel and trastuzumab | HR-, HER2+ advanced breast cancer | PFS: 25.4 months with everolimus + paclitaxel + trastuzumab vs PFS: 14.4 months with placebo + paclitaxel + trastuzumab | A | C | 2009 | 2018 | 2018 | Novartis | * | NCT00876395 | 419 |
| **Nab-sirolimus** (Nab-rapamycin, ABI-009) | | | | | | | | | | | | |
| **Phase** | **Treatment** | **Disease** | **Outcome** | **ISP** | **Status** | **FP** | **RFP** | **LUP** | **Sponsor** | **Collaborator** | **NCT Identifier** | **R** |
| 1 | Nab-sirolimus | Unresectable, advanced non-hematological malignancies | PR: 3.8% in kidney cancer patients. MTD: 100 mg/m^2^ weekly | F | C | 2008 | * | 2019 | Celgene | * | NCT00635284 | 423 |
| 2 | Nab-sirolimus | Malignant perivascular epithelioid cell tumor | ORR: 39%, PR: 35.4%, and CR: 3.2%. SD: 52%, PFS: 10.6 months, and OS: 40.8 months | A | C | 2015 | * | 2022 | Aadi Bioscience Inc | * | NCT02494570 | 424 |
| **Rapamycin** (Sirolimus, AY-22989, I-2190A) | | | | | | | | | | | | |
| **Phase** | **Treatment** | **Disease** | **Outcome** | **ISP** | **Status** | **FP** | **RFP** | **LUP** | **Sponsor** | **Collaborator** | **NCT Identifier** | **R** |
| 1 | Rapamycin | Recurrent PTEN-deficient glioblastoma | Tumor cell proliferation (Ki-67 staining) was dramatically reduced in 50% of patients after a week treatment, and was associated with the magnitude of mTOR inhibition | F | C | 2003 | * | 2020 | Jonsson Comprehensive Cancer Center | * | NCT00047073 | 434 |
| 1 | Rapamycin | Advanced cancer | SD: 40%. Significant inhibition of S6K1 kinase phosphorylation. The target rapamycin AUC of 3,810 ng-h/mL was reached at 90 mg/once weekly | A | C | 2008 | * | 2014 | University of Chicago | National Institutes of Health (NIH) | NCT00707135 NCT00708591 | 435 |
| 1 | Combination of rapamycin with ketoconazole | Advanced cancer | SD: 28%. Significant inhibition of S6K1 kinase phosphorylation. The target rapamycin AUC of 3,810 ng-h/mL was reached at 16 mg/once weekly | A | C | 2008 | * | 2014 | University of Chicago | National Institutes of Health (NIH) | NCT00707135 NCT00708591 | 435 |
| **Ridaforolimus** (AP23573, MK-8669, Deforolimus) | | | | | | | | | | | | |
| **Phase** | **Treatment** | **Disease** | **Outcome** | **ISP** | **Status** | **FP** | **RFP** | **LUP** | **Sponsor** | **Collaborator** | **NCT Identifier** | **R** |
| 1 | Ridaforolimus | Advanced solid tumor | SD: 10%. RP2D: 33 mg/m^2^ (5 days/week). Preliminary pharmacokinetic data suggest that 28 mg/m^2^ (5 days/week) may also be considered | F | T | 2011 | 2011 | 2019 | Merck Sharp & Dohme LLC | * | NCT01431534 NCT01431547 | 438 |
| 1 | Combination of ridaforolimus and dalotuzumab | Advanced cancer | ORR: 7%, and SD: 46%. PFS (> 4 months): 17% | A | C | 2008 | * | 2015 | Merck Sharp & Dohme LLC | Ariad Pharmaceuticals | NCT00730379 | 439 |
| 1 | Combining ridaforolimus and MK-0752 | Advanced solid tumor | ORR: 13.3%, and SD: (≥ 6 months): 6.6%. MTD: 20 mg/once daily (5 days/week) | A | C | 2011 | * | 2015 | Merck Sharp & Dohme LLC | * | NCT01295632 | 440 |
| 1 | Triple combination of ridaforolimus with paclitaxel and carboplatin | Solid tumor | RP2D: ridaforolimus 30 mg (days 1-5 and days 8-12) with day 1 paclitaxel (175 mg/m^2^) and carboplatin (AUC 5 mg/mL/min) on a 3-week cycle | A | C | 2010 | * | 2017 | H. Lee Moffitt Cancer Center and Research Institute | Merck Sharp & Dohme LLC | NCT01256268 | 441 |
| 1, 2 | Ridaforolimus | Refractory or advanced malignancies | CBR: 24.5%, PR: 1.4%, SD (≥8 weeks: 45.6%, OS: 37.7 weeks, and PFS: 12.1 weeks. RP2D: 40 mg/once daily (5 days/week) | A | C | 2005 | * | 2015 | Merck Sharp & Dohme LLC | Ariad Pharmaceuticals | NCT00112372 | 442 |
| 2 | Ridaforolimus | Advanced endometrial cancer | CBR: 29%, PR: 11%, SD: 18%, and 6-month PFS: 18% | A | C | 2005 | * | 2015 | Merck Sharp & Dohme LLC | Ariad Pharmaceuticals | NCT00122343 | 443 |
| 2b | Combination of ridaforolimus with trastuzumab | HER2+ trastuzumab-refractory metastatic breast cancer | PR: 15%, DOR: 19.1 weeks, SD: 41%, CBR: 34.3%, PFS: 5.4 months, and PFS rate (at 6 months): 37%. OS: 17.7 months. | F | C | 2008 | * | 2015 | Merck Sharp & Dohme LLC | * | NCT00736970 | 444 |
| 3 | Ridaforolimus | Metastatic soft-tissue sarcoma | PFS: 17.7 weeks with ridaforolimusvs vs PFS: 14.6 weeks with placebo. OS: 90.6 weeks with ridaforolimus vs OS: 85.3 weeks with placebo | A | C | 2007 | * | 2015 | Merck Sharp & Dohme LLC | Ariad Pharmaceuticals | NCT00538239 | 445 |
| **Temsirolimus** (CCI-779, Torisel) | | | | | | | | | | | | |
| **Phase** | **Treatment** | **Disease** | **Outcome** | **ISP** | **Status** | **FP** | **RFP** | **LUP** | **Sponsor** | **Collaborator** | **NCT Identifier** | **R** |
| 1 | Combination of temsirolimus with perifosine | Heavily pretreated recurrent malignant glioma | PR: 3.4%, SD: 44.8%, OS: 10.4 months, and PFS: 2.7 months. Median follow‐up amongst survivors was 8.9 months. | A | C | 2010 | 2016 | 2021 | National Cancer Institute (NCI) | * | NCT01051557 | 383 |
| 1 | Combination of temsirolimus with capecitabine | Advanced solid tumor | PR: 2.6%, SD: 50%, and DCR: 52%. RP2D: 25 mg/weekly | A | C | 2010 | * | 2014 | Georgetown University | Pfizer | NCT01050985 | 452 |
| 1 | Triple combination of temsirolimus with etoposide and cyclophosphamide | Relapsed or refractory acute lymphoblastic leukemia | ORR: 47%, and CR: 27%. Pharmacodynamic studies exhibited a dose-dependent inhibition of PI3K/AKT/mTOR pathway phosphoproteins | A | C | 2012 | * | 2020 | Therapeutic Advances in Childhood Leukemia Consortium | Pfizer | NCT01614197 | 453 |
| 1 | Triple combination of temsirolimus with rituximab and cladribine | Mantle cell lymphoma | ORR: 94%, PR: 41%, and CR: 53%. PFS: 18.7 months | A | C | 2008 | * | 2018 | Alliance for Clinical Trials in Oncology | National Cancer Institute (NCI) | NCT00787969 | 454 |
| 1, 2 | Combination of temsirolimus with lenalidomide | Relapsed and refractory lymphoma | ORR: 26%, and CR: 13% CR (diffuse large B-cell lymphoma). ORR: 64%, and CR: 18% (other lymphoma histologies with classical Hodgkin lymphoma). RP2D: 25 mg/weekly | A | C | 2010 | 2019 | 2019 | National Cancer Institute (NCI) | * | NCT01076543 | 455 |
| 2 | Temsirolimus | Relapsed or refractory primary CNS lymphoma | ORR: 54%, and PFS: 2.1 months | A | U | 2009 | * | 2013 | Charite University, Berlin, Germany | Pfizer | NCT00942747 | 456 |
| 2 | Combination of temsirolimus with bevacizumab | Pancreatic neuroendocrine tumor | ORR: 41%. PFS: 13.2 months, and PFS (at 6 months): 79%. OS: 34 months | A | C | 2009 | 2019 | 2019 | National Cancer Institute (NCI) | * | NCT01010126 | 457 |
| 2 | Combination of temsirolimus with chemotherapy | First relapse rhabdomyosarcoma | ORR: 47%. 6-month event-free survival (EFS): 69.1% | A | C | 2010 | 2017 | 2017 | National Cancer Institute (NCI) | * | NCT01222715 | 458 |
| 3 | Temsirolimus | Advanced metastatic renal cell carcinoma | OS: 10.9 months with temsirolimus vs OS: 7.3 months with IFNα. ORR: 8.6 months with temsirolimus vs ORR: 4.8 months with IFNα. PFS: 3.8 months with temsirolimus vs 1.9 with IFNα (site investigators). PFS: 5.5 months with temsirolimus vs 3.1 with IFNα (independent radiologic assessments). | A | C | 2003 | 2012 | 2012 | Pfizer | * | NCT00065468 | 392 |
| 3 | Temsirolimus | Relapsed or refractory mantle-cell lymphoma | PFS: 6.2 months. DOR: 3 months. ORR: 40% (independent review committee). ORR: 46% (investigator). OS: 21.3. 1-year survival rate: 61% | A | C | 2012 | 2017 | 2018 | Janssen Research & Development, LLC | Pharmacyclics LLC | NCT01646021 | 459 |
| **ATP-COMPETITIVE mTOR INHIBITORS** | | | | | | | | | | | | |
| **AZD8055** (R339J08R6U) | | | | | | | | | | | | |
| **Phase** | **Treatment** | **Disease** | **Outcome** | **ISP** | **Status** | **FP** | **RFP** | **LUP** | **Sponsor** | **Collaborator** | **NCT Identifier** | **R** |
| 1 | AZD8055 | Advanced solid tumor | SD (≥ 4 months): 14.2 months. MTD: 90 mg twice/daily. AZD8055 was rapidly absorbed (median Tmax ∼0.5 h), and exposure increased with increasing doses | A | C | 2008 | * | 2012 | AstraZeneca | * | NCT00731263 | 467 |
| 1 | AZD8055 | Advanced solid tumor | SD: 11.7%. AZD8055 determined a significant decrease of pAKT and p4EBP1 | A | C | 2009 | * | 2011 | AstraZeneca | * | NCT00973076 | 468 |
| **Onatasertib** (CC-223, ATG-008) | | | | | | | | | | | | |
| **Phase** | **Treatment** | **Disease** | **Outcome** | **ISP** | **Status** | **FP** | **RFP** | **LUP** | **Sponsor** | **Collaborator** | **NCT Identifier** | **R** |
| 1 | Onatasertib | Advanced solid tumor | PR: 3.5%, SD: 28.5%, and DCR: 32%. MTD: 45 mg/daily. Phosphorylation of mTORC1/mTORC2 pathway biomarkers in blood cells was inhibited with an exposure-response relationship | A | C | 2010 | * | 2017 | Celgene | * | NCT01177397 | 471 |
| 2 | Onatasertib | Non-pancreatic neuroendocrine cancer | PR: 7%, SD: 83%, DCR was 90%, and PFS: 19.5 months. | A | C | 2010 | * | 2017 | Celgene | * | NCT01177397 | 472 |
| **OSI‑027** (ASP4786, CERC 006, AEVI-006) | | | | | | | | | | | | |
| **Phase** | **Treatment** | **Disease** | **Outcome** | **ISP** | **Status** | **FP** | **RFP** | **LUP** | **Sponsor** | **Collaborator** | **NCT Identifier** | **R** |
| 1 | OSI-027 | Advanced lymphomas or advanced solid tumor | SD at the first follow-up radiological assessment (8-12 weeks): 29.7%. SD (> 6 months): 5%. MTD: 30 mg/daily | A | C | 2008 | * | 2013 | Astellas Pharma Inc | * | NCT00698243 | 478 |
| **Sapanisertib** (TAK-228, MLN0128, INK128) | | | | | | | | | | | | |
| **Phase** | **Treatment** | **Disease** | **Outcome** | **ISP** | **Status** | **FP** | **RFP** | **LUP** | **Sponsor** | **Collaborator** | **NCT Identifier** | **R** |
| 1 | Sapanisertib | Relapsed or refractory multiple myeloma, non-Hodgkin lymphoma, and Waldenström's macroglobulinemia | MR: 3%, and SD: 42.4% (multiple myeloma). PR: 3%, MR: 3%, and SD: 6% (Waldenström's macroglobulinemia). SD: 6% (non-Hodgkin lymphoma). MTDs: 4 mg/once daily, or 9 mg/once daily (3 days on and 4 days off each week), in 28-day cycles | A | C | 2010 | * | 2013 | Millennium Pharmaceuticals, Inc | * | NCT01118689 | 483 |
| 1 | Sapanisertib | Advanced nonhematological malignancies | SD (≥ 6 months): 10.7%, CBR: 45% (3 mg/once daily) or 67% (20 mg/once weekly). RP2D: 3 mg/once daily (East Asian patients), and 4 mg/once daily (Western patients) | F | T | 2017 | 2020 | 2021 | Millennium Pharmaceuticals, Inc | * | NCT03370302 | 484 |
| 1 | Sapanisertib | Advanced solid tumour | MTDs: 6 mg/once daily, 40 mg/once weekly, 9 mg/once daily for 3 days on/4 days off, and 7 mg/once daily for 5 days on/2 days off. Pharmacokinetics were time-linear and supported multiple dosing. Pharmacodynamic findings demonstrated treatment-related reductions in mTORC1/mTORC2 biomarkers | A | C | 2010 | 2020 | 2020 | Millennium Pharmaceuticals, Inc | * | NCT01058707 | 485 |
| 1 | Sapanisertib | Advanced solid tumour | ORR: 12%, and PR: 3.2%. MTD: 3 mg/once daily | F | C | 2015 | 2020 | 2020 | Millennium Pharmaceuticals, Inc | * | NCT02412722 | 486 |
| 1 | Combination of sapanisertib with paclitaxel | Advanced solid tumour | ORR: 18%, PR: 4.9%, and CR: 1.6%. MTD: 6 mg 3 days/week | F | C | 2015 | 2020 | 2020 | Millennium Pharmaceuticals, Inc | * | NCT02412722 | 486 |
| 1 | Combination of sapanisertib with metformin | Advanced solid tumour | PR: 13.3% (9.9% in PTEN-mutated tumors), SD: 46.6%, and DCR: 60% | A | A, NR | 2017 | * | 2022 | M.D. Anderson Cancer Center | National Cancer Institute (NCI) | NCT03017833 | 487 |
| 1 | Triple combination of sapanisertib with serabelisib and paclitaxel | Pretreated-advanced solid tumour | ORR: 47%, CBR: 73%, PFS: 11 months, and OS: 17 months. PR: 21%, CR: 15.7%, and SD (> 6 months): 21%. RP2D is 3 mg on days 2-4, 9-11, 16-18 and 23-25 | F | A, NR | 2017 | * | 2021 | Avera McKennan Hospital & University Health Center | * | NCT03154294 | 114 |
| 1, 2 | Combination of sapanisertib with exemestane or fulvestrant | HR+, HER2- advanced or metastatic breast cancer | CBR (at 16 weeks): 45% (pretreated everolimus-sensitive breast cancer), and 23% (pretreated everolimus-resistant breast cancer). ORR: 8% (pretreated everolimus-sensitive breast cancer), and 2% (pretreated everolimus-resistant breast cancer). MTD: 4 mg/daily in combination with exemestane or fulvestrant. | F | C | 2014 | 2020 | 2021 | Millennium Pharmaceuticals, Inc | * | NCT02049957 | 488 |
| 2 | Sapanisertib | Refractory metastatic renal cell carcinoma | ORR: 5.3%, and PFS: 2.5 months. PFS: 1.9 months (tumors with diminished or loss of PTEN expression) vs PFS: 3.7 months (tumors with intact PTEN expression) | A | A, NR | 2017 | 2022 | 2022 | Dana-Farber Cancer Institute | Calithera Biosciences Inc | NCT03097328 | 489 |
| 2 | Combination of sapanisertib with tamoxifen | HR+ breast cancer | Significant reduction in Ki-67 (from 18.3% to 15.2%), and tumor size (median decrease of 0.75 cm) | F | C | 2016 | 2021 | 2021 | The Methodist Hospital Research Institute | Millennium Pharmaceuticals, Inc | NCT02988986 | 490 |
| 2 | Combination of sapanisertib with fulvestrant | ER+, HER2- advanced breast cancer after progression on aromatase Inhibitor | PFS: 7.2 months with sapanisertib + fulvestrant vs PFS: 3.5 months with fulvestrant alone. The longest PFS benefit was observed in patients who had previously received CDK4/CDK6 inhibitors | A | C | 2016 | 2020 | 2020 | Millennium Pharmaceuticals, Inc | * | NCT02756364 | 491 |
| **Vistusertib** (AZD2014) | | | | | | | | | | | | |
| **Phase** | **Treatment** | **Disease** | **Outcome** | **ISP** | **Status** | **FP** | **RFP** | **LUP** | **Sponsor** | **Collaborator** | **NCT Identifier** | **R** |
| 1 | Combination of vistusertib with temozolomide | Previously-treated glioblastoma multiforme | PR: 8%, DOR: 7.6 months, and SD: 38%. 6-month-PFS rate: 26.6% | F | C | 2015 | * | 2020 | Canadian Cancer Trials Group | AstraZeneca | NCT02619864 | 508 |
| 1 | Combination of vistusertib with paclitaxel | High-grade serous ovarian, and squamous NSCLC | ORR: 52%, and PFS: 5.8 months (high-grade serous ovarian cancer). ORR: 35%, and PFS: 5.8 months (squamous NSCLC). RP2D: 80 mg/m^2^ twice daily for 7 weeks | F | C | 2014 | * | 2020 | Royal Marsden NHS Foundation Trust | Institute of Cancer Research UK, and AstraZeneca | NCT02193633 | 509 |
| 1, 2 | Combination of vistusertib with anastrozole | Recurrent or metastatic HR+ endometrial cancer | ORR: 24.5% with vistusertib + anastrozole vs ORR: 17.4% with anastrozole alone. PFS: 5.2 months with vistusertib + anastrozole vs PFS: 1.9 months with anastrozole alone | A | A, NR | 2016 | * | 2022 | Centre Leon Berard | * | NCT02730923 | 510 |
| 2 | Vistusertib | Relapsed or refractory diffuse large B-cell lymphoma | PR: 6.6%, PFS: 1.6 months, OS: 6.5 months, and DOR: 153 days | A | C | 2016 | * | 2020 | University of Birmingham | Bloodwise, AstraZeneca, and Cancer Research UK | NCT02752204 | 511 |
| 2 | Combination of vistusertib with fulvestrant | HR+ metastatic breast cancer | PFS: 7.6 months with vistusertib + fulvestrant vs PFS: 5.4 months with fulvestrant alone. | A | U | 2015 | * | 2020 | Queen Mary University of London | AstraZeneca | NCT02216786 | 512 |
| **BI-STERIC mTOR INHIBITORS** | | | | | | | | | | | | |
| **RMC-5552** (RMC 5552) | | | | | | | | | | | | |
| **Phase** | **Treatment** | **Disease** | **Outcome** | **ISP** | **Status** | **FP** | **RFP** | **LUP** | **Sponsor** | **Collaborator** | **NCT Identifier** | **R** |
| 1, 1b | Combination of RMC-5552 Ras inhibitors | Relapsed or refractory Ras-mutated solid tumor | ORR: 20% and SD: 60%. Five dose levels were used ranging from 1.6 mg to 12 mg/weekly | F | A, R | 2021 | * | 2023 | Revolution Medicines, Inc | * | NCT04774952 | 517 |
